# Effects of Lingonberry (*Vaccinium vitis-idaea* L.) Supplementation on Hepatic Gene Expression in High-Fat Diet Fed Mice

**DOI:** 10.3390/nu13113693

**Published:** 2021-10-21

**Authors:** Riitta Ryyti, Antti Pemmari, Rainer Peltola, Mari Hämäläinen, Eeva Moilanen

**Affiliations:** 1 The Immunopharmacology Research Group, Faculty of Medicine and Health Technology, Tampere University and Tampere University Hospital, 33014 Tampere, Finland; riitta.ryyti@tuni.fi (R.R.); antti.pemmari@tuni.fi (A.P.); mari.hamalainen@tuni.fi (M.H.); 2 Natural Resources Institute Finland, Bioeconomy and Environment, 96200 Rovaniemi, Finland; rainer.peltola@luke.fi

**Keywords:** lingonberry, low-grade inflammation, gene expression, liver, high-fat diet, lipid metabolism, nonalcoholic fatty liver disease (NAFLD)

## Abstract

The prevalence of nonalcoholic fatty liver disease (NAFLD) is growing worldwide in association with Western-style diet and increasing obesity. Lingonberry (*Vaccinium vitis-idaea* L.) is rich in polyphenols and has been shown to attenuate adverse metabolic changes in obese liver. This paper investigated the effects of lingonberry supplementation on hepatic gene expression in high-fat diet induced obesity in a mouse model. C57BL/6N male mice were fed for six weeks with either a high-fat (HF) or low-fat (LF) diet (46% and 10% energy from fat, respectively) or HF diet supplemented with air-dried lingonberry powder (HF + LGB). HF diet induced a major phenotypic change in the liver, predominantly affecting genes involved in inflammation and in glucose and lipid metabolism. Lingonberry supplementation prevented the effect of HF diet on an array of genes (in total on 263 genes) associated particularly with lipid or glucose metabolic process (such as *Mogat1*, *Plin4*, *Igfbp2*), inflammatory/immune response or cell migration (such as *Lcn2*, *Saa1*, *Saa2*, *Cxcl14*, *Gcp1*, *S100a10*) and cell cycle regulation (such as *Cdkn1a*, *Tubb2a*, *Tubb6*). The present results suggest that lingonberry supplementation prevents HF diet-induced adverse changes in the liver that are known to predispose the development of NAFLD and its comorbidities. The findings encourage carrying out human intervention trials to confirm the results, with the aim of recommending the use of lingonberries as a part of healthy diet against obesity and its hepatic and metabolic comorbidities.

## 1. Introduction

Obesity is a constantly growing health problem worldwide [1]. In 2016, 39% of the global adult population was estimated to be overweight (body mass index BMI > 25 kg/m^2^), and 13% obese (BMI > 30) [2]. Importantly, obesity is a significant risk factor for severe metabolic disorders including insulin resistance, type 2 diabetes, cardiovascular diseases and nonalcoholic fatty liver disease (NAFLD) [3]. Obesity is associated with chronic low-grade inflammation induced by changes in adipose and hepatic tissues. The inflammatory state is known to contribute to the development of the adverse metabolic changes in overweight patients and may offer a treatment target for preventing the devastating co-morbidities associated with obesity [4,5,6].

Nonalcoholic fatty liver disease (NAFLD) is the most common liver disease worldwide with an estimated global prevalence of 30% in the general population, rising up to 90% in morbidly obese patients [7]. NAFLD is a condition where fat builds up in the liver without significant alcohol consumption, and it may proceed to severe liver disease [7,8]. The most important factor in the early development of NAFLD is insulin resistance, which accelerates fat breakdown in the adipose tissue increasing concentrations of circulating free fatty acids [9]. Therefore, NAFLD often coexists with type 2 diabetes in obese people. A diet rich in saturated fat and fructose, as well as physical inactivity are additional risk factors for NAFLD [10,11]. In obesity-driven NAFLD, liver injury proceeds by degrees and is associated with major changes in gene expression profile and cellular functions which are reflected in altered metabolic and inflammatory responses: In the beginning, circulating free fatty acids from enhanced lipolysis in the adipose tissue are taken up by the liver and accumulate in hepatocytes causing simple hepatic steatosis (also known as nonalcoholic fatty liver, NAFL). Lipid accumulation induces lipotoxicity and increased oxidative and endoplasmic reticulum stress in hepatocytes resulting in cell injury. Proinflammatory chemokines, cytokines and other factors released by injured hepatocytes and activated Kupffer cells trigger inflammation, which is further augmented by infiltrating inflammatory cells. This inflammatory phase is known as nonalcoholic steatohepatitis (NASH), and according to data based on paired liver biopsies, up to 40–50% of obese patients with simple hepatic steatosis may develop NASH [12]. The development of NASH is also aggravated by endotoxin, ethanol and other products derived from gut microbiome as well as by adipocytokines and other inflammatory factors released from obese adipose tissue. These changes together with inflammatory mediators and growth factors produced by activated Kupffer cells and infiltrated inflammatory cells in the liver induce chronic inflammation and fibrosis which may, in its most severe case, lead to cirrhosis of the liver or hepatocellular carcinoma [3,8,10,13]. Therefore, obesity-related fatty liver cannot be regarded as a benign disease, but serious attempts of its prevention are indicated by dietary and other interventions.

Prevention of low-grade inflammation with nutrition would be an effective means to prevent the development of insulin resistance and NAFLD [14,15,16,17]. For example, a Mediterranean diet rich in olive oil, vegetables and fruits was demonstrated to decrease liver fat content, increase insulin sensitivity and reduce circulating insulin concentration without changes in body weight in individuals with NAFLD [18]. Berries rich in polyphenolic compounds have shown promising effects in obesity-related metabolic adverse effects and low-grade inflammation in experimental models and in human studies [19,20,21]. Lingonberry is particularly rich in polyphenols and has a remarkable antioxidant activity; the most prevalent phenolic compounds in lingonberry are benzoic acid and its derivatives, flavanol oligomers (namely procyanidins), chlorogenic acid, quercetin derivatives and anthocyanins [22,23,24,25,26]. Recently, the authors of this study, and others, have reported that lingonberry supplementation has potential to inhibit high-fat diet-induced low-grade inflammation and adverse changes in glucose and lipid metabolism as well as visceral fat gain in a mouse model of obesity [27,28,29,30]. In the present study, the aim was to extend the previous data by investigating the effects of lingonberry supplementation on hepatic gene expression in high-fat diet-induced NAFLD in a mouse model of obesity.

## 2. Materials and Methods

### 2.1. Animals and Study Design

Male C57BL/6N mice (age 8 weeks and weight 24.3 ± 0.2 g at the beginning of the experiment) were fed for 6 weeks with low-fat (LF) diet (10 kcal% fat), with high-fat (HF) diet (46 kcal% fat) or with high-fat diet supplemented with air-dried lingonberry (*Vaccinium vitis-idaea* L.) powder (HF + LGB, 20% *w*/*w*). Finnish lingonberries were used to produce the air-dried lingonberry powder where approximately 900 g of fresh lingonberries were used to produce 100 g of berry powder (Kiantama Oy, Suomussalmi, Finland).

Compositions of the custom-made diets (Research Diets, Inc., New Brunswick, NJ, USA) are shown in the Supplementary Table S1. Nutrient composition of the air-dried lingonberry powder was taken into account, and all diets were matched for their protein, fiber and other ingredients, and high-fat diets also for carbohydrates and fat (Table S1).

Mice body weights were followed with weekly measurements, and at the end of the study blood samples from fasted mice were collected under anesthesia by cardiac puncture. Thereafter mice were euthanized by cervical dislocation and tissue samples were collected for further analyses. Mice were housed in the animal facility of the Tampere University under standard conditions (12 h light/dark cycle, temperature 22 ± 1 °C, humidity 50–60%) with food and water provided ad libitum. The study was approved by the National Animal Experimental Board, and the experiments were carried out in accordance with the EU legislation for the protection of animals used for scientific purposes (Directive 2010/63/EU).

Basic results on serum levels of cholesterol, triglycerides, glucose, insulin, adipokines and inflammatory factors of these mice have been published recently [27].

### 2.2. RNA Extraction

Liver samples were stored immediately after collection in RNA Later^®^ (Ambion, Thermo Fisher Scientific, Waltham, MA, USA). For RNA extraction, tissue (25–30 mg) was cut into smaller pieces and homogenized with Qiashredder (Qiagen). RNA was extracted with RNeasy Mini Kit (Qiagen Inc., Hilden, Germany) with on-column DNase digestion (Qiagen). RNA quantity and integrity were analyzed with TapeStation system (Agilent Technologies, Santa Clara, CA, USA).

### 2.3. Next-Generation Sequencing and Data Analysis

The RNA samples (n = 9 mice per group) were sequenced in Biomedicum Functional Genomics Unit, University of Helsinki, Finland using the Illumina NextSeq 500 system. Sequencing depth was 15 million 75 bp single-end reads. Read quality was assessed using FastQC [31], and the reads were trimmed using Trimmomatic [32]. Trimmed reads were then aligned to a reference mouse genome with STAR [33]. Count matrices were prepared with featureCounts [34]. Differential expression between the groups was determined using DESeq2 [35]. Genes with an average expression of at least 5 raw counts, a fold change (FC) 1.5 or greater and false discovery rate (FDR)-corrected *p*-value < 0.05 were deemed biologically and statistically significant and included in the further analyses. Mean expression levels were given as DESeq2-normalized counts. *p*-values were adjusted by false discovery rate (FDR).

Functional analysis of the differentially expressed genes was performed using the DAVID tool [36,37] with the Gene Ontology (GO) database [38,39] and the resulting list was reduced with REVIGO [40]. Protein-protein interactions were studied using STRING [41].

### 2.4. Reverse Transcription Polymerase Chain Reaction (RT-PCR)

For PCR validation, RNA was transcribed to cDNA (Maxima First Strand cDNA Synthesis Kit, Thermo Fisher Scientific) and subjected to quantitative PCR with TaqMan Universal Master Mix (Thermo Fisher Scientific) and ABI Prism 7500 sequence detection system (Applied Biosystems, Foster City, CA, USA) using the following TaqMan Gene Expression assays (Thermo Fisher Scientific); Mm00432403_m1 (*Cd36*), Mm03047343_m1 (*Cd68*), Mm00617672_m1 (*Cidec*), Mm00444699_m1 (*Cxcl14*), Mm00725580_s1 (*Cyp2c29*), Mm00472168_m1 (*Cyp2c55*), Mm00731567_m1 (*Cyp3a11*), Mm01607174_mH (*Cyp3a59*), Mm00487306_m1 (*Cyp46a1*), Mm00492632_m1 (*Igfbp2*), Mm00434228_m1 (*IL1b*), Mm00440181_m1 (*Lepr*), Mm00503358_m1 (*Mogat1*), Mm01184322_m1 (*Pparg*), Mm04208126_mH (*Saa2*), Mm00446229_m1 (*Slc2a2*), Mm00443260_g1 (*Tnfa*). QuantiTect Primer Assays (Qiagen) were used to measure *Pparg* variants 1 and 2 (QT00100296 and QT02266166, respectively). Primers and probe for the housekeeping gene glyceraldehyde 3-phosphate dehydrogenase *Gapdh*) were GCATGGCCTTCCGTGTTC (forward, 300 nM), GATGTCATCATACTTGGCAGGTTT (reverse, 300 nM) and TCGTGGATCTGACGTGCCGCC (probe, 150 nM) (Metabion GmbH, Planegg, Germany). Results were calculated using the delta-delta CT method, and all mRNA levels were normalized against GAPDH.

### 2.5. Statistical Analyses

The analysis of NGS data is described above. The other results are expressed as mean + SEM. One or two-way ANOVA with Bonferroni post-test was used in the statistical analysis. Differences were considered significant at *p* < 0.05. Data were analyzed using the Prism computerized package (Graph Pad Software, San Diego, CA, USA).

## 3. Results

### 3.1. Body and Liver Weights

**Figure 1:**
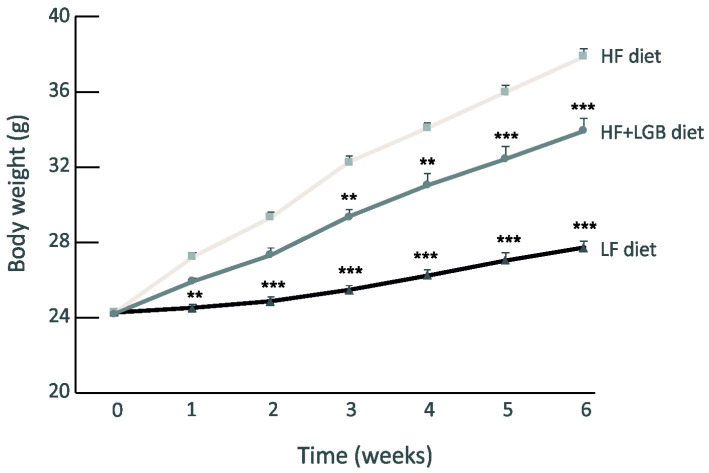
Body weight gain of the mice during the study. Animals received low-fat diet (LF diet, black line), high-fat diet (HF diet, light grey line) or high-fat diet supplemented with lingonberry (HF + LGB diet, grey line). Weight was measured once a week. The results are expressed as grams (g). Values represent mean + SEM, *n* = 9 mice per group. Two-way ANOVA with Bonferroni post-test was used in the statistical analysis. Mean values significantly different from the high-fat group (HF diet) are marked with ** = *p* < 0.01 and *** = *p* < 0.001.

In the high-fat (HF) diet group, the weight of mice increased consistently during the study when compared with the mice in the low-fat (LF) diet group. Notably, lingonberry supplementation (HF + LGB group) significantly prevented the high-fat diet-induced weight gain (*p* < 0.001 between the HF and HF + LGB groups). After 6 weeks, the average weight was 27.7 ± 0.3 g in the LF diet group, 37.9 ± 0.4 g in the HF group and 34.0 ± 0.7 g in the HF + LGB group. The weight gain in the three groups is presented in Figure 1.

Food consumption was measured weekly, and the cumulative food intake (kcal/g body weight/two mice cage) during the study did not differ between the HF (16.48 ± 0.19 kcal/g) and the lingonberry supplemented HF (16.44 ± 0.42 kcal/g) diet groups, although energy intake in the LF (14.45 ± 0.29 kcal/g, *p* < 0.01) diet group was somewhat lower.

**Figure 2:**
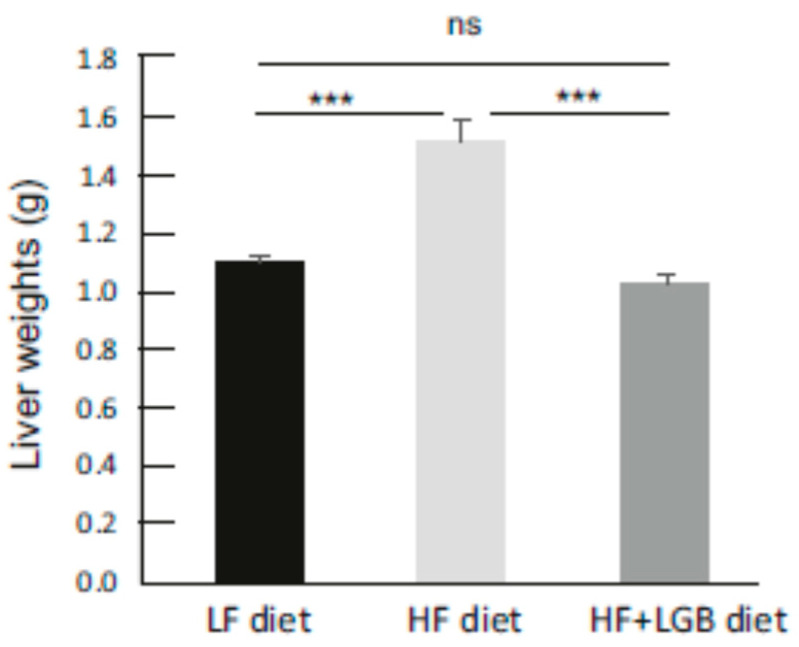
Liver weights of the mice at the end of the study. Animals received low-fat diet (LF diet, black column), high-fat diet (HF diet, light grey column) or high-fat diet supplemented with lingonberry (HF + LGB diet, grey column). The results are expressed as grams (g). Values represent mean + SEM, *n* = 9 mice per group. One-way ANOVA with Bonferroni post-test was used in the statistical analysis, *** = *p* < 0.001 and ns = not significant.

Liver weights of the mice were increased in the HF diet group (1.53 ± 0.07 g), and the difference was statistically significant when compared with the LF diet group (*p* < 0.001) and with the HF + LGB group (*p* < 0.001) (Figure 2). Interestingly, there was no difference between the LF and HF + LGB diet groups, the liver weights being 1.11 ± 0.03 g and 1.04 ± 0.03 g, respectively, suggesting that lingonberry supplementation prevents the liver weight gain induced by the HF diet. In addition, the circulating alanine aminotransferase (ALT) levels were measured. ALT activity in the serum was 8.2 ± 0.6 U/L in the LF diet group, 14.6 ± 0.7 U/L in the HF diet group and 7.2 ± 0.2 U/L in the HF + LGB group indicating that lingonberry supplementation totally prevented the high-fat diet-induced increase in the serum ALT activity (*p* < 0.001 between the HF and HF + LGB groups and *p* > 0.05 between HF + LGB and LF groups).

### 3.2. Changes in the Hepatic Gene Expression Caused by High-Fat Diet

In the HF diet group, 674 hepatic genes were upregulated in a statistically significant manner (FDR-corrected *p* < 0.05) when compared with the LF diet group, 102 of these with fold change (FC) > 1.5. Additionally, 578 genes were downregulated (FDR-corrected *p* < 0.05), 35 of these with FC < −1.5. Twenty most strongly up- and downregulated genes are presented in Table 1 and Table 2. Functions of these genes are linked particularly to lipid and cholesterol metabolism, inflammation, and cell adhesion. The most strongly downregulated gene was leptin receptor (*Lepr*), and many other robustly downregulated genes were also associated with glucose and lipid metabolism. A complete list of all significantly differentially expressed genes in the HF diet group compared with the LF diet group is provided in the Supplementary Table S2. For instance, the expression of the acute-phase inflammatory proteins serum amyloid A (*Saa*) 1 and 2, as well as the lipid metabolism and inflammation associated gene peroxisome proliferator activated receptor gamma (*Pparg*), were significantly upregulated, FC values being 1.65, 1.60, and 1.72, respectively (Table S2). Based on PCR analysis, the expression of both *Pparg* subtypes (variant 1 and variant 2) was increased in the HF diet group, variant 2 having more robust increase even though its expression was lower at the beginning. Accordingly, the expression of *Pparg* target genes (*Cd36*, *Cidec* and *Mogat1*) was increased (Table S7).

### 3.3. Differences in Hepatic Gene Expression between Lingonberry-Supplemented and Control High-Fat Diet Groups

The expression of 391 genes was lower in the HF + LGB diet group than in the HF diet group (FDR-corrected *p* < 0.05), with 66 genes with FC < −1.5. Functions of these genes include regulation of lipid metabolism, inflammation, cell proliferation and extracellular matrix assembly. As an example of the inflammatory genes, the expression of the acute phase inflammatory factors *Saa1* and *Saa2* was significantly lower in the HF + LGB diet group than in the HF diet group (Table 3).

In addition, the expression of 380 genes was higher in the HF + LGB diet group than in the HF diet group (FDR-corrected *p* < 0.05), with 27 genes with FC > 1.5. Functions of these most strongly upregulated genes are linked particularly to oxidation and reduction, fatty acid and amino acid metabolism, and response to bacteria and stilbenoid (Table 4). Accordingly, the expression of four cytochrome P450 enzymes was higher in mice fed with lingonberry supplemented HF diet than in the control HF diet group: *Cyp3a11* (FC 2.85), *Cyp2c55* (FC 2.22), *Cyp2c29* (FC 1.75) and *Cyp3a59* (FC 1.55), while the expression of *Cyp46a1* (FC −1.82) was lower in the HF + LGB diet group (Table S3). Hydroxysteroid (17-beta) dehydrogenase 6 (*Hsd17b6)* and insulin-like growth factor binding protein 2 (*Igfbp2*) are examples of other genes whose expression was higher in mice fed with HF + LGB than HF diet. A complete list of all significantly differentially expressed genes in the HF + LGB diet group compared with the HF diet group is provided in the Supplementary Table S4.

**Figure 3:**
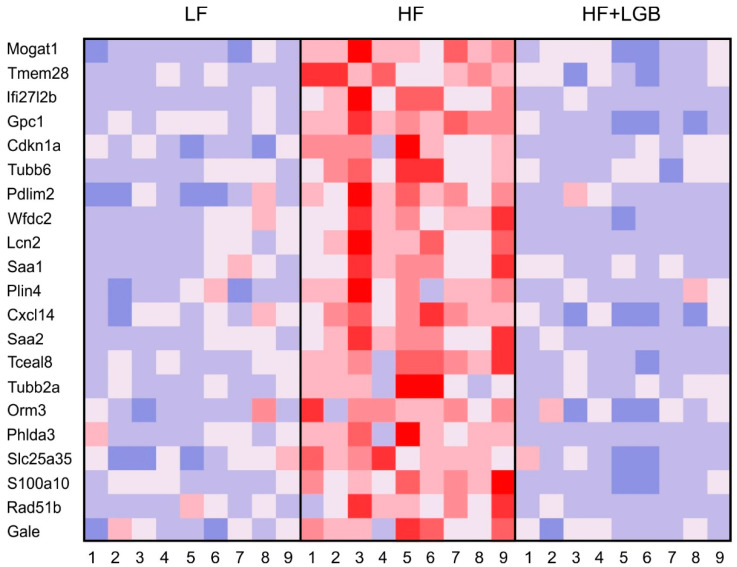
A heatmap of the genes upregulated by the HF diet (with an average fold change > 1.5 as compared with LF diet group) and whose increase was prevented by the HF + LGB diet (with an average fold change < −1.5 as compared with HF diet group). Gene expression levels are DESeq2-normalized and row-scaled; red color: higher expression; blue color: lower expression. N = 9 mice per group as indicated with the numbers on the horizontal axis.

Next, focus was on the genes which were up- or downregulated by HF diet, and the change was prevented when the diet was supplemented with lingonberry powder. There were in total 153 significantly (FDR-corrected *p* < 0.05) upregulated genes in the HF diet group, whose increase was prevented by lingonberry supplementation in a statistically significant manner. Respectively, there were, in total, 110 significantly (FDR-corrected *p* < 0.05) downregulated genes in the HF diet group whose decrease was prevented by lingonberry supplementation (Tables S5 and S6). Out of these genes, there were 23 genes with fold chain (FC) change > 1.5 or < −1.5 in both comparisons: twenty-one were upregulated by HF diet and the increase was prevented by lingonberry supplementation, whereas two genes were downregulated by HF diet and the decrease was prevented by HF + LGB diet (Table 5, Figure 3). When investigated at the functional level, lingonberry supplementation was found to prevent HF diet-induced upregulation of genes associated with lipid metabolic process (*Mogat1*, *Plin4*), inflammatory/immune response or cell migration (*Lcn2*, *Saa1*, *Saa2*, *Cxcl14*, *Gcp1*, *S100a10*), and cell cycle regulation (*Cdkn1a*, *Tubb2a*, *Tubb6*). Interestingly, lingonberry supplementation prevented the high-fat diet-induced downregulation of insulin-like growth factor binding protein 2 (*Igfbp2*). It is a gene with antidiabetic effects and may be involved in the development of glucose intolerance during HF diet (Table 5). The effects of HF diet and lingonberry supplementation on selected genes associated with inflammation and metabolism were confirmed with RT-PCR (Supplementary data, Table S7).

### 3.4. Functions and Interactions

The DAVID tool was used to perform a functional analysis on the differentially expressed genes. HF diet affected particularly “lipid metabolic process” (GO:0006629), “cellular lipid metabolic process” (GO:0044255) and “regulation of inflammatory response” (GO:0050727) when compared with the LF diet. All significantly differentially expressed functional categories (n = 5) between the HF and LF diet groups are presented in Table 6. Out of the HF vs. HF + LGB comparison, the most interesting functions relevant to the issue were selected for Table 6. Interesting biological processes affected by lingonberry supplementation were especially “lipid metabolic process” (GO:0006629), “response to stilbenoid” (GO:0035634), “carbohydrate metabolic process” (GO:0005975), “oxidation-reduction process” (GO:0055114) and “acute-phase response” (GO:0006953). All differentially expressed functional categories in HF vs. HF + LGB groups are presented in the Supplementary data in Table S8.

**Figure 4:**
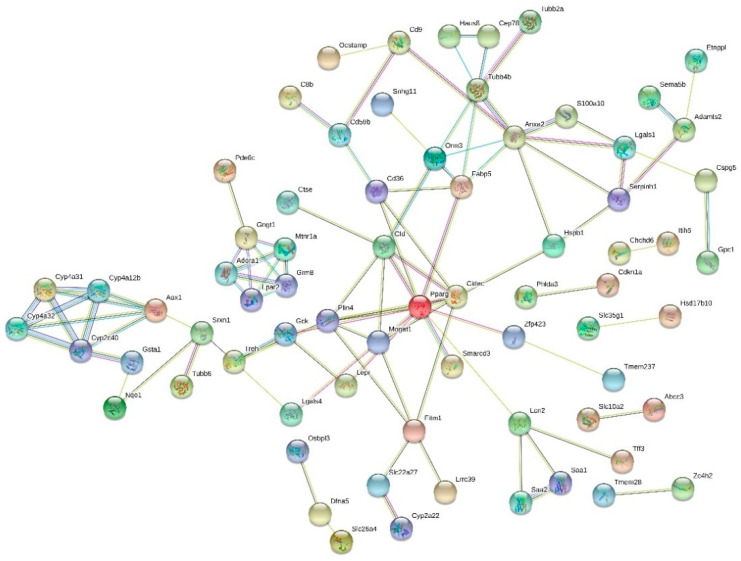
Interactions among the genes with greatest expression fold change in HF vs. LF groups. Genes with expression fold change (FC) > 1.5 or < −1.5 in high-fat (HF) vs. low-fat (LF) diet groups were studied with STRING. Genes with no identified interactions were excluded from the graph. Colors of the edges: green = activation, blue = binding, black = chemical reaction, red = inhibition, violet = catalysis, pink = posttranslational modification, yellow = transcriptional regulation, grey = other interaction.

Interactions between the protein products of the most strongly up- and downregulated (FC > 1.5 or < −1.5) genes were studied using the STRING tool. Notably strong and interesting interactions between HF vs. LF diet groups were the group of genes related to lipid metabolism/liver steatosis: peroxisome proliferator activated receptor gamma (*Pparg*), complement factor D (*Cfd*, also known as adipsin), monoacylglycerol O-acyltransferase 1 (*Mogat1*), cell death-inducing DFFA-like effector c *(Cidec*) and fatty acid binding protein 5 (*Fabp5*), the network of four cytochrome P450 enzymes (*Cyp2c40*, *Cyp4a12b*, *Cyp4a31* and *Cyp4a32*), and the network around annexin A2 (*Anxa2*) (Figure 4).

**Figure 5:**
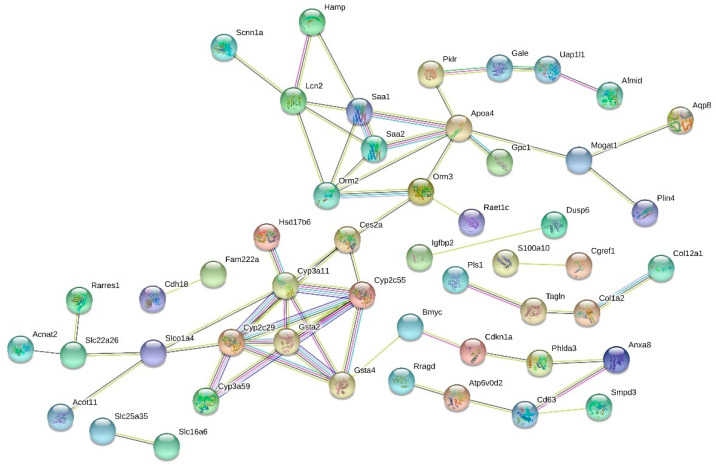
Interactions among the genes with greatest expression fold change in HF + LGB vs. HF groups. Genes with expression fold change (FC) > 1.5 or < −1.5 in high-fat diet supplemented with lingonberry (HF + LGB) vs. high-fat (HF) diet groups were studied with STRING. Genes with no identified interactions were excluded from the graph. Colors of the edges: green = activation, blue = binding, black = chemical reaction, red = inhibition, violet = catalysis, pink = posttranslational modification, yellow = transcriptional regulation, grey = other interaction.

When comparing the HF and HF + LGB diet groups, notable interactions were a connection of glutathione S-transferase alpha 2 (*Gsta2*) and glutathione S-transferase alpha 4 (*Gsta4*), cluster of four cytochrome P450 enzymes (*Cyp2c29*, *Cyp2c55*, *Cyp3a11* and *Cyp3a59*) as well as the group of apolipoprotein A-IV (*Apoa4*), serum amyloid A1 (*Saa1*) and A2 (*Saa2*) (Figure 5).

## 4. Discussion

The liver has a central role in the regulation of the metabolic homeostasis in the body. It synthesizes, stores and redistributes lipids, carbohydrates and proteins [44]. In obesity, excess fat accumulates in the liver inducing the development of nonalcoholic fatty liver disease associated with inflammation and disturbances in the hepatic metabolic performance [45]. The present study investigated the effects of lingonberry supplementation on hepatic gene expression in mice on the high-fat diet.

The high-fat diet per se had a major effect on the hepatic transcriptome. The expression of 1252 genes was altered in a statistically significant manner following high-fat diet intervention for six weeks. Functions of the differentially expressed genes were linked particularly to lipid and glucose metabolism and inflammation. The findings are consistent with previous studies in experimental models of high-fat diet-induced obesity [46,47,48,49].

Adipsin (*Cfd*), serum amyloid A1 and A2 (*Saa1*, *Saa2)* and peroxisome proliferator activated receptor gamma (*Pparg*) are examples of inflammation related genes which were significantly upregulated by high-fat diet. Adipsin is an adipokine also known as complement factor D which is involved in the activation of the alternative complement pathway. In the present data, hepatic adipsin expression was increased following the high-fat diet. The significant functional role of adipsin is underlined by the fact that it was also located in a central position in the STRING analysis. These findings support the role of complement activation in the pathogenesis of NAFLD as also discovered in biopsy studies [50].

The high-fat diet significantly upregulated the expression of peroxisome proliferator activated receptor gamma (*Pparg*), which is also supported by previous studies [45,51]. PPARγ is a transcription factor primarily expressed in adipose tissue where its activation improves insulin sensitivity, increases adipose tissue fat storing capacity and reduces inflammation. PPARγ has significant functions also in the liver: in hepatocytes, PPARγ promotes cellular uptake of free fatty acids and induces de novo lipogenesis thereby aggravating liver steatosis, whereas in Kupffer cells and in hepatic stellate cells PPARγ activation seems to be beneficial. In Kupffer cells PPARγ mediates anti-inflammatory effects by suppressing inflammatory gene expression and by polarizing M1 type Kupffer macrophages towards anti-inflammatory M2 phenotype. In hepatic stellate cells PPARγ activation inhibits fibrosis and other cirrhosis-promoting responses [52,53].

PPARγ has two isoforms, PPARγ1 and PPARγ2, encoded from a single gene using two separate promoters and alternative splicing [54]. Mouse PPARγ2 contains 30 additional amino acids at the N-terminal side. While the two PPARγ isoforms share the same DNA binding specificity, the PPARγ2 seems to have 5–10 -fold greater transcription activity than PPARγ1. Based on literature, PPARγ2 is considered the principal isoform in adipose tissue and in obese liver [54]; a greater increase was also found in the hepatic expression of *Pparg2* than *Pparg1* induced by the high-fat diet (Table S7). The functional significance of the increased *Pparg* expression by the high-fat diet in the current study is supported by enhanced expression of PPARγ target genes, such as monoacylglycerol O-acyltransferase 1 (*Mogat1*, FC 2.51, for synthesis of diacylglycerol), cluster of differentiation 36 (*CD36*, FC 1.73 for fatty acid uptake) and cell death-inducing DFFA-like effector c (*Cidec*, FC 1.72, for lipid droplet formation). Many of these were also located at central positions in the STRING analysis. Interestingly, PPARγ agonists (thiazolidinediones, TZDs) belong to the very few drugs that have shown promise in the treatment of NAFLD. They are insulin sensitizing drugs used in the treatment of diabetes, and their potential benefits in NAFLD lay on their effects on adipose and hepatic tissues [52,53,54].

Leptin receptor (*Lepr*) was the most strongly downregulated gene in the liver after high-fat feeding. Leptin is an adipokine known to regulate energy metabolism and appetite [55,56]. Circulating leptin levels are in strong positive correlation with BMI and the amount of adipose tissue; in developing obesity, leptin secretion increases and aims to resist weight gain [57]. Unfortunately, this physiological function of leptin often fails, and obesity is characterized and partly ensued by leptin resistance although circulating leptin levels remain highly increased [48,49]. Attenuation of leptin receptor signaling is a putative mechanism leading to leptin resistance [49]. In addition to the reduced expression of leptin receptor as seen in the present study, other mechanisms such as increased SOCS-3 expression [58,59,60] have been presented to contribute to leptin resistance. Serum leptin levels as measured in our previous study were significantly higher in the mice on the high-fat diet than in those in the low-fat diet group [27] suggesting that reduced *Lepr* expression is functionally associated with leptin resistance.

The present study found that lingonberry supplementation prevented high-fat diet-induced increase in body and liver weights and had major effects on hepatic transcriptome. Presumably the moderate effects of lingonberry supplementation on the weight gain are due to the constituents of lingonberry as there were no differences in the food/energy intake between the HF and HF + LGB groups. Lingonberries are rich in polyphenols and many of them, especially flavonoids, have been shown to prevent weight gain or to induce weight loss [61,62,63]. Several mechanisms of action have been proposed, particularly increased energy expenditure and modulation of lipid metabolism [61]. To support the latter, the current study found that lingonberry supplementation prevented the effects of HF diet on the expression of several hepatic genes related to lipid metabolism (see below). Reduced fat absorption and changes in the gut microbiome have also been suggested as possible mechanisms of action of polyphenols [61,63] and should be investigated in further studies. Significant differences were found between HF and HF + LGB groups in pathways involved in lipid and carbohydrate metabolism, insulin resistance, oxidation-reduction process and inflammation suggesting that lingonberry has potential to prevent metabolic adverse effects induced by developing obesity. The present transcriptome profiling extends previous findings in high-fat diet-induced obesity models in the mouse, where lingonberry has been reported to prevent liver triacylglycerol deposition and enhance insulin clearance, to downregulate acute-phase and inflammatory pathways in the liver, to activate liver Akt and AMPK pathways and to improve hepatic steatosis [19,29,64,65].

Particular interest was focused on genes which were up- or downregulated by the high-fat diet and the effect was prevented by lingonberry supplementation in a statistically significant manner. Many of those genes were associated with inflammation (*Saa1*, *Saa2*, *Lcn2*, *Cxcl14)* or lipid metabolism (*Mogat1*, *Plin4*).

Murine serum amyloid A (*Saa*) gene family is a cluster of five genes [66]. *Saa1*, *Saa2* and *Saa3* are rapidly inducible acute phase genes while *Saa4* is constitutively expressed. As seen in the present data, the expression of *Saa1* and *Saa2* is enhanced in the liver in high-fat diet fed mice, while *Saa3* is known to be expressed mainly in the adipose tissue [67]. SAA1 and SAA2 can induce the production of an array of inflammatory cytokines and chemotactic factors but they also regulate inflammatory responses and have pro-survival properties. SAA has complex interactions with lipids, particularly those associated with cholesterol transport and HDL formation linking it to the pathogenesis of atherosclerosis. In addition, SAA is involved in the pathogenesis of chronic inflammation, fibrosis and secondary amyloidosis [68]. As lingonberry supplementation prevented the high-fat diet-induced increase in the expression of *Saa1* and *Saa2* it may have beneficial effects resisting the development of various SAA-mediated pathologies.

Chemokine (C-X-C motif) ligand 14 (CXCL14) broadly modulates chemotaxis, differentiation and activation of inflammatory cells, particularly monocytes and dendritic cells, and it also has antimicrobial activity [69]. Interestingly, *Cxcl14* is highly expressed in experimental liver fibrosis with different etiologies, such as bile duct ligation, carbon tetrachloride or ethanol [70], and neutralization of CXCL14 was found to reduce carbon tetrachloride induced liver injury and steatosis in mice [71]. These data together with the present results suggest that *Cxcl14* is one of the genes involved in the high-fat diet-induced liver inflammation and fibrosis and its expression is prevented by lingonberry supplementation.

Lipocalin *Lnc2* is characterized as an adipokine whose expression is upregulated in the liver and adipose tissue in obese subjects and animal models [72,73,74]. It acts as a lipid chaperone inducing lipotoxicity and endothelial dysfunction in obese conditions, thus promoting vascular diseases [72]. It has also a role in the pathogenesis of obesity-associated insulin resistance [74] and regulation of adaptive thermogenesis in adipose tissue [75,76]. In the present study, the expression of *Lnc2* was significantly increased in the high-fat diet group when compared with the low-fat control group, while its expression was retained at a significantly lower level in the lingonberry group. This is an interesting finding which may partly explain the positive metabolic effects of lingonberry supplementation in obese conditions.

Lingonberry supplementation also prevented upregulation of genes involved in lipid metabolism, such as monoacylglycerol *O*-acyltransferase 1 (*Mogat1*). It is connected to triacylglycerol metabolism in the liver and fat absorption in the gastrointestinal tract, as well as to early onset of type 2 diabetes, hepatic steatosis and obesity. *Mogat1* is one of the enzymes converting monoacylglycerol to diacylglycerol, this phase being linked to the development of hepatic insulin resistance [77]. The expression of *Mogat1* in the liver has been shown to remarkably increase in high-fat diet fed mice models [77,78,79], and its expression is induced by obesity through direct activation of PPARγ [77].

Furthermore, lingonberry supplementation prevented upregulation of perilipin 4 (*Plin4*). Perilipins are involved in lipid droplet formation and contribute to the development of fatty liver disease where excessive lipid accumulates to hepatocytes [80]. *Plin4* is most highly expressed in adipose tissue and not detected in normal, healthy liver [81]. However, perilipin proteins are expressed in liver steatosis, and PLIN4 has been associated with increased PPARγ expression and hepatic lipid accumulation [82].

The expression of insulin-like growth factor binding protein 2 (*Igfbp2*) was downregulated by the high-fat diet and this effect was prevented by lingonberry supplementation. IGFBP2 has a significant role in systemic metabolism and as a treatment target in obesity and diabetes [83]. IGFBP2 is mainly synthesized in the liver. It stimulates glucose intake into adipocytes and enhances insulin sensitivity. In population-based studies IGFBP2 levels correlate inversely with insulin resistance [84], metabolic syndrome [85] and type 2 diabetes risk [86]. In experimental studies mice overexpressing *Igfbp2* have been reported to have lower susceptibility to develop obesity, insulin resistance and increased blood pressure [87]. Increased *Igfbp2* expression in mice on lingonberry supplemented high-fat diet is a likely mechanism involved in the improved glucose metabolism and reduced adiposity as compared with mice on control high-fat diet.

Cytochrome P450 enzymes (CYPs) are a group of monooxygenase enzymes significantly involved in lipid processing, fatty acid regulation, synthesis and breakdown of hormones and fat-soluble vitamins, and in clearance of various endogenous and exogenous compounds [88,89]. In the present study, both high-fat diet and lingonberry supplementation induced changes in the expression of CYP enzymes. An example is *Cyp3a11*, the expression of which was 2.85-fold in the HF + LGB group as compared with that in the HF group. In the mouse, CYP3a11 is linked to biological processes “oxidative demethylation” and “steroid metabolic process” [42,43]. Its expression has been shown to decrease in mice models of obesity and type 2 diabetes [90,91,92]. A similar decreasing trend by the high-fat diet was also seen in the present study, but it did not reach statistical significance during six weeks’ intervention. Since CYP3a11 in mice shares some properties of human CYP3A4 [93], further studies are needed to understand if lingonberry supplementation induces meaningful changes in drug metabolism per se or together with high-fat diet.

Smaller changes were detected in *Cyp2c29*, *Cyp2c55*, *Cyp3a59* and *Cyp46a1*, when their expression levels were compared between HF and HF + LGB groups (Table S3). *Cyp2c29* was expressed at rather high levels such as *Cyp3a11*, whereas the expression levels of the other three enzymes were lower. Recently, *Cyp2c29* was detected as a novel gene involved in liver injury and inflammation, and its overexpression was shown to protect against liver inflammation [94]. These findings support the favorable impact of lingonberry-induced increase in *Cyp2c29* expression found in the present study. *Cyp2c55* (also increased by lingonberry supplementation) is a target gene for nuclear receptor pregnane X (PXR), and is related to retinol metabolism and 19-HETE synthesis from arachidonic acid [95,96,97]. Whereas the roles of *Cyp3a59* (increased by lingonberry supplementation) and *Cyp46a1* (decreased by lingonberry supplementation) in the hepatic function or development of NAFLD remain less clear.

In the pathway analysis, “Activated response to stilbenoids” was an interesting pathway affected by lingonberry supplementation. It can thus be assumed that relevant amounts of lingonberry stilbenoids are absorbed from the gut and are functionally significant. Lingonberry contains rather high amounts of the stilbenoid resveratrol (3,4,5-trihydroxystilbene), mostly as trans-resveratrol or its glycosylated form [25,98,99]. Resveratrol has been reported to have protective effects in inflammation, oxidative stress and glucose intolerance [100,101,102,103,104,105], thus likely contributing to the beneficial effects of lingonberry supplementation found in the present study.

Similarly, other polyphenols present in lingonberry may also have positive metabolic effects. Polyphenol-rich cranberry extract was shown to reverse hepatic steatosis in mice fed with high-fat, high-sucrose diet independently of body weight loss. The cranberry extract used in that study contained similar polyphenols as lingonberry: anthocyanins and proanthocyanidins [106]. Likewise, polyphenol-rich cranberry extract and powder have been shown to attenuate hepatic inflammation and progression of NAFLD [20,107,108], and polyphenol-rich cherry extract to attenuate hepatic lipid accumulation and lower leptin concentrations when compared with high-fat control in murine models [109]. Moreover, quercetin has been shown to reduce liver fat accumulation and improve the metabolic status of high-fat diet fed mice, as well as to normalize the elevated expression of *Pparg*, a hepatic gene associated with steatosis and inflammation [51].

In conclusion, this paper has shown, for the first time, that air-dried lingonberry powder supplementation has beneficial effects on the adverse changes caused by high-fat diet in the liver, as measured by genome-wide expression analysis. The most interesting findings based on changes in the transcriptome and on the pathway analyses are connected to prevention of high-fat diet-induced low-grade inflammation and adverse effects on lipid and glucose metabolism. Further studies are needed to understand how these findings are translated into biochemical and metabolic changes in obesity; yet interestingly, our recent publication reported decreased serum levels of cholesterol, triglycerides, glucose, leptin and serum amyloid A in mice receiving lingonberry supplemented high-fat chow as compared with animals on control high fat diet [27]. Additional research is needed to explore the detailed mechanisms and effective compounds behind the detected effects of lingonberry supplementation.

## Figures and Tables

**Table 1:** The twenty most strongly upregulated genes in the high-fat (HF) diet group relative to the low-fat (LF) diet group. Mean expression levels are given as DESeq2-normalized counts. *p*-values are adjusted by false discovery rate (FDR).

Gene	Name	Functions in Mouse	Mean (LF)	Mean (HF)	Fold Change	*p*-Value (FDR adj.)
*Themis*	Thymocyte selection associated	T cell receptor signaling pathway, immune response	36.9	186.5	**2.69**	<0.0001
*Mogat1*	Monoacylglycerol O-acyltransferase 1	Lipid metabolic process	19.5	66.4	**2.51**	<0.0001
*Kbtbd11*	Kelch repeat and BTB (POZ) domain containing 11		11.9	46.4	**2.36**	<0.0001
*Aatk*	Apoptosis-associated tyrosine kinase	Apoptosis	63.4	201.3	**2.35**	<0.0001
*Tpm2*	Tropomyosin 2, beta	Actin filament stabilization	59.4	216.7	**2.31**	<0.0001
*Cfd*	Complement factor D, adipsin	Complement activation and inflammation	6.8	125.8	**2.23**	<0.0001
*Lgals1*	Lectin, galactose binding, soluble 1	Cell adhesion, regulation of apoptosis	272.4	838.3	**2.20**	<0.0001
*Adgrv1*	Adhesion G protein-coupled receptor V1	Cell adhesion	62.2	157.3	**2.14**	<0.0001
*Lrrc14b*	Leucine rich repeat containing 14B		6.2	21.2	**2.14**	<0.0001
*Tmem28*	Transmembrane protein 28	Calcium ion transport	27.4	81.9	**2.13**	<0.0001
*Slc22a29*	Solute carrier family 22. member 29	Organic anion transport	8.7	39.9	**2.11**	<0.0001
*Clstn3*	Calsyntenin 3	Cell adhesion	285.4	713.2	**2.07**	<0.0001
*Hspb1*	Heat shock protein 1	Negative regulation of apoptosis, positive regulation of interleukin-1 beta production	45.8	109.4	**2.06**	<0.0001
*Tafa2*	TAFA chemokine-like family member 2	Receptor ligand activity	4.3	16.9	**2.04**	<0.0001
*Treh*	Trehalase (brush-border membrane glycoprotein)	Metabolism	22.0	54.0	**1.97**	<0.0001
*Sema5b*	Sema domain, seven thrombospondin repeats (type 1 and type 1-like), transmembrane domain (TM) and short cytoplasmic domain, (semaphorin) 5B	Cell differentiation, positive regulation of cell migration	40.6	102.8	**1.95**	<0.0001
*Osbpl3*	Oxysterol binding protein-like 3	Lipid transport	52.0	181.6	**1.93**	<0.0001
*Fitm1*	Fat storage-inducing transmembrane protein 1	Lipid droplet organization, phospholipid biosynthetic process	409.0	939.7	**1.92**	<0.0001
*Anxa2*	Annexin A2	Regulation of cholesterol metabolism	141.8	311.8	**1.89**	<0.0001
*Hectd2os*	Hectd2, opposite strand		1342.7	3302.2	**1.89**	<0.0001

Information presented in the column “Functions in mouse” is obtained from NCBI Gene [42] and UniProt [43] databases.

**Table 2:** The twenty most strongly downregulated genes in the high-fat (HF) diet relative to the low-fat (LF) control. Mean expression levels are given as DESeq2-normalized counts. *p*-values are adjusted by false discovery rate (FDR).

Gene	Name	Functions in Mouse	Mean (LF)	Mean (HF)	Fold Change	*p*-Value (FDR adj.)
*Lepr*	Leptin receptor	Regulation of metabolism	296.0	43.2	**−3.48**	<0.0001
*Adgrf1*	Adhesion G protein-coupled receptor F1	G-protein coupled receptor activity	122.3	40.0	**−2.06**	<0.0001
*Igfbp2*	Insulin-like growth factor binding protein 2	Glucose metabolism, insulin sensitivity	7680.0	3614.7	**−1.95**	<0.0001
*Fabp5*	Fatty acid binding protein 5, epidermal	Glucose and lipid metabolism	1273.4	156.8	**−1.93**	<0.0001
*Grm8*	Glutamate receptor, metabotropic 8	Glutamate receptor activity	21.7	8.1	**−1.91**	<0.0001
*Adam11*	A disintegrin and metallopeptidase domain 11	Metalloendopeptidase activity	134.1	53.2	**−1.88**	<0.0001
*Srgap3*	Insulin-like growth factor binding protein 2	Negative regulation of cell migration	106.8	43.8	**−1.82**	<0.0001
*Cyp2c40*	Cytochrome P450, family 2, subfamily c, polypeptide 40	Arachidonic acid epoxygenase activity, metal ion binding	21.3	5.8	**−1.73**	0.0003
*Slc35g1*	Solute carrier family 35, member G1	Regulation of cytosolic calcium ion concentration	399.4	215.0	**−1.72**	<0.0001
*Lpar2*	Lysophosphatidic acid receptor 2	Activation of MAPK activity	55.6	29.3	**−1.65**	<0.0001
*Pde6c*	Phosphodiesterase 6C, cGMP specific, cone, alpha prime	3′,5′-cyclic-GMP phosphodiesterase activity, metal ion and nucleotide binding	33.5	16.1	**−1.64**	0.0008
*St3gal5*	ST3 beta-galactoside alpha−2,3-sialyltransferase 5	Protein glycosylation	2464.8	1193.9	**−1.62**	0.0005
*Sds*	Serine hydratase	L-serine ammonia-lyase activity	3961.3	2026.8	**−1.62**	0.0007
*Sox12*	SRY (sex determining region Y)-box 12	Cell differentiation	113.2	64.0	**−1.61**	<0.0001
*Tff3*	Trefoil factor 3, intestinal	Regulation of glucose metabolism	37.1	15.4	**−1.61**	0.0020
*Cadm4*	Cell adhesion molecule 4	Regulation of cell proliferation	138.7	83.8	**−1.59**	<0.0001
*Rnf145*	Ring finger protein 145	Metal ion binding, transferase activity	581.0	326.8	**−1.58**	<0.0001
*Lgals4*	Lectin, galactose binding, soluble 4	Cell adhesion	130.7	72.8	**−1.58**	0.0001
*Cspg5*	Chondroitin sulfate proteoglycan 5	Cell differentiation	21.8	9.1	**−1.56**	0.0065
*Cd9*	CD9 antigen	Cell adhesion	428.2	243.1	**−1.55**	0.0002

Information presented in the column “Functions in mouse” is obtained from NCBI Gene [42] and UniProt [43] databases.

**Table 3:** The twenty genes with the largest negative fold change (FC) in the lingonberry supplemented high-fat diet (HF + LGB) group relative to the high-fat diet (HF) group. Mean expression levels are given as DESeq2-normalized counts. *p*-values are adjusted by false discovery rate (FDR).

Gene	Name	Functions in Mouse	Mean (HF)	Mean (HF + LGB)	Fold Change	*p*-Value (FDR adj.)
*Wfdc2*	WAP four-disulfide core domain 2	Endopeptidase inhibitor activity	185.0	54.2	**−2.28**	<0.0001
*Apoa4*	Apolipoprotein A-IV	Antioxidant activity, cholesterol and lipid homeostasis	11,871.9	3002.7	**−2.13**	<0.0001
*Gpc1*	Glypican 1	Cell migration	542.7	211.2	**−2.04**	<0.0001
*Slc35f2*	Solute carrier family 35, member F2	Transmembrane transporter activity	34.4	10.5	**−2.04**	<0.0001
*Ifi27l2b*	Interferon, alpha-inducible protein 27 like 2B	Immune system process, intrinsic apoptotic signaling pathway	102.6	31.2	**−2.04**	<0.0001
*Rad51b*	RAD51 paralog B	DNA recombination and repair, positive regulation of cell proliferation	90.9	23.3	**−2.03**	<0.0001
*Lcn2*	Lipocalin 2	Apoptotic process, inflammation	174.8	42.2	**−1.99**	<0.0001
*Morc4*	Microrchidia 4	Metal ion and zinc ion binding	72.5	30.8	**−1.95**	<0.0001
*Rarres1*	Retinoic acid receptor responder (tazarotene induced) 1	Metalloendopeptidase inhibitor activity	1168.6	425.4	**−1.95**	<0.0001
*Fam129b*	Family with sequence similarity 129, member B	Negative regulation of DNA biosynthetic process and cell proliferation	304.6	130.0	**−1.87**	<0.0001
*Bmyc*	Brain expressed myelocytomatosis oncogene	Regulation of DNA transcription	89.6	38.7	**−1.83**	<0.0001
*Smpd3*	Sphingomyelin phosphodiesterase 3, neutral	Extracellular matrix assembly, regulation of cell proliferation	106.8	36.2	**−1.83**	<0.0001
*Saa2*	Serum amyloid A 2	Acute-phase response, inflammation	762.9	222.7	**−1.83**	<0.0001
*Aqp8*	Aquaporin 8	Canalicular bile acid transport, water transport	5539.7	2377.6	**−1.82**	<0.0001
*Cyp46a1*	Cytochrome P450, family 46, subfamily a, polypeptide 1	Cholesterol catabolic process, iron ion binding	92.2	32.3	**−1.82**	<0.0001
*Ly6d*	Lymphocyte antigen 6 complex, locus D	Response to stilbenoid	45.0	11.1	**−1.80**	<0.0001
*Phlda3*	Pleckstrin homology-like domain, family A, member 3	Phosphatidylinositol-phosphates binding; apoptotic process positive regulation	35.0	13.8	**−1.78**	<0.0001
*Tsc22d1*	TSC22 domain family, member 1	Regulation of apoptosis, cell proliferation	1940.3	947.4	**−1.77**	<0.0001
*Extl1*	Exostoses (multiple)-like 1	Glycosaminoglycan biosynthesis	324.5	153.5	**−1.77**	<0.0001
*Saa1*	Serum amyloid A 1	Acute-phase response, inflammation, cholesterol metabolic process	1277.5	471.0	**−1.75**	<0.0001

Information presented in the column ”Functions in mouse” is obtained from NCBI Gene [42] and UniProt [43] databases.

**Table 4:** The twenty genes with the largest positive fold change (FC) in the lingonberry supplemented high-fat diet (HF + LGB) group relative to the high-fat diet (HF) group. Mean expression levels are given as DESeq2-normalized counts. *p*-values are adjusted by false discovery rate (FDR).

Gene	Name	Functions in Mouse	Mean (HF)	Mean (HF + LGB)	Fold Change	*p*-Value (FDR adj.)
*Cyp3a11*	Cytochrome P450, family 3, subfamily a, polypeptide 11	Oxidation and reduction, steroid metabolism	6628.2	27,365.0	**2.85**	<0.0001
*Cyp2c55*	Cytochrome P450, family 2, subfamily c, polypeptide 55	Fatty acid metabolism	27.3	84.5	**2.22**	<0.0001
*Adgrf1*	Adhesion G protein-coupled receptor F1	G protein receptor activity	36.2	143.8	**1.91**	<0.0001
*Emp2*	Epithelial membrane protein 2	Cell adhesion, regulation of angiogenesis	125.1	253.5	**1.79**	<0.0001
*Cyp2c29*	Cytochrome P450, family 2, subfamily c, polypeptide 29	Fatty acid metabolism	8826.3	16,460.9	**1.75**	<0.0001
*Grid1*	Glutamate receptor, ionotropic, delta 1	Glutamate receptor activity, ion transport	17.4	42.0	**1.75**	<0.0001
*Hsd17b6*	Hydroxysteroid (17-beta) dehydrogenase 6	Estradiol dehydrogenase activity, lipid and steroid metabolic process	1545.8	3033.3	**1.74**	<0.0001
*Ces2a*	Carboxylesterase 2A	Carboxylic ester hydrolase activity, protein glycosylation	1987.2	3581.2	**1.73**	<0.0001
*Fam222a*	Family with sequence similarity 222, member A		16.2	38.3	**1.72**	0.0001
*Igfbp2*	Insulin-like growth factor binding protein 2	Glucose metabolism, insulin sensitivity	3388.8	6263.7	**1.71**	<0.0001
*Asap3*	ArfGAP with SH3 domain, ankyrin repeat and PH domain 3	Cell migration	77.5	140.1	**1.69**	<0.0001
*Neb*	Nebulin	Actin filament and protein binding	161.9	337.1	**1.68**	<0.0001
*Slc7a2*	Solute carrier family 7 (cationic amino acid transporter, y+ system), member 2	Amino acid import across plasma membrane, regulation of inflammation	7225.2	13,116.6	**1.65**	<0.0001
*Scnn1a*	Sodium channel, nonvoltage-gated 1 alpha	Sodium ion homeostasis	302.0	511.2	**1.59**	<0.0001
*Sorbs3*	Sorbin and SH3 domain containing 3	Actin filament organization, cell adhesion	333.5	564.2	**1.59**	<0.0001
*Slco1a4*	Solute carrier organic anion transporter family, member 1a4	Bile acid and bile salt transport	489.7	951.0	**1.58**	0.0008
*Gsta2*	Glutathione S-transferase, alpha 2 (Yc2)	Glutathione metabolic process, response to bacterium and stilbenoid, xenobiotic metabolic process	176.3	444.3	**1.58**	0.0026
*Csad*	Cysteine sulfinic acid decarboxylase	Amino acid metabolism	1955.8	4247.9	**1.57**	0.0029
*Enho*	Energy homeostasis associated	Negative regulation of lipid biosynthetic process	84.9	234.9	**1.57**	0.0030
*Gsta4*	Glutathione S-transferase, alpha 4	Drug binding, glutathione metabolic process	619.4	1065.4	**1.56**	<0.0001

Information presented in the column “Functions in mouse” is obtained from NCBI Gene [42] and UniProt [43] databases.

**Table 5:** The 21 genes upregulated by the high-fat (HF) diet (FC > 1.5), and whose expression was significantly lower in the lingonberry-supplemented high-fat diet group (HF + LGB) (FC < −1.5), and the 2 genes (last two rows) downregulated by the high-fat (HF) diet (FC < −1.5), and whose expression was maintained at higher expression level in the lingonberry-supplemented high-fat diet group (HF + LGB) (FC > 1.5). Mean expression levels are given as DESeq2-normalized counts. *p*-values are adjusted by false discovery rate (FDR). * Mean of normalizations performed in comparisons HF vs. LF and HF + LGB vs. HF. LF = low-fat diet.

Gene	Name	Functions in Mouse	Mean (LF)	Mean (HF) *	Mean (HF + LGB) *	FC (HF vs. LF)	*p*-Value (HF vs. LF)	FC (HF + LGB vs. HF)	*p*-Value (HF + LGB vs. HF)
*Mogat1*	Monoacylglycerol O-acyltransferase 1	Lipid metabolic process	19.5	68.4	27.1	2.51	<0.0001	−1.69	0.0003
*Tmem2*	Transmembrane protein 28	Calcium ion transport	27.4	79.5	28.5	2.13	<0.0001	−1.73	0.0001
*Ifi27l2b*	Interferon, alpha-inducible protein 27-like 2B	Regulation of growth	44.1	97.9	31.2	1.83	<0.0001	−2.04	<0.0001
*Gpc1*	Glypican 1	Cell migration	293.2	535.1	211.2	1.78	<0.0001	−2.04	<0.0001
*Cdkn1a*	Cyclin-dependent kinase inhibitor 1A (P21)	Regulation of cell cycle	46.7	108.6	49.8	1.73	0.0002	−1.69	<0.0001
*Tubb6*	Tubulin, beta 6 class V	Cell cycle	42.2	85.3	44.7	1.68	0.0002	−1.54	0.0033
*Pdlim2*	PDZ and LIM domain 2	Actin cytoskeleton organization	17.8	37.3	20.0	1.68	0.0005	−1.56	0.0017
*Wfdc2*	WAP four-disulfide core domain 2	Endopeptidase inhibitor activity	92.0	179.5	54.2	1.66	0.0005	−2.28	<0.0001
*Lcn2*	Lipocalin 2	Apoptotic process, inflammation	54.7	165.9	42.2	1.66	0.0012	−1.99	<0.0001
*Saa1*	Serum amyloid A 1	Acute-phase response, inflammation, cholesterol metabolic process	513.5	1245.2	471.0	1.65	0.0014	−1.75	<0.0001
*Plin4*	Perilipin 4	Lipid droplet-associated protein	74.5	201.9	105.4	1.65	0.0016	−1.55	0.0030
*Cxcl14*	Chemokine (C-X-C motif) ligand 14	Immune response, inflammation	13.2	25.9	8.4	1.60	0.0016	−1.74	0.0001
*Saa2*	Serum amyloid A 2	Acute-phase response, inflammation	285.4	738.1	222.7	1.60	0.0030	−1.83	<0.0001
*Tceal8*	Transcription elongation factor A (SII)-like 8	WW domain binding	322.3	546.1	275.7	1.59	<0.0001	−1.74	<0.0001
*Tubb2a*	Tubulin, beta 2A class IIA	Cell cycle	293.9	1038.1	274.6	1.59	0.0026	−1.52	0.0038
*Orm3*	Orosomucoid 3		9.7	22.3	8.5	1.58	0.0046	−1.52	0.0078
*Phlda3*	Pleckstrin homology-like domain, family A, member 3	Phosphatidylinositol-phosphates binding; apoptotic process positive regulation	17.9	34.6	13.8	1.57	0.0037	−1.78	<0.0001
*Slc25a35*	Solute carrier family 25, member 35	Mitochondrial inner membrane	11.6	21.6	11.2	1.56	0.0024	−1.53	0.0030
*S100a10*	S100 calcium binding protein A10 (calpactin)	Regulation of cell migration, inflammation	1264.2	2097.6	1116.5	1.55	<0.0001	−1.66	<0.0001
*Rad51b*	RAD51 paralog B	DNA recombination and repair, positive regulation of cell proliferation	31.9	87.1	23.3	1.54	0.0067	−2.03	<0.0001
*Gale*	Galactose-4-epimerase, UDP	UDP-N-acetylglucosamine 4-epimerase activity, identical protein binding	232.0	444.4	215.9	1.53	0.0067	−1.57	0.0020
*Adgrf1*	Adhesion G protein-coupled receptor F1	G-protein coupled receptor activity	122.3	38.1	84.5	−2.06	<0.0001	2.22	<0.0001
*Igfbp2*	Insulin-like growth factor binding protein 2	Glucose metabolism, insulin sensitivity	7680.0	3501.8	6263.7	−1.95	<0.0002	1.71	<0.0001

Information presented in the column “Functions in mouse” is obtained from NCBI Gene [42] and UniProt [43] databases.

**Table 6:** Gene Ontology (GO) terms significantly enriched among the significantly differentially expressed genes. Gene lists are obtained from the DAVID tool and reduced with REVIGO.

GO Term	Description	*p*-Value (FDR adj.)
**HF vs. LF**		
GO:0006629	Lipid metabolic process	0.0005
GO:0035634	Response to stilbenoid	0.0012
GO:0050727	Regulation of inflammatory response	0.0222
GO:0071404	Cellular response to low-density lipoprotein particle stimulus	0.0324
GO:0044255	Cellular lipid metabolic process	0.0367
**HF vs. HF + LGB**		
GO:0006629	Lipid metabolic process	4.17 × 10^−5^
GO:0072330	Monocarboxylic acid biosynthetic process	0.0030
GO:0035634	Response to stilbenoid	0.0042
GO:0005975	Carbohydrate metabolic process	0.0042
GO:0033559	Unsaturated fatty acid metabolic process	0.0073
GO:0017144	Drug metabolic process	0.0086
GO:0042866	Pyruvate biosynthetic process	0.0128
GO:0055114	Oxidation-reduction process	0.0157
GO:0006690	Eicosanoid metabolic process	0.0168
GO:0046890	Regulation of lipid biosynthetic process	0.0190
GO:0044262	Cellular carbohydrate metabolic process	0.0202
GO:0051156	Glucose 6-phosphate metabolic process	0.0241
GO:0032429	Regulation of phospholipase A2 activity	0.0255
GO:1901135	Carbohydrate derivative metabolic process	0.0246
GO:0008202	Steroid metabolic process	0.0288
GO:0019216	Regulation of lipid metabolic process	0.0294
GO:0006637	Acyl-CoA metabolic process	0.0355
GO:0006953	Acute-phase response	0.0419

HF = high-fat diet; LF = low-fat diet; HF + LGB = lingonberry supplemented high-fat diet. FDR *p*-value = False discovery rate–corrected *p*-value.

## Data Availability

All relevant data are within the paper.

## Supplementary Materials

The following are available online at https://www.mdpi.com/article/10.3390/nu13113693/s1, Table S1: Composition of the experimental diets; Table S2: All significantly differentially expressed genes in the high-fat diet group compared with the low-fat diet group; Table S3: Functions of the mouse cytochrome enzymes significantly affected by lingonberry supplementation in the high-fat diet validated with PCR; Table S4: All significantly differentially expressed genes in the lingonberry supplemented high-fat diet group compared with the high-fat diet group; Table S5: The genes upregulated by the high-fat diet and whose expression was significantly lower in the lingonberry-supplemented high-fat diet group; Table S6: The genes downregulated by the high-fat diet, and whose expression was significantly higher in the lingonberry-supplemented high-fat diet group; Table S7: Genes associated with inflammation and metabolism validated with PCR; Table S8: All significantly differentially expressed genes belonging to the significantly enriched GO terms in the high-fat diet group compared with the lingonberry supplemented high-fat diet group.
