# Supplementary material for: Effects of Lingonberry (Vaccinium vitis-idaea L.) Supplementation on Hepatic Gene Expression in High-Fat Diet Fed Mice
Source: Nutrients. 2021 Oct 21;13(11):3693. doi: 10.3390/nu13113693 (PMC8623941; doi:10.3390/nu13113693)
Supplement: Supplementary file 1 [file nutrients-13-03693-s001.zip › Table S1.pdf]

Table S1. Composition of the experimental diets.

	LF	HF	HF+LGB
Calculated energy (kcal)			
Protein	716	716	716
Carbohydrate	2840	1422	1422
Starch	2110	691	691
Sugar	730	731	731
Fat	405	1823	1823
Total energy	3961	3961	3961
Calculated energy per gram diet (kcal/g)	3.60	4.39	4.30
Calculated Energy (kcal%)			
Protein	18	18	18
Carbohydrate	72	36	36
Fat	10	46	46
Fiber (g%)	9	10	10
Lingonberry powder (g)	0	0	184*
Ingredients (g)			
			(+ from LGB powder)
Casein	200	200	194 *(+ 6)*<br>total: 200
L-Cystine	3	3	3
Corn Starch	452	73	31 *(+ 42)*<br>total: 73
Maltodextrin 10	75	100	100
Sucrose	173	173	103 *(+ 70)*<br>total: 173
Cellulose	94	94	50 *(+ 44)*<br>total: 94
Soybean Oil	25	25	24 *(+ 1)*<br>total: 25
Lard	20	178	178
Mineral Mix S10026	10	10	10
DiCalcium Phosphate	13	13	13
Calcium Carbonate	6	6	6
Potassium Citrate	17	17	17
Vitamin Mix V10001	10	10	10
Choline Bitartrate	2	2	2

The diets were manufactured by Research Diets, Inc, New Brunswick, NJ, USA. LF=low-fat diet, HF=high-fat diet, HF+LGB=lingonberry-supplemented high-fat diet. *Nutrient content/100 g lingonberry powder: fat 0.8 g, carbohydrates 61 g (of which sugars 38 g), fiber 24 g, protein 3 g.
