# Supplementary material for: Effects of Lingonberry (Vaccinium vitis-idaea L.) Supplementation on Hepatic Gene Expression in High-Fat Diet Fed Mice
Source: Nutrients. 2021 Oct 21;13(11):3693. doi: 10.3390/nu13113693 (PMC8623941; doi:10.3390/nu13113693)
Supplement: Supplementary file 1 [file nutrients-13-03693-s001.zip › Table S2.pdf]

Table S2. All significantly differentially expressed genes in the high-fat (HF) diet group compared to the low-fat (LF) diet group. Mean expression levels are given as DESeq2-normalized counts. p-values are adjusted by false discovery rate (FDR).

**Gene**	**Name**	**Mean (LF)**	**Mean (HF)**	**FC**	**p-value (FDR adj.)**
Themis	thymocyte selection associated [Source:MGI Symbol;Acc:MGI:2443552]	36.9	186.5	**2.69**	1.51E-13
Mogat1	monoacylglycerol O-acyltransferase 1 [Source:MGI Symbol;Acc:MGI:1915643]	19.5	66.4	**2.51**	1.51E-13
Kbtbd11	kelch repeat and BTB (POZ) domain containing 11 [Source:MGI Symbol;Acc:MGI:1922151]	11.9	46.4	**2.36**	6.13E-10
Aatk	apoptosis-associated tyrosine kinase [Source:MGI Symbol;Acc:MGI:1197518]	63.4	201.3	**2.35**	6.68E-13
Tpm2	tropomyosin 2. beta [Source:MGI Symbol;Acc:MGI:98810]	59.4	216.7	**2.31**	2.75E-10
Cfd	complement factor D (adipsin) [Source:MGI Symbol;Acc:MGI:87931]	6.8	125.8	**2.23**	1.57E-09
Lgals1	lectin. galactose binding. soluble 1 [Source:MGI Symbol;Acc:MGI:96777]	272.4	838.3	**2.20**	2.27E-09
Adgrv1	adhesion G protein-coupled receptor V1 [Source:MGI Symbol;Acc:MGI:1274784]	62.2	157.3	**2.14**	1.51E-13
Lrrc14b	leucine rich repeat containing 14B [Source:MGI Symbol;Acc:MGI:2145269]	6.2	21.2	**2.14**	5.62E-08
Tmem28	transmembrane protein 28 [Source:MGI Symbol;Acc:MGI:3648377]	27.4	81.9	**2.13**	4.75E-09
Slc22a29	solute carrier family 22. member 29 [Source:MGI Symbol;Acc:MGI:3605624]	8.7	39.9	**2.11**	2.17E-07
Clstn3	calsyntenin 3 [Source:MGI Symbol;Acc:MGI:2178323]	285.4	713.2	**2.07**	5.83E-10
Hspb1	heat shock protein 1 [Source:MGI Symbol;Acc:MGI:96240]	45.8	109.4	**2.06**	1.50E-09
Tafa2	TAFA chemokine like family member 2 [Source:MGI Symbol;Acc:MGI:2143691]	4.3	16.9	**2.04**	1.04E-06
Treh	trehalase (brush-border membrane glycoprotein) [Source:MGI Symbol;Acc:MGI:1926230]	22.0	54.0	**1.97**	2.80E-07
Sema5b	sema domain. seven thrombospondin repeats (type 1 and type 1-like). transmembrane domain (TM) and short cytoplasmic domain. (semaphorin) 5B [Source:MGI Symbol;Acc:MGI:107555]	40.6	102.8	**1.95**	3.12E-07
Osbpl3	oxysterol binding protein-like 3 [Source:MGI Symbol;Acc:MGI:1918970]	52.0	181.6	**1.93**	6.89E-06
Fitm1	fat storage-inducing transmembrane protein 1 [Source:MGI Symbol;Acc:MGI:1915930]	408.1	896.5	**1.92**	5.91E-08
Anxa2	annexin A2 [Source:MGI Symbol;Acc:MGI:88246]	141.8	311.8	**1.89**	1.45E-07
Hectd2os	Hectd2. opposite strand [Source:MGI Symbol;Acc:MGI:1919243]	1342.7	3302.2	**1.89**	4.14E-07
Slc10a2	solute carrier family 10. member 2 [Source:MGI Symbol;Acc:MGI:1201406]	146.4	293.4	**1.88**	4.59E-07
Slc22a27	solute carrier family 22. member 27 [Source:MGI Symbol;Acc:MGI:3042283]	14.4	67.0	**1.88**	1.59E-05
Tlcd2	TLC domain containing 2 [Source:MGI Symbol;Acc:MGI:1917141]	1061.1	1934.2	**1.85**	3.45E-23
Lrrc39	leucine rich repeat containing 39 [Source:MGI Symbol;Acc:MGI:1924557]	19.7	50.9	**1.84**	3.28E-05
Acyp2	acylphosphatase 2, muscle type [Source:MGI Symbol;Acc:MGI:1922822]	16.5	37.5	**1.83**	9.30E-06
Ifi27l2b	interferon, alpha-inducible protein 27 like 2B [Source:MGI Symbol;Acc:MGI:1916390]	44.1	99.5	**1.83**	1.01E-05
Gck	glucokinase [Source:MGI Symbol;Acc:MGI:1270854]	1729.4	3380.2	**1.82**	1.26E-06
Olig1	oligodendrocyte transcription factor 1 [Source:MGI Symbol;Acc:MGI:1355334]	168.5	315.4	**1.79**	1.26E-10

Gpc1	glypican 1 [Source:MGI Symbol;Acc:MGI:1194891]	293.2	531.4	**1.78**	5.90E-10
Gngt1	guanine nucleotide binding protein (G protein), gamma transducing activity polypeptide 1 [Source:MGI Symbol;Acc:MGI:109165]	15.5	32.7	**1.75**	0.0001589
Mup-ps14	major urinary protein, pseudogene 14 [Source:MGI Symbol;Acc:MGI:3651980]	11.0	23.6	**1.74**	6.73E-05
Rcan2	regulator of calcineurin 2 [Source:MGI Symbol;Acc:MGI:1858219]	51.7	95.6	**1.73**	1.13E-06
Mtnr1a	melatonin receptor 1A [Source:MGI Symbol;Acc:MGI:102967]	30.4	55.3	**1.73**	6.89E-06
Cd36	CD36 molecule [Source:MGI Symbol;Acc:MGI:107899]	1057.2	2079.6	**1.73**	3.64E-05
Cdkn1a	cyclin-dependent kinase inhibitor 1A (P21) [Source:MGI Symbol;Acc:MGI:104556]	46.7	109.0	**1.73**	0.0001823
Lrp11	low density lipoprotein receptor-related protein 11 [Source:MGI Symbol;Acc:MGI:2442989]	25.3	47.5	**1.72**	1.23E-07
Pparg	peroxisome proliferator activated receptor gamma [Source:MGI Symbol;Acc:MGI:97747]	197.6	367.0	**1.72**	4.71E-06
Cidec	cell death-inducing DFFA-like effector c [Source:MGI Symbol;Acc:MGI:95585]	47.6	226.8	**1.72**	0.0002158
Aox1	aldehyde oxidase 1 [Source:MGI Symbol;Acc:MGI:88035]	736.5	1312.2	**1.69**	2.13E-14
Tubb6	tubulin, beta 6 class V [Source:MGI Symbol;Acc:MGI:1915201]	42.2	85.6	**1.68**	0.0001922
Pdlim2	PDZ and LIM domain 2 [Source:MGI Symbol;Acc:MGI:2384850]	17.8	37.3	**1.68**	0.0004577
Gsta1	glutathione S-transferase, alpha 1 (Ya) [Source:MGI Symbol;Acc:MGI:1095417]	2.9	17.7	**1.67**	0.0002761
Dio1	deiodinase, iodothyronine, type I [Source:MGI Symbol;Acc:MGI:94896]	1771.8	3158.8	**1.66**	1.91E-10
Abcc3	ATP-binding cassette, sub-family C (CFTR/MRP), member 3 [Source:MGI Symbol;Acc:MGI:1923658]	2405.8	4436.2	**1.66**	2.23E-05
Pmm1	phosphomannomutase 1 [Source:MGI Symbol;Acc:MGI:1353418]	37.0	71.9	**1.66**	5.99E-05
Limk1	LIM-domain containing, protein kinase [Source:MGI Symbol;Acc:MGI:104572]	30.8	58.3	**1.66**	0.0002513
Gprc5b	G protein-coupled receptor, family C, group 5, member B [Source:MGI Symbol;Acc:MGI:1927596]	6.8	36.2	**1.66**	0.0002898
Wfdc2	WAP four-disulfide core domain 2 [Source:MGI Symbol;Acc:MGI:1914951]	92.0	183.2	**1.66**	0.0004672
Lcn2	lipocalin 2 [Source:MGI Symbol;Acc:MGI:96757]	57.5	174.4	**1.66**	0.00119
Haus8	4HAUS augmin-like complex, subunit 8 [Source:MGI Symbol;Acc:MGI:1923728]	58.6	111.2	**1.65**	0.0002257
Saa1	serum amyloid A 1 [Source:MGI Symbol;Acc:MGI:98221]	513.5	1259.6	**1.65**	0.001443
Plin4	perilipin 4 [Source:MGI Symbol;Acc:MGI:1929709]	74.5	218.7	**1.65**	0.001571
Chchd6	coiled-coil-helix-coiled-coil-helix domain containing 6 [Source:MGI Symbol;Acc:MGI:1913348]	41.5	72.5	**1.64**	8.05E-05
Mup-ps12	major urinary protein, pseudogene 12 [Source:MGI Symbol;Acc:MGI:3783148]	23.1	47.1	**1.64**	0.0005718
Synj2	synaptojanin 2 [Source:MGI Symbol;Acc:MGI:1201671]	94.8	161.4	**1.62**	3.23E-05
Gsdme	gasdermin E [Source:MGI Symbol;Acc:MGI:1889850]	14.2	25.7	**1.61**	0.0002598
Krt23	keratin 23 [Source:MGI Symbol;Acc:MGI:2148866]	42.7	117.6	**1.61**	0.002574
Cxcl14	chemokine (C-X-C motif) ligand 14 [Source:MGI Symbol;Acc:MGI:1888514]	13.2	25.0	**1.60**	0.001611
Saa2	serum amyloid A 2 [Source:MGI Symbol;Acc:MGI:98222]	285.4	744.1	**1.60**	0.002955
Tceal8	transcription elongation factor A (SII)-like 8 [Source:MGI Symbol;Acc:MGI:1913934]	322.3	529.0	**1.59**	1.41E-05

Nat1	N-acetyl transferase 1 [Source:MGI Symbol;Acc:MGI:97279]	19.7	36.9	**1.59**	0.0003832
Tubb2a	tubulin, beta 2A class IIA [Source:MGI Symbol;Acc:MGI:107861]	293.9	960.3	**1.59**	0.002633
Bc1	brain cytoplasmic RNA 1 [Source:MGI Symbol;Acc:MGI:104905]	9.5	19.6	**1.59**	0.002955
Fcor	Foxo1 corepressor [Source:MGI Symbol;Acc:MGI:1915484]	6.2	13.7	**1.59**	0.003842
Cep78	centrosomal protein 78 [Source:MGI Symbol;Acc:MGI:1924386]	24.4	42.3	**1.58**	7.28E-05
Orm3	orosomucoid 3 [Source:MGI Symbol;Acc:MGI:97445]	9.7	22.9	**1.58**	0.004587
Adora1	adenosine A1 receptor [Source:MGI Symbol;Acc:MGI:99401]	598.8	961.6	**1.57**	5.95E-06
Phlda3	pleckstrin homology like domain, family A, member 3 [Source:MGI Symbol;Acc:MGI:1351485]	17.9	34.3	**1.57**	0.003689
Mrgprb11-ps	MAS-related GPR, member B11, pseudogene [Source:MGI Symbol;Acc:MGI:3033189]	8.6	18.4	**1.57**	0.004195
Psd4	pleckstrin and Sec7 domain containing 4 [Source:MGI Symbol;Acc:MGI:2674093]	60.6	97.9	**1.56**	2.87E-06
Mapk15	mitogen-activated protein kinase 15 [Source:MGI Symbol;Acc:MGI:2652894]	95.0	156.1	**1.56**	5.81E-05
Tubb4b	tubulin, beta 4B class IVB [Source:MGI Symbol;Acc:MGI:1915472]	696.0	1205.4	**1.56**	0.0007594
Slc25a35	solute carrier family 25, member 35 [Source:MGI Symbol;Acc:MGI:1919248]	11.6	21.9	**1.56**	0.002354
Atp8b5	ATPase, class I, type 8B, member 5 [Source:MGI Symbol;Acc:MGI:2444287]	7.4	16.7	**1.56**	0.0055
Hmgcs1	3-hydroxy-3-methylglutaryl-Coenzyme A synthase 1 [Source:MGI Symbol;Acc:MGI:107592]	3754.2	7807.6	**1.56**	0.005753
S100a10	S100 calcium binding protein A10 (calpactin) [Source:MGI Symbol;Acc:MGI:1339468]	1264.2	2037.6	**1.55**	3.28E-05
Cd59b	CD59b antigen [Source:MGI Symbol;Acc:MGI:1888996]	37.9	64.0	**1.55**	0.0001868
Zc4h2	zinc finger, C4H2 domain containing [Source:MGI Symbol;Acc:MGI:2679294]	11.5	18.7	**1.55**	0.002618
Ggct	gamma-glutamyl cyclotransferase [Source:MGI Symbol;Acc:MGI:95700]	109.0	163.2	**1.54**	1.48E-05
Map9	microtubule-associated protein 9 [Source:MGI Symbol;Acc:MGI:2442208]	11.7	22.1	**1.54**	0.005236
Ocstamp	osteoclast stimulatory transmembrane protein [Source:MGI Symbol;Acc:MGI:1921864]	10.1	17.6	**1.54**	0.005268
Rad51b	RAD51 paralog B [Source:MGI Symbol;Acc:MGI:1099436]	31.9	87.3	**1.54**	0.006707
Vmn2r57	vomeronasal 2, receptor 57 [Source:MGI Symbol;Acc:MGI:3703084]	6.9	13.5	**1.54**	0.007524
Zfp423	zinc finger protein 423 [Source:MGI Symbol;Acc:MGI:1891217]	5.5	13.5	**1.54**	0.008265
Hsd17b10	hydroxysteroid (17-beta) dehydrogenase 10 [Source:MGI Symbol;Acc:MGI:1333871]	2551.4	3960.3	**1.53**	3.74E-07
Zc3h12d	zinc finger CCCH type containing 12D [Source:MGI Symbol;Acc:MGI:3045313]	66.6	101.2	**1.53**	0.0001576
Mup-ps7	major urinary protein, pseudogene 7 [Source:MGI Symbol;Acc:MGI:3651245]	11.4	22.2	**1.53**	0.005622
Gale	galactose-4-epimerase, UDP [Source:MGI Symbol;Acc:MGI:1921496]	232.0	416.7	**1.53**	0.00666
Tmem237	transmembrane protein 237 [Source:MGI Symbol;Acc:MGI:2138365]	41.1	63.9	**1.52**	0.0002516
Nqo1	NAD(P)H dehydrogenase, quinone 1 [Source:MGI Symbol;Acc:MGI:103187]	75.5	121.3	**1.52**	0.0002949
Serpinh1	serine (or cysteine) peptidase inhibitor, clade H, member 1 [Source:MGI Symbol;Acc:MGI:88283]	100.8	155.8	**1.52**	0.0006865
Stap1	signal transducing adaptor family member 1 [Source:MGI Symbol;Acc:MGI:1926193]	71.4	123.0	**1.52**	0.002458

Slc26a4	solute carrier family 26, member 4 [Source:MGI Symbol;Acc:MGI:1346029]	11.3	21.2	**1.52**	0.009592
Srxn1	sulfiredoxin 1 homolog (S. cerevisiae) [Source:MGI Symbol;Acc:MGI:104971]	676.3	1018.9	**1.51**	3.74E-07
Adamts2	a disintegrin-like and metallopeptidase (reprolysin type) with thrombospondin type 1 motif, 2 [Source:MGI Symbol;Acc:MGI:1347356]	48.0	72.8	**1.51**	0.001487
Sdf2l1	stromal cell-derived factor 2-like 1 [Source:MGI Symbol;Acc:MGI:2149842]	235.9	359.7	**1.51**	0.002925
Ctse	cathepsin E [Source:MGI Symbol;Acc:MGI:107361]	6.8	13.6	**1.51**	0.01269
Cnnm1	cyclin M1 [Source:MGI Symbol;Acc:MGI:1891366]	7.3	15.0	**1.51**	0.01284
Cyp2a22	cytochrome P450, family 2, subfamily a, polypeptide 22 [Source:MGI Symbol;Acc:MGI:3648316]	44.9	106.8	**1.51**	0.01336
Tbc1d2	TBC1 domain family, member 2 [Source:MGI Symbol;Acc:MGI:2652885]	10.3	21.0	**1.51**	0.01356
Ighm	immunoglobulin heavy constant mu [Source:MGI Symbol;Acc:MGI:96448]	113.6	224.3	**1.51**	0.01408
Spata7	spermatogenesis associated 7 [Source:MGI Symbol;Acc:MGI:2144877]	20.2	31.7	**1.49**	0.0008394
Camk2n2	calcium/calmodulin-dependent protein kinase II inhibitor 2 [Source:MGI Symbol;Acc:MGI:1920297]	26.3	42.0	**1.49**	0.002428
Nt5e	5' nucleotidase, ecto [Source:MGI Symbol;Acc:MGI:99782]	75.5	115.7	**1.49**	0.003128
Ildr2	immunoglobulin-like domain containing receptor 2 [Source:MGI Symbol;Acc:MGI:1196370]	376.5	664.8	**1.49**	0.007319
Ugdh	UDP-glucose dehydrogenase [Source:MGI Symbol;Acc:MGI:1306785]	1823.9	2725.1	**1.48**	1.05E-05
Sytl4	synaptotagmin-like 4 [Source:MGI Symbol;Acc:MGI:1351606]	40.3	63.9	**1.48**	0.0002343
Asic5	acid-sensing (proton-gated) ion channel family member 5 [Source:MGI Symbol;Acc:MGI:1929259]	56.5	92.8	**1.48**	0.000662
Pnldc1	poly(A)-specific ribonuclease (PARN)-like domain containing 1 [Source:MGI Symbol;Acc:MGI:2685159]	144.4	235.1	**1.48**	0.00299
Gpx6	glutathione peroxidase 6 [Source:MGI Symbol;Acc:MGI:1922762]	48.2	71.7	**1.48**	0.003247
Ttc39a	tetratricopeptide repeat domain 39A [Source:MGI Symbol;Acc:MGI:2444350]	8.4	29.1	**1.48**	0.009164
Ubd	ubiquitin D [Source:MGI Symbol;Acc:MGI:1344410]	13.5	41.1	**1.48**	0.01186
Slc39a5	solute carrier family 39 (metal ion transporter), member 5 [Source:MGI Symbol;Acc:MGI:1919336]	21.1	38.8	**1.48**	0.01567
Mterf2	mitochondrial transcription termination factor 2 [Source:MGI Symbol;Acc:MGI:1921488]	45.2	71.1	**1.47**	9.69E-05
Bmp7	bone morphogenetic protein 7 [Source:MGI Symbol;Acc:MGI:103302]	39.1	63.7	**1.47**	0.0002934
Stk39	serine/threonine kinase 39 [Source:MGI Symbol;Acc:MGI:1858416]	25.3	38.4	**1.47**	0.001602
Kcnk10	potassium channel, subfamily K, member 10 [Source:MGI Symbol;Acc:MGI:1919508]	29.4	49.4	**1.47**	0.001627
Tagln	transgelin [Source:MGI Symbol;Acc:MGI:106012]	57.9	84.5	**1.47**	0.003508
Fabp2	fatty acid binding protein 2, intestinal [Source:MGI Symbol;Acc:MGI:95478]	1717.6	2689.5	**1.47**	0.003871
Krt10	keratin 10 [Source:MGI Symbol;Acc:MGI:96685]	26.6	43.0	**1.47**	0.01084
Ddias	DNA damage-induced apoptosis suppressor [Source:MGI Symbol;Acc:MGI:1921291]	15.7	28.7	**1.47**	0.01417
Clba1	clathrin binding box of aftiphilin containing 1 [Source:MGI Symbol;Acc:MGI:2443738]	8.7	16.6	**1.47**	0.0143
Uck1	uridine-cytidine kinase 1 [Source:MGI Symbol;Acc:MGI:98904]	481.9	723.9	**1.46**	7.62E-06
Tfpi2	tissue factor pathway inhibitor 2 [Source:MGI Symbol;Acc:MGI:108543]	867.5	1241.9	**1.46**	1.30E-05

Them6	thioesterase superfamily member 6 [Source:MGI Symbol;Acc:MGI:1925301]	64.6	96.6	**1.46**	6.08E-05
Mmab	methylmalonic aciduria (cobalamin deficiency) cblB type homolog (human) [Source:MGI Symbol;Acc:MGI:1924947]	324.5	478.0	**1.46**	0.0001589
Aldh1a1	aldehyde dehydrogenase family 1, subfamily A1 [Source:MGI Symbol;Acc:MGI:1353450]	18215.2	27506.4	**1.45**	1.49E-06
Lect2	leukocyte cell-derived chemotaxin 2 [Source:MGI Symbol;Acc:MGI:1278342]	1632.0	2512.9	**1.45**	1.28E-05
Castor2	cytosolic arginine sensor for mTORC1 subunit 2 [Source:MGI Symbol;Acc:MGI:1933384]	47.5	71.2	**1.45**	0.0003165
Arhgef9	CDC42 guanine nucleotide exchange factor (GEF) 9 [Source:MGI Symbol;Acc:MGI:2442233]	123.2	181.8	**1.45**	0.0007031
Pard3bos3	par-3 family cell polarity regulator beta, opposite strand 3 [Source:MGI Symbol;Acc:MGI:3651159]	8.2	15.2	**1.45**	0.02074
Gpr12	G-protein coupled receptor 12 [Source:MGI Symbol;Acc:MGI:101909]	9.2	17.5	**1.45**	0.02368
Krt8	keratin 8 [Source:MGI Symbol;Acc:MGI:96705]	1990.8	2845.0	**1.44**	2.20E-05
Serf1	small EDRK-rich factor 1 [Source:MGI Symbol;Acc:MGI:1337114]	46.4	68.5	**1.44**	0.005889
Gsta2	glutathione S-transferase, alpha 2 (Yc2) [Source:MGI Symbol;Acc:MGI:95863]	97.8	173.7	**1.44**	0.02468
Plk4	polo like kinase 4 [Source:MGI Symbol;Acc:MGI:101783]	12.5	21.8	**1.44**	0.02557
Kifap3	kinesin-associated protein 3 [Source:MGI Symbol;Acc:MGI:107566]	73.2	106.6	**1.43**	1.04E-06
Gstk1	glutathione S-transferase kappa 1 [Source:MGI Symbol;Acc:MGI:1923513]	1571.4	2305.9	**1.43**	0.0001567
Mgmt	O-6-methylguanine-DNA methyltransferase [Source:MGI Symbol;Acc:MGI:96977]	320.2	479.6	**1.43**	0.0001808
Car3	carbonic anhydrase 3 [Source:MGI Symbol;Acc:MGI:88270]	53266.4	80706.8	**1.43**	0.002192
Mcm5	minichromosome maintenance complex component 5 [Source:MGI Symbol;Acc:MGI:103197]	29.7	43.7	**1.43**	0.003038
Tuba1c	tubulin, alpha 1C [Source:MGI Symbol;Acc:MGI:1095409]	236.4	389.9	**1.43**	0.0229
Col1a1	collagen, type I, alpha 1 [Source:MGI Symbol;Acc:MGI:88467]	61.2	140.6	**1.43**	0.02814
Smpd3	sphingomyelin phosphodiesterase 3, neutral [Source:MGI Symbol;Acc:MGI:1927578]	60.2	102.1	**1.43**	0.02922
Fbxo40	F-box protein 40 [Source:MGI Symbol;Acc:MGI:2443753]	10.4	19.2	**1.43**	0.03041
Nupr1	nuclear protein transcription regulator 1 [Source:MGI Symbol;Acc:MGI:1891834]	15.9	28.8	**1.43**	0.03245
Rufy4	RUN and FYVE domain containing 4 [Source:MGI Symbol;Acc:MGI:3588214]	11.3	19.6	**1.43**	0.03344
Cpxm1	carboxypeptidase X 1 (M14 family) [Source:MGI Symbol;Acc:MGI:1934569]	13.3	21.6	**1.43**	0.03387
Eda2r	ectodysplasin A2 receptor [Source:MGI Symbol;Acc:MGI:2442860]	12.5	26.3	**1.43**	0.0356
Ssc4d	scavenger receptor cysteine rich family, 4 domains [Source:MGI Symbol;Acc:MGI:1924709]	39.0	58.5	**1.42**	0.002888
Osgin2	oxidative stress induced growth inhibitor family member 2 [Source:MGI Symbol;Acc:MGI:2384798]	18.8	29.7	**1.42**	0.007718
Msmo1	methylsterol monoxygenase 1 [Source:MGI Symbol;Acc:MGI:1913484]	1937.0	3337.8	**1.42**	0.03887
Slc22a15	solute carrier family 22 (organic anion/cation transporter), member 15 [Source:MGI Symbol;Acc:MGI:3607704]	258.0	354.1	**1.41**	6.11E-05
Thnsl1	threonine synthase-like 1 (bacterial) [Source:MGI Symbol;Acc:MGI:2139347]	61.7	90.1	**1.41**	7.71E-05
Tmem98	transmembrane protein 98 [Source:MGI Symbol;Acc:MGI:1923457]	125.5	181.2	**1.41**	0.000662

Chaf1a	chromatin assembly factor 1, subunit A (p150) [Source:MGI Symbol;Acc:MGI:1351331]	28.5	44.1	**1.41**	0.001829
Ctps	cytidine 5'-triphosphate synthase [Source:MGI Symbol;Acc:MGI:1858304]	70.0	105.1	**1.41**	0.003046
Endod1	endonuclease domain containing 1 [Source:MGI Symbol;Acc:MGI:1919196]	39.7	59.5	**1.41**	0.003455
Hnf1aos1	HNF1 homeobox A, opposite strand 1 [Source:MGI Symbol;Acc:MGI:3652225]	111.7	174.9	**1.41**	0.006988
Spc24	SPC24, NDC80 kinetochore complex component, homolog (S. cerevisiae) [Source:MGI Symbol;Acc:MGI:1914879]	216.3	317.7	**1.41**	0.009292
Bhlhb9	basic helix-loop-helix domain containing, class B9 [Source:MGI Symbol;Acc:MGI:1917487]	62.3	93.7	**1.41**	0.01085
Rab23	RAB23, member RAS oncogene family [Source:MGI Symbol;Acc:MGI:99833]	96.6	148.0	**1.41**	0.01191
Ncmap	noncompact myelin associated protein [Source:MGI Symbol;Acc:MGI:2444888]	12.4	20.7	**1.41**	0.02294
Acot6	acyl-CoA thioesterase 6 [Source:MGI Symbol;Acc:MGI:1921287]	21.0	32.6	**1.41**	0.02428
Uap1l1	UDP-N-acteylglucosamine pyrophosphorylase 1-like 1 [Source:MGI Symbol;Acc:MGI:2443318]	87.8	138.2	**1.41**	0.02996
Bcl2l14	BCL2-like 14 (apoptosis facilitator) [Source:MGI Symbol;Acc:MGI:1914063]	6.9	12.3	**1.41**	0.044
Mpp7	membrane protein, palmitoylated 7 (MAGUK p55 subfamily member 7) [Source:MGI Symbol;Acc:MGI:1922989]	63.6	94.0	**1.40**	0.002852
Ces1b	carboxylesterase 1B [Source:MGI Symbol;Acc:MGI:3779470]	553.8	874.0	**1.40**	0.003583
Enc1	ectodermal-neural cortex 1 [Source:MGI Symbol;Acc:MGI:109610]	112.4	160.3	**1.40**	0.005236
Arl6	ADP-ribosylation factor-like 6 [Source:MGI Symbol;Acc:MGI:1927136]	27.6	41.3	**1.40**	0.01149
Tlr2	toll-like receptor 2 [Source:MGI Symbol;Acc:MGI:1346060]	37.9	51.5	**1.40**	0.0129
Proca1	protein interacting with cyclin A1 [Source:MGI Symbol;Acc:MGI:1918274]	45.6	64.8	**1.40**	0.01299
Mvk	mevalonate kinase [Source:MGI Symbol;Acc:MGI:107624]	275.9	417.0	**1.40**	0.01466
Atp8b4	ATPase, class I, type 8B, member 4 [Source:MGI Symbol;Acc:MGI:1859664]	32.7	49.4	**1.40**	0.02076
Adamtsl2	ADAMTS-like 2 [Source:MGI Symbol;Acc:MGI:1925044]	29.2	45.9	**1.40**	0.02915
Napepld	N-acyl phosphatidylethanolamine phospholipase D [Source:MGI Symbol;Acc:MGI:2140885]	12.7	18.5	**1.40**	0.02991
Xrcc2	X-ray repair complementing defective repair in Chinese hamster cells 2 [Source:MGI Symbol;Acc:MGI:1927345]	25.7	38.9	**1.39**	0.00441
Dop1b	DOP1 leucine zipper like protein B [Source:MGI Symbol;Acc:MGI:1917278]	252.7	381.0	**1.39**	0.006256
Tlr12	toll-like receptor 12 [Source:MGI Symbol;Acc:MGI:3045221]	270.8	392.3	**1.39**	0.008299
Raet1d	retinoic acid early transcript delta [Source:MGI Symbol;Acc:MGI:1861032]	155.7	225.5	**1.39**	0.01424
Mms22l	MMS22-like, DNA repair protein [Source:MGI Symbol;Acc:MGI:2684980]	21.2	31.8	**1.39**	0.01442
Pik3r3	phosphoinositide-3-kinase regulatory subunit 3 [Source:MGI Symbol;Acc:MGI:109277]	17.7	27.6	**1.39**	0.02552
Col1a2	collagen, type I, alpha 2 [Source:MGI Symbol;Acc:MGI:88468]	157.0	238.1	**1.39**	0.04876
Agxt2	alanine-glyoxylate aminotransferase 2 [Source:MGI Symbol;Acc:MGI:2146052]	2395.1	3338.2	**1.39**	1.20E-12
Ephx1	epoxide hydrolase 1, microsomal [Source:MGI Symbol;Acc:MGI:95405]	3292.7	4666.0	**1.39**	3.50E-05

Polr3k	polymerase (RNA) III (DNA directed) polypeptide K [Source:MGI Symbol;Acc:MGI:1914255]	264.8	367.5	**1.39**	0.0001847
Ttc30b	tetratricopeptide repeat domain 30B [Source:MGI Symbol;Acc:MGI:1919671]	71.3	98.1	**1.39**	0.0004577
Aldh3a2	aldehyde dehydrogenase family 3, subfamily A2 [Source:MGI Symbol;Acc:MGI:1353452]	7154.8	10535.1	**1.39**	0.003396
Polr2k	polymerase (RNA) II (DNA directed) polypeptide K [Source:MGI Symbol;Acc:MGI:102725]	67.5	92.4	**1.39**	0.004604
Plekha1	pleckstrin homology domain containing, family A (phosphoinositide binding specific) member 1 [Source:MGI Symbol;Acc:MGI:2442213]	173.6	241.6	**1.39**	0.009463
Aox3	aldehyde oxidase 3 [Source:MGI Symbol;Acc:MGI:1918974]	5881.7	9059.7	**1.39**	0.01346
Msl3l2	MSL3 like 2 [Source:MGI Symbol;Acc:MGI:1920640]	17.4	26.1	**1.39**	0.03242
Cd63	CD63 antigen [Source:MGI Symbol;Acc:MGI:99529]	53.4	75.7	**1.39**	0.04214
Gpat3	glycerol-3-phosphate acyltransferase 3 [Source:MGI Symbol;Acc:MGI:3603816]	80.5	127.8	**1.39**	0.04925
Them7	thioesterase superfamily member 7 [Source:MGI Symbol;Acc:MGI:1921338]	255.0	363.8	**1.38**	4.13E-06
Chpt1	choline phosphotransferase 1 [Source:MGI Symbol;Acc:MGI:2384841]	4027.8	5607.6	**1.38**	0.0002792
Impg2	interphotoreceptor matrix proteoglycan 2 [Source:MGI Symbol;Acc:MGI:3044955]	91.1	130.3	**1.38**	0.001433
Dhrs7b	dehydrogenase/reductase (SDR family) member 7B [Source:MGI Symbol;Acc:MGI:2384931]	379.0	525.4	**1.38**	0.001512
Pigh	phosphatidylinositol glycan anchor biosynthesis, class H [Source:MGI Symbol;Acc:MGI:99463]	40.1	57.6	**1.38**	0.005559
Nhlrc1	NHL repeat containing 1 [Source:MGI Symbol;Acc:MGI:2145264]	25.0	35.4	**1.38**	0.01046
Extl1	exostoses (multiple)-like 1 [Source:MGI Symbol;Acc:MGI:1888742]	213.0	322.8	**1.38**	0.01321
Aqp8	aquaporin 8 [Source:MGI Symbol;Acc:MGI:1195271]	3869.8	5347.3	**1.38**	0.04545
Nans	N-acetylneuraminic acid synthase (sialic acid synthase) [Source:MGI Symbol;Acc:MGI:2149820]	211.0	284.8	**1.37**	9.06E-06
Dck	deoxycytidine kinase [Source:MGI Symbol;Acc:MGI:102726]	94.0	129.3	**1.37**	0.001266
Ccdc66	coiled-coil domain containing 66 [Source:MGI Symbol;Acc:MGI:2443639]	40.2	56.7	**1.37**	0.007656
Twf2	twinfilin actin binding protein 2 [Source:MGI Symbol;Acc:MGI:1346078]	35.9	48.7	**1.37**	0.01179
Gpx4-ps2	glutathione peroxidase 4, pseudogene 2 [Source:MGI Symbol;Acc:MGI:3779731]	64.5	90.0	**1.37**	0.02553
Col12a1	collagen, type XII, alpha 1 [Source:MGI Symbol;Acc:MGI:88448]	26.1	37.6	**1.37**	0.03644
Zfp518b	zinc finger protein 518B [Source:MGI Symbol;Acc:MGI:2140750]	19.0	30.3	**1.37**	0.0416
Atg4a	autophagy related 4A, cysteine peptidase [Source:MGI Symbol;Acc:MGI:2147903]	116.0	157.7	**1.36**	1.29E-06
Slc35e3	solute carrier family 35, member E3 [Source:MGI Symbol;Acc:MGI:2448489]	137.9	187.7	**1.36**	0.0001589
Tnip1	TNFAIP3 interacting protein 1 [Source:MGI Symbol;Acc:MGI:1926194]	123.4	167.1	**1.36**	0.0007181
Acot9	acyl-CoA thioesterase 9 [Source:MGI Symbol;Acc:MGI:1928939]	51.3	68.1	**1.36**	0.001733
Sowahc	sosondowah ankyrin repeat domain family member C [Source:MGI Symbol;Acc:MGI:3606051]	95.0	128.8	**1.36**	0.002852
Mpc1-ps	mitochondrial pyruvate carrier 1, pseudogene [Source:MGI Symbol;Acc:MGI:3781628]	238.1	318.9	**1.36**	0.003631
Dcaf4	DDB1 and CUL4 associated factor 4 [Source:MGI Symbol;Acc:MGI:1921078]	45.1	63.2	**1.36**	0.003981
Macrod2	MACRO domain containing 2 [Source:MGI Symbol;Acc:MGI:1920149]	48.0	66.7	**1.36**	0.005883

Hist1h2bc	histone cluster 1, H2bc [Source:MGI Symbol;Acc:MGI:1915274]	1016.4	1409.2	**1.36**	0.01265
Btc	betacellulin, epidermal growth factor family member [Source:MGI Symbol;Acc:MGI:99439]	42.6	60.9	**1.36**	0.02222
Pomk	protein-O-mannose kinase [Source:MGI Symbol;Acc:MGI:1921903]	113.4	152.9	**1.35**	3.14E-05
Snx11	sorting nexin 11 [Source:MGI Symbol;Acc:MGI:1921729]	86.9	120.7	**1.35**	0.0001731
Mkrn2os	makorin, ring finger protein 2, opposite strand [Source:MGI Symbol;Acc:MGI:1917541]	266.7	355.0	**1.35**	0.000991
Bbs4	Bardet-Biedl syndrome 4 (human) [Source:MGI Symbol;Acc:MGI:2143311]	34.0	46.8	**1.35**	0.01284
Cenpm	centromere protein M [Source:MGI Symbol;Acc:MGI:1913820]	26.7	37.6	**1.35**	0.02676
Vcpkmt	valosin containing protein lysine (K) methyltransferase [Source:MGI Symbol;Acc:MGI:2684917]	28.7	40.4	**1.35**	0.02996
Acaa1b	acetyl-Coenzyme A acyltransferase 1B [Source:MGI Symbol;Acc:MGI:3605455]	18547.7	26292.0	**1.35**	0.03057
Samd4	sterile alpha motif domain containing 4 [Source:MGI Symbol;Acc:MGI:1921730]	45.4	64.3	**1.35**	0.03393
Uros	uroporphyrinogen III synthase [Source:MGI Symbol;Acc:MGI:98917]	241.7	342.3	**1.34**	3.49E-05
Cd59a	CD59a antigen [Source:MGI Symbol;Acc:MGI:109177]	951.7	1285.5	**1.34**	0.001677
Katnb1	katanin p80 (WD40-containing) subunit B 1 [Source:MGI Symbol;Acc:MGI:1921437]	37.3	52.1	**1.34**	0.009804
E2f1	E2F transcription factor 1 [Source:MGI Symbol;Acc:MGI:101941]	52.5	72.1	**1.34**	0.01012
Plgrkt	plasminogen receptor, C-terminal lysine transmembrane protein [Source:MGI Symbol;Acc:MGI:1915009]	39.0	56.8	**1.34**	0.01747
Pmepa1	prostate transmembrane protein, androgen induced 1 [Source:MGI Symbol;Acc:MGI:1929600]	31.8	43.5	**1.34**	0.02141
Wdr5b	WD repeat domain 5B [Source:MGI Symbol;Acc:MGI:1916794]	29.8	41.5	**1.34**	0.02256
Rasgrp2	RAS, guanyl releasing protein 2 [Source:MGI Symbol;Acc:MGI:1333849]	159.1	222.5	**1.34**	0.02259
Gpx4	glutathione peroxidase 4 [Source:MGI Symbol;Acc:MGI:104767]	64.5	90.0	**1.34**	0.027
R3hcc1	R3H domain and coiled-coil containing 1 [Source:MGI Symbol;Acc:MGI:1919093]	185.5	247.0	**1.33**	4.60E-06
Fancl	Fanconi anemia, complementation group L [Source:MGI Symbol;Acc:MGI:1914280]	112.6	146.1	**1.33**	0.0003081
Ube2l6	ubiquitin-conjugating enzyme E2L 6 [Source:MGI Symbol;Acc:MGI:1914500]	833.4	1079.8	**1.33**	0.0006212
Ces1g	carboxylesterase 1G [Source:MGI Symbol;Acc:MGI:88378]	3356.1	4477.6	**1.33**	0.001422
Galnt10	polypeptide N-acetylgalactosaminyltransferase 10 [Source:MGI Symbol;Acc:MGI:1890480]	95.0	126.3	**1.33**	0.003038
Mfsd9	major facilitator superfamily domain containing 9 [Source:MGI Symbol;Acc:MGI:2443548]	52.8	71.4	**1.33**	0.01491
Psmb9	proteasome (prosome, macropain) subunit, beta type 9 (large multifunctional peptidase 2) [Source:MGI Symbol;Acc:MGI:1346526]	306.9	413.3	**1.33**	0.01856
Mcm2	minichromosome maintenance complex component 2 [Source:MGI Symbol;Acc:MGI:105380]	46.7	65.5	**1.33**	0.0255
Tmem177	transmembrane protein 177 [Source:MGI Symbol;Acc:MGI:1913593]	131.7	175.2	**1.32**	3.37E-05
Suox	sulfite oxidase [Source:MGI Symbol;Acc:MGI:2446117]	1759.9	2314.9	**1.32**	3.37E-05
Psmd14	proteasome (prosome, macropain) 26S subunit, non-ATPase, 14 [Source:MGI Symbol;Acc:MGI:1913284]	717.9	937.6	**1.32**	3.69E-05

Lyplal1	lysophospholipase-like 1 [Source:MGI Symbol;Acc:MGI:2385115]	223.9	293.9	**1.32**	5.32E-05
Cryzl2	crystallin zeta like 2 [Source:MGI Symbol;Acc:MGI:2448516]	356.5	465.9	**1.32**	5.74E-05
Acad12	acyl-Coenzyme A dehydrogenase family, member 12 [Source:MGI Symbol;Acc:MGI:2443320]	174.4	233.2	**1.32**	8.92E-05
Exosc7	exosome component 7 [Source:MGI Symbol;Acc:MGI:1913696]	387.6	507.2	**1.32**	0.0001567
Dus2	dihydrouridine synthase 2 [Source:MGI Symbol;Acc:MGI:1913619]	108.5	144.6	**1.32**	0.0004466
Cfap36	cilia and flagella associated protein 36 [Source:MGI Symbol;Acc:MGI:1913994]	261.3	341.5	**1.32**	0.0007324
Aldh1a7	aldehyde dehydrogenase family 1, subfamily A7 [Source:MGI Symbol;Acc:MGI:1347050]	3662.6	4875.5	**1.32**	0.001087
Nabp1	nucleic acid binding protein 1 [Source:MGI Symbol;Acc:MGI:1923258]	158.5	213.6	**1.32**	0.001871
Srsf3	serine/arginine-rich splicing factor 3 [Source:MGI Symbol;Acc:MGI:98285]	389.7	528.7	**1.32**	0.001871
Smc2	structural maintenance of chromosomes 2 [Source:MGI Symbol;Acc:MGI:106067]	44.0	60.7	**1.32**	0.01667
Zcwpw1	zinc finger, CW type with PWWP domain 1 [Source:MGI Symbol;Acc:MGI:2685899]	34.3	47.3	**1.32**	0.01771
Fancg	Fanconi anemia, complementation group G [Source:MGI Symbol;Acc:MGI:1926471]	28.8	40.0	**1.32**	0.02337
Cxcl11	chemokine (C-X-C motif) ligand 11 [Source:MGI Symbol;Acc:MGI:1860203]	25.3	36.3	**1.32**	0.03225
Dglucy	D-glutamate cyclase [Source:MGI Symbol;Acc:MGI:2444813]	683.6	929.2	**1.31**	0.0001567
Tbc1d7	TBC1 domain family, member 7 [Source:MGI Symbol;Acc:MGI:1914296]	109.3	144.2	**1.31**	0.004195
Trim12c	tripartite motif-containing 12C [Source:MGI Symbol;Acc:MGI:4821183]	156.8	205.6	**1.31**	0.006578
Akr1c20	aldo-keto reductase family 1, member C20 [Source:MGI Symbol;Acc:MGI:2151104]	1595.1	2124.7	**1.31**	0.00686
Ccdc28a	coiled-coil domain containing 28A [Source:MGI Symbol;Acc:MGI:2443508]	131.2	171.3	**1.31**	0.007319
Cebpe	CCAAT/enhancer binding protein (C/EBP), epsilon [Source:MGI Symbol;Acc:MGI:103572]	149.0	210.9	**1.31**	0.02063
Ctcflos	CCCTC-binding factor (zinc finger protein)-like, opposite strand [Source:MGI Symbol;Acc:MGI:1921411]	43.1	60.1	**1.31**	0.02094
Snx32	sorting nexin 32 [Source:MGI Symbol;Acc:MGI:2444704]	29.9	43.3	**1.31**	0.03
Serpinb8	serine (or cysteine) peptidase inhibitor, clade B, member 8 [Source:MGI Symbol;Acc:MGI:894657]	101.2	133.5	**1.31**	0.03513
Gpx1	glutathione peroxidase 1 [Source:MGI Symbol;Acc:MGI:104887]	23709.1	32687.9	**1.31**	0.04458
Snx15	sorting nexin 15 [Source:MGI Symbol;Acc:MGI:1916274]	167.7	214.8	**1.30**	0.000138
Tmx2	thioredoxin-related transmembrane protein 2 [Source:MGI Symbol;Acc:MGI:1914208]	331.2	422.3	**1.30**	0.0002778
Actg1	actin, gamma, cytoplasmic 1 [Source:MGI Symbol;Acc:MGI:87906]	336.8	443.6	**1.30**	0.001977
Nmi	N-myc (and STAT) interactor [Source:MGI Symbol;Acc:MGI:1928368]	283.1	368.6	**1.30**	0.003869
Wdr25	WD repeat domain 25 [Source:MGI Symbol;Acc:MGI:3045255]	53.3	68.7	**1.30**	0.004045
Mrps6	mitochondrial ribosomal protein S6 [Source:MGI Symbol;Acc:MGI:2153111]	92.5	122.0	**1.30**	0.005031
Mpc1	mitochondrial pyruvate carrier 1 [Source:MGI Symbol;Acc:MGI:1915240]	915.4	1192.7	**1.30**	0.005107
Parp11	poly (ADP-ribose) polymerase family, member 11 [Source:MGI Symbol;Acc:MGI:2141505]	63.8	86.9	**1.30**	0.01101
Papss1	3'-phosphoadenosine 5'-phosphosulfate synthase 1 [Source:MGI Symbol;Acc:MGI:1330587]	81.3	105.4	**1.30**	0.01567

Klhdc7a	kelch domain containing 7A [Source:MGI Symbol;Acc:MGI:2444612]	855.6	1160.3	**1.30**	0.01727
Gtf2h5	general transcription factor IIH, polypeptide 5 [Source:MGI Symbol;Acc:MGI:107227]	523.9	681.8	**1.30**	0.02038
Ccdc77	coiled-coil domain containing 77 [Source:MGI Symbol;Acc:MGI:1914450]	38.2	52.2	**1.30**	0.02186
Samd9l	sterile alpha motif domain containing 9-like [Source:MGI Symbol;Acc:MGI:1343184]	266.6	360.0	**1.30**	0.02814
Neat1	nuclear paraspeckle assembly transcript 1 (non-protein coding) [Source:MGI Symbol;Acc:MGI:1914211]	699.3	1038.5	**1.30**	0.04465
Rgn	regucalcin [Source:MGI Symbol;Acc:MGI:108024]	19422.5	25408.2	**1.29**	1.28E-05
Sae1	SUMO1 activating enzyme subunit 1 [Source:MGI Symbol;Acc:MGI:1929264]	303.8	388.6	**1.29**	0.0004051
Khdrbs3	KH domain containing, RNA binding, signal transduction associated 3 [Source:MGI Symbol;Acc:MGI:1313312]	372.9	500.5	**1.29**	0.001198
Tpgs2	tubulin polyglutamylase complex subunit 2 [Source:MGI Symbol;Acc:MGI:1913898]	74.7	94.5	**1.29**	0.002458
Hsp90aa1	heat shock protein 90, alpha (cytosolic), class A member 1 [Source:MGI Symbol;Acc:MGI:96250]	733.0	951.3	**1.29**	0.00254
Zfp938	zinc finger protein 938 [Source:MGI Symbol;Acc:MGI:3621440]	67.1	83.3	**1.29**	0.002546
Snrnp35	small nuclear ribonucleoprotein 35 (U11/U12) [Source:MGI Symbol;Acc:MGI:1923417]	73.8	97.5	**1.29**	0.003364
Zmat3	zinc finger matrin type 3 [Source:MGI Symbol;Acc:MGI:1195270]	96.0	124.6	**1.29**	0.004423
Tmem140	transmembrane protein 140 [Source:MGI Symbol;Acc:MGI:1915737]	332.9	431.6	**1.29**	0.005283
Cyp8b1	cytochrome P450, family 8, subfamily b, polypeptide 1 [Source:MGI Symbol;Acc:MGI:1338044]	6561.5	8134.4	**1.29**	0.009164
Eipr1	EARP complex and GARP complex interacting protein 1 [Source:MGI Symbol;Acc:MGI:1289332]	137.8	179.9	**1.29**	0.009331
Smyd4	SET and MYND domain containing 4 [Source:MGI Symbol;Acc:MGI:2442796]	58.3	76.5	**1.29**	0.009505
Saa4	serum amyloid A 4 [Source:MGI Symbol;Acc:MGI:98224]	2952.7	4210.0	**1.29**	0.01925
Hp	haptoglobin [Source:MGI Symbol;Acc:MGI:96211]	37899.8	49197.7	**1.29**	0.02805
Atox1	antioxidant 1 copper chaperone [Source:MGI Symbol;Acc:MGI:1333855]	1234.7	1619.8	**1.29**	0.0286
Agbl3	ATP/GTP binding protein-like 3 [Source:MGI Symbol;Acc:MGI:1923473]	61.3	82.0	**1.29**	0.02991
Aen	apoptosis enhancing nuclease [Source:MGI Symbol;Acc:MGI:1915298]	131.7	177.2	**1.29**	0.03655
Zfp386	zinc finger protein 386 (Kruppel-like) [Source:MGI Symbol;Acc:MGI:1930708]	150.8	190.5	**1.28**	6.08E-05
Rexo2	RNA exonuclease 2 [Source:MGI Symbol;Acc:MGI:1888981]	802.2	1029.1	**1.28**	0.000232
Bet1	Bet1 golgi vesicular membrane trafficking protein [Source:MGI Symbol;Acc:MGI:1343104]	791.1	997.2	**1.28**	0.003593
Trmt12	tRNA methyltranferase 12 [Source:MGI Symbol;Acc:MGI:1915510]	63.7	86.1	**1.28**	0.004831
Tcta	T cell leukemia translocation altered gene [Source:MGI Symbol;Acc:MGI:1918829]	269.7	350.5	**1.28**	0.005283
Mlst8	MTOR associated protein, LST8 homolog (S. cerevisiae) [Source:MGI Symbol;Acc:MGI:1929514]	109.7	147.2	**1.28**	0.008121
Tcp11l1	t-complex 11 like 1 [Source:MGI Symbol;Acc:MGI:2444263]	60.7	76.6	**1.28**	0.01245
Uba5	ubiquitin-like modifier activating enzyme 5 [Source:MGI Symbol;Acc:MGI:1913913]	393.8	522.5	**1.28**	0.01491
Mad2l1	MAD2 mitotic arrest deficient-like 1 [Source:MGI Symbol;Acc:MGI:1860374]	53.3	68.7	**1.28**	0.01757
Exoc4	exocyst complex component 4 [Source:MGI Symbol;Acc:MGI:1096376]	356.3	473.1	**1.28**	0.01786

Ermp1	endoplasmic reticulum metallopeptidase 1 [Source:MGI Symbol;Acc:MGI:106250]	643.4	846.7	**1.28**	0.02368
Ndufb3	NADH:ubiquinone oxidoreductase subunit B3 [Source:MGI Symbol;Acc:MGI:1913745]	839.2	1112.5	**1.28**	0.03129
Clpb	ClpB caseinolytic peptidase B [Source:MGI Symbol;Acc:MGI:1100517]	599.9	769.1	**1.27**	2.01E-06
Gatb	glutamyl-tRNA(Gln) amidotransferase, subunit B [Source:MGI Symbol;Acc:MGI:2442496]	618.4	781.1	**1.27**	4.18E-05
Zfand2a	zinc finger, AN1-type domain 2A [Source:MGI Symbol;Acc:MGI:2140729]	163.5	211.3	**1.27**	0.002074
Pex11g	peroxisomal biogenesis factor 11 gamma [Source:MGI Symbol;Acc:MGI:1920905]	775.0	995.6	**1.27**	0.00289
Trub2	TruB pseudouridine (psi) synthase family member 2 [Source:MGI Symbol;Acc:MGI:2442186]	171.4	214.6	**1.27**	0.003623
Ntpcr	nucleoside-triphosphatase, cancer-related [Source:MGI Symbol;Acc:MGI:1913816]	112.1	144.9	**1.27**	0.004413
Wwtr1	WW domain containing transcription regulator 1 [Source:MGI Symbol;Acc:MGI:1917649]	389.7	485.0	**1.27**	0.008299
Fahd2a	fumarylacetoacetate hydrolase domain containing 2A [Source:MGI Symbol;Acc:MGI:1915376]	281.3	365.4	**1.27**	0.01186
Nhp2	NHP2 ribonucleoprotein [Source:MGI Symbol;Acc:MGI:1098547]	309.4	399.0	**1.27**	0.02519
Slc35b1	solute carrier family 35, member B1 [Source:MGI Symbol;Acc:MGI:1343133]	548.6	701.3	**1.27**	0.03051
Coq7	demethyl-Q 7 [Source:MGI Symbol;Acc:MGI:107207]	380.5	484.1	**1.27**	0.03221
Gda	guanine deaminase [Source:MGI Symbol;Acc:MGI:95678]	436.8	588.5	**1.27**	0.04204
Abhd3	abhydrolase domain containing 3 [Source:MGI Symbol;Acc:MGI:2147183]	778.8	975.7	**1.27**	7.45E-05
Plscr2	phospholipid scramblase 2 [Source:MGI Symbol;Acc:MGI:1270860]	388.0	502.3	**1.27**	0.0008111
Mtrf1	mitochondrial translational release factor 1 [Source:MGI Symbol;Acc:MGI:2384815]	124.9	160.2	**1.27**	0.00137
Thop1	thimet oligopeptidase 1 [Source:MGI Symbol;Acc:MGI:1354165]	112.9	144.5	**1.27**	0.002601
Akr1a1	aldo-keto reductase family 1, member A1 (aldehyde reductase) [Source:MGI Symbol;Acc:MGI:1929955]	6468.1	8241.9	**1.27**	0.003958
Slc25a10	solute carrier family 25 (mitochondrial carrier, dicarboxylate transporter), member 10 [Source:MGI Symbol;Acc:MGI:1353497]	4270.0	5447.9	**1.27**	0.004056
Skp1a	S-phase kinase-associated protein 1A [Source:MGI Symbol;Acc:MGI:103575]	2997.5	3816.2	**1.27**	0.005089
Mrpl45	mitochondrial ribosomal protein L45 [Source:MGI Symbol;Acc:MGI:1914286]	409.6	511.9	**1.27**	0.008194
Hmgcl	3-hydroxy-3-methylglutaryl-Coenzyme A lyase [Source:MGI Symbol;Acc:MGI:96158]	4271.8	5399.7	**1.27**	0.01267
Det1	de-etiolated homolog 1 (Arabidopsis) [Source:MGI Symbol;Acc:MGI:1923625]	121.0	147.9	**1.27**	0.0144
Tdrp	testis development related protein [Source:MGI Symbol;Acc:MGI:1919398]	256.4	333.3	**1.27**	0.01535
Tmem189	transmembrane protein 189 [Source:MGI Symbol;Acc:MGI:2142624]	219.4	287.3	**1.27**	0.0428
Mcm8	minichromosome maintenance 8 homologous recombination repair factor [Source:MGI Symbol;Acc:MGI:1913884]	37.4	48.9	**1.27**	0.04734
Ccng1	cyclin G1 [Source:MGI Symbol;Acc:MGI:102890]	760.9	958.5	**1.26**	3.51E-05
Letm1	leucine zipper-EF-hand containing transmembrane protein 1 [Source:MGI Symbol;Acc:MGI:1932557]	1174.3	1472.8	**1.26**	6.78E-05
Mrps27	mitochondrial ribosomal protein S27 [Source:MGI Symbol;Acc:MGI:1919064]	435.5	523.9	**1.26**	0.0001823
Dnajb2	DnaJ heat shock protein family (Hsp40) member B2 [Source:MGI Symbol;Acc:MGI:1928739]	805.7	998.9	**1.26**	0.0006633

Micu2	mitochondrial calcium uptake 2 [Source:MGI Symbol;Acc:MGI:1915764]	737.5	906.1	**1.26**	0.001605
Ifi35	interferon-induced protein 35 [Source:MGI Symbol;Acc:MGI:1917360]	355.3	454.1	**1.26**	0.009185
Acat1	acetyl-Coenzyme A acetyltransferase 1 [Source:MGI Symbol;Acc:MGI:87870]	8981.0	11475.2	**1.26**	0.02274
Slc25a40	solute carrier family 25, member 40 [Source:MGI Symbol;Acc:MGI:2442486]	59.3	76.2	**1.26**	0.02715
Snupn	snurportin 1 [Source:MGI Symbol;Acc:MGI:1913319]	73.9	96.6	**1.26**	0.02928
Trim34a	tripartite motif-containing 34A [Source:MGI Symbol;Acc:MGI:2137359]	72.8	91.7	**1.26**	0.044
Gstm4	glutathione S-transferase, mu 4 [Source:MGI Symbol;Acc:MGI:95862]	738.8	915.7	**1.26**	0.04574
Nudt1	nudix (nucleoside diphosphate linked moiety X)-type motif 1 [Source:MGI Symbol;Acc:MGI:109280]	306.3	394.1	**1.26**	0.047
Slc50a1	solute carrier family 50 (sugar transporter), member 1 [Source:MGI Symbol;Acc:MGI:107417]	88.3	116.1	**1.26**	0.04876
Acsf3	acyl-CoA synthetase family member 3 [Source:MGI Symbol;Acc:MGI:2182591]	587.3	735.9	**1.25**	2.28E-07
Msra	methionine sulfoxide reductase A [Source:MGI Symbol;Acc:MGI:106916]	2388.5	2961.4	**1.25**	0.0006002
Smarca4	SWI/SNF related, matrix associated, actin dependent regulator of chromatin, subfamily a, member 4 [Source:MGI Symbol;Acc:MGI:88192]	761.7	961.6	**1.25**	0.002306
Sugct	succinyl-CoA glutarate-CoA transferase [Source:MGI Symbol;Acc:MGI:1923221]	635.2	786.8	**1.25**	0.003168
Mrpl1	mitochondrial ribosomal protein L1 [Source:MGI Symbol;Acc:MGI:2137202]	494.2	605.9	**1.25**	0.007243
Jpt2	Jupiter microtubule associated homolog 2 [Source:MGI Symbol;Acc:MGI:1196260]	314.2	383.5	**1.25**	0.009862
Higd1a	HIG1 domain family, member 1A [Source:MGI Symbol;Acc:MGI:1930666]	409.5	521.9	**1.25**	0.01321
Psmb5	proteasome (prosome, macropain) subunit, beta type 5 [Source:MGI Symbol;Acc:MGI:1194513]	449.7	555.0	**1.25**	0.02174
Dut	deoxyuridine triphosphatase [Source:MGI Symbol;Acc:MGI:1346051]	132.7	166.9	**1.25**	0.02274
Enpp2	ectonucleotide pyrophosphatase/phosphodiesterase 2 [Source:MGI Symbol;Acc:MGI:1321390]	1592.0	2030.3	**1.25**	0.02334
Nt5c	5',3'-nucleotidase, cytosolic [Source:MGI Symbol;Acc:MGI:1354954]	243.9	308.7	**1.25**	0.03245
Htatip2	HIV-1 Tat interactive protein 2 [Source:MGI Symbol;Acc:MGI:1859271]	457.1	557.5	**1.25**	0.03252
Tmem120a	transmembrane protein 120A [Source:MGI Symbol;Acc:MGI:2686991]	879.8	1139.3	**1.25**	0.0391
Gpr39	G protein-coupled receptor 39 [Source:MGI Symbol;Acc:MGI:1918361]	92.4	116.6	**1.25**	0.04179
Anapc10	anaphase promoting complex subunit 10 [Source:MGI Symbol;Acc:MGI:1916249]	48.4	61.1	**1.25**	0.0472
Tpk1	thiamine pyrophosphokinase [Source:MGI Symbol;Acc:MGI:1352500]	348.6	439.5	**1.24**	2.26E-06
Tpmt	thiopurine methyltransferase [Source:MGI Symbol;Acc:MGI:98812]	733.6	904.5	**1.24**	2.65E-05
Pno1	partner of NOB1 homolog [Source:MGI Symbol;Acc:MGI:1913499]	504.5	623.8	**1.24**	2.88E-05
Ccnc	cyclin C [Source:MGI Symbol;Acc:MGI:1858199]	286.8	356.5	**1.24**	4.24E-05
Atg5	autophagy related 5 [Source:MGI Symbol;Acc:MGI:1277186]	938.3	1143.3	**1.24**	9.74E-05
Stx4a	syntaxin 4A (placental) [Source:MGI Symbol;Acc:MGI:893577]	524.4	630.9	**1.24**	0.001317
N6amt1	N-6 adenine-specific DNA methyltransferase 1 (putative) [Source:MGI Symbol;Acc:MGI:1915018]	157.1	191.9	**1.24**	0.002354
Mrps22	mitochondrial ribosomal protein S22 [Source:MGI Symbol;Acc:MGI:1928137]	303.0	367.4	**1.24**	0.004936

Iscu	iron-sulfur cluster assembly enzyme [Source:MGI Symbol;Acc:MGI:1913633]	895.6	1102.6	**1.24**	0.007729
Pold2	polymerase (DNA directed), delta 2, regulatory subunit [Source:MGI Symbol;Acc:MGI:1097163]	191.1	235.5	**1.24**	0.007931
Zfp367	zinc finger protein 367 [Source:MGI Symbol;Acc:MGI:2442266]	236.2	291.1	**1.24**	0.008962
Pex16	peroxisomal biogenesis factor 16 [Source:MGI Symbol;Acc:MGI:1338829]	1632.6	2024.5	**1.24**	0.01258
Faim	Fas apoptotic inhibitory molecule [Source:MGI Symbol;Acc:MGI:1344387]	111.2	138.5	**1.24**	0.01275
Pgp	phosphoglycolate phosphatase [Source:MGI Symbol;Acc:MGI:1914328]	423.0	510.1	**1.24**	0.0164
Cutal	cutA divalent cation tolerance homolog-like [Source:MGI Symbol;Acc:MGI:1925246]	739.8	904.5	**1.24**	0.01734
Lzic	leucine zipper and CTNNBIP1 domain containing [Source:MGI Symbol;Acc:MGI:1916401]	80.2	100.9	**1.24**	0.02156
Entpd5	ectonucleoside triphosphate diphosphohydrolase 5 [Source:MGI Symbol;Acc:MGI:1321385]	4547.6	5691.9	**1.24**	0.0229
Chd1l	chromodomain helicase DNA binding protein 1-like [Source:MGI Symbol;Acc:MGI:1915308]	162.4	206.1	**1.24**	0.03949
D11Wsu47e	DNA segment, Chr 11, Wayne State University 47, expressed [Source:MGI Symbol;Acc:MGI:106356]	56.8	72.4	**1.24**	0.045
Kif3a	kinesin family member 3A [Source:MGI Symbol;Acc:MGI:107689]	46.0	57.6	**1.24**	0.04547
Gcdh	glutaryl-Coenzyme A dehydrogenase [Source:MGI Symbol;Acc:MGI:104541]	9260.5	11527.4	**1.23**	0.0003329
Nt5dc1	5'-nucleotidase domain containing 1 [Source:MGI Symbol;Acc:MGI:2442446]	301.6	374.3	**1.23**	0.001303
Rpa1	replication protein A1 [Source:MGI Symbol;Acc:MGI:1915525]	462.3	567.5	**1.23**	0.001733
Rtn4ip1	reticulon 4 interacting protein 1 [Source:MGI Symbol;Acc:MGI:2178759]	388.1	491.8	**1.23**	0.001784
Psmc4	proteasome (prosome, macropain) 26S subunit, ATPase, 4 [Source:MGI Symbol;Acc:MGI:1346093]	1245.0	1537.8	**1.23**	0.002014
Dhrs1	dehydrogenase/reductase (SDR family) member 1 [Source:MGI Symbol;Acc:MGI:1196314]	1897.6	2374.8	**1.23**	0.005236
Prpsap2	phosphoribosyl pyrophosphate synthetase-associated protein 2 [Source:MGI Symbol;Acc:MGI:2384838]	109.9	135.0	**1.23**	0.005502
Spryd7	SPRY domain containing 7 [Source:MGI Symbol;Acc:MGI:1913924]	422.7	504.0	**1.23**	0.006564
Hadh	hydroxyacyl-Coenzyme A dehydrogenase [Source:MGI Symbol;Acc:MGI:96009]	6347.7	7844.6	**1.23**	0.008759
Triap1	TP53 regulated inhibitor of apoptosis 1 [Source:MGI Symbol;Acc:MGI:1916326]	280.1	350.7	**1.23**	0.009164
Sec22a	SEC22 homolog A, vesicle trafficking protein [Source:MGI Symbol;Acc:MGI:2447876]	241.8	293.3	**1.23**	0.009339
Pop4	processing of precursor 4, ribonuclease P/MRP family, (S. cerevisiae) [Source:MGI Symbol;Acc:MGI:1913411]	229.1	283.4	**1.23**	0.01123
Cox19	cytochrome c oxidase assembly protein 19 [Source:MGI Symbol;Acc:MGI:1915283]	494.6	614.8	**1.23**	0.0144
Tmem43	transmembrane protein 43 [Source:MGI Symbol;Acc:MGI:1921372]	149.5	183.9	**1.23**	0.02112
Dtymk	deoxythymidylate kinase [Source:MGI Symbol;Acc:MGI:108396]	205.4	250.1	**1.23**	0.02213
Fdxacb1	ferredoxin-fold anticodon binding domain containing 1 [Source:MGI Symbol;Acc:MGI:3584513]	74.7	95.4	**1.23**	0.02257
Rcan1	regulator of calcineurin 1 [Source:MGI Symbol;Acc:MGI:1890564]	274.3	345.7	**1.23**	0.02859
Pdrg1	p53 and DNA damage regulated 1 [Source:MGI Symbol;Acc:MGI:1915809]	290.6	369.3	**1.23**	0.03407
Prr13	proline rich 13 [Source:MGI Symbol;Acc:MGI:1913401]	510.4	638.1	**1.23**	0.03702
Rbm43	RNA binding motif protein 43 [Source:MGI Symbol;Acc:MGI:1918934]	79.7	97.3	**1.23**	0.03875

Pgrmc1	progesterone receptor membrane component 1 [Source:MGI Symbol;Acc:MGI:1858305]	17801.5	21567.7	**1.22**	2.75E-05
Opa3	optic atrophy 3 [Source:MGI Symbol;Acc:MGI:2686271]	797.4	962.9	**1.22**	3.90E-05
Mccc2	methylcrotonoyl-Coenzyme A carboxylase 2 (beta) [Source:MGI Symbol;Acc:MGI:1925288]	1003.7	1240.3	**1.22**	0.0001868
Mrpl16	mitochondrial ribosomal protein L16 [Source:MGI Symbol;Acc:MGI:2137219]	575.4	693.8	**1.22**	0.0009421
Slc22a1	solute carrier family 22 (organic cation transporter), member 1 [Source:MGI Symbol;Acc:MGI:108111]	5848.9	7227.5	**1.22**	0.003961
Cyb5b	cytochrome b5 type B [Source:MGI Symbol;Acc:MGI:1913677]	8335.7	9796.9	**1.22**	0.004886
Nudcd2	NudC domain containing 2 [Source:MGI Symbol;Acc:MGI:1277103]	432.8	527.2	**1.22**	0.00585
Nif3l1	Ngg1 interacting factor 3-like 1 (S. pombe) [Source:MGI Symbol;Acc:MGI:1929485]	168.3	210.0	**1.22**	0.01207
Acy3	aspartoacylase (aminoacylase) 3 [Source:MGI Symbol;Acc:MGI:1918920]	1148.8	1451.3	**1.22**	0.01251
Hprt	hypoxanthine guanine phosphoribosyl transferase [Source:MGI Symbol;Acc:MGI:96217]	957.7	1157.2	**1.22**	0.01638
Nipal3	NIPA-like domain containing 3 [Source:MGI Symbol;Acc:MGI:1921802]	176.2	222.5	**1.22**	0.01777
Sars2	seryl-aminoacyl-tRNA synthetase 2 [Source:MGI Symbol;Acc:MGI:1919234]	127.0	159.8	**1.22**	0.01785
Psma1	proteasome (prosome, macropain) subunit, alpha type 1 [Source:MGI Symbol;Acc:MGI:1347005]	1363.1	1657.4	**1.22**	0.01856
Cars2	cysteinyl-tRNA synthetase 2 (mitochondrial)(putative) [Source:MGI Symbol;Acc:MGI:1919191]	185.5	227.5	**1.22**	0.0187
Tbrg1	transforming growth factor beta regulated gene 1 [Source:MGI Symbol;Acc:MGI:1100877]	621.4	758.2	**1.22**	0.02047
Phkb	phosphorylase kinase beta [Source:MGI Symbol;Acc:MGI:97578]	344.8	424.7	**1.22**	0.02186
Ccdc167	coiled-coil domain containing 167 [Source:MGI Symbol;Acc:MGI:1915847]	105.4	131.1	**1.22**	0.02372
Arhgap18	Rho GTPase activating protein 18 [Source:MGI Symbol;Acc:MGI:1921160]	194.4	232.3	**1.22**	0.027
Wdr83	WD repeat domain containing 83 [Source:MGI Symbol;Acc:MGI:1915086]	221.7	272.5	**1.22**	0.02996
Arpp19	cAMP-regulated phosphoprotein 19 [Source:MGI Symbol;Acc:MGI:1891691]	1005.3	1218.5	**1.22**	0.04093
Agpat2	1-acylglycerol-3-phosphate O-acyltransferase 2 (lysophosphatidic acid acyltransferase, beta) [Source:MGI Symbol;Acc:MGI:1914762]	3369.1	4012.9	**1.22**	0.04345
Cct3	chaperonin containing Tcp1, subunit 3 (gamma) [Source:MGI Symbol;Acc:MGI:104708]	1174.2	1420.2	**1.21**	0.00137
Hspa14	heat shock protein 14 [Source:MGI Symbol;Acc:MGI:1354164]	218.5	264.3	**1.21**	0.001951
Ndufaf1	NADH:ubiquinone oxidoreductase complex assembly factor 1 [Source:MGI Symbol;Acc:MGI:1916952]	413.2	502.0	**1.21**	0.00204
Fkbp4	FK506 binding protein 4 [Source:MGI Symbol;Acc:MGI:95543]	2241.0	2738.6	**1.21**	0.005236
Ttc33	tetratricopeptide repeat domain 33 [Source:MGI Symbol;Acc:MGI:1914765]	288.1	348.9	**1.21**	0.007534
Myh14	myosin, heavy polypeptide 14 [Source:MGI Symbol;Acc:MGI:1919210]	370.7	456.5	**1.21**	0.009164
Taldo1	transaldolase 1 [Source:MGI Symbol;Acc:MGI:1274789]	1606.2	1950.9	**1.21**	0.01655
Stx6	syntaxin 6 [Source:MGI Symbol;Acc:MGI:1926235]	139.7	165.3	**1.21**	0.01661
Lrrc61	leucine rich repeat containing 61 [Source:MGI Symbol;Acc:MGI:2652848]	112.1	135.9	**1.21**	0.02012
Dnajc9	DnaJ heat shock protein family (Hsp40) member C9 [Source:MGI Symbol;Acc:MGI:1915326]	90.0	108.7	**1.21**	0.02318
Esd	esterase D/formylglutathione hydrolase [Source:MGI Symbol;Acc:MGI:95421]	3489.3	4217.2	**1.21**	0.02928

Ppcs	phosphopantothenoylcysteine synthetase [Source:MGI Symbol;Acc:MGI:1915237]	333.9	411.3	**1.21**	0.03306
Cycs	cytochrome c, somatic [Source:MGI Symbol;Acc:MGI:88578]	464.4	557.3	**1.21**	0.03317
Hikeshi	heat shock protein nuclear import factor [Source:MGI Symbol;Acc:MGI:96738]	209.2	257.2	**1.21**	0.03834
Mdfic	MyoD family inhibitor domain containing [Source:MGI Symbol;Acc:MGI:104611]	381.8	461.3	**1.21**	0.04194
Gpatch11	G patch domain containing 11 [Source:MGI Symbol;Acc:MGI:1858435]	110.0	137.4	**1.21**	0.04378
Ufm1	ubiquitin-fold modifier 1 [Source:MGI Symbol;Acc:MGI:1915140]	751.3	897.6	**1.21**	2.99E-06
Srr	serine racemase [Source:MGI Symbol;Acc:MGI:1351636]	1738.8	2118.9	**1.21**	0.000289
Lztfl1	leucine zipper transcription factor-like 1 [Source:MGI Symbol;Acc:MGI:1934860]	152.5	184.3	**1.21**	0.003182
Gfm2	G elongation factor, mitochondrial 2 [Source:MGI Symbol;Acc:MGI:2444783]	697.1	848.1	**1.21**	0.003414
Mpst	mercaptopyruvate sulfurtransferase [Source:MGI Symbol;Acc:MGI:2179733]	1681.3	2059.4	**1.21**	0.003815
Psmc5	protease (prosome, macropain) 26S subunit, ATPase 5 [Source:MGI Symbol;Acc:MGI:105047]	965.7	1161.3	**1.21**	0.007175
Rps6kc1	ribosomal protein S6 kinase polypeptide 1 [Source:MGI Symbol;Acc:MGI:2443419]	152.5	180.6	**1.21**	0.01284
Msrb2	methionine sulfoxide reductase B2 [Source:MGI Symbol;Acc:MGI:1923717]	224.4	267.0	**1.21**	0.01284
Scp2-ps2	sterol carrier protein 2, pseudogene 2 [Source:MGI Symbol;Acc:MGI:107679]	255.9	308.8	**1.21**	0.01995
Fbxw9	F-box and WD-40 domain protein 9 [Source:MGI Symbol;Acc:MGI:1915878]	526.2	621.2	**1.21**	0.02435
Ccdc124	coiled-coil domain containing 124 [Source:MGI Symbol;Acc:MGI:1916403]	522.4	637.0	**1.21**	0.03346
Coq4	coenzyme Q4 [Source:MGI Symbol;Acc:MGI:1098826]	105.6	128.8	**1.21**	0.03629
Spg7	SPG7, paraplegin matrix AAA peptidase subunit [Source:MGI Symbol;Acc:MGI:2385906]	733.6	869.7	**1.20**	1.75E-06
Ugt2b36	UDP glucuronosyltransferase 2 family, polypeptide B36 [Source:MGI Symbol;Acc:MGI:3576103]	10062.0	12050.7	**1.20**	0.0001305
Ldhd	lactate dehydrogenase D [Source:MGI Symbol;Acc:MGI:106428]	2922.0	3497.7	**1.20**	0.0002778
Msantd4	Myb/SANT-like DNA-binding domain containing 4 with coiled-coils [Source:MGI Symbol;Acc:MGI:1925350]	365.0	429.1	**1.20**	0.0003551
Flywch1	FLYWCH-type zinc finger 1 [Source:MGI Symbol;Acc:MGI:2442638]	256.9	306.1	**1.20**	0.00178
Bphl	biphenyl hydrolase-like (serine hydrolase, breast epithelial mucin-associated antigen) [Source:MGI Symbol;Acc:MGI:1915271]	2912.7	3507.8	**1.20**	0.00281
Osgepl1	O-sialoglycoprotein endopeptidase-like 1 [Source:MGI Symbol;Acc:MGI:1919335]	243.1	289.3	**1.20**	0.005559
Pafah2	platelet-activating factor acetylhydrolase 2 [Source:MGI Symbol;Acc:MGI:2140321]	1775.8	2155.1	**1.20**	0.005869
Prpf19	pre-mRNA processing factor 19 [Source:MGI Symbol;Acc:MGI:106247]	1597.0	1903.5	**1.20**	0.007561
Mcat	malonyl CoA:ACP acyltransferase (mitochondrial) [Source:MGI Symbol;Acc:MGI:2388651]	458.3	538.3	**1.20**	0.01012
Psmd8	proteasome (prosome, macropain) 26S subunit, non-ATPase, 8 [Source:MGI Symbol;Acc:MGI:1888669]	1872.9	2212.2	**1.20**	0.01299
Cc2d1b	coiled-coil and C2 domain containing 1B [Source:MGI Symbol;Acc:MGI:2443076]	156.8	195.3	**1.20**	0.01764
Hsd17b12	hydroxysteroid (17-beta) dehydrogenase 12 [Source:MGI Symbol;Acc:MGI:1926967]	2982.7	3518.8	**1.20**	0.02041
Smim14	small integral membrane protein 14 [Source:MGI Symbol;Acc:MGI:1915802]	903.2	1088.2	**1.20**	0.02517

Ifngr2	interferon gamma receptor 2 [Source:MGI Symbol;Acc:MGI:107654]	450.8	537.8	**1.20**	0.029
Laptm4b	lysosomal-associated protein transmembrane 4B [Source:MGI Symbol;Acc:MGI:1890494]	318.9	375.4	**1.20**	0.03181
Swsap1	SWIM type zinc finger 7 associated protein 1 [Source:MGI Symbol;Acc:MGI:1914212]	81.6	97.4	**1.20**	0.0356
Pebp1	phosphatidylethanolamine binding protein 1 [Source:MGI Symbol;Acc:MGI:1344408]	1086.4	1295.9	**1.20**	0.04574
Tsr2	TSR2 20S rRNA accumulation [Source:MGI Symbol;Acc:MGI:1916749]	183.4	217.7	**1.20**	0.0479
Rmnd5b	required for meiotic nuclear division 5 homolog B [Source:MGI Symbol;Acc:MGI:1913339]	205.1	248.7	**1.20**	0.04843
Adhfe1	alcohol dehydrogenase, iron containing, 1 [Source:MGI Symbol;Acc:MGI:1923437]	1757.4	2118.9	**1.19**	0.0005925
Pmpcb	peptidase (mitochondrial processing) beta [Source:MGI Symbol;Acc:MGI:1920328]	2011.5	2359.3	**1.19**	0.0006006
Atg7	autophagy related 7 [Source:MGI Symbol;Acc:MGI:1921494]	723.1	852.9	**1.19**	0.001784
Gstz1	glutathione transferase zeta 1 (maleylacetoacetate isomerase) [Source:MGI Symbol;Acc:MGI:1341859]	11913.1	14202.9	**1.19**	0.002955
Mrpl44	mitochondrial ribosomal protein L44 [Source:MGI Symbol;Acc:MGI:1916413]	390.1	455.2	**1.19**	0.00304
Slc48a1	solute carrier family 48 (heme transporter), member 1 [Source:MGI Symbol;Acc:MGI:1914989]	702.1	830.3	**1.19**	0.004146
Abhd4	abhydrolase domain containing 4 [Source:MGI Symbol;Acc:MGI:1915938]	752.0	893.5	**1.19**	0.004195
Micu1	mitochondrial calcium uptake 1 [Source:MGI Symbol;Acc:MGI:2384909]	475.4	567.6	**1.19**	0.004299
Klhl22	kelch-like 22 [Source:MGI Symbol;Acc:MGI:1337995]	252.8	298.3	**1.19**	0.004491
Mrrf	mitochondrial ribosome recycling factor [Source:MGI Symbol;Acc:MGI:1915121]	326.6	394.4	**1.19**	0.005738
Cmpk1	cytidine monophosphate (UMP-CMP) kinase 1 [Source:MGI Symbol;Acc:MGI:1913838]	1474.8	1717.8	**1.19**	0.01391
Thoc5	THO complex 5 [Source:MGI Symbol;Acc:MGI:1351333]	232.6	280.7	**1.19**	0.01438
Nars2	asparaginyl-tRNA synthetase 2 (mitochondrial)(putative) [Source:MGI Symbol;Acc:MGI:2142075]	329.6	394.3	**1.19**	0.01567
Fez2	fasciculation and elongation protein zeta 2 (zygin II) [Source:MGI Symbol;Acc:MGI:2675856]	513.7	616.1	**1.19**	0.03072
Rogdi	rogdi homolog [Source:MGI Symbol;Acc:MGI:1913299]	204.7	244.5	**1.19**	0.03433
Psmc2	proteasome (prosome, macropain) 26S subunit, ATPase 2 [Source:MGI Symbol;Acc:MGI:109555]	1233.1	1481.2	**1.19**	0.03523
Cox18	cytochrome c oxidase assembly protein 18 [Source:MGI Symbol;Acc:MGI:2448532]	115.1	140.6	**1.19**	0.04654
Ghitm	growth hormone inducible transmembrane protein [Source:MGI Symbol;Acc:MGI:1913342]	5430.0	6329.6	**1.18**	7.34E-05
Snap23	synaptosomal-associated protein 23 [Source:MGI Symbol;Acc:MGI:109356]	446.1	525.1	**1.18**	0.001098
Slc47a1	solute carrier family 47, member 1 [Source:MGI Symbol;Acc:MGI:1914723]	1365.7	1661.5	**1.18**	0.001303
Foxred1	FAD-dependent oxidoreductase domain containing 1 [Source:MGI Symbol;Acc:MGI:2446262]	469.9	552.7	**1.18**	0.002343
Ino80c	INO80 complex subunit C [Source:MGI Symbol;Acc:MGI:2443014]	240.6	277.2	**1.18**	0.003926
Aars2	alanyl-tRNA synthetase 2, mitochondrial [Source:MGI Symbol;Acc:MGI:2681839]	289.4	341.6	**1.18**	0.004419
Chmp2b	charged multivesicular body protein 2B [Source:MGI Symbol;Acc:MGI:1916192]	603.0	686.8	**1.18**	0.005268
Pgpep1	pyroglutamyl-peptidase I [Source:MGI Symbol;Acc:MGI:1913772]	1029.8	1238.7	**1.18**	0.006563
Tars2	threonyl-tRNA synthetase 2, mitochondrial (putative) [Source:MGI Symbol;Acc:MGI:1919057]	356.3	429.2	**1.18**	0.007227

Usp14	ubiquitin specific peptidase 14 [Source:MGI Symbol;Acc:MGI:1928898]	631.8	745.8	**1.18**	0.01186
Bcap29	B cell receptor associated protein 29 [Source:MGI Symbol;Acc:MGI:101917]	207.7	243.5	**1.18**	0.01322
Tmem18	transmembrane protein 18 [Source:MGI Symbol;Acc:MGI:2387176]	231.3	276.9	**1.18**	0.03061
Taco1	translational activator of mitochondrially encoded cytochrome c oxidase I [Source:MGI Symbol;Acc:MGI:1917457]	306.7	360.7	**1.18**	0.03903
Coq2	coenzyme Q2 4-hydroxybenzoate polyprenyltransferase [Source:MGI Symbol;Acc:MGI:1919133]	515.0	605.2	**1.18**	0.04195
Mafk	v-maf musculoaponeurotic fibrosarcoma oncogene family, protein K (avian) [Source:MGI Symbol;Acc:MGI:99951]	264.9	308.1	**1.18**	0.0479
Idh1	isocitrate dehydrogenase 1 (NADP+), soluble [Source:MGI Symbol;Acc:MGI:96413]	6909.4	7981.8	**1.18**	0.04804
Cyp2j5	cytochrome P450, family 2, subfamily j, polypeptide 5 [Source:MGI Symbol;Acc:MGI:1270149]	9741.1	11432.1	**1.17**	1.69E-05
Lias	lipoic acid synthetase [Source:MGI Symbol;Acc:MGI:1934604]	535.0	626.9	**1.17**	0.0003947
Mccc1	methylcrotonoyl-Coenzyme A carboxylase 1 (alpha) [Source:MGI Symbol;Acc:MGI:1919289]	1013.9	1192.7	**1.17**	0.0006362
Mipep	mitochondrial intermediate peptidase [Source:MGI Symbol;Acc:MGI:1917728]	564.9	666.5	**1.17**	0.002578
Bpnt1	bisphosphate 3'-nucleotidase 1 [Source:MGI Symbol;Acc:MGI:1338800]	698.0	832.7	**1.17**	0.004444
Hsd17b4	hydroxysteroid (17-beta) dehydrogenase 4 [Source:MGI Symbol;Acc:MGI:105089]	7877.5	9347.6	**1.17**	0.004645
Pepd	peptidase D [Source:MGI Symbol;Acc:MGI:97542]	718.7	857.2	**1.17**	0.005249
Tbcd	tubulin-specific chaperone d [Source:MGI Symbol;Acc:MGI:1919686]	424.1	492.8	**1.17**	0.00853
Ahsa1	AHA1, activator of heat shock protein ATPase 1 [Source:MGI Symbol;Acc:MGI:2387603]	889.0	1056.0	**1.17**	0.01072
Slc35f5	solute carrier family 35, member F5 [Source:MGI Symbol;Acc:MGI:1921400]	862.4	989.0	**1.17**	0.01099
Blmh	bleomycin hydrolase [Source:MGI Symbol;Acc:MGI:1345186]	926.3	1086.4	**1.17**	0.01817
Bckdhb	branched chain ketoacid dehydrogenase E1, beta polypeptide [Source:MGI Symbol;Acc:MGI:88137]	1064.9	1254.9	**1.17**	0.0229
Nubp1	nucleotide binding protein 1 [Source:MGI Symbol;Acc:MGI:1347073]	364.7	420.9	**1.17**	0.02671
Capn1	calpain 1 [Source:MGI Symbol;Acc:MGI:88263]	412.0	484.2	**1.17**	0.03097
Ran	RAN, member RAS oncogene family [Source:MGI Symbol;Acc:MGI:1333112]	901.0	1042.3	**1.17**	0.03245
Nrbf2	nuclear receptor binding factor 2 [Source:MGI Symbol;Acc:MGI:1354950]	142.4	169.9	**1.17**	0.03473
Dhx32	DEAH (Asp-Glu-Ala-His) box polypeptide 32 [Source:MGI Symbol;Acc:MGI:2141813]	533.6	619.8	**1.17**	0.04516
Hs1bp3	HCLS1 binding protein 3 [Source:MGI Symbol;Acc:MGI:1913224]	216.0	256.8	**1.17**	0.04929
Ppm1b	protein phosphatase 1B, magnesium dependent, beta isoform [Source:MGI Symbol;Acc:MGI:101841]	1486.8	1688.8	**1.16**	0.001087
Stradb	STE20-related kinase adaptor beta [Source:MGI Symbol;Acc:MGI:2144047]	651.4	771.6	**1.16**	0.001706
Ccdc91	coiled-coil domain containing 91 [Source:MGI Symbol;Acc:MGI:1914265]	457.5	535.7	**1.16**	0.003264
Dars2	aspartyl-tRNA synthetase 2 (mitochondrial) [Source:MGI Symbol;Acc:MGI:2442510]	272.9	323.5	**1.16**	0.01192
Rmdn3	regulator of microtubule dynamics 3 [Source:MGI Symbol;Acc:MGI:1915059]	1448.1	1679.3	**1.16**	0.01432
Riox2	ribosomal oxygenase 2 [Source:MGI Symbol;Acc:MGI:1914264]	254.1	302.0	**1.16**	0.01486

Nsfl1c	NSFL1 (p97) cofactor (p47) [Source:MGI Symbol;Acc:MGI:3042273]	539.6	630.8	**1.16**	0.01897
Sfxn5	sideroflexin 5 [Source:MGI Symbol;Acc:MGI:2137681]	532.4	632.6	**1.16**	0.02186
Spns2	spinster homolog 2 [Source:MGI Symbol;Acc:MGI:2384936]	463.4	547.9	**1.16**	0.02327
Chmp6	charged multivesicular body protein 6 [Source:MGI Symbol;Acc:MGI:3583942]	317.4	368.0	**1.16**	0.03898
Ap3s2	adaptor-related protein complex 3, sigma 2 subunit [Source:MGI Symbol;Acc:MGI:1337060]	477.4	538.8	**1.16**	0.000589
Rcbtb2	regulator of chromosome condensation (RCC1) and BTB (POZ) domain containing protein 2 [Source:MGI Symbol;Acc:MGI:1917200]	690.1	792.3	**1.16**	0.0006634
Pigk	phosphatidylinositol glycan anchor biosynthesis, class K [Source:MGI Symbol;Acc:MGI:1913863]	551.7	616.4	**1.16**	0.0008335
Nfs1	nitrogen fixation gene 1 (S. cerevisiae) [Source:MGI Symbol;Acc:MGI:1316706]	781.2	902.4	**1.16**	0.002578
Carhsp1	calcium regulated heat stable protein 1 [Source:MGI Symbol;Acc:MGI:1196368]	1766.8	2022.2	**1.16**	0.006744
Psmc6	proteasome (prosome, macropain) 26S subunit, ATPase, 6 [Source:MGI Symbol;Acc:MGI:1914339]	1310.9	1498.8	**1.16**	0.008288
Hnrnpc	heterogeneous nuclear ribonucleoprotein C [Source:MGI Symbol;Acc:MGI:107795]	1011.6	1186.1	**1.16**	0.009354
Rraga	Ras-related GTP binding A [Source:MGI Symbol;Acc:MGI:1915691]	584.9	669.9	**1.16**	0.01371
Dynll2	dynein light chain LC8-type 2 [Source:MGI Symbol;Acc:MGI:1915347]	3783.1	4296.4	**1.16**	0.01442
Rpe	ribulose-5-phosphate-3-epimerase [Source:MGI Symbol;Acc:MGI:1913896]	494.1	570.3	**1.16**	0.01737
Slc27a4	solute carrier family 27 (fatty acid transporter), member 4 [Source:MGI Symbol;Acc:MGI:1347347]	675.6	776.3	**1.16**	0.02809
Wdr41	WD repeat domain 41 [Source:MGI Symbol;Acc:MGI:2445123]	243.9	284.9	**1.16**	0.03099
Aldh6a1	aldehyde dehydrogenase family 6, subfamily A1 [Source:MGI Symbol;Acc:MGI:1915077]	8384.0	9683.2	**1.16**	0.03348
Oma1	OMA1 zinc metallopeptidase [Source:MGI Symbol;Acc:MGI:1914263]	249.6	295.6	**1.16**	0.04254
Ptpn6	protein tyrosine phosphatase, non-receptor type 6 [Source:MGI Symbol;Acc:MGI:96055]	349.4	394.9	**1.15**	0.007751
Plpbp	pyridoxal phosphate binding protein [Source:MGI Symbol;Acc:MGI:1891207]	1323.6	1529.0	**1.15**	0.009804
Brd7	bromodomain containing 7 [Source:MGI Symbol;Acc:MGI:1349766]	593.9	682.2	**1.15**	0.01264
Sh3bgrl	SH3-binding domain glutamic acid-rich protein like [Source:MGI Symbol;Acc:MGI:1930849]	2001.9	2244.5	**1.15**	0.01431
Cryz	crystallin, zeta [Source:MGI Symbol;Acc:MGI:88527]	2033.6	2329.7	**1.15**	0.01567
Dbnl	drebrin-like [Source:MGI Symbol;Acc:MGI:700006]	506.5	575.4	**1.15**	0.02074
Stx7	syntaxin 7 [Source:MGI Symbol;Acc:MGI:1858210]	290.9	331.4	**1.15**	0.02366
Psmd2	proteasome (prosome, macropain) 26S subunit, non-ATPase, 2 [Source:MGI Symbol;Acc:MGI:1096584]	2637.4	3058.5	**1.15**	0.04386
Eif2d	eukaryotic translation initiation factor 2D [Source:MGI Symbol;Acc:MGI:109342]	524.4	613.0	**1.15**	0.04396
Dele1	DAP3 binding cell death enhancer 1 [Source:MGI Symbol;Acc:MGI:1914089]	598.0	686.3	**1.14**	0.004472
Syap1	synapse associated protein 1 [Source:MGI Symbol;Acc:MGI:1914293]	1007.4	1128.7	**1.14**	0.009564
Hibch	3-hydroxyisobutyryl-Coenzyme A hydrolase [Source:MGI Symbol;Acc:MGI:1923792]	1349.7	1538.1	**1.14**	0.01119
Usp5	ubiquitin specific peptidase 5 (isopeptidase T) [Source:MGI Symbol;Acc:MGI:1347343]	1077.4	1221.8	**1.14**	0.01335
Calm1	calmodulin 1 [Source:MGI Symbol;Acc:MGI:88251]	3031.6	3422.5	**1.14**	0.01439

Klhl5	kelch-like 5 [Source:MGI Symbol;Acc:MGI:1919028]	670.9	768.2	**1.14**	0.01821
Naa35	N(alpha)-acetyltransferase 35, NatC auxiliary subunit [Source:MGI Symbol;Acc:MGI:1925939]	420.3	476.1	**1.14**	0.01956
Acads	acyl-Coenzyme A dehydrogenase, short chain [Source:MGI Symbol;Acc:MGI:87868]	3436.3	3892.4	**1.14**	0.02086
Gps1	G protein pathway suppressor 1 [Source:MGI Symbol;Acc:MGI:2384801]	525.1	596.4	**1.14**	0.02141
Mkrn1	makorin, ring finger protein, 1 [Source:MGI Symbol;Acc:MGI:1859353]	506.5	582.5	**1.14**	0.02198
Anapc4	anaphase promoting complex subunit 4 [Source:MGI Symbol;Acc:MGI:1098673]	309.6	340.2	**1.14**	0.02393
Plpp6	phospholipid phosphatase 6 [Source:MGI Symbol;Acc:MGI:1921661]	257.2	285.8	**1.14**	0.02814
Arl6ip1	ADP-ribosylation factor-like 6 interacting protein 1 [Source:MGI Symbol;Acc:MGI:1858943]	3232.8	3690.5	**1.14**	0.03273
Itm2b	integral membrane protein 2B [Source:MGI Symbol;Acc:MGI:1309517]	15649.1	17669.3	**1.14**	0.03629
Ddx1	DEAD (Asp-Glu-Ala-Asp) box polypeptide 1 [Source:MGI Symbol;Acc:MGI:2144727]	1211.8	1361.9	**1.14**	0.03655
Ubap1	ubiquitin-associated protein 1 [Source:MGI Symbol;Acc:MGI:2149543]	707.9	804.5	**1.13**	0.003689
Afg3l1	AFG3-like AAA ATPase 1 [Source:MGI Symbol;Acc:MGI:1928277]	1275.4	1437.1	**1.13**	0.004167
Ctso	cathepsin O [Source:MGI Symbol;Acc:MGI:2139628]	1007.8	1129.9	**1.13**	0.009368
Pitrm1	pitrilysin metallepetidase 1 [Source:MGI Symbol;Acc:MGI:1916867]	615.5	697.7	**1.13**	0.03413
Agmo	alkylglycerol monooxygenase [Source:MGI Symbol;Acc:MGI:2442495]	1053.1	1194.9	**1.13**	0.04952
Dync1i2	dynein cytoplasmic 1 intermediate chain 2 [Source:MGI Symbol;Acc:MGI:107750]	758.8	839.2	**1.13**	0.009103
Dld	dihydrolipoamide dehydrogenase [Source:MGI Symbol;Acc:MGI:107450]	2438.7	2703.6	**1.13**	0.01194
Apmap	adipocyte plasma membrane associated protein [Source:MGI Symbol;Acc:MGI:1919131]	587.7	645.9	**1.13**	0.0149
Pex3	peroxisomal biogenesis factor 3 [Source:MGI Symbol;Acc:MGI:1929646]	566.4	637.2	**1.13**	0.0245
Ap1ar	adaptor-related protein complex 1 associated regulatory protein [Source:MGI Symbol;Acc:MGI:2384822]	381.3	430.1	**1.13**	0.03655
Immt	inner membrane protein, mitochondrial [Source:MGI Symbol;Acc:MGI:1923864]	1870.5	2078.9	**1.12**	0.009185
Aggf1	angiogenic factor with G patch and FHA domains 1 [Source:MGI Symbol;Acc:MGI:1913799]	542.5	612.4	**1.12**	0.02508
Glod4	glyoxalase domain containing 4 [Source:MGI Symbol;Acc:MGI:1914451]	704.1	783.4	**1.12**	0.02516
Tprgl	transformation related protein 63 regulated like [Source:MGI Symbol;Acc:MGI:1915058]	909.5	1008.3	**1.12**	0.03903
Gfm1	G elongation factor, mitochondrial 1 [Source:MGI Symbol;Acc:MGI:107339]	1360.5	1510.2	**1.12**	0.04863
Adss	adenylosuccinate synthetase, non muscle [Source:MGI Symbol;Acc:MGI:87948]	559.7	623.8	**1.11**	0.01267
Bsdc1	BSD domain containing 1 [Source:MGI Symbol;Acc:MGI:1913466]	955.3	1049.4	**1.11**	0.01796
Vps36	vacuolar protein sorting 36 [Source:MGI Symbol;Acc:MGI:1917410]	516.8	571.7	**1.11**	0.01995
Pafah1b1	platelet-activating factor acetylhydrolase, isoform 1b, subunit 1 [Source:MGI Symbol;Acc:MGI:109520]	1401.3	1544.1	**1.11**	0.02634
Vapb	vesicle-associated membrane protein, associated protein B and C [Source:MGI Symbol;Acc:MGI:1928744]	1612.7	1767.3	**1.10**	0.006306
Trap1	TNF receptor-associated protein 1 [Source:MGI Symbol;Acc:MGI:1915265]	2926.4	3189.1	**1.10**	0.007534

Vma21	VMA21 vacuolar H+-ATPase homolog (S. cerevisiae) [Source:MGI Symbol;Acc:MGI:1914298]	488.6	537.1	**1.09**	0.04471
Itfg1	integrin alpha FG-GAP repeat containing 1 [Source:MGI Symbol;Acc:MGI:106419]	1061.5	1130.1	**1.09**	0.03306
Riok3	RIO kinase 3 [Source:MGI Symbol;Acc:MGI:1914128]	1433.7	1337.4	**-1.08**	0.0184
Cops2	COP9 signalosome subunit 2 [Source:MGI Symbol;Acc:MGI:1330276]	1322.9	1216.2	**-1.08**	0.04044
Etf1	eukaryotic translation termination factor 1 [Source:MGI Symbol;Acc:MGI:2385071]	1733.9	1564.6	**-1.09**	0.007048
Golph3	golgi phosphoprotein 3 [Source:MGI Symbol;Acc:MGI:1913879]	1356.0	1241.5	**-1.09**	0.01561
Mbd2	methyl-CpG binding domain protein 2 [Source:MGI Symbol;Acc:MGI:1333813]	869.4	790.0	**-1.09**	0.04112
Srsf11	serine/arginine-rich splicing factor 11 [Source:MGI Symbol;Acc:MGI:1916457]	581.4	562.3	**-1.12**	0.0355
Mfn1	mitofusin 1 [Source:MGI Symbol;Acc:MGI:1914664]	1031.9	935.4	**-1.12**	0.04883
Desi1	desumoylating isopeptidase 1 [Source:MGI Symbol;Acc:MGI:106313]	739.5	667.1	**-1.13**	0.008246
Gsk3a	glycogen synthase kinase 3 alpha [Source:MGI Symbol;Acc:MGI:2152453]	901.4	812.0	**-1.13**	0.01252
Amfr	autocrine motility factor receptor [Source:MGI Symbol;Acc:MGI:1345634]	6046.2	5250.9	**-1.13**	5.49E-06
Fxr1	fragile X mental retardation gene 1, autosomal homolog [Source:MGI Symbol;Acc:MGI:104860]	1006.9	884.1	**-1.13**	0.00361
Serinc3	serine incorporator 3 [Source:MGI Symbol;Acc:MGI:1349457]	3748.3	3316.0	**-1.13**	0.005493
Pcmt1	protein-L-isoaspartate (D-aspartate) O-methyltransferase 1 [Source:MGI Symbol;Acc:MGI:97502]	889.0	782.1	**-1.13**	0.006016
Dnajc7	DnaJ heat shock protein family (Hsp40) member C7 [Source:MGI Symbol;Acc:MGI:1928373]	781.5	681.1	**-1.13**	0.02811
Pias2	protein inhibitor of activated STAT 2 [Source:MGI Symbol;Acc:MGI:1096566]	542.5	474.6	**-1.13**	0.03654
Acin1	apoptotic chromatin condensation inducer 1 [Source:MGI Symbol;Acc:MGI:1891824]	725.3	682.5	**-1.13**	0.04237
Zmym5	zinc finger, MYM-type 5 [Source:MGI Symbol;Acc:MGI:3041170]	509.4	459.5	**-1.13**	0.04536
Uckl1	uridine-cytidine kinase 1-like 1 [Source:MGI Symbol;Acc:MGI:1915806]	248.0	228.1	**-1.13**	0.04876
Ncl	nucleolin [Source:MGI Symbol;Acc:MGI:97286]	3580.2	3135.0	**-1.14**	0.003037
Itgb1	integrin beta 1 (fibronectin receptor beta) [Source:MGI Symbol;Acc:MGI:96610]	3140.1	2684.1	**-1.14**	0.005268
Oxa1l	oxidase assembly 1-like [Source:MGI Symbol;Acc:MGI:1916339]	881.3	773.2	**-1.14**	0.006313
Ybx3	Y box protein 3 [Source:MGI Symbol;Acc:MGI:2137670]	460.3	402.5	**-1.14**	0.03129
Prr14	proline rich 14 [Source:MGI Symbol;Acc:MGI:2384565]	432.1	392.0	**-1.14**	0.04396
Ddx5	DEAD (Asp-Glu-Ala-Asp) box polypeptide 5 [Source:MGI Symbol;Acc:MGI:105037]	4044.1	3686.3	**-1.14**	0.0491
Slc6a13	solute carrier family 6 (neurotransmitter transporter, GABA), member 13 [Source:MGI Symbol;Acc:MGI:95629]	2699.0	2363.2	**-1.15**	0.0005451
Tial1	Tia1 cytotoxic granule-associated RNA binding protein-like 1 [Source:MGI Symbol;Acc:MGI:107913]	693.1	624.0	**-1.15**	0.00204
Pskh1	protein serine kinase H1 [Source:MGI Symbol;Acc:MGI:3528383]	694.3	613.7	**-1.15**	0.005643
Stat6	signal transducer and activator of transcription 6 [Source:MGI Symbol;Acc:MGI:103034]	1059.1	928.5	**-1.15**	0.01207
Wbp1l	WW domain binding protein 1 like [Source:MGI Symbol;Acc:MGI:107577]	5116.3	4423.4	**-1.15**	0.01271
Arih2	ariadne RBR E3 ubiquitin protein ligase 2 [Source:MGI Symbol;Acc:MGI:1344361]	718.4	629.5	**-1.15**	0.01811

Son	Son DNA binding protein [Source:MGI Symbol;Acc:MGI:98353]	2007.0	1800.8	**-1.15**	0.03108
Tlk2	tousled-like kinase 2 (Arabidopsis) [Source:MGI Symbol;Acc:MGI:1346023]	369.9	323.8	**-1.15**	0.03281
Snap29	synaptosomal-associated protein 29 [Source:MGI Symbol;Acc:MGI:1914724]	580.9	500.2	**-1.16**	0.002618
Fxr2	fragile X mental retardation, autosomal homolog 2 [Source:MGI Symbol;Acc:MGI:1346074]	633.7	559.2	**-1.16**	0.006519
Wbp11	WW domain binding protein 11 [Source:MGI Symbol;Acc:MGI:1891823]	525.7	466.6	**-1.16**	0.007319
Ppp1r15b	protein phosphatase 1, regulatory subunit 15B [Source:MGI Symbol;Acc:MGI:2444211]	1977.2	1722.2	**-1.16**	0.01228
Taok2	TAO kinase 2 [Source:MGI Symbol;Acc:MGI:1915919]	566.5	513.2	**-1.16**	0.03092
Zrsr1	zinc finger (CCCH type), RNA binding motif and serine/arginine rich 1 [Source:MGI Symbol;Acc:MGI:98885]	336.6	296.2	**-1.16**	0.03247
Akt1	thymoma viral proto-oncogene 1 [Source:MGI Symbol;Acc:MGI:87986]	950.9	831.6	**-1.16**	0.001706
Zfp110	zinc finger protein 110 [Source:MGI Symbol;Acc:MGI:1890378]	485.5	416.2	**-1.16**	0.00183
Dusp11	dual specificity phosphatase 11 (RNA/RNP complex 1-interacting) [Source:MGI Symbol;Acc:MGI:1919352]	627.8	565.3	**-1.16**	0.002782
F12	coagulation factor XII (Hageman factor) [Source:MGI Symbol;Acc:MGI:1891012]	9307.2	7903.2	**-1.16**	0.01158
Plg	plasminogen [Source:MGI Symbol;Acc:MGI:97620]	45242.1	38734.6	**-1.16**	0.01671
Sf1	splicing factor 1 [Source:MGI Symbol;Acc:MGI:1095403]	1253.8	1098.5	**-1.16**	0.01951
Sfmbt1	Scm-like with four mbt domains 1 [Source:MGI Symbol;Acc:MGI:1859609]	579.5	492.3	**-1.16**	0.0285
Git2	GIT ArfGAP 2 [Source:MGI Symbol;Acc:MGI:1347053]	189.3	168.6	**-1.16**	0.0291
Usp3	ubiquitin specific peptidase 3 [Source:MGI Symbol;Acc:MGI:2152450]	475.6	406.7	**-1.16**	0.02991
Fiz1	Flt3 interacting zinc finger protein 1 [Source:MGI Symbol;Acc:MGI:1344336]	270.5	236.7	**-1.16**	0.04561
Sf3b1	splicing factor 3b, subunit 1 [Source:MGI Symbol;Acc:MGI:1932339]	1577.3	1405.6	**-1.16**	0.04683
Zc3h14	zinc finger CCCH type containing 14 [Source:MGI Symbol;Acc:MGI:1919824]	822.9	701.5	**-1.17**	0.002234
Hbs1l	Hbs1-like (S. cerevisiae) [Source:MGI Symbol;Acc:MGI:1891704]	1028.1	887.0	**-1.17**	0.003588
Ctdsp2	CTD (carboxy-terminal domain, RNA polymerase II, polypeptide A) small phosphatase 2 [Source:MGI Symbol;Acc:MGI:1098748]	843.7	708.5	**-1.17**	0.004299
Idh3a	isocitrate dehydrogenase 3 (NAD+) alpha [Source:MGI Symbol;Acc:MGI:1915084]	570.3	496.0	**-1.17**	0.005283
Kdm5a	lysine (K)-specific demethylase 5A [Source:MGI Symbol;Acc:MGI:2136980]	525.5	448.0	**-1.17**	0.005559
Kat2b	K(lysine) acetyltransferase 2B [Source:MGI Symbol;Acc:MGI:1343094]	1859.1	1584.7	**-1.17**	0.03019
Ric8b	RIC8 guanine nucleotide exchange factor B [Source:MGI Symbol;Acc:MGI:2682307]	152.9	131.5	**-1.17**	0.04254
Gpld1	glycosylphosphatidylinositol specific phospholipase D1 [Source:MGI Symbol;Acc:MGI:106604]	4409.4	3764.8	**-1.17**	0.04331
Arl6ip6	ADP-ribosylation factor-like 6 interacting protein 6 [Source:MGI Symbol;Acc:MGI:1929507]	166.8	140.0	**-1.17**	0.04786
Tm9sf2	transmembrane 9 superfamily member 2 [Source:MGI Symbol;Acc:MGI:1915309]	2625.7	2195.9	**-1.18**	2.23E-05
Dpyd	dihydropyrimidine dehydrogenase [Source:MGI Symbol;Acc:MGI:2139667]	9510.8	7947.4	**-1.18**	0.005493

Stag2	stromal antigen 2 [Source:MGI Symbol;Acc:MGI:1098583]	1188.6	986.6	**-1.18**	0.009103
Serpinf1	serine (or cysteine) peptidase inhibitor, clade F, member 1 [Source:MGI Symbol;Acc:MGI:108080]	10494.7	8799.1	**-1.18**	0.009108
Tfr2	transferrin receptor 2 [Source:MGI Symbol;Acc:MGI:1354956]	6381.2	5438.8	**-1.18**	0.009164
Pxk	PX domain containing serine/threonine kinase [Source:MGI Symbol;Acc:MGI:1289230]	357.4	308.4	**-1.18**	0.01196
Larp1	La ribonucleoprotein domain family, member 1 [Source:MGI Symbol;Acc:MGI:1890165]	2616.4	2192.6	**-1.18**	0.01311
Cdk8	cyclin-dependent kinase 8 [Source:MGI Symbol;Acc:MGI:1196224]	417.6	361.0	**-1.18**	0.01636
Alb	albumin [Source:MGI Symbol;Acc:MGI:87991]	1450451.6	1166955.2	**-1.18**	0.02508
Sart3	squamous cell carcinoma antigen recognized by T cells 3 [Source:MGI Symbol;Acc:MGI:1858230]	224.9	193.2	**-1.18**	0.02582
Aass	aminoadipate-semialdehyde synthase [Source:MGI Symbol;Acc:MGI:1353573]	5085.3	4361.6	**-1.18**	0.0265
Trmt1	tRNA methyltransferase 1 [Source:MGI Symbol;Acc:MGI:1289155]	459.2	389.6	**-1.18**	0.02772
Kdm3a	lysine (K)-specific demethylase 3A [Source:MGI Symbol;Acc:MGI:98847]	214.7	181.2	**-1.18**	0.02857
Polr1b	polymerase (RNA) I polypeptide B [Source:MGI Symbol;Acc:MGI:108014]	229.3	200.6	**-1.18**	0.03376
mt-Nd2	mitochondrially encoded NADH dehydrogenase 2 [Source:MGI Symbol;Acc:MGI:102500]	132563.8	112666.5	**-1.18**	0.03594
Edc4	enhancer of mRNA decapping 4 [Source:MGI Symbol;Acc:MGI:2446249]	227.0	204.5	**-1.18**	0.03655
Ncstn	nicastrin [Source:MGI Symbol;Acc:MGI:1891700]	911.8	751.4	**-1.19**	0.0007861
Tshz1	teashirt zinc finger family member 1 [Source:MGI Symbol;Acc:MGI:1346031]	385.7	332.0	**-1.19**	0.00387
Ercc5	excision repair cross-complementing rodent repair deficiency, complementation group 5 [Source:MGI Symbol;Acc:MGI:103582]	259.5	221.0	**-1.19**	0.005267
Paics	phosphoribosylaminoimidazole carboxylase, phosphoribosylaminoribosylaminoimidazole, succinocarboxamide synthetase [Source:MGI Symbol;Acc:MGI:1914304]	3686.3	3138.9	**-1.19**	0.006263
Atxn7l3	ataxin 7-like 3 [Source:MGI Symbol;Acc:MGI:3036270]	571.1	483.3	**-1.19**	0.006696
Znrf1	zinc and ring finger 1 [Source:MGI Symbol;Acc:MGI:2177308]	434.6	397.1	**-1.19**	0.007656
Soat2	sterol O-acyltransferase 2 [Source:MGI Symbol;Acc:MGI:1332226]	671.3	559.3	**-1.19**	0.01077
Med17	mediator complex subunit 17 [Source:MGI Symbol;Acc:MGI:2182585]	197.7	166.1	**-1.19**	0.01093
Eif3a	eukaryotic translation initiation factor 3, subunit A [Source:MGI Symbol;Acc:MGI:95301]	2818.8	2340.9	**-1.19**	0.01149
Zhx2	zinc fingers and homeoboxes 2 [Source:MGI Symbol;Acc:MGI:2683087]	276.1	237.3	**-1.19**	0.01485
Gmps	guanine monophosphate synthetase [Source:MGI Symbol;Acc:MGI:2448526]	707.7	598.5	**-1.19**	0.02186
Dtnb	dystrobrevin, beta [Source:MGI Symbol;Acc:MGI:1203728]	383.7	326.5	**-1.19**	0.02372
Socs7	suppressor of cytokine signaling 7 [Source:MGI Symbol;Acc:MGI:2651588]	327.9	274.2	**-1.19**	0.02582
Ripk2	receptor (TNFRSF)-interacting serine-threonine kinase 2 [Source:MGI Symbol;Acc:MGI:1891456]	174.5	152.2	**-1.19**	0.03419
Clk2	CDC-like kinase 2 [Source:MGI Symbol;Acc:MGI:1098669]	130.9	118.8	**-1.19**	0.03621
Pbxip1	pre B cell leukemia transcription factor interacting protein 1 [Source:MGI Symbol;Acc:MGI:2441670]	249.7	204.3	**-1.19**	0.03655
Ces2a	carboxylesterase 2A [Source:MGI Symbol;Acc:MGI:2142491]	2500.0	2052.4	**-1.19**	0.04105

Ptcd1	pentatricopeptide repeat domain 1 [Source:MGI Symbol;Acc:MGI:1919049]	188.6	158.6	**-1.19**	0.04254
Atrx	ATRX, chromatin remodeler [Source:MGI Symbol;Acc:MGI:103067]	756.7	640.4	**-1.19**	0.0479
Ndel1	nudE neurodevelopment protein 1 like 1 [Source:MGI Symbol;Acc:MGI:1932915]	543.1	462.0	**-1.19**	0.04876
Pabpc1	poly(A) binding protein, cytoplasmic 1 [Source:MGI Symbol;Acc:MGI:1349722]	6055.7	5058.9	**-1.20**	4.33E-05
Trim28	tripartite motif-containing 28 [Source:MGI Symbol;Acc:MGI:109274]	1067.0	902.4	**-1.20**	4.80E-05
Zfyve27	zinc finger, FYVE domain containing 27 [Source:MGI Symbol;Acc:MGI:1919602]	284.2	243.1	**-1.20**	0.001671
Clk3	CDC-like kinase 3 [Source:MGI Symbol;Acc:MGI:1098670]	412.0	358.9	**-1.20**	0.001751
Cd47	CD47 antigen (Rh-related antigen, integrin-associated signal transducer) [Source:MGI Symbol;Acc:MGI:96617]	1513.7	1224.5	**-1.20**	0.001784
Zbtb7a	zinc finger and BTB domain containing 7a [Source:MGI Symbol;Acc:MGI:1335091]	918.4	768.5	**-1.20**	0.00278
Scarb2	scavenger receptor class B, member 2 [Source:MGI Symbol;Acc:MGI:1196458]	3542.9	2994.8	**-1.20**	0.006657
Itih2	inter-alpha trypsin inhibitor, heavy chain 2 [Source:MGI Symbol;Acc:MGI:96619]	17002.7	13960.0	**-1.20**	0.01796
Rabggtb	Rab geranylgeranyl transferase, b subunit [Source:MGI Symbol;Acc:MGI:99537]	767.6	630.8	**-1.20**	0.01807
Akap8l	A kinase (PRKA) anchor protein 8-like [Source:MGI Symbol;Acc:MGI:1860606]	207.6	190.6	**-1.20**	0.01822
Edrf1	erythroid differentiation regulatory factor 1 [Source:MGI Symbol;Acc:MGI:1919831]	304.8	261.0	**-1.20**	0.02428
Naglu	alpha-N-acetylglucosaminidase (Sanfilippo disease IIIB) [Source:MGI Symbol;Acc:MGI:1351641]	539.1	441.3	**-1.20**	0.03107
Zfp384	zinc finger protein 384 [Source:MGI Symbol;Acc:MGI:2443203]	273.9	235.3	**-1.20**	0.03312
Rgl1	ral guanine nucleotide dissociation stimulator,-like 1 [Source:MGI Symbol;Acc:MGI:107484]	247.6	200.2	**-1.20**	0.04472
Mecp2	methyl CpG binding protein 2 [Source:MGI Symbol;Acc:MGI:99918]	325.9	272.6	**-1.20**	0.04786
Rexo4	REX4, 3'-5' exonuclease [Source:MGI Symbol;Acc:MGI:2684957]	259.4	223.6	**-1.21**	0.001422
Sdc2	syndecan 2 [Source:MGI Symbol;Acc:MGI:1349165]	3208.9	2654.0	**-1.21**	0.001971
Plod3	procollagen-lysine, 2-oxoglutarate 5-dioxygenase 3 [Source:MGI Symbol;Acc:MGI:1347008]	394.6	327.9	**-1.21**	0.001977
Nfx1	nuclear transcription factor, X-box binding 1 [Source:MGI Symbol;Acc:MGI:1921414]	871.9	740.2	**-1.21**	0.002595
Srsf4	serine/arginine-rich splicing factor 4 [Source:MGI Symbol;Acc:MGI:1890577]	657.7	572.9	**-1.21**	0.002601
Slc17a2	solute carrier family 17 (sodium phosphate), member 2 [Source:MGI Symbol;Acc:MGI:2443098]	2687.6	2277.0	**-1.21**	0.005869
Rgl2	ral guanine nucleotide dissociation stimulator-like 2 [Source:MGI Symbol;Acc:MGI:107483]	181.8	162.1	**-1.21**	0.005869
Mark4	MAP/microtubule affinity regulating kinase 4 [Source:MGI Symbol;Acc:MGI:1920955]	300.5	254.2	**-1.21**	0.006988
Sidt2	SID1 transmembrane family, member 2 [Source:MGI Symbol;Acc:MGI:2446134]	2513.2	2112.6	**-1.21**	0.007931
Mepce	methylphosphate capping enzyme [Source:MGI Symbol;Acc:MGI:106477]	465.5	397.9	**-1.21**	0.009103
Manba	mannosidase, beta A, lysosomal [Source:MGI Symbol;Acc:MGI:88175]	309.5	253.6	**-1.21**	0.009756
Prpf38b	PRP38 pre-mRNA processing factor 38 (yeast) domain containing B [Source:MGI Symbol;Acc:MGI:1914171]	411.4	369.1	**-1.21**	0.01228
Ahsg	alpha-2-HS-glycoprotein [Source:MGI Symbol;Acc:MGI:107189]	115534.1	97320.3	**-1.21**	0.01477

N4bp1	NEDD4 binding protein 1 [Source:MGI Symbol;Acc:MGI:2136825]	305.3	254.3	**-1.21**	0.01611
B4galt3	UDP-Gal:betaGlcNAc beta 1,4-galactosyltransferase, polypeptide 3 [Source:MGI Symbol;Acc:MGI:1928767]	155.8	130.4	**-1.21**	0.01727
Gpat4	glycerol-3-phosphate acyltransferase 4 [Source:MGI Symbol;Acc:MGI:2142716]	3744.2	3160.5	**-1.21**	0.01858
Abhd17b	abhydrolase domain containing 17B [Source:MGI Symbol;Acc:MGI:1917816]	435.7	357.6	**-1.21**	0.02605
Tcirg1	T cell, immune regulator 1, ATPase, H+ transporting, lysosomal V0 protein A3 [Source:MGI Symbol;Acc:MGI:1350931]	401.8	341.3	**-1.21**	0.02682
Mllt10	myeloid/lymphoid or mixed-lineage leukemia; translocated to, 10 [Source:MGI Symbol;Acc:MGI:1329038]	372.8	311.9	**-1.21**	0.03736
Hnrnpdl	heterogeneous nuclear ribonucleoprotein D-like [Source:MGI Symbol;Acc:MGI:1355299]	725.7	609.7	**-1.21**	0.001067
Brd1	bromodomain containing 1 [Source:MGI Symbol;Acc:MGI:1924161]	421.6	351.8	**-1.21**	0.001814
Cpsf7	cleavage and polyadenylation specific factor 7 [Source:MGI Symbol;Acc:MGI:1917826]	295.4	258.4	**-1.21**	0.002405
Rrn3	RRN3 RNA polymerase I transcription factor homolog (yeast) [Source:MGI Symbol;Acc:MGI:1925255]	327.8	276.5	**-1.21**	0.003396
Igf1	insulin-like growth factor 1 [Source:MGI Symbol;Acc:MGI:96432]	9830.7	8196.7	**-1.21**	0.003414
Ddx21	DEAD (Asp-Glu-Ala-Asp) box polypeptide 21 [Source:MGI Symbol;Acc:MGI:1860494]	662.7	539.2	**-1.21**	0.00458
Gnl3	guanine nucleotide binding protein-like 3 (nucleolar) [Source:MGI Symbol;Acc:MGI:1353651]	462.0	386.8	**-1.21**	0.006914
Pnisr	PNN interacting serine/arginine-rich [Source:MGI Symbol;Acc:MGI:1913875]	319.8	300.3	**-1.21**	0.006952
Plekhg3	pleckstrin homology domain containing, family G (with RhoGef domain) member 3 [Source:MGI Symbol;Acc:MGI:2388284]	637.0	540.0	**-1.21**	0.009339
Tns2	tensin 2 [Source:MGI Symbol;Acc:MGI:2387586]	1329.5	1103.5	**-1.21**	0.01072
Gpc4	glypican 4 [Source:MGI Symbol;Acc:MGI:104902]	705.4	577.8	**-1.21**	0.01237
Cnot3	CCR4-NOT transcription complex, subunit 3 [Source:MGI Symbol;Acc:MGI:2385261]	232.4	194.3	**-1.21**	0.01627
Serpina1d	serine (or cysteine) peptidase inhibitor, clade A, member 1D [Source:MGI Symbol;Acc:MGI:891968]	42169.2	34557.8	**-1.21**	0.01856
Slc25a22	solute carrier family 25 (mitochondrial carrier, glutamate), member 22 [Source:MGI Symbol;Acc:MGI:1915517]	3070.0	2563.3	**-1.21**	0.01953
Phrf1	PHD and ring finger domains 1 [Source:MGI Symbol;Acc:MGI:2141847]	300.6	254.5	**-1.21**	0.02809
Cnp	2',3'-cyclic nucleotide 3' phosphodiesterase [Source:MGI Symbol;Acc:MGI:88437]	283.0	224.7	**-1.21**	0.03102
Pnpla6	patatin-like phospholipase domain containing 6 [Source:MGI Symbol;Acc:MGI:1354723]	214.3	185.9	**-1.21**	0.03103
Rsbn1	rosbin, round spermatid basic protein 1 [Source:MGI Symbol;Acc:MGI:2444993]	140.1	114.6	**-1.21**	0.03822
Gsdmd	gasdermin D [Source:MGI Symbol;Acc:MGI:1916396]	530.6	441.7	**-1.21**	0.0398
Rdh13	retinol dehydrogenase 13 (all-trans and 9-cis) [Source:MGI Symbol;Acc:MGI:1918732]	146.8	122.9	**-1.21**	0.0424
Zfp281	zinc finger protein 281 [Source:MGI Symbol;Acc:MGI:3029290]	417.2	345.2	**-1.21**	0.04396
Tnks1bp1	tankyrase 1 binding protein 1 [Source:MGI Symbol;Acc:MGI:2446193]	912.5	761.2	**-1.21**	0.04928
Pcbp2	poly(rC) binding protein 2 [Source:MGI Symbol;Acc:MGI:108202]	4017.5	3259.6	**-1.22**	1.62E-05

Ergic2	ERGIC and golgi 2 [Source:MGI Symbol;Acc:MGI:1914706]	850.1	707.6	**-1.22**	6.03E-05
Hnrnpa1	heterogeneous nuclear ribonucleoprotein A1 [Source:MGI Symbol;Acc:MGI:104820]	591.1	485.6	**-1.22**	6.68E-05
Cry2	cryptochrome 2 (photolyase-like) [Source:MGI Symbol;Acc:MGI:1270859]	736.5	609.9	**-1.22**	0.0006038
Zdhhc20	zinc finger, DHHC domain containing 20 [Source:MGI Symbol;Acc:MGI:1923215]	299.6	234.7	**-1.22**	0.002307
Scarb1	scavenger receptor class B, member 1 [Source:MGI Symbol;Acc:MGI:893578]	2969.1	2425.6	**-1.22**	0.00373
Clk4	CDC like kinase 4 [Source:MGI Symbol;Acc:MGI:1098551]	285.9	259.5	**-1.22**	0.004307
Hgfac	hepatocyte growth factor activator [Source:MGI Symbol;Acc:MGI:1859281]	4690.6	3816.2	**-1.22**	0.005236
Grn	granulin [Source:MGI Symbol;Acc:MGI:95832]	2965.0	2401.6	**-1.22**	0.01023
Ilf3	interleukin enhancer binding factor 3 [Source:MGI Symbol;Acc:MGI:1339973]	242.5	202.1	**-1.22**	0.01084
Kdm4b	lysine (K)-specific demethylase 4B [Source:MGI Symbol;Acc:MGI:2442355]	239.7	199.1	**-1.22**	0.01176
Ogdh	oxoglutarate (alpha-ketoglutarate) dehydrogenase (lipoamide) [Source:MGI Symbol;Acc:MGI:1098267]	3397.6	2799.4	**-1.22**	0.01232
Adck2	aarF domain containing kinase 2 [Source:MGI Symbol;Acc:MGI:1889336]	140.4	117.0	**-1.22**	0.01311
Zmym2	zinc finger, MYM-type 2 [Source:MGI Symbol;Acc:MGI:1923257]	377.9	312.8	**-1.22**	0.01659
Pan2	PAN2 poly(A) specific ribonuclease subunit [Source:MGI Symbol;Acc:MGI:1918984]	326.0	278.6	**-1.22**	0.02428
Trim39	tripartite motif-containing 39 [Source:MGI Symbol;Acc:MGI:1890659]	127.5	113.5	**-1.22**	0.02809
Cyp2c68	cytochrome P450, family 2, subfamily c, polypeptide 68 [Source:MGI Symbol;Acc:MGI:3612287]	1900.2	1514.6	**-1.22**	0.03766
Cirbp	cold inducible RNA binding protein [Source:MGI Symbol;Acc:MGI:893588]	376.6	289.6	**-1.22**	0.04547
Eif4b	eukaryotic translation initiation factor 4B [Source:MGI Symbol;Acc:MGI:95304]	6182.9	5013.6	**-1.23**	1.55E-07
Safb	scaffold attachment factor B [Source:MGI Symbol;Acc:MGI:2146974]	550.2	485.4	**-1.23**	0.0004571
Khdc4	KH domain containing 4, pre-mRNA splicing factor [Source:MGI Symbol;Acc:MGI:1921450]	390.1	337.9	**-1.23**	0.0005595
Gaa	glucosidase, alpha, acid [Source:MGI Symbol;Acc:MGI:95609]	1705.5	1369.5	**-1.23**	0.0006117
Btd	biotinidase [Source:MGI Symbol;Acc:MGI:1347001]	1180.9	929.2	**-1.23**	0.0006127
Safb2	scaffold attachment factor B2 [Source:MGI Symbol;Acc:MGI:2146808]	506.1	448.4	**-1.23**	0.001102
F10	coagulation factor X [Source:MGI Symbol;Acc:MGI:103107]	8526.7	6862.8	**-1.23**	0.001212
Cflar	CASP8 and FADD-like apoptosis regulator [Source:MGI Symbol;Acc:MGI:1336166]	1279.2	1037.7	**-1.23**	0.006578
Ccnl1	cyclin L1 [Source:MGI Symbol;Acc:MGI:1922664]	228.8	204.1	**-1.23**	0.007415
Ctsf	cathepsin F [Source:MGI Symbol;Acc:MGI:1861434]	482.3	392.1	**-1.23**	0.007713
Ap4m1	adaptor-related protein complex AP-4, mu 1 [Source:MGI Symbol;Acc:MGI:1337063]	288.0	234.9	**-1.23**	0.00917
Klhl3	kelch-like 3 [Source:MGI Symbol;Acc:MGI:2445185]	137.8	111.6	**-1.23**	0.02274
Zcchc7	zinc finger, CCHC domain containing 7 [Source:MGI Symbol;Acc:MGI:2442912]	127.0	106.4	**-1.23**	0.03051
Cd38	CD38 antigen [Source:MGI Symbol;Acc:MGI:107474]	167.7	132.1	**-1.23**	0.03344
Usp36	ubiquitin specific peptidase 36 [Source:MGI Symbol;Acc:MGI:1919594]	293.9	253.7	**-1.23**	0.03344

Tsc22d4	TSC22 domain family, member 4 [Source:MGI Symbol;Acc:MGI:1926079]	312.1	235.9	**-1.23**	0.0355
Zcchc2	zinc finger, CCHC domain containing 2 [Source:MGI Symbol;Acc:MGI:2444114]	451.5	372.9	**-1.23**	0.03766
Rusc2	RUN and SH3 domain containing 2 [Source:MGI Symbol;Acc:MGI:2140371]	216.7	184.9	**-1.23**	0.04368
Baz1a	bromodomain adjacent to zinc finger domain 1A [Source:MGI Symbol;Acc:MGI:1309478]	130.6	107.5	**-1.23**	0.04465
Ankrd10	ankyrin repeat domain 10 [Source:MGI Symbol;Acc:MGI:1921840]	69.6	59.4	**-1.23**	0.04507
Brap	BRCA1 associated protein [Source:MGI Symbol;Acc:MGI:1919649]	3167.8	2553.1	**-1.24**	9.09E-08
Eef2	eukaryotic translation elongation factor 2 [Source:MGI Symbol;Acc:MGI:95288]	29911.1	23618.9	**-1.24**	9.75E-07
Naga	N-acetyl galactosaminidase, alpha [Source:MGI Symbol;Acc:MGI:1261422]	601.6	481.4	**-1.24**	8.92E-05
Baiap2l1	BAI1-associated protein 2-like 1 [Source:MGI Symbol;Acc:MGI:1914148]	604.0	486.5	**-1.24**	0.001971
Bmp1	bone morphogenetic protein 1 [Source:MGI Symbol;Acc:MGI:88176]	1884.9	1531.0	**-1.24**	0.007048
Taf4	TATA-box binding protein associated factor 4 [Source:MGI Symbol;Acc:MGI:2152346]	231.5	187.8	**-1.24**	0.01023
Luc7l2	LUC7-like 2 (S. cerevisiae) [Source:MGI Symbol;Acc:MGI:2183260]	972.1	828.9	**-1.24**	0.01137
Bmp6	bone morphogenetic protein 6 [Source:MGI Symbol;Acc:MGI:88182]	99.8	75.2	**-1.24**	0.01747
Ric1	RAB6A GEF complex partner 1 [Source:MGI Symbol;Acc:MGI:1924893]	575.3	464.4	**-1.24**	0.01821
Gne	glucosamine (UDP-N-acetyl)-2-epimerase/N-acetylmannosamine kinase [Source:MGI Symbol;Acc:MGI:1354951]	2591.9	2199.8	**-1.24**	0.02734
Itih3	inter-alpha trypsin inhibitor, heavy chain 3 [Source:MGI Symbol;Acc:MGI:96620]	10064.0	8113.5	**-1.24**	0.03113
Aspg	asparaginase [Source:MGI Symbol;Acc:MGI:2144822]	3472.2	2868.6	**-1.25**	0.0002256
Akap8	A kinase (PRKA) anchor protein 8 [Source:MGI Symbol;Acc:MGI:1928488]	421.2	358.8	**-1.25**	0.000814
Arid4b	AT rich interactive domain 4B (RBP1-like) [Source:MGI Symbol;Acc:MGI:2137512]	308.0	259.4	**-1.25**	0.001303
S100pbp	S100P binding protein [Source:MGI Symbol;Acc:MGI:1921898]	271.6	219.0	**-1.25**	0.001602
Brpf1	bromodomain and PHD finger containing, 1 [Source:MGI Symbol;Acc:MGI:1926033]	215.3	180.7	**-1.25**	0.002023
Crebbp	CREB binding protein [Source:MGI Symbol;Acc:MGI:1098280]	315.7	259.3	**-1.25**	0.00204
Dcaf6	DDB1 and CUL4 associated factor 6 [Source:MGI Symbol;Acc:MGI:1921356]	734.1	593.9	**-1.25**	0.005869
Ttc17	tetratricopeptide repeat domain 17 [Source:MGI Symbol;Acc:MGI:1921819]	516.7	407.1	**-1.25**	0.009715
Tbpl1	TATA box binding protein-like 1 [Source:MGI Symbol;Acc:MGI:1339946]	105.8	87.9	**-1.25**	0.0155
Cyp4f14	cytochrome P450, family 4, subfamily f, polypeptide 14 [Source:MGI Symbol;Acc:MGI:1927669]	4451.0	3629.9	**-1.25**	0.01899
Atxn7	ataxin 7 [Source:MGI Symbol;Acc:MGI:2179277]	370.7	299.0	**-1.25**	0.02319
Ptch1	patched 1 [Source:MGI Symbol;Acc:MGI:105373]	484.0	375.2	**-1.25**	0.03244
Asap3	ArfGAP with SH3 domain, ankyrin repeat and PH domain 3 [Source:MGI Symbol;Acc:MGI:2684986]	102.3	85.1	**-1.25**	0.0345
Arl5b	ADP-ribosylation factor-like 5B [Source:MGI Symbol;Acc:MGI:1923119]	696.3	562.0	**-1.25**	0.03467
Pptc7	PTC7 protein phosphatase homolog [Source:MGI Symbol;Acc:MGI:2444593]	493.8	403.7	**-1.25**	0.03655
Tomm40l	translocase of outer mitochondrial membrane 40-like [Source:MGI Symbol;Acc:MGI:3589112]	276.2	208.3	**-1.25**	0.04102

Frrs1	ferric-chelate reductase 1 [Source:MGI Symbol;Acc:MGI:108076]	474.7	378.9	**-1.26**	3.37E-05
Chp1	calcineurin-like EF hand protein 1 [Source:MGI Symbol;Acc:MGI:1927185]	5855.5	4728.5	**-1.26**	0.001231
Vtn	vitronectin [Source:MGI Symbol;Acc:MGI:98940]	39303.3	31200.9	**-1.26**	0.005268
Cd300lg	CD300 molecule like family member G [Source:MGI Symbol;Acc:MGI:1289168]	274.6	214.8	**-1.26**	0.00817
Elmod3	ELMO/CED-12 domain containing 3 [Source:MGI Symbol;Acc:MGI:2445168]	341.4	272.1	**-1.26**	0.009592
Arfip2	ADP-ribosylation factor interacting protein 2 [Source:MGI Symbol;Acc:MGI:1924182]	155.3	129.8	**-1.26**	0.01
Pdcd11	programmed cell death 11 [Source:MGI Symbol;Acc:MGI:1341788]	325.6	272.2	**-1.26**	0.01308
Sclt1	sodium channel and clathrin linker 1 [Source:MGI Symbol;Acc:MGI:1914411]	114.5	90.3	**-1.26**	0.02922
Tgfbr3	transforming growth factor, beta receptor III [Source:MGI Symbol;Acc:MGI:104637]	157.7	122.9	**-1.26**	0.03725
Slc16a11	solute carrier family 16 (monocarboxylic acid transporters), member 11 [Source:MGI Symbol;Acc:MGI:2663709]	353.1	269.8	**-1.26**	0.04091
Scnn1a	sodium channel, nonvoltage-gated 1 alpha [Source:MGI Symbol;Acc:MGI:101782]	396.9	324.2	**-1.26**	0.04245
Dvl1	dishevelled segment polarity protein 1 [Source:MGI Symbol;Acc:MGI:94941]	899.7	740.2	**-1.27**	1.23E-07
Kng1	kininogen 1 [Source:MGI Symbol;Acc:MGI:1097705]	32524.9	25152.8	**-1.27**	4.80E-05
Kdsr	3-ketodihydrosphingosine reductase [Source:MGI Symbol;Acc:MGI:1918000]	549.4	432.1	**-1.27**	0.00119
Sltm	SAFB-like, transcription modulator [Source:MGI Symbol;Acc:MGI:1913910]	446.9	355.7	**-1.27**	0.001619
Sema4g	sema domain, immunoglobulin domain (Ig), transmembrane domain (TM) and short cytoplasmic domain, (semaphorin) 4G [Source:MGI Symbol;Acc:MGI:1347047]	4340.8	3584.2	**-1.27**	0.003128
Sirt1	sirtuin 1 [Source:MGI Symbol;Acc:MGI:2135607]	197.8	158.3	**-1.27**	0.003265
Taok3	TAO kinase 3 [Source:MGI Symbol;Acc:MGI:3041177]	1347.6	1043.3	**-1.27**	0.00371
Ldb1	LIM domain binding 1 [Source:MGI Symbol;Acc:MGI:894762]	477.1	400.3	**-1.27**	0.00373
Shf	Src homology 2 domain containing F [Source:MGI Symbol;Acc:MGI:3613669]	271.5	209.5	**-1.27**	0.004299
Fnbp4	formin binding protein 4 [Source:MGI Symbol;Acc:MGI:1860513]	175.9	154.0	**-1.27**	0.004636
Dvl3	dishevelled segment polarity protein 3 [Source:MGI Symbol;Acc:MGI:108100]	262.5	214.1	**-1.27**	0.009164
Siah2	siah E3 ubiquitin protein ligase 2 [Source:MGI Symbol;Acc:MGI:108062]	341.0	265.5	**-1.27**	0.01235
Npr1	natriuretic peptide receptor 1 [Source:MGI Symbol;Acc:MGI:97371]	181.3	144.5	**-1.27**	0.01284
St3gal1	ST3 beta-galactoside alpha-2,3-sialyltransferase 1 [Source:MGI Symbol;Acc:MGI:98304]	1267.0	973.9	**-1.27**	0.0209
Foxa3	forkhead box A3 [Source:MGI Symbol;Acc:MGI:1347477]	634.4	530.5	**-1.27**	0.03461
Amigo2	adhesion molecule with Ig like domain 2 [Source:MGI Symbol;Acc:MGI:2145995]	136.9	104.9	**-1.27**	0.03467
Cep95	centrosomal protein 95 [Source:MGI Symbol;Acc:MGI:2443502]	74.7	60.6	**-1.27**	0.03472
Mta1	metastasis associated 1 [Source:MGI Symbol;Acc:MGI:2150037]	645.0	508.2	**-1.27**	6.25E-07
Tmem63b	transmembrane protein 63b [Source:MGI Symbol;Acc:MGI:2387609]	1379.8	1093.0	**-1.27**	4.61E-06
C1ra	complement component 1, r subcomponent A [Source:MGI Symbol;Acc:MGI:1355313]	3082.7	2425.2	**-1.27**	0.0004346

Ints6	integrator complex subunit 6 [Source:MGI Symbol;Acc:MGI:1202397]	297.7	238.2	**-1.27**	0.002619
Vwce	von Willebrand factor C and EGF domains [Source:MGI Symbol;Acc:MGI:1919018]	589.9	460.6	**-1.27**	0.004413
Pkdcc	protein kinase domain containing, cytoplasmic [Source:MGI Symbol;Acc:MGI:2147077]	425.7	344.5	**-1.27**	0.00651
Slc12a4	solute carrier family 12, member 4 [Source:MGI Symbol;Acc:MGI:1309465]	313.7	246.2	**-1.27**	0.007931
Tent4a	terminal nucleotidyltransferase 4A [Source:MGI Symbol;Acc:MGI:2682295]	360.2	286.2	**-1.27**	0.01267
Cecr2	CECR2, histone acetyl-lysine reader [Source:MGI Symbol;Acc:MGI:1923799]	233.7	188.3	**-1.27**	0.01438
Arhgef15	Rho guanine nucleotide exchange factor (GEF) 15 [Source:MGI Symbol;Acc:MGI:3045246]	128.7	99.0	**-1.27**	0.02027
Adgrl2	adhesion G protein-coupled receptor L2 [Source:MGI Symbol;Acc:MGI:2139714]	783.0	610.0	**-1.27**	0.02174
Ephb4	Eph receptor B4 [Source:MGI Symbol;Acc:MGI:104757]	456.4	353.6	**-1.28**	5.55E-08
F7	coagulation factor VII [Source:MGI Symbol;Acc:MGI:109325]	1751.2	1375.5	**-1.28**	0.001976
Pan3	PAN3 poly(A) specific ribonuclease subunit [Source:MGI Symbol;Acc:MGI:1919837]	229.5	186.7	**-1.28**	0.001977
Map3k21	mitogen-activated protein kinase kinase kinase 21 [Source:MGI Symbol;Acc:MGI:2385307]	116.3	88.4	**-1.28**	0.002955
Atp1b1	ATPase, Na+/K+ transporting, beta 1 polypeptide [Source:MGI Symbol;Acc:MGI:88108]	1190.1	905.7	**-1.28**	0.004959
Mat2a	methionine adenosyltransferase II, alpha [Source:MGI Symbol;Acc:MGI:2443731]	1112.1	886.0	**-1.28**	0.004979
Tsc22d2	TSC22 domain family, member 2 [Source:MGI Symbol;Acc:MGI:1919283]	385.1	302.2	**-1.28**	0.006151
Hal	histidine ammonia lyase [Source:MGI Symbol;Acc:MGI:96010]	6753.4	5132.5	**-1.28**	0.007045
Lrrc29	leucine rich repeat containing 29 [Source:MGI Symbol;Acc:MGI:2443262]	78.5	62.0	**-1.28**	0.007823
Mrc1	mannose receptor, C type 1 [Source:MGI Symbol;Acc:MGI:97142]	723.8	540.8	**-1.28**	0.01174
Idua	iduronidase, alpha-L [Source:MGI Symbol;Acc:MGI:96418]	97.9	74.9	**-1.28**	0.01192
Slc6a6	solute carrier family 6 (neurotransmitter transporter, taurine), member 6 [Source:MGI Symbol;Acc:MGI:98488]	1767.2	1323.9	**-1.28**	0.01299
Bmp2	bone morphogenetic protein 2 [Source:MGI Symbol;Acc:MGI:88177]	319.7	243.7	**-1.28**	0.01983
Kdr	kinase insert domain protein receptor [Source:MGI Symbol;Acc:MGI:96683]	778.9	572.2	**-1.28**	0.01996
Tet3	tet methylcytosine dioxygenase 3 [Source:MGI Symbol;Acc:MGI:2446229]	328.2	256.7	**-1.28**	0.02259
Plxnb1	plexin B1 [Source:MGI Symbol;Acc:MGI:2154238]	842.1	643.5	**-1.28**	0.02399
Agbl5	ATP/GTP binding protein-like 5 [Source:MGI Symbol;Acc:MGI:2441745]	126.5	96.1	**-1.28**	0.02612
Il17rb	interleukin 17 receptor B [Source:MGI Symbol;Acc:MGI:1355292]	130.0	100.4	**-1.28**	0.03108
Myo7a	myosin VIIA [Source:MGI Symbol;Acc:MGI:104510]	170.1	129.0	**-1.28**	0.03629
Pla1a	phospholipase A1 member A [Source:MGI Symbol;Acc:MGI:1934677]	746.4	590.5	**-1.29**	0.0001715
Fgg	fibrinogen gamma chain [Source:MGI Symbol;Acc:MGI:95526]	61815.1	46299.2	**-1.29**	0.0001913
Cxcl12	chemokine (C-X-C motif) ligand 12 [Source:MGI Symbol;Acc:MGI:103556]	5823.7	4456.8	**-1.29**	0.0004672
St3gal4	ST3 beta-galactoside alpha-2,3-sialyltransferase 4 [Source:MGI Symbol;Acc:MGI:1316743]	1457.8	1143.0	**-1.29**	0.001191
Slc39a14	solute carrier family 39 (zinc transporter), member 14 [Source:MGI Symbol;Acc:MGI:2384851]	2958.7	2389.3	**-1.29**	0.002348

Ctif	CBP80/20-dependent translation initiation factor [Source:MGI Symbol;Acc:MGI:2685518]	572.1	446.9	**-1.29**	0.002705
Gcnt2	glucosaminyl (N-acetyl) transferase 2, I-branching enzyme [Source:MGI Symbol;Acc:MGI:1100870]	457.0	357.6	**-1.29**	0.002711
Sertad2	SERTA domain containing 2 [Source:MGI Symbol;Acc:MGI:1931026]	382.0	295.3	**-1.29**	0.002852
Ano8	anoctamin 8 [Source:MGI Symbol;Acc:MGI:2687327]	76.2	57.9	**-1.29**	0.01609
Cnnm4	cyclin M4 [Source:MGI Symbol;Acc:MGI:2151060]	60.0	46.9	**-1.29**	0.02546
Cyp2f2	cytochrome P450, family 2, subfamily f, polypeptide 2 [Source:MGI Symbol;Acc:MGI:88608]	13639.3	10761.2	**-1.30**	2.10E-06
Epha1	Eph receptor A1 [Source:MGI Symbol;Acc:MGI:107381]	835.6	661.2	**-1.30**	1.71E-05
Slc12a9	solute carrier family 12 (potassium/chloride transporters), member 9 [Source:MGI Symbol;Acc:MGI:1933532]	100.1	87.6	**-1.30**	0.001097
Camta2	calmodulin binding transcription activator 2 [Source:MGI Symbol;Acc:MGI:2135957]	335.2	266.0	**-1.30**	0.001377
Rassf5	Ras association (RalGDS/AF-6) domain family member 5 [Source:MGI Symbol;Acc:MGI:1926375]	260.2	196.1	**-1.30**	0.001447
Glb1	galactosidase, beta 1 [Source:MGI Symbol;Acc:MGI:88151]	244.0	183.8	**-1.30**	0.001739
Prpf40b	pre-mRNA processing factor 40B [Source:MGI Symbol;Acc:MGI:1925583]	141.9	117.3	**-1.30**	0.004409
Micall1	microtubule associated monooxygenase, calponin and LIM domain containing -like 1 [Source:MGI Symbol;Acc:MGI:105870]	247.4	196.2	**-1.30**	0.005911
C3	complement component 3 [Source:MGI Symbol;Acc:MGI:88227]	87811.9	65964.4	**-1.30**	0.008649
Dgka	diacylglycerol kinase, alpha [Source:MGI Symbol;Acc:MGI:102952]	112.2	89.8	**-1.30**	0.009164
Fbxo21	F-box protein 21 [Source:MGI Symbol;Acc:MGI:1924223]	3026.3	2274.3	**-1.30**	0.01346
Txndc11	thioredoxin domain containing 11 [Source:MGI Symbol;Acc:MGI:1923620]	472.5	361.2	**-1.30**	0.01734
Gimap8	GTPase, IMAP family member 8 [Source:MGI Symbol;Acc:MGI:2685303]	58.6	41.8	**-1.30**	0.02644
Mbd6	methyl-CpG binding domain protein 6 [Source:MGI Symbol;Acc:MGI:106378]	423.0	336.2	**-1.31**	6.36E-05
Zkscan17	zinc finger with KRAB and SCAN domains 17 [Source:MGI Symbol;Acc:MGI:2679270]	617.9	476.2	**-1.31**	0.0003349
Sorbs3	sorbin and SH3 domain containing 3 [Source:MGI Symbol;Acc:MGI:700013]	465.6	376.2	**-1.31**	0.00441
Pitpnc1	phosphatidylinositol transfer protein, cytoplasmic 1 [Source:MGI Symbol;Acc:MGI:1919045]	531.3	398.9	**-1.31**	0.01084
Zfp595	zinc finger protein 595 [Source:MGI Symbol;Acc:MGI:3040707]	64.2	52.9	**-1.31**	0.01228
Ppat	phosphoribosyl pyrophosphate amidotransferase [Source:MGI Symbol;Acc:MGI:2387203]	455.8	350.8	**-1.31**	0.02924
Tspan4	tetraspanin 4 [Source:MGI Symbol;Acc:MGI:1928097]	792.7	587.5	**-1.31**	0.04338
Urad	ureidoimidazoline (2-oxo-4-hydroxy-4-carboxy-5) decarboxylase [Source:MGI Symbol;Acc:MGI:3647519]	447.8	345.2	**-1.31**	0.04507
Trip10	thyroid hormone receptor interactor 10 [Source:MGI Symbol;Acc:MGI:2146901]	149.8	113.7	**-1.32**	0.0004559
Tbc1d2b	TBC1 domain family, member 2B [Source:MGI Symbol;Acc:MGI:1914266]	545.6	411.9	**-1.32**	0.004604
Plec	plectin [Source:MGI Symbol;Acc:MGI:1277961]	1183.2	883.8	**-1.32**	0.01535
Phf11c	PHD finger protein 11C [Source:MGI Symbol;Acc:MGI:3648476]	46.5	36.9	**-1.32**	0.02012

Slc6a8	solute carrier family 6 (neurotransmitter transporter, creatine), member 8 [Source:MGI Symbol;Acc:MGI:2147834]	142.5	99.2	**-1.32**	0.02259
Smim1	small integral membrane protein 1 [Source:MGI Symbol;Acc:MGI:1916109]	89.5	63.5	**-1.32**	0.02435
Ppargc1a	peroxisome proliferative activated receptor, gamma, coactivator 1 alpha [Source:MGI Symbol;Acc:MGI:1342774]	197.1	142.5	**-1.32**	0.03245
Tmppe	transmembrane protein with metallophosphoesterase domain [Source:MGI Symbol;Acc:MGI:5317335]	40.8	29.3	**-1.32**	0.04471
Tk1	thymidine kinase 1 [Source:MGI Symbol;Acc:MGI:98763]	314.7	235.8	**-1.32**	0.04818
Scamp1	secretory carrier membrane protein 1 [Source:MGI Symbol;Acc:MGI:1349480]	1279.3	923.6	**-1.33**	0.0001033
C2	complement component 2 (within H-2S) [Source:MGI Symbol;Acc:MGI:88226]	581.4	433.7	**-1.33**	0.0004672
Pou6f1	POU domain, class 6, transcription factor 1 [Source:MGI Symbol;Acc:MGI:102935]	157.2	115.7	**-1.33**	0.002565
Npc1	NPC intracellular cholesterol transporter 1 [Source:MGI Symbol;Acc:MGI:1097712]	3192.7	2376.1	**-1.33**	0.002619
Crlf2	cytokine receptor-like factor 2 [Source:MGI Symbol;Acc:MGI:1889506]	117.8	85.8	**-1.33**	0.00368
Amigo1	adhesion molecule with Ig like domain 1 [Source:MGI Symbol;Acc:MGI:2653612]	141.2	103.7	**-1.33**	0.005201
Cnst	consortin, connexin sorting protein [Source:MGI Symbol;Acc:MGI:2445141]	174.2	129.7	**-1.33**	0.01585
Ugt1a6a	UDP glucuronosyltransferase 1 family, polypeptide A6A [Source:MGI Symbol;Acc:MGI:2137698]	51.0	40.8	**-1.33**	0.04723
Vegfa	vascular endothelial growth factor A [Source:MGI Symbol;Acc:MGI:103178]	1325.1	1058.1	**-1.34**	7.41E-11
Rbfox2	RNA binding protein, fox-1 homolog (C. elegans) 2 [Source:MGI Symbol;Acc:MGI:1933973]	422.2	325.2	**-1.34**	1.69E-05
Tmtc2	transmembrane and tetratricopeptide repeat containing 2 [Source:MGI Symbol;Acc:MGI:1914057]	240.1	174.8	**-1.34**	0.006062
Brpf3	bromodomain and PHD finger containing, 3 [Source:MGI Symbol;Acc:MGI:2146836]	500.7	381.5	**-1.34**	0.007243
Fes	feline sarcoma oncogene [Source:MGI Symbol;Acc:MGI:95514]	87.5	69.1	**-1.34**	0.01183
Hic2	hypermethylated in cancer 2 [Source:MGI Symbol;Acc:MGI:1929869]	45.9	34.0	**-1.34**	0.01983
Dnajb5	DnaJ heat shock protein family (Hsp40) member B5 [Source:MGI Symbol;Acc:MGI:1930018]	65.8	46.8	**-1.34**	0.02063
Dclk3	doublecortin-like kinase 3 [Source:MGI Symbol;Acc:MGI:3039580]	251.6	173.5	**-1.34**	0.0231
Snhg1	small nucleolar RNA host gene 1 [Source:MGI Symbol;Acc:MGI:3763743]	122.4	87.4	**-1.34**	0.03169
Cdhr5	cadherin-related family member 5 [Source:MGI Symbol;Acc:MGI:1919290]	1057.7	783.0	**-1.35**	3.28E-05
Tmem25	transmembrane protein 25 [Source:MGI Symbol;Acc:MGI:1918937]	409.5	335.3	**-1.35**	0.0001134
Dnase1l3	deoxyribonuclease 1-like 3 [Source:MGI Symbol;Acc:MGI:1314633]	879.0	635.6	**-1.35**	0.001445
Fcgr2b	Fc receptor, IgG, low affinity IIb [Source:MGI Symbol;Acc:MGI:95499]	763.1	557.7	**-1.35**	0.001605
H2-Q7	histocompatibility 2, Q region locus 7 [Source:MGI Symbol;Acc:MGI:95936]	880.3	606.7	**-1.35**	0.00324
Il1r1	interleukin 1 receptor, type I [Source:MGI Symbol;Acc:MGI:96545]	364.7	280.2	**-1.35**	0.006182
Atrn	attractin [Source:MGI Symbol;Acc:MGI:1341628]	1484.2	1062.4	**-1.35**	0.01029
Rapgef4	Rap guanine nucleotide exchange factor (GEF) 4 [Source:MGI Symbol;Acc:MGI:1917723]	1752.7	1308.7	**-1.35**	0.01093
Lifr	LIF receptor alpha [Source:MGI Symbol;Acc:MGI:96788]	3398.1	2652.7	**-1.35**	0.01336

Dnajb9	DnaJ heat shock protein family (Hsp40) member B9 [Source:MGI Symbol;Acc:MGI:1351618]	991.3	714.2	**-1.35**	0.01554
Pcgf2	polycomb group ring finger 2 [Source:MGI Symbol;Acc:MGI:99161]	64.4	45.2	**-1.35**	0.01655
Cbfa2t3	CBFA2/RUNX1 translocation partner 3 [Source:MGI Symbol;Acc:MGI:1338013]	60.1	41.9	**-1.35**	0.02642
Ccnl2	cyclin L2 [Source:MGI Symbol;Acc:MGI:1927119]	644.6	557.0	**-1.36**	1.25E-06
Smoc1	SPARC related modular calcium binding 1 [Source:MGI Symbol;Acc:MGI:1929878]	2674.7	1997.4	**-1.36**	3.04E-06
Zfp358	zinc finger protein 358 [Source:MGI Symbol;Acc:MGI:2153740]	248.9	181.1	**-1.36**	1.24E-05
Mmp14	matrix metallopeptidase 14 (membrane-inserted) [Source:MGI Symbol;Acc:MGI:101900]	572.9	392.0	**-1.36**	0.001423
Pdia5	protein disulfide isomerase associated 5 [Source:MGI Symbol;Acc:MGI:1919849]	849.3	613.0	**-1.36**	0.002595
Ces3b	carboxylesterase 3B [Source:MGI Symbol;Acc:MGI:3644960]	4789.2	3599.4	**-1.36**	0.01284
Pabpc4	poly(A) binding protein, cytoplasmic 4 [Source:MGI Symbol;Acc:MGI:2385206]	489.4	360.4	**-1.37**	3.09E-07
Col4a3bp	collagen, type IV, alpha 3 (Goodpasture antigen) binding protein [Source:MGI Symbol;Acc:MGI:1915268]	972.8	712.1	**-1.37**	2.01E-06
Galc	galactosylceramidase [Source:MGI Symbol;Acc:MGI:95636]	161.1	110.6	**-1.37**	0.00137
Sall2	spalt like transcription factor 2 [Source:MGI Symbol;Acc:MGI:1354373]	61.2	41.4	**-1.37**	0.002458
Tle2	transducin-like enhancer of split 2 [Source:MGI Symbol;Acc:MGI:104635]	90.1	73.2	**-1.37**	0.003842
Fzd5	frizzled class receptor 5 [Source:MGI Symbol;Acc:MGI:108571]	196.3	131.3	**-1.37**	0.004423
Abcc5	ATP-binding cassette, sub-family C (CFTR/MRP), member 5 [Source:MGI Symbol;Acc:MGI:1351644]	39.4	29.4	**-1.37**	0.02671
Fgb	fibrinogen beta chain [Source:MGI Symbol;Acc:MGI:99501]	84079.6	59538.1	**-1.38**	4.60E-06
Nrbp2	nuclear receptor binding protein 2 [Source:MGI Symbol;Acc:MGI:2385017]	571.4	451.7	**-1.38**	8.05E-05
Cyp2j9	cytochrome P450, family 2, subfamily j, polypeptide 9 [Source:MGI Symbol;Acc:MGI:1921769]	83.3	63.0	**-1.38**	0.002876
Srd5a1	steroid 5 alpha-reductase 1 [Source:MGI Symbol;Acc:MGI:98400]	1397.2	984.2	**-1.38**	0.007561
Zyg11a	zyg-11 family member A, cell cycle regulator [Source:MGI Symbol;Acc:MGI:2446208]	58.0	41.1	**-1.38**	0.03228
Fkbp5	FK506 binding protein 5 [Source:MGI Symbol;Acc:MGI:104670]	592.7	409.3	**-1.38**	0.04818
Agtr1a	angiotensin II receptor, type 1a [Source:MGI Symbol;Acc:MGI:87964]	1315.0	912.6	**-1.39**	3.75E-07
Zfp385b	zinc finger protein 385B [Source:MGI Symbol;Acc:MGI:2444734]	315.5	225.8	**-1.39**	0.000322
Fga	fibrinogen alpha chain [Source:MGI Symbol;Acc:MGI:1316726]	94162.5	65723.6	**-1.39**	0.0003361
Setd1b	SET domain containing 1B [Source:MGI Symbol;Acc:MGI:2652820]	517.4	365.8	**-1.39**	0.003396
Cabyr	calcium-binding tyrosine-(Y)-phosphorylation regulated (fibrousheathin 2) [Source:MGI Symbol;Acc:MGI:1918382]	16.0	8.6	**-1.39**	0.04867
Rbm33	RNA binding motif protein 33 [Source:MGI Symbol;Acc:MGI:1919670]	661.6	486.6	**-1.39**	0.0003913
Igsf8	immunoglobulin superfamily, member 8 [Source:MGI Symbol;Acc:MGI:2154090]	220.8	147.5	**-1.39**	0.001551
Abca8a	ATP-binding cassette, sub-family A (ABC1), member 8a [Source:MGI Symbol;Acc:MGI:2386846]	2073.7	1456.5	**-1.39**	0.004195
Pafah1b3	platelet-activating factor acetylhydrolase, isoform 1b, subunit 3 [Source:MGI Symbol;Acc:MGI:108414]	61.9	39.9	**-1.39**	0.008835

Smim22	small integral membrane protein 22 [Source:MGI Symbol;Acc:MGI:3643379]	42.9	26.7	**-1.39**	0.03306
Mup-ps20	major urinary protein, pseudogene 20 [Source:MGI Symbol;Acc:MGI:3651976]	115.6	45.9	**-1.39**	0.04091
Cyp2c67	cytochrome P450, family 2, subfamily c, polypeptide 67 [Source:MGI Symbol;Acc:MGI:3612288]	3835.0	2729.5	**-1.40**	2.39E-05
Apom	apolipoprotein M [Source:MGI Symbol;Acc:MGI:1930124]	3606.0	2529.4	**-1.40**	5.03E-05
Zfp516	zinc finger protein 516 [Source:MGI Symbol;Acc:MGI:2443957]	84.3	58.7	**-1.40**	0.00135
Tent5a	terminal nucleotidyltransferase 5A [Source:MGI Symbol;Acc:MGI:2670964]	263.2	180.3	**-1.40**	0.002711
Irs2	insulin receptor substrate 2 [Source:MGI Symbol;Acc:MGI:109334]	454.3	298.9	**-1.40**	0.006306
Syne3	spectrin repeat containing, nuclear envelope family member 3 [Source:MGI Symbol;Acc:MGI:2442408]	135.3	88.3	**-1.40**	0.01347
Scd1	stearoyl-Coenzyme A desaturase 1 [Source:MGI Symbol;Acc:MGI:98239]	374533.2	198130.1	**-1.40**	0.04944
Enpep	glutamyl aminopeptidase [Source:MGI Symbol;Acc:MGI:106645]	2109.2	1386.3	**-1.41**	0.0007335
Pgap1	post-GPI attachment to proteins 1 [Source:MGI Symbol;Acc:MGI:2443342]	468.1	327.9	**-1.41**	0.0008394
Dll1	delta like canonical Notch ligand 1 [Source:MGI Symbol;Acc:MGI:104659]	52.8	36.3	**-1.41**	0.001605
Slc7a2	solute carrier family 7 (cationic amino acid transporter, y+ system), member 2 [Source:MGI Symbol;Acc:MGI:99828]	11160.9	7630.1	**-1.41**	0.004604
Susd4	sushi domain containing 4 [Source:MGI Symbol;Acc:MGI:2138351]	444.1	316.0	**-1.41**	0.008512
Tedc2	tubulin epsilon and delta complex 2 [Source:MGI Symbol;Acc:MGI:1919266]	1017.3	777.1	**-1.41**	0.01701
Ugt2b38	UDP glucuronosyltransferase 2 family, polypeptide B38 [Source:MGI Symbol;Acc:MGI:2140794]	46.4	18.7	**-1.41**	0.0324
Slco2a1	solute carrier organic anion transporter family, member 2a1 [Source:MGI Symbol;Acc:MGI:1346021]	867.5	600.2	**-1.42**	0.0001651
Exoc3l2	exocyst complex component 3-like 2 [Source:MGI Symbol;Acc:MGI:1921713]	121.1	83.3	**-1.42**	0.0002598
Cbx7	chromobox 7 [Source:MGI Symbol;Acc:MGI:1196439]	244.5	168.6	**-1.42**	0.0002778
Col13a1	collagen, type XIII, alpha 1 [Source:MGI Symbol;Acc:MGI:1277201]	74.8	51.9	**-1.42**	0.005395
Grem2	gremlin 2, DAN family BMP antagonist [Source:MGI Symbol;Acc:MGI:1344367]	306.8	196.8	**-1.42**	0.007165
Cyp39a1	cytochrome P450, family 39, subfamily a, polypeptide 1 [Source:MGI Symbol;Acc:MGI:1927096]	337.0	209.9	**-1.42**	0.0147
AA986860	expressed sequence AA986860 [Source:MGI Symbol;Acc:MGI:2138143]	14.3	9.7	**-1.42**	0.03798
Slc7a5	solute carrier family 7 (cationic amino acid transporter, y+ system), member 5 [Source:MGI Symbol;Acc:MGI:1298205]	46.8	31.6	**-1.43**	0.002955
Exoc3l	exocyst complex component 3-like [Source:MGI Symbol;Acc:MGI:3041195]	52.0	34.8	**-1.43**	0.003522
Cd207	CD207 antigen [Source:MGI Symbol;Acc:MGI:2180021]	22.2	11.9	**-1.43**	0.03126
Cyp17a1	cytochrome P450, family 17, subfamily a, polypeptide 1 [Source:MGI Symbol;Acc:MGI:88586]	613.8	306.6	**-1.43**	0.03487
Cps1	carbamoyl-phosphate synthetase 1 [Source:MGI Symbol;Acc:MGI:891996]	46243.9	30712.3	**-1.44**	0.002115
Fgfr2	fibroblast growth factor receptor 2 [Source:MGI Symbol;Acc:MGI:95523]	497.0	332.8	**-1.45**	1.80E-05
Gabbr2	gamma-aminobutyric acid (GABA) B receptor, 2 [Source:MGI Symbol;Acc:MGI:2386030]	1051.2	649.9	**-1.45**	0.004551
Ihh	Indian hedgehog [Source:MGI Symbol;Acc:MGI:96533]	37.2	23.8	**-1.45**	0.02314

Slc10a1	solute carrier family 10 (sodium/bile acid cotransporter family), member 1 [Source:MGI Symbol;Acc:MGI:97379]	18932.7	12668.1	**-1.46**	2.95E-05
Plcxd1	phosphatidylinositol-specific phospholipase C, X domain containing 1 [Source:MGI Symbol;Acc:MGI:2685422]	54.5	32.9	**-1.46**	0.003724
Rec8	REC8 meiotic recombination protein [Source:MGI Symbol;Acc:MGI:1929645]	42.3	23.8	**-1.46**	0.01074
Zfpm1	zinc finger protein, multitype 1 [Source:MGI Symbol;Acc:MGI:1095400]	1116.2	757.0	**-1.47**	3.14E-06
Gm2a	GM2 ganglioside activator protein [Source:MGI Symbol;Acc:MGI:95762]	2404.0	1543.6	**-1.47**	3.77E-05
Gja4	gap junction protein, alpha 4 [Source:MGI Symbol;Acc:MGI:95715]	46.7	31.2	**-1.47**	0.009164
Ugcg	UDP-glucose ceramide glucosyltransferase [Source:MGI Symbol;Acc:MGI:1332243]	463.2	294.5	**-1.48**	1.29E-06
Adamts7	a disintegrin-like and metallopeptidase (reprolysin type) with thrombospondin type 1 motif, 7 [Source:MGI Symbol;Acc:MGI:1347346]	133.4	87.1	**-1.48**	0.0001913
Avpr1a	arginine vasopressin receptor 1A [Source:MGI Symbol;Acc:MGI:1859216]	325.6	198.8	**-1.48**	0.006657
Acmsd	amino carboxymuconate semialdehyde decarboxylase [Source:MGI Symbol;Acc:MGI:2386323]	202.3	113.0	**-1.48**	0.009164
Pck1	phosphoenolpyruvate carboxykinase 1, cytosolic [Source:MGI Symbol;Acc:MGI:97501]	30743.5	17222.3	**-1.49**	0.0149
Etnppl	ethanolamine phosphate phospholyase [Source:MGI Symbol;Acc:MGI:1919010]	2583.8	1568.4	**-1.51**	0.0003939
Syne4	spectrin repeat containing, nuclear envelope family member 4 [Source:MGI Symbol;Acc:MGI:2141950]	24.9	14.2	**-1.51**	0.008649
Kcnt2	potassium channel, subfamily T, member 2 [Source:MGI Symbol;Acc:MGI:3036273]	25.1	13.0	**-1.51**	0.01191
Cyp4a31	cytochrome P450, family 4, subfamily a, polypeptide 31 [Source:MGI Symbol;Acc:MGI:3028580]	261.9	104.0	**-1.51**	0.01232
Slc38a2	solute carrier family 38, member 2 [Source:MGI Symbol;Acc:MGI:1915010]	1867.7	1271.3	**-1.52**	0.001559
Acpp	acid phosphatase, prostate [Source:MGI Symbol;Acc:MGI:1928480]	275.6	138.1	**-1.52**	0.004843
Ikzf4	IKAROS family zinc finger 4 [Source:MGI Symbol;Acc:MGI:1343139]	13.7	6.1	**-1.52**	0.01088
C8b	complement component 8, beta polypeptide [Source:MGI Symbol;Acc:MGI:88236]	4459.6	2904.9	**-1.53**	2.31E-05
Snhg11	small nucleolar RNA host gene 11 [Source:MGI Symbol;Acc:MGI:2441845]	56.6	37.4	**-1.53**	0.000725
Itih5	inter-alpha (globulin) inhibitor H5 [Source:MGI Symbol;Acc:MGI:1925751]	224.5	130.9	**-1.53**	0.001368
Smarcd3	SWI/SNF related, matrix associated, actin dependent regulator of chromatin, subfamily d, member 3 [Source:MGI Symbol;Acc:MGI:1914243]	22.3	13.9	**-1.53**	0.002818
Cyp4a32	cytochrome P450, family 4, subfamily a, polypeptide 32 [Source:MGI Symbol;Acc:MGI:3717148]	433.8	254.0	**-1.53**	0.003061
Cyp4a12b	cytochrome P450, family 4, subfamily a, polypeptide 12B [Source:MGI Symbol;Acc:MGI:3611747]	931.8	468.9	**-1.53**	0.00823
Tgfbr3l	transforming growth factor, beta receptor III-like [Source:MGI Symbol;Acc:MGI:3833469]	13.0	8.2	**-1.54**	0.007319
Cd9	CD9 antigen [Source:MGI Symbol;Acc:MGI:88348]	428.2	243.1	**-1.55**	0.0002466
Cspg5	chondroitin sulfate proteoglycan 5 [Source:MGI Symbol;Acc:MGI:1352747]	21.8	9.1	**-1.56**	0.006452
Rnf145	ring finger protein 145 [Source:MGI Symbol;Acc:MGI:1921565]	581.0	326.8	**-1.58**	2.02E-06
Lgals4	lectin, galactose binding, soluble 4 [Source:MGI Symbol;Acc:MGI:107536]	130.7	72.8	**-1.58**	0.0001426
Cadm4	cell adhesion molecule 4 [Source:MGI Symbol;Acc:MGI:2449088]	138.7	83.8	**-1.59**	2.05E-06

Sox12	SRY (sex determining region Y)-box 12 [Source:MGI Symbol;Acc:MGI:98360]	113.2	64.0	**-1.61**	2.15E-05
Tff3	trefoil factor 3, intestinal [Source:MGI Symbol;Acc:MGI:104638]	37.1	15.4	**-1.61**	0.001994
St3gal5	ST3 beta-galactoside alpha-2,3-sialyltransferase 5 [Source:MGI Symbol;Acc:MGI:1339963]	2464.8	1193.9	**-1.62**	0.0004875
Sds	serine dehydratase [Source:MGI Symbol;Acc:MGI:98270]	3961.3	2026.8	**-1.62**	0.0007093
Pde6c	phosphodiesterase 6C, cGMP specific, cone, alpha prime [Source:MGI Symbol;Acc:MGI:105956]	33.5	16.1	**-1.64**	0.0007941
Lpar2	lysophosphatidic acid receptor 2 [Source:MGI Symbol;Acc:MGI:1858422]	55.6	29.3	**-1.65**	5.57E-06
Slc35g1	solute carrier family 35, member G1 [Source:MGI Symbol;Acc:MGI:2444789]	399.4	215.0	**-1.72**	1.23E-07
Cyp2c40	cytochrome P450, family 2, subfamily c, polypeptide 40 [Source:MGI Symbol;Acc:MGI:1306815]	21.3	5.8	**-1.73**	0.00033
Srgap3	SLIT-ROBO Rho GTPase activating protein 3 [Source:MGI Symbol;Acc:MGI:2152938]	106.8	43.8	**-1.82**	3.39E-05
Adam11	a disintegrin and metallopeptidase domain 11 [Source:MGI Symbol;Acc:MGI:1098667]	134.1	53.2	**-1.88**	2.00E-06
Grm8	glutamate receptor, metabotropic 8 [Source:MGI Symbol;Acc:MGI:1351345]	21.7	8.1	**-1.91**	6.89E-06
Fabp5	fatty acid binding protein 5, epidermal [Source:MGI Symbol;Acc:MGI:101790]	1273.4	156.8	**-1.93**	3.18E-06
Igfbp2	insulin-like growth factor binding protein 2 [Source:MGI Symbol;Acc:MGI:96437]	7680.0	3614.7	**-1.95**	1.38E-11
Adgrf1	adhesion G protein-coupled receptor F1 [Source:MGI Symbol;Acc:MGI:1924846]	122.3	40.0	**-2.06**	3.18E-07
Lepr	leptin receptor [Source:MGI Symbol;Acc:MGI:104993]	296.0	43.2	**-3.48**	3.89E-24
