# Supplementary material for: Effects of Lingonberry (Vaccinium vitis-idaea L.) Supplementation on Hepatic Gene Expression in High-Fat Diet Fed Mice
Source: Nutrients. 2021 Oct 21;13(11):3693. doi: 10.3390/nu13113693 (PMC8623941; doi:10.3390/nu13113693)
Supplement: Supplementary file 1 [file nutrients-13-03693-s001.zip › Table S3.pdf]

Table S3: Functions of the mouse cytochrome enzymes significantly affected by lingonberry supplementation in the high-fat (HF+LGB) diet, validated with PCR.

	High-fat (HF) diet vs. low-fat (LF) diet				Lingonberry- supplemented high-fat diet (HF+LGB) vs. high-fat (HF) diet					
	FC (NGS)	p-value[1] (NGS)	FC (PCR)	p-value[2] (PCR)	FC (NGS)	p-value[1] (NGS)	FC (PCR)	p-value[2] (PCR)	Functions in mouse	Biological process
**HF+LGB vs HF: downregulated**										
Cyp46a1 Mean expression level (NGS): LF: 90.8 HF: 92.2 HF+LGB: 32.3	1.02	0.96	-1.12	> 0.9999	**-1.82**	2.40E-05	-2.86	0.0081	Cholesterol 24-hydroxylase activity. Steroid hydroxylase activity.	Cholesterol catabolic process. Xenobiotic metabolic process.
**HF+LGB vs HF: upregulated**										
Cyp3a11 Mean expression level (NGS): LF: 10345.2 HF: 6628.2 HF+LGB: 27365.0	-1.19	0.64	-1.56	0.0986	**2.85**	1.59E-22	3.83	< 0.0001	Monooxygenase activity. Oxidoreductase activity.	Oxidative demethylation. Steroid metabolic process. Xenobiotic metabolic process.
Cyp2c55 Mean expression level (NGS): LF: 34.8 HF: 27.3 HF+LGB: 84.5	-1.14	0.66	-1.39	0.6249	**2.22**	2.41E-11	3.14	< 0.0001	Metabolizes arachidonic acid mainly to 19-hydroxyeicosatetraenoic acid (HETE). Steroid hydroxylase activity.	Arachidonic acid metabolic process. Xenobiotic metabolic process. Organic acid metabolic process.
Cyp2c29 Mean expression level (NGS): LF: 9786.0 HF: 8826.3 HF+LGB: 16460.9	-1.04	0.89	-1.24	0.1827	**1.75**	4.57E-14	1.87	< 0.0001	Metabolizes arachidonic acid to produce 14.15-cis-epoxyeicosatrienoic acid (EET). Steroid hydroxylase activity.	Epoxygenase P450 pathway. Xenobiotic metabolic process.
Cyp3a59 Mean expression level (NGS): LF: 85.7 HF: 93.5 HF+LGB: 168.1	1.14	0.71	3.45	0.6249	**1.55**	0.0008	2.68	0.0451	Steroid hydroxylase activity.	Oxidative demethylation. Steroid metabolic process.

Red denotes upregulation and blue downregulation. Information presented in the columns ”Functions in mouse” and ”Biological process” is obtained from NCBI Gene [42] and UniProt [43] databases. Cyp = Cytochrome P450. [1] p-values are adjusted by false discovery rate (FDR). [2]p-values are adjusted with Bonferroni correction.
