# Supplementary material for: Effects of Lingonberry (Vaccinium vitis-idaea L.) Supplementation on Hepatic Gene Expression in High-Fat Diet Fed Mice
Source: Nutrients. 2021 Oct 21;13(11):3693. doi: 10.3390/nu13113693 (PMC8623941; doi:10.3390/nu13113693)
Supplement: Supplementary file 1 [file nutrients-13-03693-s001.zip › Table S4.pdf]

Table S4. All significantly differentially expressed genes in the lingonberry supplemented high-fat diet group (HF+LGB) compared to the high-fat (HF) diet group. Mean expression levels are given as DESeq2-normalized counts. p-values are adjusted by false discovery rate (FDR).

**Gene**	**Name**	**Mean (HF)**	**Mean (HF+LGB)**	**FC**	**p-value (FDR adj.)**
Cyp3a11	cytochrome P450, family 3, subfamily a, polypeptide 11 [Source:MGI Symbol;Acc:MGI:88609]	6628.2	27365.0	**2.85**	1.59E-22
Cyp2c55	cytochrome P450, family 2, subfamily c, polypeptide 55 [Source:MGI Symbol;Acc:MGI:1919332]	27.3	84.5	**2.22**	2.41E-11
Adgrf1	adhesion G protein-coupled receptor F1 [Source:MGI Symbol;Acc:MGI:1924846]	36.2	143.8	**1.91**	3.08E-06
Emp2	epithelial membrane protein 2 [Source:MGI Symbol;Acc:MGI:1098726]	125.1	253.5	**1.79**	1.66E-08
Cyp2c29	cytochrome P450, family 2, subfamily c, polypeptide 29 [Source:MGI Symbol;Acc:MGI:103238]	8826.3	16460.9	**1.75**	4.57E-14
Grid1	glutamate receptor, ionotropic, delta 1 [Source:MGI Symbol;Acc:MGI:95812]	17.4	42.0	**1.75**	4.32E-05
Hsd17b6	hydroxysteroid (17-beta) dehydrogenase 6 [Source:MGI Symbol;Acc:MGI:1351670]	1545.8	3033.3	**1.74**	7.72E-08
Ces2a	carboxylesterase 2A [Source:MGI Symbol;Acc:MGI:2142491]	1987.2	3581.2	**1.73**	3.76E-17
Fam222a	family with sequence similarity 222, member A [Source:MGI Symbol;Acc:MGI:3605543]	16.2	38.3	**1.72**	0.0001053
Igfbp2	insulin-like growth factor binding protein 2 [Source:MGI Symbol;Acc:MGI:96437]	3388.8	6263.7	**1.71**	7.44E-09
Asap3	ArfGAP with SH3 domain, ankyrin repeat and PH domain 3 [Source:MGI Symbol;Acc:MGI:2684986]	77.5	140.1	**1.69**	1.81E-10
Neb	nebulin [Source:MGI Symbol;Acc:MGI:97292]	161.9	337.1	**1.68**	7.88E-05
Slc7a2	solute carrier family 7 (cationic amino acid transporter, y+ system), member 2 [Source:MGI Symbol;Acc:MGI:99828]	7225.2	13116.6	**1.65**	8.80E-07
Scnn1a	sodium channel, nonvoltage-gated 1 alpha [Source:MGI Symbol;Acc:MGI:101782]	302.0	511.2	**1.59**	4.76E-08
Sorbs3	sorbin and SH3 domain containing 3 [Source:MGI Symbol;Acc:MGI:700013]	333.5	564.2	**1.59**	4.76E-08
Slco1a4	solute carrier organic anion transporter family, member 1a4 [Source:MGI Symbol;Acc:MGI:1351896]	489.7	951.0	**1.58**	0.0007586
Gsta2	glutathione S-transferase, alpha 2 (Yc2) [Source:MGI Symbol;Acc:MGI:95863]	176.3	444.3	**1.58**	0.002626
Csad	cysteine sulfinic acid decarboxylase [Source:MGI Symbol;Acc:MGI:2180098]	1955.8	4247.9	**1.57**	0.0029
Enho	energy homeostasis associated [Source:MGI Symbol;Acc:MGI:1916888]	84.9	234.9	**1.57**	0.003017
Gsta4	glutathione S-transferase, alpha 4 [Source:MGI Symbol;Acc:MGI:1309515]	619.4	1065.4	**1.56**	4.53E-05
Aph1c	aph1 homolog C, gamma secretase subunit [Source:MGI Symbol;Acc:MGI:1915568]	7.1	14.8	**1.56**	0.003691
Slc22a26	solute carrier family 22 (organic cation transporter), member 26 [Source:MGI Symbol;Acc:MGI:2385316]	11.7	25.9	**1.56**	0.003821
Atp6v0d2	ATPase, H+ transporting, lysosomal V0 subunit D2 [Source:MGI Symbol;Acc:MGI:1924415]	51.1	78.8	**1.55**	0.0001459
Cyp3a59	cytochrome P450, family 3, subfamily a, polypeptide 59 [Source:MGI Symbol;Acc:MGI:3769707]	92.5	166.4	**1.55**	0.0007957
Afmid	arylformamidase [Source:MGI Symbol;Acc:MGI:2448704]	423.6	629.0	**1.52**	6.64E-06
Akr1c19	aldo-keto reductase family 1, member C19 [Source:MGI Symbol;Acc:MGI:2653678]	993.6	1617.2	**1.52**	0.0002397
Hamp	hepcidin antimicrobial peptide [Source:MGI Symbol;Acc:MGI:1933533]	12002.0	19080.1	**1.52**	0.0008929

Cyp2c23	cytochrome P450, family 2, subfamily c, polypeptide 23 [Source:MGI Symbol;Acc:MGI:1888897]	3948.2	5855.5	**1.49**	2.96E-05
Tmc4	transmembrane channel-like gene family 4 [Source:MGI Symbol;Acc:MGI:2669035]	13.4	19.6	**1.49**	0.009889
Tmem266	transmembrane protein 266 [Source:MGI Symbol;Acc:MGI:2142980]	12.7	23.6	**1.49**	0.01029
Ces1b	carboxylesterase 1B [Source:MGI Symbol;Acc:MGI:3779470]	874.0	1262.1	**1.48**	7.07E-06
Cth	cystathionase (cystathionine gamma-lyase) [Source:MGI Symbol;Acc:MGI:1339968]	4489.8	6720.0	**1.48**	0.0001609
Slco2a1	solute carrier organic anion transporter family, member 2a1 [Source:MGI Symbol;Acc:MGI:1346021]	600.2	914.8	**1.48**	0.0003746
Malat1	metastasis associated lung adenocarcinoma transcript 1 (non-coding RNA) [Source:MGI Symbol;Acc:MGI:1919539]	1803.4	1954.7	**1.48**	0.003689
Foxa3	forkhead box A3 [Source:MGI Symbol;Acc:MGI:1347477]	530.5	716.4	**1.47**	3.67E-05
Tk1	thymidine kinase 1 [Source:MGI Symbol;Acc:MGI:98763]	235.8	347.3	**1.47**	0.0009929
Cyp7a1	cytochrome P450, family 7, subfamily a, polypeptide 1 [Source:MGI Symbol;Acc:MGI:106091]	1447.1	3249.6	**1.47**	0.01557
Lrit2	leucine-rich repeat, immunoglobulin-like and transmembrane domains 2 [Source:MGI Symbol;Acc:MGI:2444885]	86.0	124.7	**1.46**	1.36E-05
Gls2	glutaminase 2 (liver, mitochondrial) [Source:MGI Symbol;Acc:MGI:2143539]	5346.9	7970.8	**1.46**	0.001205
Agxt	alanine-glyoxylate aminotransferase [Source:MGI Symbol;Acc:MGI:1329033]	4547.3	6855.1	**1.46**	0.002623
Gucy2c	guanylate cyclase 2c [Source:MGI Symbol;Acc:MGI:106903]	6.6	20.6	**1.45**	0.01229
Rnase4	ribonuclease, RNase A family 4 [Source:MGI Symbol;Acc:MGI:1926217]	12573.7	17139.7	**1.44**	1.12E-06
Epop	elongin BC and polycomb repressive complex 2 associated protein [Source:MGI Symbol;Acc:MGI:2143991]	37.9	56.9	**1.44**	0.00325
Acot1	acyl-CoA thioesterase 1 [Source:MGI Symbol;Acc:MGI:1349396]	279.3	433.4	**1.44**	0.01131
Cyp4a14	cytochrome P450, family 4, subfamily a, polypeptide 14 [Source:MGI Symbol;Acc:MGI:1096550]	7275.3	12604.3	**1.44**	0.01498
Ripor2	RHO family interacting cell polarization regulator 2 [Source:MGI Symbol;Acc:MGI:2444879]	32.3	56.5	**1.44**	0.01974
Ranbp3l	RAN binding protein 3-like [Source:MGI Symbol;Acc:MGI:2444654]	8.4	13.9	**1.44**	0.02283
Vldlr	very low density lipoprotein receptor [Source:MGI Symbol;Acc:MGI:98935]	109.5	166.5	**1.43**	0.007598
Flvcr2	feline leukemia virus subgroup C cellular receptor 2 [Source:MGI Symbol;Acc:MGI:2384974]	67.1	107.3	**1.43**	0.01696
Osgin1	oxidative stress induced growth inhibitor 1 [Source:MGI Symbol;Acc:MGI:1919089]	902.4	1728.9	**1.43**	0.02755
C3	complement component 3 [Source:MGI Symbol;Acc:MGI:88227]	65964.4	96192.1	**1.42**	0.000161
Arl13b	ADP-ribosylation factor-like 13B [Source:MGI Symbol;Acc:MGI:1915396]	85.4	121.4	**1.41**	0.000188
Hal	histidine ammonia lyase [Source:MGI Symbol;Acc:MGI:96010]	5132.5	7305.0	**1.41**	0.002421
Osbp2	oxysterol binding protein 2 [Source:MGI Symbol;Acc:MGI:1921559]	12.9	19.7	**1.41**	0.02405
Tafa2	TAFA chemokine like family member 2 [Source:MGI Symbol;Acc:MGI:2143691]	17.7	46.0	**1.41**	0.03175
Tmem25	transmembrane protein 25 [Source:MGI Symbol;Acc:MGI:1918937]	335.3	411.5	**1.40**	1.44E-06
Fbxo21	F-box protein 21 [Source:MGI Symbol;Acc:MGI:1924223]	2274.3	3145.7	**1.40**	5.91E-06

Pde4c	phosphodiesterase 4C, cAMP specific [Source:MGI Symbol;Acc:MGI:99556]	156.6	206.4	**1.40**	2.82E-05
Unc93a2	unc-93 homolog A2 [Source:MGI Symbol;Acc:MGI:3712668]	43.3	63.9	**1.40**	0.006322
Glt1d1	glycosyltransferase 1 domain containing 1 [Source:MGI Symbol;Acc:MGI:2442755]	339.6	438.5	**1.39**	0.0004248
Cryl1	crystallin, lambda 1 [Source:MGI Symbol;Acc:MGI:1915881]	256.3	367.6	**1.39**	0.0007138
Aox3	aldehyde oxidase 3 [Source:MGI Symbol;Acc:MGI:1918974]	9059.7	12562.6	**1.39**	0.0008279
Ttc39b	tetratricopeptide repeat domain 39B [Source:MGI Symbol;Acc:MGI:1917113]	527.8	742.7	**1.39**	0.004675
Sox12	SRY (sex determining region Y)-box 12 [Source:MGI Symbol;Acc:MGI:98360]	64.0	91.4	**1.39**	0.007351
Tcf24	transcription factor 24 [Source:MGI Symbol;Acc:MGI:3780500]	18.0	98.2	**1.39**	0.0103
Sds	serine dehydratase [Source:MGI Symbol;Acc:MGI:98270]	2143.7	2800.6	**1.39**	0.01229
P2rx3	purinergic receptor P2X, ligand-gated ion channel, 3 [Source:MGI Symbol;Acc:MGI:1097160]	8.2	14.0	**1.39**	0.04578
Ihh	Indian hedgehog [Source:MGI Symbol;Acc:MGI:96533]	23.8	32.9	**1.39**	0.04646
Col4a5	collagen, type IV, alpha 5 [Source:MGI Symbol;Acc:MGI:88456]	23.8	32.9	**1.39**	0.04826
Dhtkd1	dehydrogenase E1 and transketolase domain containing 1 [Source:MGI Symbol;Acc:MGI:2445096]	947.1	1281.7	**1.39**	0.0004383
Adamts7	a disintegrin-like and metallopeptidase (reprolysin type) with thrombospondin type 1 motif, 7 [Source:MGI Symbol;Acc:MGI:1347346]	87.1	116.3	**1.39**	0.001295
Dop1b	DOP1 leucine zipper like protein B [Source:MGI Symbol;Acc:MGI:1917278]	381.0	532.8	**1.39**	0.002316
Dll4	delta like canonical Notch ligand 4 [Source:MGI Symbol;Acc:MGI:1859388]	44.8	66.2	**1.39**	0.0199
Grm8	glutamate receptor, metabotropic 8 [Source:MGI Symbol;Acc:MGI:1351345]	7.6	16.2	**1.39**	0.04768
Sema4g	sema domain, immunoglobulin domain (Ig), transmembrane domain (TM) and short cytoplasmic domain, (semaphorin) 4G [Source:MGI Symbol;Acc:MGI:1347047]	3584.2	4634.9	**1.38**	2.95E-05
Sgsm2	small G protein signaling modulator 2 [Source:MGI Symbol;Acc:MGI:2144695]	134.4	183.7	**1.38**	0.00154
Etnppl	ethanolamine phosphate phospholyase [Source:MGI Symbol;Acc:MGI:1919010]	1568.4	2374.0	**1.38**	0.03362
Cyp3a25	cytochrome P450, family 3, subfamily a, polypeptide 25 [Source:MGI Symbol;Acc:MGI:1930638]	4294.6	5923.8	**1.37**	2.13E-05
Slc25a48	solute carrier family 25, member 48 [Source:MGI Symbol;Acc:MGI:2145373]	355.0	483.4	**1.37**	0.002091
Zfp867	zinc finger protein 867 [Source:MGI Symbol;Acc:MGI:2681848]	46.4	60.2	**1.37**	0.002967
Kmt2a	lysine (K)-specific methyltransferase 2A [Source:MGI Symbol;Acc:MGI:96995]	231.8	309.1	**1.37**	0.007017
Aifm3	apoptosis-inducing factor, mitochondrion-associated 3 [Source:MGI Symbol;Acc:MGI:1919418]	83.4	118.3	**1.37**	0.007053
Papss2	3'-phosphoadenosine 5'-phosphosulfate synthase 2 [Source:MGI Symbol;Acc:MGI:1330223]	2693.7	3754.5	**1.37**	0.0091
Ngef	neuronal guanine nucleotide exchange factor [Source:MGI Symbol;Acc:MGI:1858414]	546.3	740.7	**1.37**	0.01845
Zc3h11a	zinc finger CCCH type containing 11A [Source:MGI Symbol;Acc:MGI:1917829]	47.2	64.0	**1.37**	0.02876
Itih2	inter-alpha trypsin inhibitor, heavy chain 2 [Source:MGI Symbol;Acc:MGI:96619]	13960.0	18862.9	**1.36**	0.0005758
Dqx1	DEAQ RNA-dependent ATPase [Source:MGI Symbol;Acc:MGI:2136388]	148.3	191.9	**1.36**	0.003168
Cps1	carbamoyl-phosphate synthetase 1 [Source:MGI Symbol;Acc:MGI:891996]	30712.3	39913.5	**1.36**	0.01186

Ces1e	carboxylesterase 1E [Source:MGI Symbol;Acc:MGI:95432]	3497.1	4821.8	**1.35**	8.21E-05
Ocln	occludin [Source:MGI Symbol;Acc:MGI:106183]	218.9	292.3	**1.35**	0.001125
Amigo1	adhesion molecule with Ig like domain 1 [Source:MGI Symbol;Acc:MGI:2653612]	103.7	136.3	**1.35**	0.001385
Tcp11l2	t-complex 11 (mouse) like 2 [Source:MGI Symbol;Acc:MGI:2444679]	315.5	414.6	**1.35**	0.002028
Ang	angiogenin, ribonuclease, RNase A family, 5 [Source:MGI Symbol;Acc:MGI:88022]	2275.4	2941.3	**1.35**	0.01048
Il17rb	interleukin 17 receptor B [Source:MGI Symbol;Acc:MGI:1355292]	100.4	137.6	**1.35**	0.01439
Ablim1	actin-binding LIM protein 1 [Source:MGI Symbol;Acc:MGI:1194500]	258.3	370.8	**1.35**	0.02588
Asns	asparagine synthetase [Source:MGI Symbol;Acc:MGI:1350929]	26.6	117.6	**1.35**	0.02718
Slc38a4	solute carrier family 38, member 4 [Source:MGI Symbol;Acc:MGI:1916604]	19185.8	25069.6	**1.34**	5.93E-06
Ivd	isovaleryl coenzyme A dehydrogenase [Source:MGI Symbol;Acc:MGI:1929242]	3324.8	4345.1	**1.34**	8.65E-05
Cyp4f15	cytochrome P450, family 4, subfamily f, polypeptide 15 [Source:MGI Symbol;Acc:MGI:2146921]	1629.0	2077.9	**1.34**	0.0001279
Tle2	transducin-like enhancer of split 2 [Source:MGI Symbol;Acc:MGI:104635]	73.2	83.1	**1.34**	0.002752
Cecr2	CECR2, histone acetyl-lysine reader [Source:MGI Symbol;Acc:MGI:1923799]	188.3	241.5	**1.34**	0.003024
Ppp4r4	protein phosphatase 4, regulatory subunit 4 [Source:MGI Symbol;Acc:MGI:1921771]	68.0	85.6	**1.34**	0.003172
Abca8a	ATP-binding cassette, sub-family A (ABC1), member 8a [Source:MGI Symbol;Acc:MGI:2386846]	1456.5	1887.0	**1.34**	0.008746
Zfp275	zinc finger protein 275 [Source:MGI Symbol;Acc:MGI:1350985]	131.3	173.1	**1.34**	0.01167
Spsb4	splA/ryanodine receptor domain and SOCS box containing 4 [Source:MGI Symbol;Acc:MGI:2183445]	58.2	84.3	**1.34**	0.02325
Tnfrsf14	tumor necrosis factor receptor superfamily, member 14 (herpesvirus entry mediator) [Source:MGI Symbol;Acc:MGI:2675303]	42.7	55.3	**1.34**	0.04006
Disp2	dispatched RND tramsporter family member 2 [Source:MGI Symbol;Acc:MGI:2388733]	33.7	50.9	**1.34**	0.04696
Akap8l	A kinase (PRKA) anchor protein 8-like [Source:MGI Symbol;Acc:MGI:1860606]	190.6	222.6	**1.33**	8.70E-05
Setd1b	SET domain containing 1B [Source:MGI Symbol;Acc:MGI:2652820]	365.8	485.3	**1.33**	0.006854
Zfp595	zinc finger protein 595 [Source:MGI Symbol;Acc:MGI:3040707]	52.9	65.9	**1.33**	0.008621
Rnf39	ring finger protein 39 [Source:MGI Symbol;Acc:MGI:2156378]	59.5	77.3	**1.33**	0.01229
Zfp36	zinc finger protein 36 [Source:MGI Symbol;Acc:MGI:99180]	1341.4	1638.1	**1.33**	0.01381
Lifr	LIF receptor alpha [Source:MGI Symbol;Acc:MGI:96788]	2652.7	3303.2	**1.33**	0.02301
Ccl9	chemokine (C-C motif) ligand 9 [Source:MGI Symbol;Acc:MGI:104533]	602.1	819.8	**1.33**	0.03395
Ggt6	gamma-glutamyltransferase 6 [Source:MGI Symbol;Acc:MGI:1918772]	229.9	308.0	**1.32**	0.0029
Dclk3	doublecortin-like kinase 3 [Source:MGI Symbol;Acc:MGI:3039580]	173.5	234.5	**1.32**	0.01426
Dclre1c	DNA cross-link repair 1C [Source:MGI Symbol;Acc:MGI:2441769]	64.4	84.2	**1.32**	0.02648
Setd4	SET domain containing 4 [Source:MGI Symbol;Acc:MGI:2136890]	59.1	66.5	**1.32**	0.02876
Aass	aminoadipate-semialdehyde synthase [Source:MGI Symbol;Acc:MGI:1353573]	4361.6	5503.4	**1.31**	0.0002212
Gnmt	glycine N-methyltransferase [Source:MGI Symbol;Acc:MGI:1202304]	25071.1	31859.5	**1.31**	0.003741

Lrit1	leucine-rich repeat, immunoglobulin-like and transmembrane domains 1 [Source:MGI Symbol;Acc:MGI:2385320]	268.5	352.4	**1.31**	0.004971
Srd5a2	steroid 5 alpha-reductase 2 [Source:MGI Symbol;Acc:MGI:2150380]	34.8	46.3	**1.31**	0.0317
Irs2	insulin receptor substrate 2 [Source:MGI Symbol;Acc:MGI:109334]	298.9	402.9	**1.31**	0.03764
Crtc2	CREB regulated transcription coactivator 2 [Source:MGI Symbol;Acc:MGI:1921593]	405.7	497.8	**1.30**	8.80E-07
Rnf145	ring finger protein 145 [Source:MGI Symbol;Acc:MGI:1921565]	332.9	439.1	**1.30**	0.002344
Sptbn2	spectrin beta, non-erythrocytic 2 [Source:MGI Symbol;Acc:MGI:1313261]	1492.3	1925.5	**1.30**	0.008029
Bach1	BTB and CNC homology 1, basic leucine zipper transcription factor 1 [Source:MGI Symbol;Acc:MGI:894680]	608.4	797.7	**1.30**	0.02199
Gcnt4	glucosaminyl (N-acetyl) transferase 4, core 2 (beta-1,6-N-acetylglucosaminyltransferase) [Source:MGI Symbol;Acc:MGI:2684919]	100.7	128.6	**1.30**	0.03032
Pdia5	protein disulfide isomerase associated 5 [Source:MGI Symbol;Acc:MGI:1919849]	613.0	766.5	**1.29**	1.27E-06
Gpat4	glycerol-3-phosphate acyltransferase 4 [Source:MGI Symbol;Acc:MGI:2142716]	3160.5	3943.1	**1.29**	0.0001372
Khnyn	KH and NYN domain containing [Source:MGI Symbol;Acc:MGI:2451333]	553.5	699.5	**1.29**	0.0002183
Plekhg3	pleckstrin homology domain containing, family G (with RhoGef domain) member 3 [Source:MGI Symbol;Acc:MGI:2388284]	540.0	660.2	**1.29**	0.0006909
Mtx3	metaxin 3 [Source:MGI Symbol;Acc:MGI:2686040]	241.4	294.4	**1.29**	0.005964
Map3k21	mitogen-activated protein kinase kinase kinase 21 [Source:MGI Symbol;Acc:MGI:2385307]	88.4	114.5	**1.29**	0.01105
Urad	ureidoimidazoline (2-oxo-4-hydroxy-4-carboxy-5) decarboxylase [Source:MGI Symbol;Acc:MGI:3647519]	345.2	420.1	**1.29**	0.01462
Csrp2	cysteine and glycine-rich protein 2 [Source:MGI Symbol;Acc:MGI:1202907]	372.2	470.0	**1.29**	0.02429
Wdr90	WD repeat domain 90 [Source:MGI Symbol;Acc:MGI:1921267]	54.0	66.4	**1.29**	0.02648
Prlr	prolactin receptor [Source:MGI Symbol;Acc:MGI:97763]	1592.7	2057.3	**1.29**	0.03003
Zfp516	zinc finger protein 516 [Source:MGI Symbol;Acc:MGI:2443957]	58.7	75.6	**1.29**	0.0322
Tut4	terminal uridylyl transferase 4 [Source:MGI Symbol;Acc:MGI:2445126]	317.3	405.8	**1.29**	0.03837
Lrp1	low density lipoprotein receptor-related protein 1 [Source:MGI Symbol;Acc:MGI:96828]	7833.8	10214.5	**1.29**	0.0482
Arhgap4	Rho GTPase activating protein 4 [Source:MGI Symbol;Acc:MGI:2159577]	41.4	53.3	**1.29**	0.04838
Epb41l5	erythrocyte membrane protein band 4.1 like 5 [Source:MGI Symbol;Acc:MGI:103006]	585.5	738.5	**1.28**	2.35E-06
Dbt	dihydrolipoamide branched chain transacylase E2 [Source:MGI Symbol;Acc:MGI:105386]	1575.1	1973.8	**1.28**	0.0001473
Marf1	meiosis regulator and mRNA stability 1 [Source:MGI Symbol;Acc:MGI:2444505]	966.4	1176.3	**1.28**	0.0004031
Kdm3a	lysine (K)-specific demethylase 3A [Source:MGI Symbol;Acc:MGI:98847]	181.2	221.0	**1.28**	0.0004065
Fads6	fatty acid desaturase domain family, member 6 [Source:MGI Symbol;Acc:MGI:3039592]	2607.0	3293.8	**1.28**	0.0007991
Zcchc2	zinc finger, CCHC domain containing 2 [Source:MGI Symbol;Acc:MGI:2444114]	372.9	477.6	**1.28**	0.0126
Helz2	helicase with zinc finger 2, transcriptional coactivator [Source:MGI Symbol;Acc:MGI:2385169]	910.6	1125.5	**1.28**	0.02306

Zfp292	zinc finger protein 292 [Source:MGI Symbol;Acc:MGI:1353423]	199.6	245.2	**1.28**	0.0235
Bmp6	bone morphogenetic protein 6 [Source:MGI Symbol;Acc:MGI:88182]	75.2	96.1	**1.28**	0.02497
Mir22hg	Mir22 host gene (non-protein coding) [Source:MGI Symbol;Acc:MGI:1914348]	383.8	474.7	**1.28**	0.02755
Ppm1k	protein phosphatase 1K (PP2C domain containing) [Source:MGI Symbol;Acc:MGI:2442111]	1606.6	2050.6	**1.28**	0.03666
C8b	complement component 8, beta polypeptide [Source:MGI Symbol;Acc:MGI:88236]	2904.9	3664.4	**1.28**	0.04588
Gch1	GTP cyclohydrolase 1 [Source:MGI Symbol;Acc:MGI:95675]	2810.8	3487.0	**1.27**	0.0001134
Cxcl12	chemokine (C-X-C motif) ligand 12 [Source:MGI Symbol;Acc:MGI:103556]	4456.8	5544.4	**1.27**	0.0003473
Prkd3	protein kinase D3 [Source:MGI Symbol;Acc:MGI:1922542]	4251.2	5189.3	**1.27**	0.0006588
Dcaf6	DDB1 and CUL4 associated factor 6 [Source:MGI Symbol;Acc:MGI:1921356]	593.9	729.1	**1.27**	0.0009032
Camkk2	calcium/calmodulin-dependent protein kinase kinase 2, beta [Source:MGI Symbol;Acc:MGI:2444812]	131.5	158.8	**1.27**	0.001098
Fam193b	family with sequence similarity 193, member B [Source:MGI Symbol;Acc:MGI:2385851]	249.5	241.8	**1.27**	0.01751
Rnf38	ring finger protein 38 [Source:MGI Symbol;Acc:MGI:1920719]	99.9	123.7	**1.27**	0.02079
Ell	elongation factor RNA polymerase II [Source:MGI Symbol;Acc:MGI:109377]	408.2	497.5	**1.27**	2.03E-05
Tmem63a	transmembrane protein 63a [Source:MGI Symbol;Acc:MGI:2384789]	260.0	327.9	**1.27**	0.0004031
Kansl3	KAT8 regulatory NSL complex subunit 3 [Source:MGI Symbol;Acc:MGI:1918055]	715.7	856.0	**1.27**	0.0007138
Sun1	Sad1 and UNC84 domain containing 1 [Source:MGI Symbol;Acc:MGI:1924303]	234.9	288.5	**1.27**	0.001586
Ddx17	DEAD (Asp-Glu-Ala-Asp) box polypeptide 17 [Source:MGI Symbol;Acc:MGI:1914290]	1457.4	1634.7	**1.27**	0.004639
Ahcy	S-adenosylhomocysteine hydrolase [Source:MGI Symbol;Acc:MGI:87968]	8118.4	9858.2	**1.27**	0.005594
Abat	4-aminobutyrate aminotransferase [Source:MGI Symbol;Acc:MGI:2443582]	6344.2	7891.9	**1.27**	0.02139
Tet3	tet methylcytosine dioxygenase 3 [Source:MGI Symbol;Acc:MGI:2446229]	256.7	317.5	**1.27**	0.0253
Clk1	CDC-like kinase 1 [Source:MGI Symbol;Acc:MGI:107403]	463.8	507.4	**1.27**	0.02999
Abcb11	ATP-binding cassette, sub-family B (MDR/TAP), member 11 [Source:MGI Symbol;Acc:MGI:1351619]	6455.6	8149.7	**1.27**	0.03756
Irf6	interferon regulatory factor 6 [Source:MGI Symbol;Acc:MGI:1859211]	457.0	563.0	**1.27**	0.0398
Rbm33	RNA binding motif protein 33 [Source:MGI Symbol;Acc:MGI:1919670]	486.6	574.0	**1.27**	0.04164
Bcat2	branched chain aminotransferase 2, mitochondrial [Source:MGI Symbol;Acc:MGI:1276534]	314.7	391.8	**1.27**	0.04579
Zfp568	zinc finger protein 568 [Source:MGI Symbol;Acc:MGI:2142347]	184.8	231.3	**1.27**	0.04826
Agtr1a	angiotensin II receptor, type 1a [Source:MGI Symbol;Acc:MGI:87964]	912.6	1138.2	**1.26**	0.002344
Kyat1	kynurenine aminotransferase 1 [Source:MGI Symbol;Acc:MGI:1917516]	1441.1	1749.6	**1.26**	0.00348
Slc38a3	solute carrier family 38, member 3 [Source:MGI Symbol;Acc:MGI:1923507]	27635.7	34173.6	**1.26**	0.006145
Hpd	4-hydroxyphenylpyruvic acid dioxygenase [Source:MGI Symbol;Acc:MGI:96213]	30601.6	36550.5	**1.26**	0.006151
Kansl1l	KAT8 regulatory NSL complex subunit 1-like [Source:MGI Symbol;Acc:MGI:1915941]	156.0	181.7	**1.26**	0.006164
Slc8b1	solute carrier family 8 (sodium/lithium/calcium exchanger), member B1 [Source:MGI Symbol;Acc:MGI:2180781]	767.3	938.1	**1.26**	0.01553

Amdhd1	amidohydrolase domain containing 1 [Source:MGI Symbol;Acc:MGI:1919011]	3715.3	4528.7	**1.26**	0.0158
Marveld2	MARVEL (membrane-associating) domain containing 2 [Source:MGI Symbol;Acc:MGI:2446166]	169.6	212.7	**1.26**	0.02502
Slc6a6	solute carrier family 6 (neurotransmitter transporter, taurine), member 6 [Source:MGI Symbol;Acc:MGI:98488]	1323.9	1646.4	**1.26**	0.02855
Tdg	thymine DNA glycosylase [Source:MGI Symbol;Acc:MGI:108247]	61.6	72.6	**1.26**	0.0307
Insr	insulin receptor [Source:MGI Symbol;Acc:MGI:96575]	1951.0	2438.9	**1.26**	0.03488
Hyal1	hyaluronoglucosaminidase 1 [Source:MGI Symbol;Acc:MGI:96298]	243.9	286.2	**1.26**	0.03669
Upf2	UPF2 regulator of nonsense transcripts homolog (yeast) [Source:MGI Symbol;Acc:MGI:2449307]	269.8	319.2	**1.25**	0.0002736
Ints6	integrator complex subunit 6 [Source:MGI Symbol;Acc:MGI:1202397]	238.2	276.3	**1.25**	0.002307
Cds2	CDP-diacylglycerol synthase (phosphatidate cytidylyltransferase) 2 [Source:MGI Symbol;Acc:MGI:1332236]	1479.9	1831.8	**1.25**	0.006183
Luc7l2	LUC7-like 2 (S. cerevisiae) [Source:MGI Symbol;Acc:MGI:2183260]	828.9	944.6	**1.25**	0.009124
Fam234b	family with sequence similarity 234, member B [Source:MGI Symbol;Acc:MGI:1921775]	340.3	414.0	**1.25**	0.01589
Cyp3a13	cytochrome P450, family 3, subfamily a, polypeptide 13 [Source:MGI Symbol;Acc:MGI:88610]	1705.0	2124.6	**1.25**	0.02246
Tnrc6a	trinucleotide repeat containing 6a [Source:MGI Symbol;Acc:MGI:2385292]	508.0	589.7	**1.25**	0.02344
Bdp1	B double prime 1, subunit of RNA polymerase III transcription initiation factor IIIB [Source:MGI Symbol;Acc:MGI:1347077]	181.3	214.0	**1.25**	0.04905
Dcaf11	DDB1 and CUL4 associated factor 11 [Source:MGI Symbol;Acc:MGI:90168]	5471.0	6527.8	**1.24**	0.00117
Setdb1	SET domain, bifurcated 1 [Source:MGI Symbol;Acc:MGI:1934229]	313.6	369.2	**1.24**	0.003821
Mbtd1	mbt domain containing 1 [Source:MGI Symbol;Acc:MGI:2143977]	267.7	319.3	**1.24**	0.005724
Cyp2c67	cytochrome P450, family 2, subfamily c, polypeptide 67 [Source:MGI Symbol;Acc:MGI:3612288]	2729.5	3250.8	**1.24**	0.0126
Cyp39a1	cytochrome P450, family 39, subfamily a, polypeptide 1 [Source:MGI Symbol;Acc:MGI:1927096]	209.9	249.3	**1.24**	0.02325
Smg6	Smg-6 homolog, nonsense mediated mRNA decay factor (C. elegans) [Source:MGI Symbol;Acc:MGI:2144117]	477.7	576.3	**1.24**	0.02766
Fnbp4	formin binding protein 4 [Source:MGI Symbol;Acc:MGI:1860513]	154.0	168.0	**1.24**	0.03269
Itpkc	inositol 1,4,5-trisphosphate 3-kinase C [Source:MGI Symbol;Acc:MGI:2442554]	83.7	99.9	**1.24**	0.03556
Zcchc7	zinc finger, CCHC domain containing 7 [Source:MGI Symbol;Acc:MGI:2442912]	106.4	125.0	**1.24**	0.04579
Fam76b	family with sequence similarity 76, member B [Source:MGI Symbol;Acc:MGI:1920076]	92.2	111.7	**1.24**	0.04798
Vegfa	vascular endothelial growth factor A [Source:MGI Symbol;Acc:MGI:103178]	1058.1	1186.7	**1.23**	0.0001057
Peli1	pellino 1 [Source:MGI Symbol;Acc:MGI:1914495]	307.1	367.3	**1.23**	0.0002736
Kdm5a	lysine (K)-specific demethylase 5A [Source:MGI Symbol;Acc:MGI:2136980]	448.0	529.3	**1.23**	0.0008025
Retreg1	reticulophagy regulator 1 [Source:MGI Symbol;Acc:MGI:1913520]	1251.4	1472.0	**1.23**	0.001063
Akap8	A kinase (PRKA) anchor protein 8 [Source:MGI Symbol;Acc:MGI:1928488]	358.8	405.7	**1.23**	0.01048
Edc4	enhancer of mRNA decapping 4 [Source:MGI Symbol;Acc:MGI:2446249]	204.5	232.9	**1.23**	0.01146

Itpr2	inositol 1,4,5-triphosphate receptor 2 [Source:MGI Symbol;Acc:MGI:99418]	421.0	499.9	**1.23**	0.01735
Wdr81	WD repeat domain 81 [Source:MGI Symbol;Acc:MGI:2681828]	757.4	889.3	**1.23**	0.01986
Crebzf	CREB/ATF bZIP transcription factor [Source:MGI Symbol;Acc:MGI:2675296]	327.0	377.3	**1.23**	0.0218
Zfp397	zinc finger protein 397 [Source:MGI Symbol;Acc:MGI:1916506]	181.6	204.8	**1.23**	0.02564
Map3k5	mitogen-activated protein kinase kinase kinase 5 [Source:MGI Symbol;Acc:MGI:1346876]	402.2	494.5	**1.23**	0.03029
Tfdp2	transcription factor Dp 2 [Source:MGI Symbol;Acc:MGI:107167]	295.7	352.4	**1.23**	0.0307
Zfp955a	zinc finger protein 955A [Source:MGI Symbol;Acc:MGI:4834570]	82.1	97.3	**1.23**	0.03263
Kdm6a	lysine (K)-specific demethylase 6A [Source:MGI Symbol;Acc:MGI:1095419]	164.7	194.1	**1.23**	0.03474
Pgap1	post-GPI attachment to proteins 1 [Source:MGI Symbol;Acc:MGI:2443342]	327.9	391.0	**1.23**	0.03485
Baz2a	bromodomain adjacent to zinc finger domain, 2A [Source:MGI Symbol;Acc:MGI:2151152]	464.7	547.3	**1.23**	0.03723
Med1	mediator complex subunit 1 [Source:MGI Symbol;Acc:MGI:1100846]	319.8	370.4	**1.23**	0.0402
Kdm7a	lysine (K)-specific demethylase 7A [Source:MGI Symbol;Acc:MGI:2443388]	295.8	359.7	**1.23**	0.04144
Smim13	small integral membrane protein 13 [Source:MGI Symbol;Acc:MGI:2652854]	463.0	542.7	**1.23**	0.0421
Adnp2	ADNP homeobox 2 [Source:MGI Symbol;Acc:MGI:2448562]	121.2	147.3	**1.23**	0.04522
Zfp113	zinc finger protein 113 [Source:MGI Symbol;Acc:MGI:1929116]	89.0	104.2	**1.23**	0.04673
Hbs1l	Hbs1-like (S. cerevisiae) [Source:MGI Symbol;Acc:MGI:1891704]	887.0	1038.3	**1.22**	8.63E-05
Sfxn2	sideroflexin 2 [Source:MGI Symbol;Acc:MGI:2137678]	720.5	848.7	**1.22**	0.0002115
Nags	N-acetylglutamate synthase [Source:MGI Symbol;Acc:MGI:2387600]	1027.6	1224.7	**1.22**	0.0004604
Whamm	WAS protein homolog associated with actin, golgi membranes and microtubules [Source:MGI Symbol;Acc:MGI:2142282]	511.8	605.8	**1.22**	0.00314
Prpf38b	PRP38 pre-mRNA processing factor 38 (yeast) domain containing B [Source:MGI Symbol;Acc:MGI:1914171]	369.1	410.5	**1.22**	0.004042
Slc17a2	solute carrier family 17 (sodium phosphate), member 2 [Source:MGI Symbol;Acc:MGI:2443098]	2277.0	2649.0	**1.22**	0.005023
Pah	phenylalanine hydroxylase [Source:MGI Symbol;Acc:MGI:97473]	17210.3	19854.7	**1.22**	0.00567
Slc25a22	solute carrier family 25 (mitochondrial carrier, glutamate), member 22 [Source:MGI Symbol;Acc:MGI:1915517]	2563.3	3017.3	**1.22**	0.007361
Zfp263	zinc finger protein 263 [Source:MGI Symbol;Acc:MGI:1921370]	136.6	155.5	**1.22**	0.0126
Slc6a12	solute carrier family 6 (neurotransmitter transporter, betaine/GABA), member 12 [Source:MGI Symbol;Acc:MGI:95628]	1035.3	1257.9	**1.22**	0.01495
Sertad2	SERTA domain containing 2 [Source:MGI Symbol;Acc:MGI:1931026]	295.3	356.3	**1.22**	0.03003
Maml1	mastermind like transcriptional coactivator 1 [Source:MGI Symbol;Acc:MGI:1890504]	263.7	316.1	**1.22**	0.03285
Cep68	centrosomal protein 68 [Source:MGI Symbol;Acc:MGI:2667663]	205.1	241.9	**1.22**	0.03485
Keg1	kidney expressed gene 1 [Source:MGI Symbol;Acc:MGI:1928492]	2873.3	3417.9	**1.22**	0.04321
Erbb3	erb-b2 receptor tyrosine kinase 3 [Source:MGI Symbol;Acc:MGI:95411]	1962.8	2381.1	**1.22**	0.04578

Scamp1	secretory carrier membrane protein 1 [Source:MGI Symbol;Acc:MGI:1349480]	923.6	1109.2	**1.21**	0.0005475
Tle1	transducin-like enhancer of split 1 [Source:MGI Symbol;Acc:MGI:104636]	749.8	861.8	**1.21**	0.0006909
Rbfox2	RNA binding protein, fox-1 homolog (C. elegans) 2 [Source:MGI Symbol;Acc:MGI:1933973]	325.2	375.7	**1.21**	0.001794
Smc5	structural maintenance of chromosomes 5 [Source:MGI Symbol;Acc:MGI:2385088]	214.4	244.8	**1.21**	0.005724
Taok3	TAO kinase 3 [Source:MGI Symbol;Acc:MGI:3041177]	1043.3	1269.6	**1.21**	0.0107
Zbtb44	zinc finger and BTB domain containing 44 [Source:MGI Symbol;Acc:MGI:1925123]	784.4	916.1	**1.21**	0.01426
Arid4b	AT rich interactive domain 4B (RBP1-like) [Source:MGI Symbol;Acc:MGI:2137512]	259.4	291.1	**1.21**	0.02403
Zhx3	zinc fingers and homeoboxes 3 [Source:MGI Symbol;Acc:MGI:2444772]	1962.8	2381.1	**1.21**	0.04082
Atg2a	autophagy related 2A [Source:MGI Symbol;Acc:MGI:1916291]	847.2	999.0	**1.21**	0.04335
Farp2	FERM, RhoGEF and pleckstrin domain protein 2 [Source:MGI Symbol;Acc:MGI:2385126]	339.7	403.4	**1.21**	0.04788
Tmem63b	transmembrane protein 63b [Source:MGI Symbol;Acc:MGI:2387609]	1093.0	1278.6	**1.21**	0.001527
Slc35d1	solute carrier family 35 (UDP-glucuronic acid/UDP-N-acetylgalactosamine dual transporter), member D1 [Source:MGI Symbol;Acc:MGI:2140361]	2133.8	2535.6	**1.21**	0.002526
Acadsb	acyl-Coenzyme A dehydrogenase, short/branched chain [Source:MGI Symbol;Acc:MGI:1914135]	2358.1	2718.2	**1.21**	0.003017
Mms19	MMS19 cytosolic iron-sulfur assembly component [Source:MGI Symbol;Acc:MGI:1919449]	314.9	360.2	**1.21**	0.005597
Msl2	MSL complex subunit 2 [Source:MGI Symbol;Acc:MGI:1925103]	389.8	453.7	**1.21**	0.007017
Tns2	tensin 2 [Source:MGI Symbol;Acc:MGI:2387586]	1103.5	1297.8	**1.21**	0.008746
Sec14l4	SEC14-like lipid binding 4 [Source:MGI Symbol;Acc:MGI:2144095]	2929.2	3401.2	**1.21**	0.008964
Sprtn	SprT-like N-terminal domain [Source:MGI Symbol;Acc:MGI:2685351]	169.6	194.4	**1.21**	0.02319
Prpf39	pre-mRNA processing factor 39 [Source:MGI Symbol;Acc:MGI:104602]	215.6	240.8	**1.21**	0.02608
Zmym2	zinc finger, MYM-type 2 [Source:MGI Symbol;Acc:MGI:1923257]	312.8	356.0	**1.21**	0.03339
Ccnl2	cyclin L2 [Source:MGI Symbol;Acc:MGI:1927119]	557.0	553.0	**1.21**	0.03393
Mllt10	myeloid/lymphoid or mixed-lineage leukemia; translocated to, 10 [Source:MGI Symbol;Acc:MGI:1329038]	311.9	356.9	**1.21**	0.04817
Rnf214	ring finger protein 214 [Source:MGI Symbol;Acc:MGI:2444451]	284.4	327.1	**1.21**	0.04896
Jup	junction plakoglobin [Source:MGI Symbol;Acc:MGI:96650]	703.1	820.3	**1.20**	0.002527
Dlg3	discs large MAGUK scaffold protein 3 [Source:MGI Symbol;Acc:MGI:1888986]	319.7	373.2	**1.20**	0.003261
Cdc40	cell division cycle 40 [Source:MGI Symbol;Acc:MGI:1918963]	378.0	438.2	**1.20**	0.003287
Scarb1	scavenger receptor class B, member 1 [Source:MGI Symbol;Acc:MGI:893578]	2425.6	2809.0	**1.20**	0.009534
Sf3b1	splicing factor 3b, subunit 1 [Source:MGI Symbol;Acc:MGI:1932339]	1405.6	1586.7	**1.20**	0.01146
Fubp1	far upstream element (FUSE) binding protein 1 [Source:MGI Symbol;Acc:MGI:1196294]	296.4	328.4	**1.20**	0.01642
Dpyd	dihydropyrimidine dehydrogenase [Source:MGI Symbol;Acc:MGI:2139667]	7947.4	9202.4	**1.20**	0.01764
Atp11b	ATPase, class VI, type 11B [Source:MGI Symbol;Acc:MGI:1923545]	670.1	779.1	**1.20**	0.03274

Dmtf1	cyclin D binding myb-like transcription factor 1 [Source:MGI Symbol;Acc:MGI:1344415]	169.5	187.5	**1.20**	0.03339
Sardh	sarcosine dehydrogenase [Source:MGI Symbol;Acc:MGI:2183102]	14259.8	16784.0	**1.20**	0.03575
Llgl2	LLGL2 scribble cell polarity complex component [Source:MGI Symbol;Acc:MGI:1918843]	421.9	493.9	**1.20**	0.03879
Srsf11	serine/arginine-rich splicing factor 11 [Source:MGI Symbol;Acc:MGI:1916457]	562.3	608.8	**1.19**	0.0001371
Zmym5	zinc finger, MYM-type 5 [Source:MGI Symbol;Acc:MGI:3041170]	459.5	512.0	**1.19**	0.004236
Baiap2l1	BAI1-associated protein 2-like 1 [Source:MGI Symbol;Acc:MGI:1914148]	486.5	554.5	**1.19**	0.004956
Sun2	Sad1 and UNC84 domain containing 2 [Source:MGI Symbol;Acc:MGI:2443011]	1739.9	2029.5	**1.19**	0.0107
Cfhr1	complement factor H-related 1 [Source:MGI Symbol;Acc:MGI:2138169]	2753.4	3155.4	**1.19**	0.01245
Sart3	squamous cell carcinoma antigen recognized by T cells 3 [Source:MGI Symbol;Acc:MGI:1858230]	193.2	221.3	**1.19**	0.01426
Tesk2	testis-specific kinase 2 [Source:MGI Symbol;Acc:MGI:2385204]	198.2	222.2	**1.19**	0.01523
Samd8	sterile alpha motif domain containing 8 [Source:MGI Symbol;Acc:MGI:1914880]	506.0	592.2	**1.19**	0.01879
Hip1r	huntingtin interacting protein 1 related [Source:MGI Symbol;Acc:MGI:1352504]	315.5	356.8	**1.19**	0.02247
Zdhhc20	zinc finger, DHHC domain containing 20 [Source:MGI Symbol;Acc:MGI:1923215]	234.7	272.7	**1.19**	0.02648
Ankzf1	ankyrin repeat and zinc finger domain containing 1 [Source:MGI Symbol;Acc:MGI:1098746]	240.1	266.6	**1.19**	0.02958
Dpys	dihydropyrimidinase [Source:MGI Symbol;Acc:MGI:1928679]	4336.9	4957.4	**1.19**	0.02978
Aox1	aldehyde oxidase 1 [Source:MGI Symbol;Acc:MGI:88035]	1312.2	1558.5	**1.19**	0.03331
Ptcd1	pentatricopeptide repeat domain 1 [Source:MGI Symbol;Acc:MGI:1919049]	158.6	182.6	**1.19**	0.03369
Tmem131l	transmembrane 131 like [Source:MGI Symbol;Acc:MGI:2443399]	388.5	456.1	**1.19**	0.03519
Ldlrad3	low density lipoprotein receptor class A domain containing 3 [Source:MGI Symbol;Acc:MGI:2138856]	126.3	148.9	**1.19**	0.0402
Lcat	lecithin cholesterol acyltransferase [Source:MGI Symbol;Acc:MGI:96755]	5641.4	6538.3	**1.19**	0.04978
Cables2	CDK5 and Abl enzyme substrate 2 [Source:MGI Symbol;Acc:MGI:2182335]	384.2	433.8	**1.18**	0.01857
Abcc6	ATP-binding cassette, sub-family C (CFTR/MRP), member 6 [Source:MGI Symbol;Acc:MGI:1351634]	2384.1	2681.0	**1.18**	0.02271
Med17	mediator complex subunit 17 [Source:MGI Symbol;Acc:MGI:2182585]	166.1	190.8	**1.18**	0.03473
Zcchc8	zinc finger, CCHC domain containing 8 [Source:MGI Symbol;Acc:MGI:1917900]	207.5	230.3	**1.18**	0.03764
Pde3b	phosphodiesterase 3B, cGMP-inhibited [Source:MGI Symbol;Acc:MGI:1333863]	1479.3	1706.0	**1.18**	0.04414
Phlpp1	PH domain and leucine rich repeat protein phosphatase 1 [Source:MGI Symbol;Acc:MGI:2138327]	418.7	481.4	**1.18**	0.04646
Sirt1	sirtuin 1 [Source:MGI Symbol;Acc:MGI:2135607]	158.3	176.7	**1.18**	0.04843
Mthfd1	methylenetetrahydrofolate dehydrogenase (NADP+ dependent), methenyltetrahydrofolate cyclohydrolase, formyltetrahydrofolate synthase [Source:MGI Symbol;Acc:MGI:1342005]	3664.6	4244.1	**1.18**	0.04916
Clk4	CDC like kinase 4 [Source:MGI Symbol;Acc:MGI:1098551]	259.5	266.1	**1.18**	0.04998
Slc30a1	solute carrier family 30 (zinc transporter), member 1 [Source:MGI Symbol;Acc:MGI:1345281]	667.2	751.9	**1.17**	0.005672
Kyat3	kynurenine aminotransferase 3 [Source:MGI Symbol;Acc:MGI:2677849]	2467.6	2768.2	**1.17**	0.01202
Cpsf7	cleavage and polyadenylation specific factor 7 [Source:MGI Symbol;Acc:MGI:1917826]	258.4	276.3	**1.17**	0.01629

Sgms2	sphingomyelin synthase 2 [Source:MGI Symbol;Acc:MGI:1921692]	992.3	1118.6	**1.17**	0.01834
Taok2	TAO kinase 2 [Source:MGI Symbol;Acc:MGI:1915919]	513.2	552.9	**1.17**	0.01846
Sf1	splicing factor 1 [Source:MGI Symbol;Acc:MGI:1095403]	1098.5	1219.9	**1.17**	0.04355
Esco1	establishment of sister chromatid cohesion N-acetyltransferase 1 [Source:MGI Symbol;Acc:MGI:1925055]	159.4	175.1	**1.17**	0.04696
Parp6	poly (ADP-ribose) polymerase family, member 6 [Source:MGI Symbol;Acc:MGI:1914537]	169.9	192.6	**1.17**	0.04923
Ing5	inhibitor of growth family, member 5 [Source:MGI Symbol;Acc:MGI:1922816]	159.2	178.9	**1.17**	0.04981
Ddx5	DEAD (Asp-Glu-Ala-Asp) box polypeptide 5 [Source:MGI Symbol;Acc:MGI:105037]	3686.3	3989.4	**1.16**	0.0005817
Brap	BRCA1 associated protein [Source:MGI Symbol;Acc:MGI:1919649]	2553.1	2840.4	**1.16**	0.001171
Ctdsp2	CTD (carboxy-terminal domain, RNA polymerase II, polypeptide A) small phosphatase 2 [Source:MGI Symbol;Acc:MGI:1098748]	708.5	801.2	**1.16**	0.004843
Dmwd	dystrophia myotonica-containing WD repeat motif [Source:MGI Symbol;Acc:MGI:94907]	280.7	308.7	**1.16**	0.008675
Spin1	spindlin 1 [Source:MGI Symbol;Acc:MGI:109242]	847.8	947.1	**1.16**	0.009208
Pcmtd2	protein-L-isoaspartate (D-aspartate) O-methyltransferase domain containing 2 [Source:MGI Symbol;Acc:MGI:1923927]	732.7	815.0	**1.16**	0.01426
Lgr4	leucine-rich repeat-containing G protein-coupled receptor 4 [Source:MGI Symbol;Acc:MGI:1891468]	933.7	1055.7	**1.16**	0.01457
Acin1	apoptotic chromatin condensation inducer 1 [Source:MGI Symbol;Acc:MGI:1891824]	682.5	734.3	**1.16**	0.01475
Brd8	bromodomain containing 8 [Source:MGI Symbol;Acc:MGI:1925906]	193.3	213.0	**1.16**	0.01735
Slc19a2	solute carrier family 19 (thiamine transporter), member 2 [Source:MGI Symbol;Acc:MGI:1928761]	1713.9	1960.6	**1.16**	0.02356
Tdo2	tryptophan 2,3-dioxygenase [Source:MGI Symbol;Acc:MGI:1928486]	28365.2	31658.7	**1.16**	0.0239
Ces1c	carboxylesterase 1C [Source:MGI Symbol;Acc:MGI:95420]	34910.6	39445.5	**1.16**	0.02957
Oga	O-GlcNAcase [Source:MGI Symbol;Acc:MGI:1932139]	1027.9	1163.8	**1.16**	0.03339
Ehhadh	enoyl-Coenzyme A, hydratase/3-hydroxyacyl Coenzyme A dehydrogenase [Source:MGI Symbol;Acc:MGI:1277964]	5574.8	6491.1	**1.16**	0.03422
Aadat	aminoadipate aminotransferase [Source:MGI Symbol;Acc:MGI:1345167]	1436.9	1610.6	**1.16**	0.03471
Ccdc9	coiled-coil domain containing 9 [Source:MGI Symbol;Acc:MGI:1921443]	267.6	298.9	**1.16**	0.04116
Ythdc1	YTH domain containing 1 [Source:MGI Symbol;Acc:MGI:2443713]	336.4	369.7	**1.16**	0.04453
Pak4	p21 (RAC1) activated kinase 4 [Source:MGI Symbol;Acc:MGI:1917834]	163.3	187.6	**1.16**	0.04817
Ip6k1	inositol hexaphosphate kinase 1 [Source:MGI Symbol;Acc:MGI:1351633]	812.7	895.0	**1.16**	1.59E-05
Ilrun	inflammation and lipid regulator with UBA-like and NBR1-like domains [Source:MGI Symbol;Acc:MGI:106281]	4690.9	5279.4	**1.16**	2.96E-05
Ndfip1	Nedd4 family interacting protein 1 [Source:MGI Symbol;Acc:MGI:1929601]	2635.0	2916.6	**1.16**	6.15E-05
Cdc14b	CDC14 cell division cycle 14B [Source:MGI Symbol;Acc:MGI:2441808]	558.9	637.4	**1.16**	0.02001
Gpkow	G patch domain and KOW motifs [Source:MGI Symbol;Acc:MGI:1859610]	273.9	302.8	**1.16**	0.02167

Kcnn2	potassium intermediate/small conductance calcium-activated channel, subfamily N, member 2 [Source:MGI Symbol;Acc:MGI:2153182]	447.2	494.4	**1.16**	0.03473
Prr14	proline rich 14 [Source:MGI Symbol;Acc:MGI:2384565]	392.0	426.4	**1.16**	0.03853
Rexo1	REX1, RNA exonuclease 1 [Source:MGI Symbol;Acc:MGI:1914182]	316.9	336.8	**1.16**	0.04696
Sqor	sulfide quinone oxidoreductase [Source:MGI Symbol;Acc:MGI:1929899]	2926.7	3298.4	**1.16**	0.04863
Usp19	ubiquitin specific peptidase 19 [Source:MGI Symbol;Acc:MGI:1918722]	872.3	961.6	**1.16**	0.04868
Fech	ferrochelatase [Source:MGI Symbol;Acc:MGI:95513]	2798.1	3070.7	**1.15**	1.70E-05
Stk11	serine/threonine kinase 11 [Source:MGI Symbol;Acc:MGI:1341870]	1549.5	1693.4	**1.15**	0.0006258
Agfg2	ArfGAP with FG repeats 2 [Source:MGI Symbol;Acc:MGI:2443267]	624.6	708.7	**1.15**	0.008376
Rdx	radixin [Source:MGI Symbol;Acc:MGI:97887]	4316.6	4799.2	**1.15**	0.01327
Zc3h14	zinc finger CCCH type containing 14 [Source:MGI Symbol;Acc:MGI:1919824]	701.5	770.2	**1.15**	0.02147
Tprkb	Tp53rk binding protein [Source:MGI Symbol;Acc:MGI:1917036]	2602.8	2891.1	**1.15**	0.03395
Cwf19l1	CWF19-like 1, cell cycle control (S. pombe) [Source:MGI Symbol;Acc:MGI:1919752]	227.9	255.3	**1.15**	0.03648
Stag2	stromal antigen 2 [Source:MGI Symbol;Acc:MGI:1098583]	986.6	1094.8	**1.15**	0.04403
L2hgdh	L-2-hydroxyglutarate dehydrogenase [Source:MGI Symbol;Acc:MGI:2384968]	1028.9	1148.7	**1.15**	0.0464
Fbxo3	F-box protein 3 [Source:MGI Symbol;Acc:MGI:1929084]	2186.9	2435.9	**1.14**	0.0002567
Clcc1	chloride channel CLIC-like 1 [Source:MGI Symbol;Acc:MGI:2385186]	749.3	833.3	**1.14**	0.0004102
Paics	phosphoribosylaminoimidazole carboxylase, phosphoribosylaminoribosylaminoimidazole, succinocarboxamide synthetase [Source:MGI Symbol;Acc:MGI:1914304]	3138.9	3398.6	**1.14**	0.0009373
Zfp207	zinc finger protein 207 [Source:MGI Symbol;Acc:MGI:1340045]	832.1	904.1	**1.14**	0.01029
Impa1	inositol (myo)-1(or 4)-monophosphatase 1 [Source:MGI Symbol;Acc:MGI:1933158]	943.0	1048.6	**1.14**	0.01565
Stard7	START domain containing 7 [Source:MGI Symbol;Acc:MGI:2139090]	2751.8	3065.5	**1.14**	0.01579
Arfgap2	ADP-ribosylation factor GTPase activating protein 2 [Source:MGI Symbol;Acc:MGI:1924288]	1762.0	1931.3	**1.14**	0.01939
Rbm39	RNA binding motif protein 39 [Source:MGI Symbol;Acc:MGI:2157953]	1298.8	1376.4	**1.14**	0.02159
Eprs	glutamyl-prolyl-tRNA synthetase [Source:MGI Symbol;Acc:MGI:97838]	1665.9	1830.6	**1.14**	0.03156
Tcf3	transcription factor 3 [Source:MGI Symbol;Acc:MGI:98510]	278.6	306.8	**1.14**	0.03219
Nectin3	nectin cell adhesion molecule 3 [Source:MGI Symbol;Acc:MGI:1930171]	972.8	1076.2	**1.14**	0.03269
Brf1	BRF1, RNA polymerase III transcription initiation factor 90 kDa subunit [Source:MGI Symbol;Acc:MGI:1919558]	249.8	265.8	**1.14**	0.03668
Son	Son DNA binding protein [Source:MGI Symbol;Acc:MGI:98353]	1800.8	1914.7	**1.14**	0.04874
Akt2	thymoma viral proto-oncogene 2 [Source:MGI Symbol;Acc:MGI:104874]	1163.8	1268.4	**1.13**	0.0005174
Rnf4	ring finger protein 4 [Source:MGI Symbol;Acc:MGI:1201691]	1484.5	1627.9	**1.13**	0.0007675
Trim28	tripartite motif-containing 28 [Source:MGI Symbol;Acc:MGI:109274]	902.4	995.7	**1.13**	0.002871

Heca	hdc homolog, cell cycle regulator [Source:MGI Symbol;Acc:MGI:2685715]	804.3	880.5	**1.13**	0.009214
Jade1	jade family PHD finger 1 [Source:MGI Symbol;Acc:MGI:1925835]	1000.5	1101.4	**1.13**	0.01169
Adhfe1	alcohol dehydrogenase, iron containing, 1 [Source:MGI Symbol;Acc:MGI:1923437]	2118.9	2339.7	**1.13**	0.02147
Zfp110	zinc finger protein 110 [Source:MGI Symbol;Acc:MGI:1890378]	416.2	452.8	**1.13**	0.0385
Amfr	autocrine motility factor receptor [Source:MGI Symbol;Acc:MGI:1345634]	5250.9	5796.3	**1.13**	4.75E-05
Tmem183a	transmembrane protein 183A [Source:MGI Symbol;Acc:MGI:1914729]	2122.6	2277.6	**1.13**	0.02301
Nab1	Ngfi-A binding protein 1 [Source:MGI Symbol;Acc:MGI:107564]	1375.4	1507.8	**1.13**	0.02938
Rnf6	ring finger protein (C3H2C3 type) 6 [Source:MGI Symbol;Acc:MGI:1921382]	559.3	615.2	**1.13**	0.03568
Mcl1	myeloid cell leukemia sequence 1 [Source:MGI Symbol;Acc:MGI:101769]	1931.0	2082.8	**1.13**	0.04981
Eif4b	eukaryotic translation initiation factor 4B [Source:MGI Symbol;Acc:MGI:95304]	5013.6	5421.0	**1.12**	0.04164
Dcaf8	DDB1 and CUL4 associated factor 8 [Source:MGI Symbol;Acc:MGI:91860]	2335.1	2498.6	**1.11**	0.03338
Osbpl9	oxysterol binding protein-like 9 [Source:MGI Symbol;Acc:MGI:1923784]	2138.5	2299.1	**1.11**	0.03675
Hp1bp3	heterochromatin protein 1, binding protein 3 [Source:MGI Symbol;Acc:MGI:109369]	1666.1	1783.6	**1.10**	0.01826
Hoga1	4-hydroxy-2-oxoglutarate aldolase 1 [Source:MGI Symbol;Acc:MGI:1914682]	2801.7	3047.9	**1.10**	0.03269
Ctdsp1	CTD (carboxy-terminal domain, RNA polymerase II, polypeptide A) small phosphatase 1 [Source:MGI Symbol;Acc:MGI:2654470]	2538.7	2721.8	**1.10**	0.03329
Nr1h2	nuclear receptor subfamily 1, group H, member 2 [Source:MGI Symbol;Acc:MGI:1352463]	645.3	677.6	**1.10**	0.03648
Cldn12	claudin 12 [Source:MGI Symbol;Acc:MGI:1929288]	1508.0	1605.4	**1.09**	0.01202
Lamp1	lysosomal-associated membrane protein 1 [Source:MGI Symbol;Acc:MGI:96745]	11676.6	10428.6	**-1.11**	0.02848
Psme3	proteaseome (prosome, macropain) activator subunit 3 (PA28 gamma, Ki) [Source:MGI Symbol;Acc:MGI:1096366]	1008.3	898.0	**-1.12**	0.0307
Itgb5	integrin beta 5 [Source:MGI Symbol;Acc:MGI:96614]	1413.4	1256.8	**-1.12**	0.04506
Mpp6	membrane protein, palmitoylated 6 (MAGUK p55 subfamily member 6) [Source:MGI Symbol;Acc:MGI:1927340]	1459.4	1270.6	**-1.13**	0.01239
Rab1a	RAB1A, member RAS oncogene family [Source:MGI Symbol;Acc:MGI:97842]	2862.1	2501.6	**-1.13**	0.001892
Ddx1	DEAD (Asp-Glu-Ala-Asp) box polypeptide 1 [Source:MGI Symbol;Acc:MGI:2144727]	1361.9	1199.7	**-1.13**	0.004373
Def8	differentially expressed in FDCP 8 [Source:MGI Symbol;Acc:MGI:1346331]	997.1	868.4	**-1.13**	0.01146
Selenot	selenoprotein T [Source:MGI Symbol;Acc:MGI:1916477]	3024.4	2599.7	**-1.14**	5.42E-05
Rcbtb2	regulator of chromosome condensation (RCC1) and BTB (POZ) domain containing protein 2 [Source:MGI Symbol;Acc:MGI:1917200]	792.3	683.3	**-1.14**	0.001527
Calm1	calmodulin 1 [Source:MGI Symbol;Acc:MGI:88251]	3422.5	2967.4	**-1.14**	0.02928
Sh3bgrl	SH3-binding domain glutamic acid-rich protein like [Source:MGI Symbol;Acc:MGI:1930849]	2244.5	1920.9	**-1.14**	0.03838
Arf6	ADP-ribosylation factor 6 [Source:MGI Symbol;Acc:MGI:99435]	539.3	462.7	**-1.14**	0.04905
Ufm1	ubiquitin-fold modifier 1 [Source:MGI Symbol;Acc:MGI:1915140]	897.6	765.8	**-1.15**	0.0005673

Tprgl	transformation related protein 63 regulated like [Source:MGI Symbol;Acc:MGI:1915058]	1008.3	861.5	**-1.15**	0.0139
Anapc4	anaphase promoting complex subunit 4 [Source:MGI Symbol;Acc:MGI:1098673]	340.2	295.8	**-1.15**	0.03003
Rnpep	arginyl aminopeptidase (aminopeptidase B) [Source:MGI Symbol;Acc:MGI:2384902]	625.0	539.9	**-1.15**	0.03648
Ubxn4	UBX domain protein 4 [Source:MGI Symbol;Acc:MGI:1915062]	1759.0	1520.8	**-1.15**	0.04347
Mettl9	methyltransferase like 9 [Source:MGI Symbol;Acc:MGI:1914862]	942.3	811.9	**-1.15**	0.0464
Abhd5	abhydrolase domain containing 5 [Source:MGI Symbol;Acc:MGI:1914719]	592.5	507.7	**-1.16**	0.002153
Ccng1	cyclin G1 [Source:MGI Symbol;Acc:MGI:102890]	958.5	822.5	**-1.16**	0.00314
Dbnl	drebrin-like [Source:MGI Symbol;Acc:MGI:700006]	575.4	492.8	**-1.16**	0.01505
Ube2f	ubiquitin-conjugating enzyme E2F (putative) [Source:MGI Symbol;Acc:MGI:1915171]	523.0	435.0	**-1.16**	0.01523
Fam114a1	family with sequence similarity 114, member A1 [Source:MGI Symbol;Acc:MGI:1915553]	612.1	515.6	**-1.16**	0.01622
Opa3	optic atrophy 3 [Source:MGI Symbol;Acc:MGI:2686271]	962.9	821.6	**-1.16**	0.01771
Tmem167	transmembrane protein 167 [Source:MGI Symbol;Acc:MGI:1913324]	733.5	624.2	**-1.16**	0.0259
Tram1	translocating chain-associating membrane protein 1 [Source:MGI Symbol;Acc:MGI:1919515]	1920.5	1653.3	**-1.16**	0.02777
Psmd14	proteasome (prosome, macropain) 26S subunit, non-ATPase, 14 [Source:MGI Symbol;Acc:MGI:1913284]	937.6	805.5	**-1.16**	0.03172
Rnf220	ring finger protein 220 [Source:MGI Symbol;Acc:MGI:1913993]	362.5	307.5	**-1.16**	0.04116
Cdk11b	cyclin-dependent kinase 11B [Source:MGI Symbol;Acc:MGI:88353]	617.3	515.4	**-1.16**	0.03339
Mgat2	mannoside acetylglucosaminyltransferase 2 [Source:MGI Symbol;Acc:MGI:2384966]	794.4	664.4	**-1.16**	0.0464
Ehd1	EH-domain containing 1 [Source:MGI Symbol;Acc:MGI:1341878]	805.3	691.5	**-1.17**	0.002199
Yipf5	Yip1 domain family, member 5 [Source:MGI Symbol;Acc:MGI:1914430]	702.2	591.7	**-1.17**	0.003172
Pgam1	phosphoglycerate mutase 1 [Source:MGI Symbol;Acc:MGI:97552]	352.4	295.2	**-1.17**	0.01193
Fam107b	family with sequence similarity 107, member B [Source:MGI Symbol;Acc:MGI:1913790]	1597.4	1341.2	**-1.17**	0.02274
Mfsd5	major facilitator superfamily domain containing 5 [Source:MGI Symbol;Acc:MGI:2145901]	274.1	227.0	**-1.17**	0.0301
Rangap1	RAN GTPase activating protein 1 [Source:MGI Symbol;Acc:MGI:103071]	832.9	694.3	**-1.17**	0.03339
Cblc	Casitas B-lineage lymphoma c [Source:MGI Symbol;Acc:MGI:1931457]	373.6	315.9	**-1.17**	0.03422
Stx4a	syntaxin 4A (placental) [Source:MGI Symbol;Acc:MGI:893577]	630.9	521.5	**-1.17**	0.03518
Rab4a	RAB4A, member RAS oncogene family [Source:MGI Symbol;Acc:MGI:105069]	473.0	400.6	**-1.17**	0.03648
Psmd8	proteasome (prosome, macropain) 26S subunit, non-ATPase, 8 [Source:MGI Symbol;Acc:MGI:1888669]	2212.2	1870.3	**-1.17**	0.0402
Vat1	vesicle amine transport 1 [Source:MGI Symbol;Acc:MGI:1349450]	611.1	523.9	**-1.17**	0.04151
Snx12	sorting nexin 12 [Source:MGI Symbol;Acc:MGI:1919331]	193.8	165.0	**-1.17**	0.04223
Maf	avian musculoaponeurotic fibrosarcoma oncogene homolog [Source:MGI Symbol;Acc:MGI:96909]	727.2	582.0	**-1.17**	0.04779
Eif2d	eukaryotic translation initiation factor 2D [Source:MGI Symbol;Acc:MGI:109342]	613.0	508.6	**-1.18**	0.001189

Ppt1	palmitoyl-protein thioesterase 1 [Source:MGI Symbol;Acc:MGI:1298204]	484.4	402.4	**-1.18**	0.003327
Nudt4	nudix (nucleoside diphosphate linked moiety X)-type motif 4 [Source:MGI Symbol;Acc:MGI:1918457]	3948.0	3274.9	**-1.18**	0.007351
Cndp2	CNDP dipeptidase 2 (metallopeptidase M20 family) [Source:MGI Symbol;Acc:MGI:1913304]	822.2	696.9	**-1.18**	0.01202
Serp1	stress-associated endoplasmic reticulum protein 1 [Source:MGI Symbol;Acc:MGI:92638]	4585.5	3787.7	**-1.18**	0.01798
Slc25a5	solute carrier family 25 (mitochondrial carrier, adenine nucleotide translocator), member 5 [Source:MGI Symbol;Acc:MGI:1353496]	5945.8	4913.7	**-1.18**	0.02564
Ginm1	glycoprotein integral membrane 1 [Source:MGI Symbol;Acc:MGI:2384905]	605.3	504.1	**-1.18**	0.02853
Aldh1a7	aldehyde dehydrogenase family 1, subfamily A7 [Source:MGI Symbol;Acc:MGI:1347050]	4875.5	4175.1	**-1.18**	0.03748
Uso1	USO1 vesicle docking factor [Source:MGI Symbol;Acc:MGI:1929095]	1814.1	1532.6	**-1.18**	0.04116
Tmem8	transmembrane protein 8 [Source:MGI Symbol;Acc:MGI:1926283]	539.5	448.4	**-1.18**	0.04482
Tcta	T cell leukemia translocation altered gene [Source:MGI Symbol;Acc:MGI:1918829]	350.5	290.5	**-1.18**	0.04568
Mrpl16	mitochondrial ribosomal protein L16 [Source:MGI Symbol;Acc:MGI:2137219]	693.8	579.6	**-1.19**	0.00288
Glrx	glutaredoxin [Source:MGI Symbol;Acc:MGI:2135625]	432.1	350.1	**-1.19**	0.006081
Hsd17b12	hydroxysteroid (17-beta) dehydrogenase 12 [Source:MGI Symbol;Acc:MGI:1926967]	3518.8	2991.8	**-1.19**	0.007312
Wars	tryptophanyl-tRNA synthetase [Source:MGI Symbol;Acc:MGI:104630]	272.8	221.2	**-1.19**	0.0221
Aaas	achalasia, adrenocortical insufficiency, alacrimia [Source:MGI Symbol;Acc:MGI:2443767]	171.0	144.0	**-1.19**	0.02445
Igsf11	immunoglobulin superfamily, member 11 [Source:MGI Symbol;Acc:MGI:2388477]	482.6	411.6	**-1.19**	0.02755
Enpp2	ectonucleotide pyrophosphatase/phosphodiesterase 2 [Source:MGI Symbol;Acc:MGI:1321390]	2030.3	1648.8	**-1.19**	0.03104
Lyplal1	lysophospholipase-like 1 [Source:MGI Symbol;Acc:MGI:2385115]	293.9	241.9	**-1.19**	0.03235
Ndufa8	NADH:ubiquinone oxidoreductase subunit A8 [Source:MGI Symbol;Acc:MGI:1915625]	1617.0	1334.6	**-1.19**	0.03701
Ormdl3	ORM1-like 3 (S. cerevisiae) [Source:MGI Symbol;Acc:MGI:1913862]	1772.2	1463.7	**-1.19**	0.0465
Sec13	SEC13 homolog, nuclear pore and COPII coat complex component [Source:MGI Symbol;Acc:MGI:99832]	932.5	769.8	**-1.20**	0.0001472
Calm3	calmodulin 3 [Source:MGI Symbol;Acc:MGI:103249]	927.2	763.6	**-1.20**	0.002091
Smpd2	sphingomyelin phosphodiesterase 2, neutral [Source:MGI Symbol;Acc:MGI:1278330]	433.8	346.3	**-1.20**	0.003104
Stimate	STIM activating enhancer [Source:MGI Symbol;Acc:MGI:1921500]	271.3	227.9	**-1.20**	0.003359
Rexo2	RNA exonuclease 2 [Source:MGI Symbol;Acc:MGI:1888981]	1029.1	855.9	**-1.20**	0.008075
Rbms2	RNA binding motif, single stranded interacting protein 2 [Source:MGI Symbol;Acc:MGI:1861776]	199.0	165.2	**-1.20**	0.01263
Taldo1	transaldolase 1 [Source:MGI Symbol;Acc:MGI:1274789]	1950.9	1632.0	**-1.20**	0.01298
Gpr137	G protein-coupled receptor 137 [Source:MGI Symbol;Acc:MGI:2147529]	264.1	212.8	**-1.20**	0.01586
Smarca4	SWI/SNF related, matrix associated, actin dependent regulator of chromatin, subfamily a, member 4 [Source:MGI Symbol;Acc:MGI:88192]	961.6	790.3	**-1.20**	0.02256
Ctsa	cathepsin A [Source:MGI Symbol;Acc:MGI:97748]	2548.3	2088.7	**-1.20**	0.02527

Tmem177	transmembrane protein 177 [Source:MGI Symbol;Acc:MGI:1913593]	175.2	142.6	**-1.20**	0.04646
Polr3k	polymerase (RNA) III (DNA directed) polypeptide K [Source:MGI Symbol;Acc:MGI:1914255]	367.5	299.1	**-1.20**	0.04768
Ctsd	cathepsin D [Source:MGI Symbol;Acc:MGI:88562]	3452.0	2878.4	**-1.20**	0.04811
Cyp2j6	cytochrome P450, family 2, subfamily j, polypeptide 6 [Source:MGI Symbol;Acc:MGI:1270148]	1313.2	1056.3	**-1.21**	0.001573
Sec22b	SEC22 homolog B, vesicle trafficking protein [Source:MGI Symbol;Acc:MGI:1338759]	857.0	701.8	**-1.21**	0.005964
Gapdh	glyceraldehyde-3-phosphate dehydrogenase [Source:MGI Symbol;Acc:MGI:95640]	7093.4	5821.9	**-1.21**	0.02082
Exosc7	exosome component 7 [Source:MGI Symbol;Acc:MGI:1913696]	507.2	419.6	**-1.21**	0.02848
Hpcal1	hippocalcin-like 1 [Source:MGI Symbol;Acc:MGI:1855689]	211.5	172.8	**-1.21**	0.03302
Slc22a1	solute carrier family 22 (organic cation transporter), member 1 [Source:MGI Symbol;Acc:MGI:108111]	7227.5	6024.4	**-1.21**	0.03352
Ehbp1l1	EH domain binding protein 1-like 1 [Source:MGI Symbol;Acc:MGI:3612340]	134.4	110.3	**-1.21**	0.03893
Parp3	poly (ADP-ribose) polymerase family, member 3 [Source:MGI Symbol;Acc:MGI:1891258]	489.5	400.9	**-1.21**	0.04113
Apip	APAF1 interacting protein [Source:MGI Symbol;Acc:MGI:1926788]	312.3	253.8	**-1.21**	0.04497
Bet1	Bet1 golgi vesicular membrane trafficking protein [Source:MGI Symbol;Acc:MGI:1343104]	997.2	824.3	**-1.21**	0.04805
Dck	deoxycytidine kinase [Source:MGI Symbol;Acc:MGI:102726]	129.3	107.2	**-1.21**	0.04989
Ipo13	importin 13 [Source:MGI Symbol;Acc:MGI:2385205]	200.9	161.9	**-1.21**	0.04998
Dnajc10	DnaJ heat shock protein family (Hsp40) member C10 [Source:MGI Symbol;Acc:MGI:1914111]	516.7	418.9	**-1.21**	0.0005824
Tnfrsf1a	tumor necrosis factor receptor superfamily, member 1a [Source:MGI Symbol;Acc:MGI:1314884]	1645.9	1359.5	**-1.21**	0.002344
Wbp1	WW domain binding protein 1 [Source:MGI Symbol;Acc:MGI:104710]	575.1	465.5	**-1.21**	0.006077
Ltbr	lymphotoxin B receptor [Source:MGI Symbol;Acc:MGI:104875]	1996.1	1610.7	**-1.21**	0.013
Nudcd2	NudC domain containing 2 [Source:MGI Symbol;Acc:MGI:1277103]	527.2	422.1	**-1.21**	0.02323
Pdia6	protein disulfide isomerase associated 6 [Source:MGI Symbol;Acc:MGI:1919103]	3850.9	3184.3	**-1.21**	0.02502
Pdhb	pyruvate dehydrogenase (lipoamide) beta [Source:MGI Symbol;Acc:MGI:1915513]	2033.6	1657.0	**-1.21**	0.03086
Cd81	CD81 antigen [Source:MGI Symbol;Acc:MGI:1096398]	4073.3	3372.2	**-1.21**	0.03237
Hyi	hydroxypyruvate isomerase (putative) [Source:MGI Symbol;Acc:MGI:1915430]	1385.3	1106.4	**-1.21**	0.03263
Hspa13	heat shock protein 70 family, member 13 [Source:MGI Symbol;Acc:MGI:1309463]	527.5	425.7	**-1.21**	0.03592
Snx8	sorting nexin 8 [Source:MGI Symbol;Acc:MGI:2443816]	280.6	220.7	**-1.21**	0.03654
Tmem258	transmembrane protein 258 [Source:MGI Symbol;Acc:MGI:1916288]	501.7	392.1	**-1.21**	0.04863
Sae1	SUMO1 activating enzyme subunit 1 [Source:MGI Symbol;Acc:MGI:1929264]	388.6	312.3	**-1.22**	0.003172
Samm50	SAMM50 sorting and assembly machinery component [Source:MGI Symbol;Acc:MGI:1915903]	1747.3	1429.7	**-1.22**	0.003815
Tmem165	transmembrane protein 165 [Source:MGI Symbol;Acc:MGI:894407]	155.1	125.4	**-1.22**	0.01009
Cryzl2	crystallin zeta like 2 [Source:MGI Symbol;Acc:MGI:2448516]	465.9	373.8	**-1.22**	0.01263
Dcn	decorin [Source:MGI Symbol;Acc:MGI:94872]	2014.1	1652.4	**-1.22**	0.01624

Tank	TRAF family member-associated Nf-kappa B activator [Source:MGI Symbol;Acc:MGI:107676]	260.5	204.0	**-1.22**	0.01939
Cnp	2',3'-cyclic nucleotide 3' phosphodiesterase [Source:MGI Symbol;Acc:MGI:88437]	224.7	183.4	**-1.22**	0.02418
Oasl1	2'-5' oligoadenylate synthetase-like 1 [Source:MGI Symbol;Acc:MGI:2180849]	254.6	204.0	**-1.22**	0.02429
Galnt10	polypeptide N-acetylgalactosaminyltransferase 10 [Source:MGI Symbol;Acc:MGI:1890480]	126.3	101.1	**-1.22**	0.03368
Agpat2	1-acylglycerol-3-phosphate O-acyltransferase 2 (lysophosphatidic acid acyltransferase, beta) [Source:MGI Symbol;Acc:MGI:1914762]	4012.9	3202.3	**-1.23**	0.003172
Abcg2	ATP binding cassette subfamily G member 2 (Junior blood group) [Source:MGI Symbol;Acc:MGI:1347061]	1391.2	1123.3	**-1.23**	0.003691
Sptlc2	serine palmitoyltransferase, long chain base subunit 2 [Source:MGI Symbol;Acc:MGI:108074]	362.7	288.6	**-1.23**	0.003821
Uck1	uridine-cytidine kinase 1 [Source:MGI Symbol;Acc:MGI:98904]	723.9	587.8	**-1.23**	0.005543
Ttr	transthyretin [Source:MGI Symbol;Acc:MGI:98865]	129337.5	102039.7	**-1.23**	0.01839
Tmem192	transmembrane protein 192 [Source:MGI Symbol;Acc:MGI:1920317]	220.3	173.3	**-1.23**	0.02093
Dnajc9	DnaJ heat shock protein family (Hsp40) member C9 [Source:MGI Symbol;Acc:MGI:1915326]	108.7	82.9	**-1.23**	0.03471
Arpp19	cAMP-regulated phosphoprotein 19 [Source:MGI Symbol;Acc:MGI:1891691]	1218.5	968.4	**-1.23**	0.03667
Mppe1	metallophosphoesterase 1 [Source:MGI Symbol;Acc:MGI:2661311]	210.3	167.5	**-1.24**	0.001401
Arhgdia	Rho GDP dissociation inhibitor (GDI) alpha [Source:MGI Symbol;Acc:MGI:2178103]	1155.1	917.9	**-1.24**	0.01579
Rgs12	regulator of G-protein signaling 12 [Source:MGI Symbol;Acc:MGI:1918979]	88.1	67.7	**-1.24**	0.04089
Uba5	ubiquitin-like modifier activating enzyme 5 [Source:MGI Symbol;Acc:MGI:1913913]	522.5	413.1	**-1.25**	0.002091
Gusb	glucuronidase, beta [Source:MGI Symbol;Acc:MGI:95872]	473.7	379.2	**-1.25**	0.005265
Itm2c	integral membrane protein 2C [Source:MGI Symbol;Acc:MGI:1927594]	631.0	506.3	**-1.25**	0.007741
Tmed3	transmembrane p24 trafficking protein 3 [Source:MGI Symbol;Acc:MGI:1913361]	256.4	198.7	**-1.25**	0.008616
Cstb	cystatin B [Source:MGI Symbol;Acc:MGI:109514]	673.4	517.9	**-1.25**	0.02876
Pnpla5	patatin-like phospholipase domain containing 5 [Source:MGI Symbol;Acc:MGI:1923022]	31.5	1.3	**-1.25**	0.03304
Uchl3	ubiquitin carboxyl-terminal esterase L3 (ubiquitin thiolesterase) [Source:MGI Symbol;Acc:MGI:1355274]	278.6	214.5	**-1.25**	0.03375
Tnfsf12	tumor necrosis factor (ligand) superfamily, member 12 [Source:MGI Symbol;Acc:MGI:1196259]	115.2	89.5	**-1.25**	0.03879
Mfsd9	major facilitator superfamily domain containing 9 [Source:MGI Symbol;Acc:MGI:2443548]	71.4	57.2	**-1.25**	0.04177
Frmd8	FERM domain containing 8 [Source:MGI Symbol;Acc:MGI:1914707]	355.2	276.9	**-1.25**	0.04391
Tbc1d7	TBC1 domain family, member 7 [Source:MGI Symbol;Acc:MGI:1914296]	144.2	113.7	**-1.26**	0.00759
Fbxo6	F-box protein 6 [Source:MGI Symbol;Acc:MGI:1354743]	617.8	483.0	**-1.26**	0.01029
Tpm1	tropomyosin 1, alpha [Source:MGI Symbol;Acc:MGI:98809]	439.5	346.7	**-1.26**	0.01137
Hsd17b10	hydroxysteroid (17-beta) dehydrogenase 10 [Source:MGI Symbol;Acc:MGI:1333871]	3960.3	3064.6	**-1.26**	0.01579
Pyroxd2	pyridine nucleotide-disulphide oxidoreductase domain 2 [Source:MGI Symbol;Acc:MGI:1921830]	346.0	269.9	**-1.26**	0.01879

Tnip1	TNFAIP3 interacting protein 1 [Source:MGI Symbol;Acc:MGI:1926194]	167.1	130.8	**-1.26**	0.02225
Glrx5	glutaredoxin 5 [Source:MGI Symbol;Acc:MGI:1920296]	1953.6	1538.5	**-1.26**	0.03198
Ces2g	carboxylesterase 2G [Source:MGI Symbol;Acc:MGI:1919611]	872.2	689.8	**-1.26**	0.03471
Cotl1	coactosin-like 1 (Dictyostelium) [Source:MGI Symbol;Acc:MGI:1919292]	267.2	201.5	**-1.26**	0.038
Ucp2	uncoupling protein 2 (mitochondrial, proton carrier) [Source:MGI Symbol;Acc:MGI:109354]	533.9	422.6	**-1.26**	0.03879
Bax	BCL2-associated X protein [Source:MGI Symbol;Acc:MGI:99702]	231.3	174.5	**-1.26**	0.04321
Pspc1	paraspeckle protein 1 [Source:MGI Symbol;Acc:MGI:1913895]	152.0	118.5	**-1.26**	0.04407
Ces2e	carboxylesterase 2E [Source:MGI Symbol;Acc:MGI:2443170]	4375.6	3499.3	**-1.26**	0.04981
Rtn4ip1	reticulon 4 interacting protein 1 [Source:MGI Symbol;Acc:MGI:2178759]	491.8	386.8	**-1.27**	0.0001202
Cycs	cytochrome c, somatic [Source:MGI Symbol;Acc:MGI:88578]	557.3	428.6	**-1.27**	0.005194
Tmem43	transmembrane protein 43 [Source:MGI Symbol;Acc:MGI:1921372]	183.9	143.9	**-1.27**	0.0112
Rcan1	regulator of calcineurin 1 [Source:MGI Symbol;Acc:MGI:1890564]	345.7	264.9	**-1.27**	0.01405
Scamp5	secretory carrier membrane protein 5 [Source:MGI Symbol;Acc:MGI:1928948]	296.7	233.7	**-1.27**	0.0176
Plin3	perilipin 3 [Source:MGI Symbol;Acc:MGI:1914155]	933.0	717.1	**-1.27**	0.02091
Actb	actin, beta [Source:MGI Symbol;Acc:MGI:87904]	7210.3	5539.9	**-1.27**	0.02564
Omd	osteomodulin [Source:MGI Symbol;Acc:MGI:1350918]	67.5	50.2	**-1.27**	0.0307
Atox1	antioxidant 1 copper chaperone [Source:MGI Symbol;Acc:MGI:1333855]	1619.8	1236.3	**-1.27**	0.038
Tkt	transketolase [Source:MGI Symbol;Acc:MGI:105992]	2854.2	2235.4	**-1.27**	0.04905
Srxn1	sulfiredoxin 1 homolog (S. cerevisiae) [Source:MGI Symbol;Acc:MGI:104971]	1018.9	794.4	**-1.27**	0.007351
Pgrmc2	progesterone receptor membrane component 2 [Source:MGI Symbol;Acc:MGI:1918054]	1896.2	1490.3	**-1.27**	0.007591
Sdr39u1	short chain dehydrogenase/reductase family 39U, member 1 [Source:MGI Symbol;Acc:MGI:1916876]	220.5	173.7	**-1.27**	0.01128
Fam84b	family with sequence similarity 84, member B [Source:MGI Symbol;Acc:MGI:3026924]	168.3	128.5	**-1.27**	0.01128
A4gnt	alpha-1,4-N-acetylglucosaminyltransferase [Source:MGI Symbol;Acc:MGI:2143261]	20.7	2.8	**-1.27**	0.03879
Ndufb3	NADH:ubiquinone oxidoreductase subunit B3 [Source:MGI Symbol;Acc:MGI:1913745]	1112.5	837.4	**-1.27**	0.04347
Polr2k	polymerase (RNA) II (DNA directed) polypeptide K [Source:MGI Symbol;Acc:MGI:102725]	92.4	69.9	**-1.27**	0.04809
Gmppb	GDP-mannose pyrophosphorylase B [Source:MGI Symbol;Acc:MGI:2660880]	266.4	201.7	**-1.28**	0.004042
Lrrc59	leucine rich repeat containing 59 [Source:MGI Symbol;Acc:MGI:2138133]	1309.1	995.3	**-1.28**	0.006854
Slc25a40	solute carrier family 25, member 40 [Source:MGI Symbol;Acc:MGI:2442486]	76.2	56.2	**-1.28**	0.009534
Spp2	secreted phosphoprotein 2 [Source:MGI Symbol;Acc:MGI:1922646]	2176.3	1654.1	**-1.28**	0.01202
Nhp2	NHP2 ribonucleoprotein [Source:MGI Symbol;Acc:MGI:1098547]	399.0	300.4	**-1.28**	0.01841
Abracl	ABRA C-terminal like [Source:MGI Symbol;Acc:MGI:1920362]	174.1	129.9	**-1.28**	0.03104
Wwtr1	WW domain containing transcription regulator 1 [Source:MGI Symbol;Acc:MGI:1917649]	485.0	362.2	**-1.28**	0.03491

Spc25	SPC25, NDC80 kinetochore complex component, homolog (S. cerevisiae) [Source:MGI Symbol;Acc:MGI:1913692]	109.8	80.9	**-1.28**	0.03723
Slc22a15	solute carrier family 22 (organic anion/cation transporter), member 15 [Source:MGI Symbol;Acc:MGI:3607704]	354.1	274.0	**-1.29**	0.0007185
Sdhaf1	succinate dehydrogenase complex assembly factor 1 [Source:MGI Symbol;Acc:MGI:1915582]	280.0	205.8	**-1.29**	0.005265
Ebpl	emopamil binding protein-like [Source:MGI Symbol;Acc:MGI:1915427]	1924.7	1483.5	**-1.29**	0.009534
Cs	citrate synthase [Source:MGI Symbol;Acc:MGI:88529]	2831.7	2156.2	**-1.29**	0.01405
Plin2	perilipin 2 [Source:MGI Symbol;Acc:MGI:87920]	14325.5	10966.1	**-1.29**	0.02608
Gstp3	glutathione S-transferase pi 3 [Source:MGI Symbol;Acc:MGI:2385078]	406.8	300.7	**-1.29**	0.03471
Fuca2	fucosidase, alpha-L- 2, plasma [Source:MGI Symbol;Acc:MGI:1914098]	176.7	133.6	**-1.30**	0.001307
Rhoq	ras homolog family member Q [Source:MGI Symbol;Acc:MGI:1931553]	294.6	218.3	**-1.30**	0.00256
Shkbp1	Sh3kbp1 binding protein 1 [Source:MGI Symbol;Acc:MGI:2385803]	105.6	81.1	**-1.30**	0.004165
Mad2l1	MAD2 mitotic arrest deficient-like 1 [Source:MGI Symbol;Acc:MGI:1860374]	68.7	51.3	**-1.30**	0.007361
Nup93	nucleoporin 93 [Source:MGI Symbol;Acc:MGI:1919055]	93.7	69.5	**-1.30**	0.02199
Slc44a3	solute carrier family 44, member 3 [Source:MGI Symbol;Acc:MGI:2384860]	194.3	144.4	**-1.30**	0.02997
Nme1	NME/NM23 nucleoside diphosphate kinase 1 [Source:MGI Symbol;Acc:MGI:97355]	440.8	319.9	**-1.30**	0.03627
Endod1	endonuclease domain containing 1 [Source:MGI Symbol;Acc:MGI:1919196]	59.5	43.4	**-1.30**	0.03723
Manf	mesencephalic astrocyte-derived neurotrophic factor [Source:MGI Symbol;Acc:MGI:1922090]	994.3	712.7	**-1.30**	0.04401
Rogdi	rogdi homolog [Source:MGI Symbol;Acc:MGI:1913299]	244.5	186.0	**-1.31**	8.45E-05
Ctsz	cathepsin Z [Source:MGI Symbol;Acc:MGI:1891190]	3799.7	2821.7	**-1.31**	0.002316
Apcs	serum amyloid P-component [Source:MGI Symbol;Acc:MGI:98229]	1405.9	1088.8	**-1.31**	0.01135
Vac14	Vac14 homolog (S. cerevisiae) [Source:MGI Symbol;Acc:MGI:2157980]	446.2	335.2	**-1.31**	0.0161
Rnd2	Rho family GTPase 2 [Source:MGI Symbol;Acc:MGI:1338755]	197.9	151.7	**-1.31**	0.02871
Ppm1h	protein phosphatase 1H (PP2C domain containing) [Source:MGI Symbol;Acc:MGI:2442087]	66.3	46.1	**-1.31**	0.04184
Cd59b	CD59b antigen [Source:MGI Symbol;Acc:MGI:1888996]	64.0	46.0	**-1.31**	0.0473
Ugp2	UDP-glucose pyrophosphorylase 2 [Source:MGI Symbol;Acc:MGI:2183447]	11113.4	8055.0	**-1.32**	0.0001459
Fbxw9	F-box and WD-40 domain protein 9 [Source:MGI Symbol;Acc:MGI:1915878]	621.2	467.0	**-1.32**	0.0003718
Pgp	phosphoglycolate phosphatase [Source:MGI Symbol;Acc:MGI:1914328]	510.1	382.3	**-1.32**	0.0007675
Slc23a1	solute carrier family 23 (nucleobase transporters), member 1 [Source:MGI Symbol;Acc:MGI:1341903]	1367.2	1024.2	**-1.32**	0.004167
Gpd2	glycerol phosphate dehydrogenase 2, mitochondrial [Source:MGI Symbol;Acc:MGI:99778]	1000.1	746.6	**-1.32**	0.004971
Gyg	glycogenin [Source:MGI Symbol;Acc:MGI:1351614]	127.4	95.1	**-1.32**	0.005265
Gck	glucokinase [Source:MGI Symbol;Acc:MGI:1270854]	3380.2	2354.6	**-1.32**	0.01283
Stat5a	signal transducer and activator of transcription 5A [Source:MGI Symbol;Acc:MGI:103036]	232.4	167.6	**-1.32**	0.01911

Cyp4a12a	cytochrome P450, family 4, subfamily a, polypeptide 12a [Source:MGI Symbol;Acc:MGI:88612]	2032.1	1587.9	**-1.32**	0.02654
Slc13a3	solute carrier family 13 (sodium-dependent dicarboxylate transporter), member 3 [Source:MGI Symbol;Acc:MGI:2149635]	242.0	172.5	**-1.32**	0.04116
Olig1	oligodendrocyte transcription factor 1 [Source:MGI Symbol;Acc:MGI:1355334]	315.4	230.7	**-1.32**	0.04646
Synj2	synaptojanin 2 [Source:MGI Symbol;Acc:MGI:1201671]	161.4	118.5	**-1.32**	0.04772
Arl8a	ADP-ribosylation factor-like 8A [Source:MGI Symbol;Acc:MGI:1915974]	301.7	220.6	**-1.33**	0.0008354
Cebpe	CCAAT/enhancer binding protein (C/EBP), epsilon [Source:MGI Symbol;Acc:MGI:103572]	210.9	150.2	**-1.33**	0.005083
Dnph1	2'-deoxynucleoside 5'-phosphate N-hydrolase 1 [Source:MGI Symbol;Acc:MGI:3039376]	154.4	107.3	**-1.33**	0.005782
Ikbke	inhibitor of kappaB kinase epsilon [Source:MGI Symbol;Acc:MGI:1929612]	260.2	187.1	**-1.33**	0.007626
Alyref2	Aly/REF export factor 2 [Source:MGI Symbol;Acc:MGI:1913144]	64.4	48.2	**-1.33**	0.01048
Ufsp1	UFM1-specific peptidase 1 [Source:MGI Symbol;Acc:MGI:1917490]	50.7	35.9	**-1.33**	0.01523
Gpi1	glucose phosphate isomerase 1 [Source:MGI Symbol;Acc:MGI:95797]	2670.4	1941.2	**-1.33**	0.02146
Cln6	ceroid-lipofuscinosis, neuronal 6 [Source:MGI Symbol;Acc:MGI:2159324]	93.7	63.7	**-1.33**	0.03086
Comtd1	catechol-O-methyltransferase domain containing 1 [Source:MGI Symbol;Acc:MGI:1916406]	83.5	59.3	**-1.33**	0.03382
Slc22a3	solute carrier family 22 (organic cation transporter), member 3 [Source:MGI Symbol;Acc:MGI:1333817]	117.6	81.3	**-1.33**	0.03749
Nek6	NIMA (never in mitosis gene a)-related expressed kinase 6 [Source:MGI Symbol;Acc:MGI:1891638]	1031.1	750.5	**-1.34**	0.0001397
Snrpn	small nuclear ribonucleoprotein N [Source:MGI Symbol;Acc:MGI:98347]	104.2	76.1	**-1.34**	0.0003749
Gga2	golgi associated, gamma adaptin ear containing, ARF binding protein 2 [Source:MGI Symbol;Acc:MGI:1921355]	286.0	211.3	**-1.34**	0.0004816
Hexa	hexosaminidase A [Source:MGI Symbol;Acc:MGI:96073]	1128.1	832.2	**-1.34**	0.001313
Swsap1	SWIM type zinc finger 7 associated protein 1 [Source:MGI Symbol;Acc:MGI:1914212]	97.4	71.8	**-1.34**	0.001834
Bco1	beta-carotene oxygenase 1 [Source:MGI Symbol;Acc:MGI:1926923]	115.7	80.4	**-1.34**	0.01029
Csf2rb	colony stimulating factor 2 receptor, beta, low-affinity (granulocyte-macrophage) [Source:MGI Symbol;Acc:MGI:1339759]	104.0	73.5	**-1.34**	0.01096
Macrod2	MACRO domain containing 2 [Source:MGI Symbol;Acc:MGI:1920149]	66.7	47.1	**-1.34**	0.01104
Pnpla3	patatin-like phospholipase domain containing 3 [Source:MGI Symbol;Acc:MGI:2151796]	28.1	3.7	**-1.34**	0.01798
F2r	coagulation factor II (thrombin) receptor [Source:MGI Symbol;Acc:MGI:101802]	472.5	336.1	**-1.34**	0.01929
Lgals3	lectin, galactose binding, soluble 3 [Source:MGI Symbol;Acc:MGI:96778]	92.0	62.3	**-1.34**	0.03637
Anxa1	annexin A1 [Source:MGI Symbol;Acc:MGI:96819]	49.9	35.1	**-1.34**	0.03764
Tbc1d31	TBC1 domain family, member 31 [Source:MGI Symbol;Acc:MGI:2684931]	147.9	102.3	**-1.34**	0.04251
Sult1c2	sulfotransferase family, cytosolic, 1C, member 2 [Source:MGI Symbol;Acc:MGI:1916333]	216.3	151.6	**-1.35**	0.002042
Mrps6	mitochondrial ribosomal protein S6 [Source:MGI Symbol;Acc:MGI:2153111]	122.0	87.1	**-1.35**	0.002705
Plscr1	phospholipid scramblase 1 [Source:MGI Symbol;Acc:MGI:893575]	202.7	146.2	**-1.36**	0.003172

Spata24	spermatogenesis associated 24 [Source:MGI Symbol;Acc:MGI:1918492]	57.4	41.5	**-1.36**	0.01939
Bik	BCL2-interacting killer [Source:MGI Symbol;Acc:MGI:1206591]	84.0	54.7	**-1.36**	0.03519
Slc50a1	solute carrier family 50 (sugar transporter), member 1 [Source:MGI Symbol;Acc:MGI:107417]	116.1	80.8	**-1.37**	0.0007586
Slc17a9	solute carrier family 17, member 9 [Source:MGI Symbol;Acc:MGI:1919107]	60.2	42.8	**-1.37**	0.004385
Plekha1	pleckstrin homology domain containing, family A (phosphoinositide binding specific) member 1 [Source:MGI Symbol;Acc:MGI:2442213]	241.6	171.6	**-1.37**	0.00507
Car2	carbonic anhydrase 2 [Source:MGI Symbol;Acc:MGI:88269]	84.6	59.2	**-1.37**	0.009214
Castor1	cytosolic arginine sensor for mTORC1 subunit 1 [Source:MGI Symbol;Acc:MGI:1919212]	36.8	24.8	**-1.37**	0.01799
Tkfc	triokinase, FMN cyclase [Source:MGI Symbol;Acc:MGI:2385084]	7512.8	5138.1	**-1.37**	0.03331
Wnt4	wingless-type MMTV integration site family, member 4 [Source:MGI Symbol;Acc:MGI:98957]	35.9	24.2	**-1.37**	0.03701
Slc35b1	solute carrier family 35, member B1 [Source:MGI Symbol;Acc:MGI:1343133]	701.3	498.7	**-1.38**	0.0008804
Anxa5	annexin A5 [Source:MGI Symbol;Acc:MGI:106008]	1614.3	1138.1	**-1.38**	0.003733
Twf2	twinfilin actin binding protein 2 [Source:MGI Symbol;Acc:MGI:1346078]	48.7	33.6	**-1.38**	0.013
Gas6	growth arrest specific 6 [Source:MGI Symbol;Acc:MGI:95660]	645.9	450.5	**-1.38**	0.01523
Ppp1r1b	protein phosphatase 1, regulatory inhibitor subunit 1B [Source:MGI Symbol;Acc:MGI:94860]	78.3	53.7	**-1.38**	0.01557
Sccpdh	saccharopine dehydrogenase (putative) [Source:MGI Symbol;Acc:MGI:1924486]	45.4	32.0	**-1.38**	0.02155
Crygn	crystallin, gamma N [Source:MGI Symbol;Acc:MGI:2449167]	30.5	20.4	**-1.38**	0.03274
Trhde	TRH-degrading enzyme [Source:MGI Symbol;Acc:MGI:2384311]	42.1	26.8	**-1.38**	0.03654
Ifi27l2a	interferon, alpha-inducible protein 27 like 2A [Source:MGI Symbol;Acc:MGI:1924183]	80.8	51.0	**-1.38**	0.03741
Zmat3	zinc finger matrin type 3 [Source:MGI Symbol;Acc:MGI:1195270]	124.6	84.9	**-1.39**	0.000135
Cyp2d40	cytochrome P450, family 2, subfamily d, polypeptide 40 [Source:MGI Symbol;Acc:MGI:1919004]	1305.0	908.2	**-1.39**	0.001125
Ggct	gamma-glutamyl cyclotransferase [Source:MGI Symbol;Acc:MGI:95700]	163.2	113.3	**-1.39**	0.004381
Clstn3	calsyntenin 3 [Source:MGI Symbol;Acc:MGI:2178323]	673.1	484.2	**-1.39**	0.01393
Bmp4	bone morphogenetic protein 4 [Source:MGI Symbol;Acc:MGI:88180]	35.0	23.0	**-1.39**	0.03893
Prss23	protease, serine 23 [Source:MGI Symbol;Acc:MGI:1923703]	68.5	45.1	**-1.39**	0.04788
Ripply1	ripply transcriptional repressor 1 [Source:MGI Symbol;Acc:MGI:3614797]	16.2	9.5	**-1.39**	0.04826
Khk	ketohexokinase [Source:MGI Symbol;Acc:MGI:1096353]	10723.7	7540.3	**-1.39**	0.0003746
Ptp4a3	protein tyrosine phosphatase 4a3 [Source:MGI Symbol;Acc:MGI:1277098]	113.1	79.1	**-1.39**	0.002623
Entpd2	ectonucleoside triphosphate diphosphohydrolase 2 [Source:MGI Symbol;Acc:MGI:1096863]	53.1	35.5	**-1.39**	0.01128
Serinc2	serine incorporator 2 [Source:MGI Symbol;Acc:MGI:1919132]	186.6	135.6	**-1.39**	0.01186
Rec8	REC8 meiotic recombination protein [Source:MGI Symbol;Acc:MGI:1929645]	23.8	14.3	**-1.39**	0.04113
Gngt1	guanine nucleotide binding protein (G protein), gamma transducing activity polypeptide 1 [Source:MGI Symbol;Acc:MGI:109165]	32.7	20.5	**-1.39**	0.0464

Cyp1a1	cytochrome P450, family 1, subfamily a, polypeptide 1 [Source:MGI Symbol;Acc:MGI:88588]	14.1	7.3	**-1.39**	0.04761
Ttbk1	tau tubulin kinase 1 [Source:MGI Symbol;Acc:MGI:2147036]	24.7	12.0	**-1.39**	0.04986
Lrrc28	leucine rich repeat containing 28 [Source:MGI Symbol;Acc:MGI:1915689]	427.1	295.8	**-1.40**	6.42E-05
Rps6ka1	ribosomal protein S6 kinase polypeptide 1 [Source:MGI Symbol;Acc:MGI:104558]	376.2	271.1	**-1.40**	0.0008082
Trim7	tripartite motif-containing 7 [Source:MGI Symbol;Acc:MGI:2137353]	215.1	141.3	**-1.40**	0.002513
Vcpkmt	valosin containing protein lysine (K) methyltransferase [Source:MGI Symbol;Acc:MGI:2684917]	40.4	26.8	**-1.40**	0.007797
Cd36	CD36 molecule [Source:MGI Symbol;Acc:MGI:107899]	2079.6	1395.7	**-1.40**	0.01047
Mblac1	metallo-beta-lactamase domain containing 1 [Source:MGI Symbol;Acc:MGI:2679717]	26.0	16.3	**-1.40**	0.02079
Limk1	LIM-domain containing, protein kinase [Source:MGI Symbol;Acc:MGI:104572]	58.3	37.3	**-1.40**	0.02368
Ralgps1	Ral GEF with PH domain and SH3 binding motif 1 [Source:MGI Symbol;Acc:MGI:1922008]	95.0	59.3	**-1.40**	0.02495
Tuba1c	tubulin, alpha 1C [Source:MGI Symbol;Acc:MGI:1095409]	389.9	242.4	**-1.40**	0.03269
Plcxd1	phosphatidylinositol-specific phospholipase C, X domain containing 1 [Source:MGI Symbol;Acc:MGI:2685422]	32.9	19.9	**-1.40**	0.03746
Mup9	major urinary protein 9 [Source:MGI Symbol;Acc:MGI:3782918]	171.4	75.5	**-1.40**	0.0385
Cntnap1	contactin associated protein-like 1 [Source:MGI Symbol;Acc:MGI:1858201]	69.9	40.0	**-1.40**	0.04355
Ncmap	noncompact myelin associated protein [Source:MGI Symbol;Acc:MGI:2444888]	20.7	11.8	**-1.40**	0.04407
Plcd3	phospholipase C, delta 3 [Source:MGI Symbol;Acc:MGI:107451]	14.5	7.6	**-1.40**	0.0464
Col3a1	collagen, type III, alpha 1 [Source:MGI Symbol;Acc:MGI:88453]	540.9	318.3	**-1.40**	0.04646
Gypc	glycophorin C [Source:MGI Symbol;Acc:MGI:1098566]	213.3	148.2	**-1.41**	1.22E-06
Slc17a4	solute carrier family 17 (sodium phosphate), member 4 [Source:MGI Symbol;Acc:MGI:2442850]	1009.1	667.6	**-1.41**	0.003894
Lrrc14b	leucine rich repeat containing 14B [Source:MGI Symbol;Acc:MGI:2145269]	20.4	11.4	**-1.41**	0.03471
Ighm	immunoglobulin heavy constant mu [Source:MGI Symbol;Acc:MGI:96448]	224.3	113.5	**-1.41**	0.03648
Gna14	guanine nucleotide binding protein, alpha 14 [Source:MGI Symbol;Acc:MGI:95769]	59.3	31.4	**-1.41**	0.03654
Zfp423	zinc finger protein 423 [Source:MGI Symbol;Acc:MGI:1891217]	13.5	6.9	**-1.41**	0.03668
Igf2bp2	insulin-like growth factor 2 mRNA binding protein 2 [Source:MGI Symbol;Acc:MGI:1890358]	15.9	8.6	**-1.41**	0.03668
Hhipl1	hedgehog interacting protein-like 1 [Source:MGI Symbol;Acc:MGI:1919265]	40.5	23.1	**-1.41**	0.03764
Ubd	ubiquitin D [Source:MGI Symbol;Acc:MGI:1344410]	41.1	20.1	**-1.41**	0.038
Nans	N-acetylneuraminic acid synthase (sialic acid synthase) [Source:MGI Symbol;Acc:MGI:2149820]	284.8	200.6	**-1.42**	2.94E-07
Reep5	receptor accessory protein 5 [Source:MGI Symbol;Acc:MGI:1270152]	302.7	208.1	**-1.42**	0.0004816
Pik3r3	phosphoinositide-3-kinase regulatory subunit 3 [Source:MGI Symbol;Acc:MGI:109277]	27.6	15.9	**-1.42**	0.01505
Gsdme	gasdermin E [Source:MGI Symbol;Acc:MGI:1889850]	25.7	15.4	**-1.42**	0.01565
Capg	capping protein (actin filament), gelsolin-like [Source:MGI Symbol;Acc:MGI:1098259]	31.3	18.7	**-1.42**	0.02608
Lrrc39	leucine rich repeat containing 39 [Source:MGI Symbol;Acc:MGI:1924557]	50.9	29.9	**-1.42**	0.03339

Mrgprb11-ps	MAS-related GPR, member B11, pseudogene [Source:MGI Symbol;Acc:MGI:3033189]	18.4	9.4	**-1.42**	0.0345
Mup21	major urinary protein 21 [Source:MGI Symbol;Acc:MGI:3650630]	2879.9	1713.6	**-1.43**	0.004042
Mtnr1a	melatonin receptor 1A [Source:MGI Symbol;Acc:MGI:102967]	55.3	35.3	**-1.43**	0.005597
Ms4a4d	membrane-spanning 4-domains, subfamily A, member 4D [Source:MGI Symbol;Acc:MGI:1913857]	52.0	32.3	**-1.43**	0.006037
Mup6	major urinary protein 6 [Source:MGI Symbol;Acc:MGI:3650962]	54.7	33.9	**-1.43**	0.01128
Zfp651	zinc finger protein 651 [Source:MGI Symbol;Acc:MGI:2670992]	24.0	14.8	**-1.43**	0.01476
Ccdc3	coiled-coil domain containing 3 [Source:MGI Symbol;Acc:MGI:1921436]	35.0	22.7	**-1.43**	0.01907
Pik3c2g	phosphatidylinositol-4-phosphate 3-kinase catalytic subunit type 2 gamma [Source:MGI Symbol;Acc:MGI:1203730]	117.8	69.4	**-1.43**	0.01939
Ttc39a	tetratricopeptide repeat domain 39A [Source:MGI Symbol;Acc:MGI:2444350]	29.1	10.7	**-1.43**	0.02091
Ocstamp	osteoclast stimulatory transmembrane protein [Source:MGI Symbol;Acc:MGI:1921864]	17.6	10.5	**-1.43**	0.02322
Nupr1	nuclear protein transcription regulator 1 [Source:MGI Symbol;Acc:MGI:1891834]	28.8	12.8	**-1.43**	0.0307
Mirt2	myocardial infraction associated transcript 2 [Source:MGI Symbol;Acc:MGI:3642813]	13.7	6.9	**-1.43**	0.03235
Tubb4b	tubulin, beta 4B class IVB [Source:MGI Symbol;Acc:MGI:1915472]	1205.4	780.6	**-1.44**	0.005953
Anxa2	annexin A2 [Source:MGI Symbol;Acc:MGI:88246]	305.8	184.0	**-1.44**	0.01758
Col1a1	collagen, type I, alpha 1 [Source:MGI Symbol;Acc:MGI:88467]	140.6	61.8	**-1.44**	0.01798
Mycn	v-myc avian myelocytomatosis viral related oncogene, neuroblastoma derived [Source:MGI Symbol;Acc:MGI:97357]	33.2	15.7	**-1.44**	0.02091
Themis	thymocyte selection associated [Source:MGI Symbol;Acc:MGI:2443552]	185.7	90.1	**-1.44**	0.02728
Orm1	orosomucoid 1 [Source:MGI Symbol;Acc:MGI:97443]	5093.9	3579.2	**-1.45**	1.54E-06
Rasgrp2	RAS, guanyl releasing protein 2 [Source:MGI Symbol;Acc:MGI:1333849]	222.5	139.2	**-1.45**	0.001892
Notumos	notum palmitoleoyl-protein carboxylesterase, opposite strand [Source:MGI Symbol;Acc:MGI:3698431]	37.6	23.0	**-1.45**	0.002344
Dio1	deiodinase, iodothyronine, type I [Source:MGI Symbol;Acc:MGI:94896]	3158.8	2030.7	**-1.45**	0.002397
Mr1	major histocompatibility complex, class I-related [Source:MGI Symbol;Acc:MGI:1195463]	129.1	81.3	**-1.45**	0.004101
Lgals1	lectin, galactose binding, soluble 1 [Source:MGI Symbol;Acc:MGI:96777]	830.0	469.1	**-1.45**	0.02179
Pdzk1ip1	PDZK1 interacting protein 1 [Source:MGI Symbol;Acc:MGI:1914432]	62.7	29.9	**-1.45**	0.02325
Pgd	phosphogluconate dehydrogenase [Source:MGI Symbol;Acc:MGI:97553]	973.7	594.1	**-1.46**	0.007134
Pdzrn3	PDZ domain containing RING finger 3 [Source:MGI Symbol;Acc:MGI:1933157]	112.9	65.7	**-1.46**	0.008675
Gask1a	golgi associated kinase 1A [Source:MGI Symbol;Acc:MGI:3041196]	104.4	43.9	**-1.46**	0.01828
Chrna4	cholinergic receptor, nicotinic, alpha polypeptide 4 [Source:MGI Symbol;Acc:MGI:87888]	88.7	19.3	**-1.47**	0.004406
Dct	dopachrome tautomerase [Source:MGI Symbol;Acc:MGI:102563]	74.0	43.6	**-1.47**	0.005724
Ctps	cytidine 5'-triphosphate synthase [Source:MGI Symbol;Acc:MGI:1858304]	105.1	65.8	**-1.48**	0.0009825
Fkbp7	FK506 binding protein 7 [Source:MGI Symbol;Acc:MGI:1336879]	52.9	32.7	**-1.49**	0.001125

Haus8	4HAUS augmin-like complex, subunit 8 [Source:MGI Symbol;Acc:MGI:1923728]	111.2	65.9	**-1.49**	0.002853
Insig2	insulin induced gene 2 [Source:MGI Symbol;Acc:MGI:1920249]	3701.9	2078.3	**-1.49**	0.006534
Them6	thioesterase superfamily member 6 [Source:MGI Symbol;Acc:MGI:1925301]	96.6	62.4	**-1.51**	2.77E-05
Col1a2	collagen, type I, alpha 2 [Source:MGI Symbol;Acc:MGI:88468]	238.1	137.7	**-1.51**	0.007797
Tagln	transgelin [Source:MGI Symbol;Acc:MGI:106012]	84.5	49.9	**-1.52**	0.002153
Tubb2a	tubulin, beta 2A class IIA [Source:MGI Symbol;Acc:MGI:107861]	960.3	274.6	**-1.52**	0.003821
Orm3	orosomucoid 3 [Source:MGI Symbol;Acc:MGI:97445]	22.9	8.5	**-1.52**	0.007797
Scara5	scavenger receptor class A, member 5 [Source:MGI Symbol;Acc:MGI:1918395]	45.6	16.8	**-1.52**	0.008644
Fam124a	family with sequence similarity 124, member A [Source:MGI Symbol;Acc:MGI:3645930]	188.1	114.8	**-1.53**	4.34E-05
Rragd	Ras-related GTP binding D [Source:MGI Symbol;Acc:MGI:1098604]	36.0	20.7	**-1.53**	0.0008025
Hapln4	hyaluronan and proteoglycan link protein 4 [Source:MGI Symbol;Acc:MGI:2679531]	109.6	61.3	**-1.53**	0.00192
Mup-ps20	major urinary protein, pseudogene 20 [Source:MGI Symbol;Acc:MGI:3651976]	45.9	8.6	**-1.53**	0.002344
Slc25a35	solute carrier family 25, member 35 [Source:MGI Symbol;Acc:MGI:1919248]	21.9	11.2	**-1.53**	0.003017
Cpxm1	carboxypeptidase X 1 (M14 family) [Source:MGI Symbol;Acc:MGI:1934569]	21.6	10.8	**-1.53**	0.006322
Cgref1	cell growth regulator with EF hand domain 1 [Source:MGI Symbol;Acc:MGI:1915817]	38.9	16.5	**-1.53**	0.006797
Fam241b	family with sequence similarity 241, member B [Source:MGI Symbol;Acc:MGI:1917144]	73.3	44.3	**-1.54**	0.0001991
Tubb6	tubulin, beta 6 class V [Source:MGI Symbol;Acc:MGI:1915201]	85.6	44.7	**-1.54**	0.003254
Slc6a8	solute carrier family 6 (neurotransmitter transporter, creatine), member 8 [Source:MGI Symbol;Acc:MGI:2147834]	99.2	56.9	**-1.55**	0.0003208
Dusp6	dual specificity phosphatase 6 [Source:MGI Symbol;Acc:MGI:1914853]	692.8	367.2	**-1.55**	0.001212
Raet1e	retinoic acid early transcript 1E [Source:MGI Symbol;Acc:MGI:2675273]	66.7	41.4	**-1.55**	0.002853
Plin4	perilipin 4 [Source:MGI Symbol;Acc:MGI:1929709]	218.7	105.4	**-1.55**	0.003033
Pdlim2	PDZ and LIM domain 2 [Source:MGI Symbol;Acc:MGI:2384850]	37.3	20.0	**-1.56**	0.00167
Anxa8	annexin A8 [Source:MGI Symbol;Acc:MGI:1201374]	15.6	5.5	**-1.56**	0.003116
Pnldc1	poly(A)-specific ribonuclease (PARN)-like domain containing 1 [Source:MGI Symbol;Acc:MGI:2685159]	235.1	113.3	**-1.56**	0.003691
Gale	galactose-4-epimerase, UDP [Source:MGI Symbol;Acc:MGI:1921496]	416.7	215.9	**-1.57**	0.00202
Slc16a11	solute carrier family 16 (monocarboxylic acid transporters), member 11 [Source:MGI Symbol;Acc:MGI:2663709]	269.8	140.6	**-1.57**	0.002162
Mtmr11	myotubularin related protein 11 [Source:MGI Symbol;Acc:MGI:2652817]	24.4	7.2	**-1.57**	0.002193
Nhlrc1	NHL repeat containing 1 [Source:MGI Symbol;Acc:MGI:2145264]	35.4	20.7	**-1.59**	0.0001333
Pls1	plastin 1 (I-isoform) [Source:MGI Symbol;Acc:MGI:104809]	31.3	9.9	**-1.59**	0.001386
Cd63	CD63 antigen [Source:MGI Symbol;Acc:MGI:99529]	75.7	38.0	**-1.60**	0.001139
Pklr	pyruvate kinase liver and red blood cell [Source:MGI Symbol;Acc:MGI:97604]	8379.8	3927.1	**-1.60**	0.001434

C4a	complement component 4A (Rodgers blood group) [Source:MGI Symbol;Acc:MGI:98320]	159.0	85.6	**-1.61**	2.96E-05
Mypop	Myb-related transcription factor, partner of profilin [Source:MGI Symbol;Acc:MGI:2446472]	26.5	12.8	**-1.61**	0.000281
Acot11	acyl-CoA thioesterase 11 [Source:MGI Symbol;Acc:MGI:1913736]	177.5	76.4	**-1.61**	0.001217
Col12a1	collagen, type XII, alpha 1 [Source:MGI Symbol;Acc:MGI:88448]	37.6	20.9	**-1.64**	4.20E-05
Cdh18	cadherin 18 [Source:MGI Symbol;Acc:MGI:1344366]	32.3	15.6	**-1.65**	8.45E-05
Slc16a13	solute carrier family 16 (monocarboxylic acid transporters), member 13 [Source:MGI Symbol;Acc:MGI:1916559]	32.7	15.0	**-1.65**	0.0005186
S100a10	S100 calcium binding protein A10 (calpactin) [Source:MGI Symbol;Acc:MGI:1339468]	2037.6	1116.5	**-1.66**	5.53E-07
Acnat2	acyl-coenzyme A amino acid N-acyltransferase 2 [Source:MGI Symbol;Acc:MGI:2444345]	187.5	92.4	**-1.66**	4.76E-06
Fam110a	family with sequence similarity 110, member A [Source:MGI Symbol;Acc:MGI:1921097]	68.2	36.8	**-1.68**	5.50E-07
Cdkn1a	cyclin-dependent kinase inhibitor 1A (P21) [Source:MGI Symbol;Acc:MGI:104556]	109.0	49.8	**-1.69**	6.97E-05
Uap1l1	UDP-N-acteylglucosamine pyrophosphorylase 1-like 1 [Source:MGI Symbol;Acc:MGI:2443318]	138.2	65.3	**-1.69**	0.0001397
Mogat1	monoacylglycerol O-acyltransferase 1 [Source:MGI Symbol;Acc:MGI:1915643]	67.7	27.1	**-1.69**	0.0002708
Orm2	orosomucoid 2 [Source:MGI Symbol;Acc:MGI:97444]	127.6	64.3	**-1.73**	6.72E-05
Tmem28	transmembrane protein 28 [Source:MGI Symbol;Acc:MGI:3648377]	78.1	28.5	**-1.73**	0.0001305
Tceal8	transcription elongation factor A (SII)-like 8 [Source:MGI Symbol;Acc:MGI:1913934]	529.0	275.7	**-1.74**	8.04E-08
Slc16a6	solute carrier family 16 (monocarboxylic acid transporters), member 6 [Source:MGI Symbol;Acc:MGI:2144585]	79.9	36.6	**-1.74**	1.26E-05
Cxcl14	chemokine (C-X-C motif) ligand 14 [Source:MGI Symbol;Acc:MGI:1888514]	25.0	8.4	**-1.74**	0.0001079
Saa1	serum amyloid A 1 [Source:MGI Symbol;Acc:MGI:98221]	1277.5	471.0	**-1.75**	7.88E-05
Tsc22d1	TSC22 domain family, member 1 [Source:MGI Symbol;Acc:MGI:109127]	1940.3	947.4	**-1.77**	2.94E-07
Extl1	exostoses (multiple)-like 1 [Source:MGI Symbol;Acc:MGI:1888742]	324.5	153.5	**-1.77**	1.44E-06
Phlda3	pleckstrin homology like domain, family A, member 3 [Source:MGI Symbol;Acc:MGI:1351485]	35.0	13.8	**-1.78**	2.61E-05
Ly6d	lymphocyte antigen 6 complex, locus D [Source:MGI Symbol;Acc:MGI:96881]	45.0	11.1	**-1.80**	2.69E-05
Aqp8	aquaporin 8 [Source:MGI Symbol;Acc:MGI:1195271]	5539.7	2377.6	**-1.82**	2.35E-06
Cyp46a1	cytochrome P450, family 46, subfamily a, polypeptide 1 [Source:MGI Symbol;Acc:MGI:1341877]	92.2	32.3	**-1.82**	2.40E-05
Bmyc	brain expressed myelocytomatosis oncogene [Source:MGI Symbol;Acc:MGI:88184]	89.6	38.7	**-1.83**	1.27E-06
Smpd3	sphingomyelin phosphodiesterase 3, neutral [Source:MGI Symbol;Acc:MGI:1927578]	106.8	36.2	**-1.83**	1.56E-05
Saa2	serum amyloid A 2 [Source:MGI Symbol;Acc:MGI:98222]	762.9	222.7	**-1.83**	2.03E-05
Fam129b	family with sequence similarity 129, member B [Source:MGI Symbol;Acc:MGI:2442910]	304.6	130.0	**-1.87**	2.43E-07
Morc4	microrchidia 4 [Source:MGI Symbol;Acc:MGI:1922996]	72.5	30.8	**-1.95**	1.81E-09
Rarres1	retinoic acid receptor responder (tazarotene induced) 1 [Source:MGI Symbol;Acc:MGI:1924461]	1168.6	425.4	**-1.95**	2.67E-07
Lcn2	lipocalin 2 [Source:MGI Symbol;Acc:MGI:96757]	174.8	42.2	**-1.99**	4.71E-07

Rad51b	RAD51 paralog B [Source:MGI Symbol;Acc:MGI:1099436]	90.9	23.3	**-2.03**	2.34E-07
Gpc1	glypican 1 [Source:MGI Symbol;Acc:MGI:1194891]	542.7	211.2	**-2.04**	3.53E-10
Slc35f2	solute carrier family 35, member F2 [Source:MGI Symbol;Acc:MGI:1919272]	34.4	10.5	**-2.04**	7.57E-08
Ifi27l2b	interferon, alpha-inducible protein 27 like 2B [Source:MGI Symbol;Acc:MGI:1916390]	102.6	31.2	**-2.04**	7.95E-08
Apoa4	apolipoprotein A-IV [Source:MGI Symbol;Acc:MGI:88051]	11871.9	3002.7	**-2.13**	1.61E-08
Wfdc2	WAP four-disulfide core domain 2 [Source:MGI Symbol;Acc:MGI:1914951]	185.0	54.2	**-2.28**	2.28E-11
