# Supplementary material for: Effects of Lingonberry (Vaccinium vitis-idaea L.) Supplementation on Hepatic Gene Expression in High-Fat Diet Fed Mice
Source: Nutrients. 2021 Oct 21;13(11):3693. doi: 10.3390/nu13113693 (PMC8623941; doi:10.3390/nu13113693)
Supplement: Supplementary file 1 [file nutrients-13-03693-s001.zip › Table S5.pdf]

Table S5. The genes upregulated by the high-fat (HF) diet and whose expression was significantly lower in the lingonberry-supplemented high-fat diet group (HF+LGB) (p < 0.05). Mean expression levels are given as DESeq2-normalized counts. p-values are adjusted by false discovery rate (FDR). *Mean of normalizations performed in comparisons HF vs LF and HF+LGB vs HF. LF = low-fat diet.

**Gene**	**Name**	**Mean (LF)**	**Mean (HF)***	**Mean (HF+LGB)***	**FC (HF vs LF)**	**p-value (FDR ajd.) (HF vs LF)**	**FC (HF+LGB vs HF)**	**p-value (FDR ajd.) (HF+LGB vs HF)**
Themis	thymocyte selection associated [Source:MGI Symbol;Acc:MGI:2443552]	34.1	185.7	90.1	**2.69**	< 0.0001	**-1.44**	0.0273
Mogat1	monoacylglycerol O-acyltransferase 1 [Source:MGI Symbol;Acc:MGI:1915643]	19.5	68.4	27.1	**2.51**	< 0.0001	**-1.69**	0.0003
Lgals1	lectin. galactose binding. soluble 1 [Source:MGI Symbol;Acc:MGI:96777]	285.0	830.0	469.1	**2.20**	< 0.0001	**-1.45**	0.0218
Lrrc14b	leucine rich repeat containing 14B [Source:MGI Symbol;Acc:MGI:2145269]	6.3	20.4	11.4	**2.14**	< 0.0001	**-1.41**	0.0347
Tmem28	transmembrane protein 28 [Source:MGI Symbol;Acc:MGI:3648377]	27.4	79.5	28.5	**2.13**	< 0.0001	**-1.73**	0.0001
Clstn3	calsyntenin 3 [Source:MGI Symbol;Acc:MGI:2178323]	277.3	673.1	484.2	**2.07**	< 0.0001	**-1.39**	0.0139
Anxa2	annexin A2 [Source:MGI Symbol;Acc:MGI:88246]	145.5	305.8	184.0	**1.89**	< 0.0001	**-1.44**	0.0176
Lrrc39	leucine rich repeat containing 39 [Source:MGI Symbol;Acc:MGI:1924557]	19.7	50.9	29.9	**1.84**	0.0003	**-1.42**	0.0334
Ifi27l2b	interferon. alpha-inducible protein 27 like 2B [Source:MGI Symbol;Acc:MGI:1916390]	44.1	97.9	31.2	**1.83**	0.0001	**-2.04**	< 0.0001
Gck	glucokinase [Source:MGI Symbol;Acc:MGI:1270854]	1729.4	3380.2	2354.6	**1.82**	< 0.0001	**-1.32**	0.0128
Olig1	oligodendrocyte transcription factor 1 [Source:MGI Symbol;Acc:MGI:1355334]	168.5	315.4	230.7	**1.79**	< 0.0001	**-1.32**	0.0465
Gpc1	glypican 1 [Source:MGI Symbol;Acc:MGI:1194891]	293.2	535.1	211.2	**1.78**	< 0.0001	**-2.04**	< 0.0001
Gngt1	guanine nucleotide binding protein (G protein). gamma transducing activity polypeptide 1 [Source:MGI Symbol;Acc:MGI:109165]	15.5	32.7	20.5	**1.75**	0.0002	**-1.39**	0.0464
Mup-ps14	major urinary protein. pseudogene 14 [Source:MGI Symbol;Acc:MGI:3651980]	11.0	23.6	11.8	**1.74**	0.0007	**-1.43**	0.0113
Mtnr1a	melatonin receptor 1A [Source:MGI Symbol;Acc:MGI:102967]	30.4	55.3	35.3	**1.73**	< 0.0001	**-1.43**	0.0056
Cd36	CD36 molecule [Source:MGI Symbol;Acc:MGI:107899]	1057.2	2079.6	1395.7	**1.73**	0.0004	**-1.40**	0.0105
Cdkn1a	cyclin-dependent kinase inhibitor 1A (P21) [Source:MGI Symbol;Acc:MGI:104556]	46.7	108.6	49.8	**1.73**	0.0002	**-1.69**	< 0.0001
Tubb6	tubulin. beta 6 class V [Source:MGI Symbol;Acc:MGI:1915201]	42.2	85.3	44.7	**1.68**	0.0002	**-1.54**	0.0033

Pdlim2	PDZ and LIM domain 2 [Source:MGI Symbol;Acc:MGI:2384850]	17.8	37.3	20.0	**1.68**	0.0005	**-1.56**	0.0017
Dio1	deiodinase. iodothyronine. type I [Source:MGI Symbol;Acc:MGI:94896]	1771.8	3158.8	2030.7	**1.66**	< 0.0001	**-1.45**	0.0024
Limk1	LIM-domain containing. protein kinase [Source:MGI Symbol;Acc:MGI:104572]	30.8	58.3	37.3	**1.66**	0.0003	**-1.40**	0.0237
Wfdc2	WAP four-disulfide core domain 2 [Source:MGI Symbol;Acc:MGI:1914951]	92.0	179.5	54.2	**1.66**	0.0005	**-2.28**	< 0.0001
Lcn2	lipocalin 2 [Source:MGI Symbol;Acc:MGI:96757]	54.7	165.9	42.2	**1.66**	0.0012	**-1.99**	< 0.0001
Haus8	4HAUS augmin-like complex. subunit 8 [Source:MGI Symbol;Acc:MGI:1923728]	58.6	111.2	65.9	**1.65**	0.0002	**-1.49**	0.0029
Saa1	serum amyloid A 1 [Source:MGI Symbol;Acc:MGI:98221]	513.5	1245.2	471.0	**1.65**	0.0014	**-1.75**	< 0.0001
Plin4	perilipin 4 [Source:MGI Symbol;Acc:MGI:1929709]	74.5	201.9	105.4	**1.65**	0.0016	**-1.55**	0.0030
Mup-ps12	major urinary protein. pseudogene 12 [Source:MGI Symbol;Acc:MGI:3783148]	23.1	47.1	21.2	**1.64**	0.0006	**-1.43**	0.0113
Synj2	synaptojanin 2 [Source:MGI Symbol;Acc:MGI:1201671]	94.8	161.4	118.5	**1.62**	< 0.0001	**-1.32**	0.0477
Gsdme	gasdermin E [Source:MGI Symbol;Acc:MGI:1889850]	14.2	25.7	15.4	**1.61**	0.0003	**-1.42**	0.0157
Cxcl14	chemokine (C-X-C motif) ligand 14 [Source:MGI Symbol;Acc:MGI:1888514]	13.2	25.9	8.4	**1.60**	0.0016	**-1.74**	0.0001
Saa2	serum amyloid A 2 [Source:MGI Symbol;Acc:MGI:98222]	285.4	738.1	222.7	**1.60**	0.0030	**-1.83**	< 0.0001
Tceal8	transcription elongation factor A (SII)-like 8 [Source:MGI Symbol;Acc:MGI:1913934]	322.3	546.1	275.7	**1.59**	< 0.0001	**-1.74**	< 0.0001
Tubb2a	tubulin. beta 2A class IIA [Source:MGI Symbol;Acc:MGI:107861]	293.9	1038.1	274.6	**1.59**	0.0026	**-1.52**	0.0038
Orm3	orosomucoid 3 [Source:MGI Symbol;Acc:MGI:97445]	9.7	22.3	8.5	**1.58**	0.0046	**-1.52**	0.0078
Phlda3	pleckstrin homology like domain. family A. member 3 [Source:MGI Symbol;Acc:MGI:1351485]	17.9	34.6	13.8	**1.57**	0.0037	**-1.78**	< 0.0001
Mrgprb11-ps	MAS-related GPR. member B11. pseudogene [Source:MGI Symbol;Acc:MGI:3033189]	8.6	18.4	9.4	**1.57**	0.0042	**-1.42**	0.0345
Tubb4b	tubulin. beta 4B class IVB [Source:MGI Symbol;Acc:MGI:1915472]	696.0	1205.4	780.6	**1.56**	0.0008	**-1.44**	0.0060
Slc25a35	solute carrier family 25. member 35 [Source:MGI Symbol;Acc:MGI:1919248]	11.6	21.6	11.2	**1.56**	0.0024	**-1.53**	0.0030
S100a10	S100 calcium binding protein A10 (calpactin) [Source:MGI Symbol;Acc:MGI:1339468]	1264.2	2097.6	1116.5	**1.55**	< 0.0001	**-1.66**	< 0.0001

Cd59b	CD59b antigen [Source:MGI Symbol;Acc:MGI:1888996]	37.9	64.0	46.0	**1.55**	0.0002	**-1.31**	0.0473
Ggct	gamma-glutamyl cyclotransferase [Source:MGI Symbol;Acc:MGI:95700]	109.0	163.2	113.3	**1.54**	< 0.0001	**-1.39**	0.0044
Ocstamp	osteoclast stimulatory transmembrane protein [Source:MGI Symbol;Acc:MGI:1921864]	10.1	17.6	10.5	**1.54**	0.0053	**-1.43**	0.0232
Rad51b	RAD51 paralog B [Source:MGI Symbol;Acc:MGI:1099436]	31.9	87.1	23.3	**1.54**	0.0067	**-2.03**	< 0.0001
Zfp423	zinc finger protein 423 [Source:MGI Symbol;Acc:MGI:1891217]	5.5	13.5	6.9	**1.54**	0.0083	**-1.41**	0.0367
Hsd17b10	hydroxysteroid (17-beta) dehydrogenase 10 [Source:MGI Symbol;Acc:MGI:1333871]	2551.4	3960.3	3064.6	**1.53**	< 0.0001	**-1.26**	0.0158
Mup-ps7	major urinary protein. pseudogene 7 [Source:MGI Symbol;Acc:MGI:3651245]	11.4	22.2	16.4	**1.53**	0.0056	**-1.43**	0.0113
Gale	galactose-4-epimerase. UDP [Source:MGI Symbol;Acc:MGI:1921496]	232.0	444.4	215.9	**1.53**	0.0067	**-1.57**	0.0020
Srxn1	sulfiredoxin 1 homolog (S. cerevisiae) [Source:MGI Symbol;Acc:MGI:104971]	676.3	1018.9	794.4	**1.51**	< 0.0001	**-1.27**	0.0074
Ctse	cathepsin E [Source:MGI Symbol;Acc:MGI:107361]	6.8	13.6	8.9	**1.51**	0.0127	**-1.31**	0.0023
Ighm	immunoglobulin heavy constant mu [Source:MGI Symbol;Acc:MGI:96448]	113.6	224.3	113.5	**1.51**	0.0141	**-1.41**	0.0365
Pnldc1	poly(A)-specific ribonuclease (PARN)-like domain containing 1 [Source:MGI Symbol;Acc:MGI:2685159]	144.4	235.1	113.3	**1.48**	0.0030	**-1.56**	0.0037
Ttc39a	tetratricopeptide repeat domain 39A [Source:MGI Symbol;Acc:MGI:2444350]	8.4	29.1	10.7	**1.48**	0.0092	**-1.43**	0.0209
Ubd	ubiquitin D [Source:MGI Symbol;Acc:MGI:1344410]	13.5	41.1	20.1	**1.48**	0.0119	**-1.41**	0.0380
Tagln	transgelin [Source:MGI Symbol;Acc:MGI:106012]	57.9	84.5	49.9	**1.47**	0.0035	**-1.52**	0.0022
Uck1	uridine-cytidine kinase 1 [Source:MGI Symbol;Acc:MGI:98904]	481.9	723.9	587.8	**1.46**	< 0.0001	**-1.23**	0.0055
Them6	thioesterase superfamily member 6 [Source:MGI Symbol;Acc:MGI:1925301]	64.6	96.6	62.4	**1.46**	< 0.0001	**-1.51**	< 0.0001
Tuba1c	tubulin. alpha 1C [Source:MGI Symbol;Acc:MGI:1095409]	236.4	389.9	242.4	**1.43**	0.0229	**-1.40**	0.0327
Col1a1	collagen. type I. alpha 1 [Source:MGI Symbol;Acc:MGI:88467]	61.2	140.6	61.8	**1.43**	0.0281	**-1.44**	0.0180
Smpd3	sphingomyelin phosphodiesterase 3. neutral [Source:MGI Symbol;Acc:MGI:1927578]	60.2	102.1	38.8	**1.43**	0.0292	**-1.83**	< 0.0001
Nupr1	nuclear protein transcription regulator 1 [Source:MGI Symbol;Acc:MGI:1891834]	15.9	28.8	12.8	**1.43**	0.0325	**-1.43**	0.0307

Cpxm1	carboxypeptidase X 1 (M14 family) [Source:MGI Symbol;Acc:MGI:1934569]	13.3	21.6	10.8	**1.43**	0.0339	**-1.53**	0.0063
Col3a1	collagen. type III. alpha 1 [Source:MGI Symbol;Acc:MGI:88453]	322.9	540.9	318.3	**1.42**	0.0390	**-1.40**	0.0465
Slc22a15	solute carrier family 22 (organic anion/cation transporter). member 15 [Source:MGI Symbol;Acc:MGI:3607704]	258.0	354.1	274.0	**1.41**	< 0.0001	**-1.29**	0.0007
Ctps	cytidine 5'-triphosphate synthase [Source:MGI Symbol;Acc:MGI:1858304]	70.0	105.1	65.8	**1.41**	0.0031	**-1.48**	0.0009
Endod1	endonuclease domain containing 1 [Source:MGI Symbol;Acc:MGI:1919196]	39.7	59.5	43.4	**1.41**	0.0035	**-1.30**	0.0372
Ncmap	noncompact myelin associated protein [Source:MGI Symbol;Acc:MGI:2444888]	12.4	20.7	11.8	**1.41**	0.0229	**-1.40**	0.0441
Uap1l1	UDP-N-acteylglucosamine pyrophosphorylase 1-like 1 [Source:MGI Symbol;Acc:MGI:2443318]	87.8	138.2	65.3	**1.41**	0.0300	**-1.69**	0.0001
Pik3r3	phosphoinositide-3-kinase regulatory subunit 3 [Source:MGI Symbol;Acc:MGI:109277]	17.7	27.6	15.9	**1.39**	0.0255	**-1.42**	0.0151
Col1a2	collagen. type I. alpha 2 [Source:MGI Symbol;Acc:MGI:88468]	157.0	238.1	137.7	**1.39**	0.0488	**-1.51**	0.0078
Polr3k	polymerase (RNA) III (DNA directed) polypeptide K [Source:MGI Symbol;Acc:MGI:1914255]	264.8	367.5	299.1	**1.39**	0.0002	**-1.20**	0.0477
Polr2k	polymerase (RNA) II (DNA directed) polypeptide K [Source:MGI Symbol;Acc:MGI:102725]	67.5	92.4	69.9	**1.39**	0.0046	**-1.27**	0.0481
Plekha1	pleckstrin homology domain containing. family A (phosphoinositide binding specific) member 1 [Source:MGI Symbol;Acc:MGI:2442213]	173.6	241.6	171.6	**1.39**	0.0095	**-1.37**	0.0051
Prss23	protease. serine 23 [Source:MGI Symbol;Acc:MGI:1923703]	47.6	68.5	45.1	**1.39**	0.0300	**-1.39**	0.0479
Cd63	CD63 antigen [Source:MGI Symbol;Acc:MGI:99529]	53.4	75.7	38.0	**1.39**	0.0421	**-1.60**	0.0011
Nhlrc1	NHL repeat containing 1 [Source:MGI Symbol;Acc:MGI:2145264]	25.0	35.4	20.7	**1.38**	0.0105	**-1.59**	0.0001
Extl1	exostoses (multiple)-like 1 [Source:MGI Symbol;Acc:MGI:1888742]	213.0	322.8	155.5	**1.38**	0.0132	**-1.77**	< 0.0001
Aqp8	aquaporin 8 [Source:MGI Symbol;Acc:MGI:1195271]	3869.8	5347.3	2480.4	**1.38**	0.0455	**-1.82**	< 0.0001
Nans	N-acetylneuraminic acid synthase (sialic acid synthase) [Source:MGI Symbol;Acc:MGI:2149820]	211.0	284.8	200.6	**1.37**	< 0.0001	**-1.42**	< 0.0001
Dck	deoxycytidine kinase [Source:MGI Symbol;Acc:MGI:102726]	94.0	129.3	107.2	**1.37**	0.0013	**-1.21**	0.0499

Twf2	twinfilin actin binding protein 2 [Source:MGI Symbol;Acc:MGI:1346078]	35.9	48.7	33.6	**1.37**	0.0118	**-1.38**	0.0130
Col12a1	collagen. type XII. alpha 1 [Source:MGI Symbol;Acc:MGI:88448]	26.1	37.6	20.9	**1.37**	0.0364	**-1.64**	< 0.0001
Tnip1	TNFAIP3 interacting protein 1 [Source:MGI Symbol;Acc:MGI:1926194]	123.4	167.1	130.8	**1.36**	0.0007	**-1.26**	0.0223
Mpc1-ps	mitochondrial pyruvate carrier 1. pseudogene [Source:MGI Symbol;Acc:MGI:3781628]	238.1	318.9	271.2	**1.36**	0.0036	**-1.42**	0.0345
Macrod2	MACRO domain containing 2 [Source:MGI Symbol;Acc:MGI:1920149]	48.0	66.7	47.1	**1.36**	0.0059	**-1.34**	0.0110
Ccdc3	coiled-coil domain containing 3 [Source:MGI Symbol;Acc:MGI:1921436]	25.8	35.0	22.7	**1.36**	0.0474	**-1.43**	0.0191
Vcpkmt	valosin containing protein lysine (K) methyltransferase [Source:MGI Symbol;Acc:MGI:2684917]	28.7	40.4	26.8	**1.35**	0.0300	**-1.40**	0.0078
Rasgrp2	RAS. guanyl releasing protein 2 [Source:MGI Symbol;Acc:MGI:1333849]	159.1	222.5	139.2	**1.34**	0.0226	**-1.45**	0.0019
Galnt10	polypeptide N-acetylgalactosaminyltransferase 10 [Source:MGI Symbol;Acc:MGI:1890480]	95.0	126.3	101.1	**1.33**	0.0030	**-1.22**	0.0337
Mfsd9	major facilitator superfamily domain containing 9 [Source:MGI Symbol;Acc:MGI:2443548]	52.8	71.4	57.2	**1.33**	0.0149	**-1.25**	0.0418
Tmem177	transmembrane protein 177 [Source:MGI Symbol;Acc:MGI:1913593]	131.7	175.2	142.6	**1.32**	< 0.0001	**-1.20**	0-0465
Psmd14	proteasome (prosome. macropain) 26S subunit. non-ATPase. 14 [Source:MGI Symbol;Acc:MGI:1913284]	717.9	937.6	805.5	**1.32**	< 0.0001	**-1.16**	0.0317
Lyplal1	lysophospholipase-like 1 [Source:MGI Symbol;Acc:MGI:2385115]	223.9	293.9	241.9	**1.32**	< 0.0001	**-1.19**	0.0324
Cryzl2	crystallin zeta like 2 [Source:MGI Symbol;Acc:MGI:2448516]	356.5	465.9	373.8	**1.32**	< 0.0001	**-1.22**	0.0126
Exosc7	exosome component 7 [Source:MGI Symbol;Acc:MGI:1913696]	387.6	507.2	419.6	**1.32**	0.0002	**-1.21**	0.0285
Aldh1a7	aldehyde dehydrogenase family 1. subfamily A7 [Source:MGI Symbol;Acc:MGI:1347050]	3662.6	4875.5	4175.1	**1.32**	0.0011	**-1.18**	0.0375
Tbc1d7	TBC1 domain family. member 7 [Source:MGI Symbol;Acc:MGI:1914296]	109.3	144.2	113.7	**1.31**	0.0042	**-1.26**	0.0076
Cebpe	CCAAT/enhancer binding protein (C/EBP). epsilon [Source:MGI Symbol;Acc:MGI:103572]	149.0	210.9	150.2	**1.31**	0.0206	**-1.33**	0.0051
Mrps6	mitochondrial ribosomal protein S6 [Source:MGI Symbol;Acc:MGI:2153111]	92.5	122.0	87.1	**1.30**	0.0050	**-1.35**	0.0027

Sae1	SUMO1 activating enzyme subunit 1 [Source:MGI Symbol;Acc:MGI:1929264]	303.8	388.6	312.3	**1.29**	0.0004	**-1.22**	0.0032
Zmat3	zinc finger matrin type 3 [Source:MGI Symbol;Acc:MGI:1195270]	96.0	124.6	84.9	**1.29**	0.0044	**-1.39**	0.0001
Atox1	antioxidant 1 copper chaperone [Source:MGI Symbol;Acc:MGI:1333855]	1234.7	1619.8	1236.3	**1.29**	0.0286	**-1.27**	0.0380
Rexo2	RNA exonuclease 2 [Source:MGI Symbol;Acc:MGI:1888981]	802.2	1029.1	855.9	**1.28**	0.0002	**-1.20**	0.0081
Bet1	Bet1 golgi vesicular membrane trafficking protein [Source:MGI Symbol;Acc:MGI:1343104]	791.1	997.2	824.3	**1.28**	0.0036	**-1.21**	0.0481
Tcta	T cell leukemia translocation altered gene [Source:MGI Symbol;Acc:MGI:1918829]	269.7	350.5	290.5	**1.28**	0.0053	**-1.18**	0.0457
Uba5	ubiquitin-like modifier activating enzyme 5 [Source:MGI Symbol;Acc:MGI:1913913]	393.8	522.5	413.1	**1.28**	0.0149	**-1.25**	0.0021
Mad2l1	MAD2 mitotic arrest deficient-like 1 [Source:MGI Symbol;Acc:MGI:1860374]	53.3	68.7	51.3	**1.28**	0.0176	**-1.30**	0.0074
Ndufb3	NADH:ubiquinone oxidoreductase subunit B3 [Source:MGI Symbol;Acc:MGI:1913745]	839.2	1112.5	837.4	**1.28**	0.0313	**-1.27**	0.0435
Wwtr1	WW domain containing transcription regulator 1 [Source:MGI Symbol;Acc:MGI:1917649]	389.7	485.0	362.2	**1.27**	0.0083	**-1.28**	0.0349
Nhp2	NHP2 ribonucleoprotein [Source:MGI Symbol;Acc:MGI:1098547]	309.4	399.0	300.4	**1.27**	0.0252	**-1.28**	0.0184
Slc35b1	solute carrier family 35. member B1 [Source:MGI Symbol;Acc:MGI:1343133]	548.6	701.3	498.7	**1.27**	0.0305	**-1.38**	0.0009
Ccng1	cyclin G1 [Source:MGI Symbol;Acc:MGI:102890]	760.9	958.5	822.5	**1.26**	< 0.0001	**-1.16**	0.0031
Slc25a40	solute carrier family 25. member 40 [Source:MGI Symbol;Acc:MGI:2442486]	59.3	76.2	56.2	**1.26**	0.0272	**-1.28**	0.0095
Snupn	snurportin 1 [Source:MGI Symbol;Acc:MGI:1913319]	73.9	96.6	86.2	**1.26**	0.0293	**-1.34**	0.0004
Ucp2	uncoupling protein 2 (mitochondrial. proton carrier) [Source:MGI Symbol;Acc:MGI:109354]	427.0	533.9	422.6	**1.26**	0.0381	**-1.26**	0.0388
Slc50a1	solute carrier family 50 (sugar transporter). member 1 [Source:MGI Symbol;Acc:MGI:107417]	88.3	116.1	80.8	**1.26**	0.0488	**-1.37**	0.0008
Smarca4	SWI/SNF related. matrix associated. actin dependent regulator of chromatin. subfamily a. member 4 [Source:MGI Symbol;Acc:MGI:88192]	761.7	961.6	790.3	**1.25**	0.0023	**-1.20**	0.0226
Dut	deoxyuridine triphosphatase [Source:MGI Symbol;Acc:MGI:1346051]	132.7	166.9	150.4	**1.25**	0.0227	**-1.47**	0.0057
Enpp2	ectonucleotide pyrophosphatase/phosphodiesterase 2 [Source:MGI Symbol;Acc:MGI:1321390]	1592.0	2030.3	1648.8	**1.25**	0.0233	**-1.19**	0.0310

Hexb	hexosaminidase B [Source:MGI Symbol;Acc:MGI:96074]	234.2	302.1	235.9	**1.25**	0.0424	**-1.34**	0.0013
Tpmt	thiopurine methyltransferase [Source:MGI Symbol;Acc:MGI:98812]	733.6	904.5	788.4	**1.24**	< 0.0001	**-1.26**	0.0114
Stx4a	syntaxin 4A (placental) [Source:MGI Symbol;Acc:MGI:893577]	524.4	630.9	521.5	**1.24**	0.0013	**-1.17**	0.0352
Pgp	phosphoglycolate phosphatase [Source:MGI Symbol;Acc:MGI:1914328]	423.0	510.1	382.3	**1.24**	0.0164	**-1.32**	0.0008
Rtn4ip1	reticulon 4 interacting protein 1 [Source:MGI Symbol;Acc:MGI:2178759]	388.1	491.8	386.8	**1.23**	0.0018	**-1.27**	0.0001
Tmem43	transmembrane protein 43 [Source:MGI Symbol;Acc:MGI:1921372]	149.5	183.9	143.9	**1.23**	0.0211	**-1.27**	0.0112
Rcan1	regulator of calcineurin 1 [Source:MGI Symbol;Acc:MGI:1890564]	274.3	345.7	264.9	**1.23**	0.0286	**-1.27**	0.0141
Opa3	optic atrophy 3 [Source:MGI Symbol;Acc:MGI:2686271]	797.4	962.9	821.6	**1.22**	< 0.0001	**-1.16**	0.0177
Mrpl16	mitochondrial ribosomal protein L16 [Source:MGI Symbol;Acc:MGI:2137219]	575.4	693.8	579.6	**1.22**	0.0009	**-1.19**	0.0029
Slc22a1	solute carrier family 22 (organic cation transporter). member 1 [Source:MGI Symbol;Acc:MGI:108111]	5848.9	7227.5	6024.4	**1.22**	0.0040	**-1.21**	0.0335
Nudcd2	NudC domain containing 2 [Source:MGI Symbol;Acc:MGI:1277103]	432.8	527.2	422.1	**1.22**	0.0059	**-1.21**	0.0232
Arpp19	cAMP-regulated phosphoprotein 19 [Source:MGI Symbol;Acc:MGI:1891691]	1005.3	1218.5	968.4	**1.22**	0.0409	**-1.23**	0.0367
Agpat2	1-acylglycerol-3-phosphate O-acyltransferase 2 (lysophosphatidic acid acyltransferase. beta) [Source:MGI Symbol;Acc:MGI:1914762]	3369.1	4012.9	3202.3	**1.22**	0.0435	**-1.23**	0.0032
Taldo1	transaldolase 1 [Source:MGI Symbol;Acc:MGI:1274789]	1606.2	1950.9	1632.0	**1.21**	0.0166	**-1.20**	0.0130
Dnajc9	DnaJ heat shock protein family (Hsp40) member C9 [Source:MGI Symbol;Acc:MGI:1915326]	90.0	108.7	82.9	**1.21**	0.0232	**-1.23**	0.0347
Ppcs	phosphopantothenoylcysteine synthetase [Source:MGI Symbol;Acc:MGI:1915237]	333.9	411.3	350.3	**1.21**	0.0331	**-1.31**	0.0114
Cycs	cytochrome c. somatic [Source:MGI Symbol;Acc:MGI:88578]	464.4	557.3	428.6	**1.21**	0.0332	**-1.27**	0.0052
Ufm1	ubiquitin-fold modifier 1 [Source:MGI Symbol;Acc:MGI:1915140]	751.3	897.6	765.8	**1.21**	< 0.0001	**-1.15**	0.0006
Fbxw9	F-box and WD-40 domain protein 9 [Source:MGI Symbol;Acc:MGI:1915878]	526.2	621.2	467.0	**1.21**	0.0244	**-1.32**	0.0004

Psmd8	proteasome (prosome. macropain) 26S subunit. non-ATPase. 8 [Source:MGI Symbol;Acc:MGI:1888669]	1872.9	2212.2	1870.3	**1.20**	0.0130	**-1.17**	0.0402
Hsd17b12	hydroxysteroid (17-beta) dehydrogenase 12 [Source:MGI Symbol;Acc:MGI:1926967]	2982.7	3518.8	2991.8	**1.20**	0.0204	**-1.19**	0.0073
Swsap1	SWIM type zinc finger 7 associated protein 1 [Source:MGI Symbol;Acc:MGI:1914212]	81.6	97.4	71.8	**1.20**	0.0356	**-1.34**	0.0018
Rogdi	rogdi homolog [Source:MGI Symbol;Acc:MGI:1913299]	204.7	244.5	186.0	**1.19**	0.0343	**-1.31**	< 0.0001
Mafk	v-maf musculoaponeurotic fibrosarcoma oncogene family. protein K (avian) [Source:MGI Symbol;Acc:MGI:99951]	264.9	308.1	263.9	**1.18**	0.0479	**-1.17**	0.0478
Stimate	STIM activating enhancer [Source:MGI Symbol;Acc:MGI:1921500]	237.4	271.3	227.9	**1.16**	0.0316	**-1.20**	0.0034
Rcbtb2	regulator of chromosome condensation (RCC1) and BTB (POZ) domain containing protein 2 [Source:MGI Symbol;Acc:MGI:1917200]	690.1	792.3	683.3	**1.16**	0.0007	**-1.14**	0.0015
Rraga	Ras-related GTP binding A [Source:MGI Symbol;Acc:MGI:1915691]	584.9	669.9	597.5	**1.16**	0.0137	**-1.53**	0.0008
Sh3bgrl	SH3-binding domain glutamic acid-rich protein like [Source:MGI Symbol;Acc:MGI:1930849]	2001.9	2244.5	1920.9	**1.15**	0.0143	**-1.14**	0.0384
Dbnl	drebrin-like [Source:MGI Symbol;Acc:MGI:700006]	506.5	575.4	492.8	**1.15**	0.0207	**-1.16**	0.0151
Eif2d	eukaryotic translation initiation factor 2D [Source:MGI Symbol;Acc:MGI:109342]	524.4	613.0	508.6	**1.15**	0.0440	**-1.18**	0.0012
Calm1	calmodulin 1 [Source:MGI Symbol;Acc:MGI:88251]	3031.6	3422.5	2967.4	**1.14**	0.0144	**-1.14**	0.0293
Anapc4	anaphase promoting complex subunit 4 [Source:MGI Symbol;Acc:MGI:1098673]	309.6	340.2	295.8	**1.14**	0.0239	**-1.15**	0.0300
Ddx1	DEAD (Asp-Glu-Ala-Asp) box polypeptide 1 [Source:MGI Symbol;Acc:MGI:2144727]	1211.8	1361.9	1199.7	**1.14**	0.0366	**-1.13**	0.0044
Ctso	cathepsin O [Source:MGI Symbol;Acc:MGI:2139628]	1007.8	1129.9	1068.1	**1.13**	0.0094	**-1.31**	0.0023
Tprgl	transformation related protein 63 regulated like [Source:MGI Symbol;Acc:MGI:1915058]	909.5	1008.3	861.5	**1.12**	0.0390	**-1.15**	0.0139
