# Supplementary material for: Effects of Lingonberry (Vaccinium vitis-idaea L.) Supplementation on Hepatic Gene Expression in High-Fat Diet Fed Mice
Source: Nutrients. 2021 Oct 21;13(11):3693. doi: 10.3390/nu13113693 (PMC8623941; doi:10.3390/nu13113693)
Supplement: Supplementary file 1 [file nutrients-13-03693-s001.zip › Table S6.pdf]

Table S6. The genes downregulated by the high-fat (HF) diet, and whose expression was significantly higher in the lingonberry-supplemented high-fat diet group (HF+LGB) (p < 0.05). Mean expression levels are given as DESeq2-normalized counts. p-values are adjusted by false discovery rate (FDR). *Mean of normalizations performed in comparisons HF vs LF and HF+LGB vs HF. LF = low-fat diet.

Gene	Name	Mean (LF)	Mean (HF)*	Mean (HF+LGB)*	FC (HF vs LF)	p-value (FDR ajd.) (HF vs LF)	FC (HF+LGB vs HF)	p-value (FDR ajd.) (HF+LGB vs HF)
Adgrf1	adhesion G protein-coupled receptor F1 [Source:MGI Symbol;Acc:MGI:1924846]	122.3	38.1	84.5	**-2.06**	< 0.0001	**2.22**	< 0.0001
Igfbp2	insulin-like growth factor binding protein 2 [Source:MGI Symbol;Acc:MGI:96437]	7680.0	3501.8	6263.7	**-1.95**	< 0.0001	**1.71**	< 0.0001
Grm8	glutamate receptor, metabotropic 8 [Source:MGI Symbol;Acc:MGI:1351345]	20.9	7.6	16.2	**-1.91**	< 0.0001	**1.39**	0.0477
Sds	serine dehydratase [Source:MGI Symbol;Acc:MGI:98270]	3836.6	2143.7	2800.6	**-1.62**	0.0007	**1.39**	0.0123
Sox12	SRY (sex determining region Y)-box 12 [Source:MGI Symbol;Acc:MGI:98360]	111.7	64.0	91.4	**-1.61**	< 0.0001	**1.39**	0.0074
Rnf145	ring finger protein 145 [Source:MGI Symbol;Acc:MGI:1921565]	555.3	332.9	439.1	**-1.58**	< 0.0001	**1.30**	0.0023
C8b	complement component 8, beta polypeptide [Source:MGI Symbol;Acc:MGI:88236]	4459.6	2904.9	3664.4	**-1.53**	< 0.0001	**1.28**	0.0459
Etnppl	ethanolamine phosphate phospholyase [Source:MGI Symbol;Acc:MGI:1919010]	2583.8	1568.4	2374.0	**-1.51**	0.0004	**1.38**	0.0336
Adamts7	a disintegrin-like and metallopeptidase (reprolysin type) with thrombospondin type 1 motif, 7 [Source:MGI Symbol;Acc:MGI:1347346]	133.4	87.1	116.3	**-1.48**	0.0002	**1.39**	0.0013
Ihh	Indian hedgehog [Source:MGI Symbol;Acc:MGI:96533]	37.2	23.8	32.9	**-1.45**	0.0231	**1.39**	0.0465
Cps1	carbamoyl-phosphate synthetase 1 [Source:MGI Symbol;Acc:MGI:891996]	46243.9	30712.3	39913.5	**-1.44**	0.0021	**1.36**	0.0119
Slco2a1	solute carrier organic anion transporter family, member 2a1 [Source:MGI Symbol;Acc:MGI:1346021]	867.5	600.2	914.8	**-1.42**	0.0002	**1.48**	0.0004
Cyp39a1	cytochrome P450, family 39, subfamily a, polypeptide 1 [Source:MGI Symbol;Acc:MGI:1927096]	337.0	209.9	249.3	**-1.42**	0.0147	**1.24**	0.0233
Pgap1	post-GPI attachment to proteins 1 [Source:MGI Symbol;Acc:MGI:2443342]	468.1	327.9	391.0	**-1.41**	0.0008	**1.23**	0.0349
Slc7a2	solute carrier family 7 (cationic amino acid transporter, y+ system), member 2 [Source:MGI Symbol;Acc:MGI:99828]	11160.9	7630.1	12519.6	**-1.41**	0.0046	**1.65**	< 0.0001

Cyp2c67	cytochrome P450, family 2, subfamily c, polypeptide 67 [Source:MGI Symbol;Acc:MGI:3612288]	3835.0	2729.5	3250.8	**-1.40**	< 0.0001	**1.24**	0.0126
Zfp516	zinc finger protein 516 [Source:MGI Symbol;Acc:MGI:2443957]	84.3	58.7	75.6	**-1.40**	0.0014	**1.29**	0.0322
Irs2	insulin receptor substrate 2 [Source:MGI Symbol;Acc:MGI:109334]	454.3	298.9	402.9	**-1.40**	0.0063	**1.31**	0.0376
Rbm33	RNA binding motif protein 33 [Source:MGI Symbol;Acc:MGI:1919670]	661.6	486.6	574.0	**-1.39**	0.0004	**1.27**	0.0416
Abca8a	ATP-binding cassette, sub-family A (ABC1), member 8a [Source:MGI Symbol;Acc:MGI:2386846]	2073.7	1456.5	1887.0	**-1.39**	0.0042	**1.34**	0.0088
Agtr1a	angiotensin II receptor, type 1a [Source:MGI Symbol;Acc:MGI:87964]	1315.0	912.6	1138.2	**-1.39**	< 0.0001	**1.26**	0.0023
Setd1b	SET domain containing 1B [Source:MGI Symbol;Acc:MGI:2652820]	517.4	365.8	485.3	**-1.39**	0.0034	**1.33**	0.0069
Col4a5	collagen, type IV, alpha 5 [Source:MGI Symbol;Acc:MGI:88456]	34.5	22.6	40.8	**-1.38**	0.0424	**1.39**	0.0483
Tle2	transducin-like enhancer of split 2 [Source:MGI Symbol;Acc:MGI:104635]	90.1	73.2	83.1	**-1.37**	0.0038	**1.34**	0.0029
Ccnl2	cyclin L2 [Source:MGI Symbol;Acc:MGI:1927119]	644.6	557.0	553.0	**-1.36**	< 0.0001	**1.21**	0.0339
Pdia5	protein disulfide isomerase associated 5 [Source:MGI Symbol;Acc:MGI:1919849]	849.3	613.0	766.5	**-1.36**	0.0026	**1.29**	< 0.0001
Tmem25	transmembrane protein 25 [Source:MGI Symbol;Acc:MGI:1918937]	409.5	335.3	411.5	**-1.35**	0.0001	**1.40**	< 0.0001
Lifr	LIF receptor alpha [Source:MGI Symbol;Acc:MGI:96788]	3398.1	2652.7	3303.2	**-1.35**	0.0134	**1.33**	0.0230
Vegfa	vascular endothelial growth factor A [Source:MGI Symbol;Acc:MGI:103178]	1325.1	1058.1	1186.7	**-1.34**	< 0.0001	**1.23**	0.0001
Rbfox2	RNA binding protein, fox-1 homolog (C. elegans) 2 [Source:MGI Symbol;Acc:MGI:1933973]	422.2	325.2	375.7	**-1.34**	< 0.0001	**1.21**	0.0018
Dclk3	doublecortin-like kinase 3 [Source:MGI Symbol;Acc:MGI:3039580]	251.6	173.5	234.5	**-1.34**	0.023	**1.32**	0.0143
Scamp1	secretory carrier membrane protein 1 [Source:MGI Symbol;Acc:MGI:1349480]	1279.3	923.6	1109.2	**-1.33**	0.0001	**1.21**	0.0005
Amigo1	adhesion molecule with Ig like domain 1 [Source:MGI Symbol;Acc:MGI:2653612]	141.2	103.7	136.3	**-1.33**	0.0052	**1.35**	0.0014
Tk1	thymidine kinase 1 [Source:MGI Symbol;Acc:MGI:98763]	314.7	235.8	347.3	**-1.32**	0.0482	**1.47**	0.0010
Sorbs3	sorbin and SH3 domain containing 3 [Source:MGI Symbol;Acc:MGI:700013]	465.6	376.2	551.9	**-1.31**	0.0044	**1.59**	< 0.0001

Zfp595	zinc finger protein 595 [Source:MGI Symbol;Acc:MGI:3040707]	64.2	52.9	65.9	**-1.31**	0.0123	**1.33**	0.0086
Urad	ureidoimidazoline (2-oxo-4-hydroxy-4-carboxy-5) decarboxylase [Source:MGI Symbol;Acc:MGI:3647519]	447.8	345.2	420.1	**-1.31**	0.0451	**1.29**	0.0146
C3	complement component 3 [Source:MGI Symbol;Acc:MGI:88227]	87811.9	65964.4	96192.1	**-1.30**	0.0087	**1.42**	0.0002
Fbxo21	F-box protein 21 [Source:MGI Symbol;Acc:MGI:1924223]	3026.3	2274.3	3145.7	**-1.30**	0.0135	**1.40**	< 0.0001
Cxcl12	chemokine (C-X-C motif) ligand 12 [Source:MGI Symbol;Acc:MGI:103556]	5823.7	4456.8	5544.4	**-1.29**	0.0005	**1.27**	0.0003
Sertad2	SERTA domain containing 2 [Source:MGI Symbol;Acc:MGI:1931026]	382.0	295.3	356.3	**-1.29**	0.0029	**1.22**	0.0300
Map3k21	mitogen-activated protein kinase kinase kinase 21 [Source:MGI Symbol;Acc:MGI:2385307]	116.3	88.4	114.5	**-1.28**	0.0030	**1.29**	0.0111
Hal	histidine ammonia lyase [Source:MGI Symbol;Acc:MGI:96010]	6753.4	5132.5	7305.0	**-1.28**	0.0070	**1.41**	0.0024
Slc6a6	solute carrier family 6 (neurotransmitter transporter, taurine), member 6 [Source:MGI Symbol;Acc:MGI:98488]	1767.2	1323.9	1646.4	**-1.28**	0.0130	**1.26**	0.0286
Tet3	tet methylcytosine dioxygenase 3 [Source:MGI Symbol;Acc:MGI:2446229]	328.2	256.7	317.5	**-1.28**	0.0226	**1.27**	0.0253
Il17rb	interleukin 17 receptor B [Source:MGI Symbol;Acc:MGI:1355292]	130.0	100.4	137.6	**-1.28**	0.0311	**1.35**	0.0144
Tmem63b	transmembrane protein 63b [Source:MGI Symbol;Acc:MGI:2387609]	1379.8	1093.0	1278.6	**-1.27**	< 0.0001	**1.21**	0.0015
Ints6	integrator complex subunit 6 [Source:MGI Symbol;Acc:MGI:1202397]	297.7	238.2	276.3	**-1.27**	0.0026	**1.25**	0.0023
Cecr2	CECR2, histone acetyl-lysine reader [Source:MGI Symbol;Acc:MGI:1923799]	233.7	188.3	241.5	**-1.27**	0.0144	**1.34**	0.0030
Zfp292	zinc finger protein 292 [Source:MGI Symbol;Acc:MGI:1353423]	256.0	199.6	245.2	**-1.27**	0.0449	**1.28**	0.0235
Sema4g	sema domain, immunoglobulin domain (Ig), transmembrane domain (TM) and short cytoplasmic domain, (semaphorin) 4G [Source:MGI Symbol;Acc:MGI:1347047]	4340.8	3584.2	4634.9	**-1.27**	0.0031	**1.38**	< 0.0001
Sirt1	sirtuin 1 [Source:MGI Symbol;Acc:MGI:2135607]	197.8	158.3	176.7	**-1.27**	0.0033	**1.18**	0.0484
Taok3	TAO kinase 3 [Source:MGI Symbol;Acc:MGI:3041177]	1347.6	1043.3	1269.6	**-1.27**	0.0037	**1.21**	0.0107

Fnbp4	formin binding protein 4 [Source:MGI Symbol;Acc:MGI:1860513]	175.9	154.0	168.0	**-1.27**	0.0046	**1.24**	0.0327
Foxa3	forkhead box A3 [Source:MGI Symbol;Acc:MGI:1347477]	634.4	530.5	716.4	**-1.27**	0.0346	**1.47**	< 0.0001
Scnn1a	sodium channel, nonvoltage-gated 1 alpha [Source:MGI Symbol;Acc:MGI:101782]	396.9	324.2	505.2	**-1.26**	0.0425	**1.59**	< 0.0001
Akap8	A kinase (PRKA) anchor protein 8 [Source:MGI Symbol;Acc:MGI:1928488]	421.2	358.8	405.7	**-1.25**	0.00084	**1.23**	0.0105
Arid4b	AT rich interactive domain 4B (RBP1-like) [Source:MGI Symbol;Acc:MGI:2137512]	308.0	259.4	291.1	**-1.25**	0.0013	**1.21**	0.0240
Dcaf6	DDB1 and CUL4 associated factor 6 [Source:MGI Symbol;Acc:MGI:1921356]	734.1	593.9	729.1	**-1.25**	0.0059	**1.27**	0.0009
Asap3	ArfGAP with SH3 domain, ankyrin repeat and PH domain 3 [Source:MGI Symbol;Acc:MGI:2684986]	102.3	85.1	141.9	**-1.25**	0.0345	**1.69**	< 0.0001
Brap	BRCA1 associated protein [Source:MGI Symbol;Acc:MGI:1919649]	3167.8	2553.1	2840.4	**-1.24**	< 0.0001	**1.16**	0.0012
Baiap2l1	BAI1-associated protein 2-like 1 [Source:MGI Symbol;Acc:MGI:1914148]	604.0	486.5	554.5	**-1.24**	0.0020	**1.19**	0.0050
Luc7l2	LUC7-like 2 (S. cerevisiae) [Source:MGI Symbol;Acc:MGI:2183260]	972.1	828.9	944.6	**-1.24**	0.0114	**1.25**	0.0091
Bmp6	bone morphogenetic protein 6 [Source:MGI Symbol;Acc:MGI:88182]	99.8	75.2	96.1	**-1.24**	0.0175	**1.28**	0.0250
Eif4b	eukaryotic translation initiation factor 4B [Source:MGI Symbol;Acc:MGI:95304]	6182.9	5013.6	5421.0	**-1.23**	< 0.0001	**1.12**	0.0416
Zcchc7	zinc finger, CCHC domain containing 7 [Source:MGI Symbol;Acc:MGI:2442912]	127.0	106.4	125.0	**-1.23**	0.0305	**1.24**	0.0458
Zcchc2	zinc finger, CCHC domain containing 2 [Source:MGI Symbol;Acc:MGI:2444114]	451.5	372.9	477.6	**-1.23**	0.0377	**1.28**	0.0126
Zdhhc20	zinc finger, DHHC domain containing 20 [Source:MGI Symbol;Acc:MGI:1923215]	299.6	234.7	272.7	**-1.22**	0.0023	**1.19**	0.0265
Scarb1	scavenger receptor class B, member 1 [Source:MGI Symbol;Acc:MGI:893578]	2969.1	2425.6	2809.0	**-1.22**	0.0037	**1.20**	0.0095
Clk4	CDC like kinase 4 [Source:MGI Symbol;Acc:MGI:1098551]	285.9	259.5	266.1	**-1.22**	0.0043	**1.18**	0.0500
Zmym2	zinc finger, MYM-type 2 [Source:MGI Symbol;Acc:MGI:1923257]	377.9	312.8	356.0	**-1.22**	0.0166	**1.21**	0.0334
Kdm6a	lysine (K)-specific demethylase 6A [Source:MGI Symbol;Acc:MGI:1095419]	198.2	164.7	194.1	**-1.22**	0.0476	**1.23**	0.0347

Cpsf7	cleavage and polyadenylation specific factor 7 [Source:MGI Symbol;Acc:MGI:1917826]	295.4	258.4	276.3	**-1.21**	0.0024	**1.17**	0.0163
Plekhg3	pleckstrin homology domain containing, family G (with RhoGef domain) member 3 [Source:MGI Symbol;Acc:MGI:2388284]	637.0	540.0	660.2	**-1.21**	0.0093	**1.29**	0.0007
Tns2	tensin 2 [Source:MGI Symbol;Acc:MGI:2387586]	1329.5	1103.5	1297.8	**-1.21**	0.0107	**1.21**	0.0087
Slc25a22	solute carrier family 25 (mitochondrial carrier, glutamate), member 22 [Source:MGI Symbol;Acc:MGI:1915517]	3070.0	2563.3	3017.3	**-1.21**	0.0195	**1.22**	0.0074
Slc17a2	solute carrier family 17 (sodium phosphate), member 2 [Source:MGI Symbol;Acc:MGI:2443098]	2687.6	2277.0	2649.0	**-1.21**	0.0059	**1.22**	0.0050
Prpf38b	PRP38 pre-mRNA processing factor 38 (yeast) domain containing B [Source:MGI Symbol;Acc:MGI:1914171]	411.4	369.1	410.5	**-1.21**	0.0123	**1.22**	0.0040
Gpat4	glycerol-3-phosphate acyltransferase 4 [Source:MGI Symbol;Acc:MGI:2142716]	3744.2	3160.5	3943.1	**-1.21**	0.0186	**1.29**	0.0001
Mllt10	myeloid/lymphoid or mixed-lineage leukemia; translocated to, 10 [Source:MGI Symbol;Acc:MGI:1329038]	372.8	311.9	356.9	**-1.21**	0.0374	**1.21**	0.0482
Trim28	tripartite motif-containing 28 [Source:MGI Symbol;Acc:MGI:109274]	1067.0	902.4	995.7	**-1.20**	< 0.0001	**1.13**	0.0029
Itih2	inter-alpha trypsin inhibitor, heavy chain 2 [Source:MGI Symbol;Acc:MGI:96619]	17002.7	13960.0	18862.9	**-1.20**	0.0180	**1.36**	0.0006
Akap8l	A kinase (PRKA) anchor protein 8-like [Source:MGI Symbol;Acc:MGI:1860606]	207.6	190.6	222.6	**-1.20**	0.0182	**1.33**	< 0.0001
Atp11b	ATPase, class VI, type 11B [Source:MGI Symbol;Acc:MGI:1923545]	808.7	670.0	779.1	**-1.20**	0.0363	**1.20**	0.0327
Tnrc6a	trinucleotide repeat containing 6a [Source:MGI Symbol;Acc:MGI:2385292]	567.1	508.0	589.7	**-1.20**	0.0425	**1.25**	0.0234
Paics	phosphoribosylaminoimidazole carboxylase, phosphoribosylaminoribosylaminoimidazole, succinocarboxamide synthetase [Source:MGI Symbol;Acc:MGI:1914304]	3686.3	3138.9	3398.6	**-1.19**	0.0063	**1.14**	0.0009
Med17	mediator complex subunit 17 [Source:MGI Symbol;Acc:MGI:2182585]	197.7	166.1	190.8	**-1.19**	0.0109	**1.18**	0.0347
Ces2a	carboxylesterase 2A [Source:MGI Symbol;Acc:MGI:2142491]	2500.0	2052.4	3659.3	**-1.19**	0.0411	**1.73**	< 0.0001
Ptcd1	pentatricopeptide repeat domain 1 [Source:MGI Symbol;Acc:MGI:1919049]	188.6	158.6	182.6	**-1.19**	0.0425	**1.19**	0.0337

Dpyd	dihydropyrimidine dehydrogenase [Source:MGI Symbol;Acc:MGI:2139667]	9510.8	7947.4	9202.4	**-1.18**	0.0055	**1.20**	0.0176
Stag2	stromal antigen 2 [Source:MGI Symbol;Acc:MGI:1098583]	1188.6	986.6	1094.8	**-1.18**	0.0091	**1.15**	0.0440
Sart3	squamous cell carcinoma antigen recognized by T cells 3 [Source:MGI Symbol;Acc:MGI:1858230]	224.9	193.2	221.3	**-1.18**	0.0258	**1.19**	0.0143
Aass	aminoadipate-semialdehyde synthase [Source:MGI Symbol;Acc:MGI:1353573]	5085.3	4361.6	5503.4	**-1.18**	0.0265	**1.31**	0.0002
Kdm3a	lysine (K)-specific demethylase 3A [Source:MGI Symbol;Acc:MGI:98847]	214.7	181.2	221.0	**-1.18**	0.0286	**1.28**	0.0004
Edc4	enhancer of mRNA decapping 4 [Source:MGI Symbol;Acc:MGI:2446249]	227.0	204.5	232.9	**-1.18**	0.0366	**1.23**	0.0115
Zc3h14	zinc finger CCCH type containing 14 [Source:MGI Symbol;Acc:MGI:1919824]	822.9	701.5	770.2	**-1.17**	0.0022	**1.15**	0.0215
Hbs1l	Hbs1-like (S. cerevisiae) [Source:MGI Symbol;Acc:MGI:1891704]	1028.1	887.0	1038.3	**-1.17**	0.0036	**1.22**	< 0.0001
Ctdsp2	CTD (carboxy-terminal domain, RNA polymerase II, polypeptide A) small phosphatase 2 [Source:MGI Symbol;Acc:MGI:1098748]	843.7	708.5	801.2	**-1.17**	0.0043	**1.16**	0.0048
Kdm5a	lysine (K)-specific demethylase 5A [Source:MGI Symbol;Acc:MGI:2136980]	525.5	448.0	529.3	**-1.17**	0.0056	**1.23**	0.0008
Zfp110	zinc finger protein 110 [Source:MGI Symbol;Acc:MGI:1890378]	485.5	416.2	452.8	**-1.16**	0.0018	**1.13**	0.0385
Sf1	splicing factor 1 [Source:MGI Symbol;Acc:MGI:1095403]	1253.8	1098.5	1219.9	**-1.16**	0.0195	**1.17**	0.0436
Sf3b1	splicing factor 3b, subunit 1 [Source:MGI Symbol;Acc:MGI:1932339]	1577.3	1405.6	1586.7	**-1.16**	0.0468	**1.20**	0.0115
Taok2	TAO kinase 2 [Source:MGI Symbol;Acc:MGI:1915919]	566.5	513.2	552.9	**-1.16**	0.0309	**1.17**	0.0185
Son	Son DNA binding protein [Source:MGI Symbol;Acc:MGI:98353]	2007.0	1800.8	1914.7	**-1.15**	0.0311	**1.14**	0.0487
Prr14	proline rich 14 [Source:MGI Symbol;Acc:MGI:2384565]	432.1	392.0	426.4	**-1.14**	0.0440	**1.16**	0.0385
Ddx5	DEAD (Asp-Glu-Ala-Asp) box polypeptide 5 [Source:MGI Symbol;Acc:MGI:105037]	4044.1	3686.3	3989.4	**-1.14**	0.0491	**1.16**	0.0006
Amfr	autocrine motility factor receptor [Source:MGI Symbol;Acc:MGI:1345634]	6046.2	5250.9	5796.3	**-1.13**	< 0.0001	**1.13**	< 0.0001
Acin1	apoptotic chromatin condensation inducer 1 [Source:MGI Symbol;Acc:MGI:1891824]	725.3	682.5	734.3	**-1.13**	0.0424	**1.16**	0.0148

Zmym5	zinc finger, MYM-type 5 [Source:MGI Symbol;Acc:MGI:3041170]	509.4	459.5	512.0	**-1.13**	0.0454	**1.19**	0.0042
Srsf11	serine/arginine-rich splicing factor 11 [Source:MGI Symbol;Acc:MGI:1916457]	581.4	562.3	608.8	**-1.12**	0.0355	**1.19**	0.0001
