# Supplementary material for: Effects of Lingonberry (Vaccinium vitis-idaea L.) Supplementation on Hepatic Gene Expression in High-Fat Diet Fed Mice
Source: Nutrients. 2021 Oct 21;13(11):3693. doi: 10.3390/nu13113693 (PMC8623941; doi:10.3390/nu13113693)
Supplement: Supplementary file 1 [file nutrients-13-03693-s001.zip › Table S7.pdf]

Table S7: Genes associated with inflammation and metabolism validated with PCR.

		High-fat (HF) diet vs. low-fat (LF) diet				Lingonberry- supplemented high-fat diet (HF+LGB) vs. high-fat (HF) diet			
**Gene**	**Name**	**FC (NGS)**	**p-value[1] (NGS)**	**FC (PCR)**	**p-value[2] (PCR)**	**FC (NGS)**	**p-value[1] (NGS)**	**FC (PCR)**	**p-value[2] (PCR)**
TNF-α	Tumor necrosis factor	**1.13**	0.68	**1.00**	0.99	**1.15**	0.73	**-1.05**	0.81
Slc2a2	Solute carrier family 2 member 2	**-1.15**	0.21	**-1.20**	0.09	**-1.04**	0.90	**1.04**	0.79
Il-1b	Interleukin 1 beta	**1.20**	0.60	**1.01**	0.96	**-1.03**	0.94	**1.18**	0.55
Cd68	CD68 antigen	**-1.08**	0.80	**-1.02**	0.86	**-1.02**	0.95	**1.19**	0.26
Saa2	Serum amyloid A 2	**1.60**	0.0030	**2.18**	0.0091	**-1.83**	2.03E-05	**-2.05**	0.011
Cxcl14	Chemokine (C-X-C motif) ligand 14	**1.60**	0.0016	**1.41**	0.092	**-1.74**	0.00019	**-2.44**	0.016
Lepr	Leptin receptor	**-3.48**	3.89E-24	**-9.43**	0.0019	**1.01**	0.94	**1.00**	0.99
Igfbp2	Insulin-like growth factor-binding protein 2	**-1.95**	1.38E-11	**-1.35**	0.033	**1.71**	7.44E-09	**2.13**	0.012
Pparg	Peroxisome proliferator activated receptor gamma	**1.72***	8.36E-06*			**-1.07***	0.90*		
Pparg_var1	peroxisome proliferator activated receptor gamma_variant1	*	*	**2.07**	< 0.0001	*	*	**2.28**	0.5594
Pparg_var2	peroxisome proliferator activated receptor gamma_variant2	*	*	**4.72**	< 0.0001	*	*	**-1.05**	> 0.9999
Cd36	CD36 molecule	**1.73**	3.28E-07	**2.05**	0.0008	**-1.40**	0.01047	**-1.39**	0.0673
Cidec	cell death-inducing DFFA-like effector c	**1.72**	2.90E-06	**3.57**	0.0019	**-1.30**	0.165	**-1.82**	0.0532
Mogat1	monoacylglycerol O-acyltransferase 1	**2.51**	1.51E-13	**3.18**	< 0.0001	**-1.69**	0.00027	**-1.90**	0.0003

Red denotes upregulation and blue downregulation. [1] p-values are adjusted by false discovery rate (FDR). [2]p-values are adjusted with Bonferroni correction.
*NGS was unable to distinguish between the Pparg isoforms.
