# Supplementary material for: Effects of Lingonberry (Vaccinium vitis-idaea L.) Supplementation on Hepatic Gene Expression in High-Fat Diet Fed Mice
Source: Nutrients. 2021 Oct 21;13(11):3693. doi: 10.3390/nu13113693 (PMC8623941; doi:10.3390/nu13113693)
Supplement: Supplementary file 1 [file nutrients-13-03693-s001.zip › Table S8.pdf]

Table S8. All significantly differentially expressed genes belonging to the significantly enriched GO terms in the high-fat (HF) diet group compared to the lingonberry-supplemented high-fat (HF+LGB) diet group. p-values are adjusted by false discovery rate (FDR). GO = Gene ontology.

GO Term	Description	p-value (FRD adj.)	Genes
GO: 0044283	small molecule biosynthetic process	1.02E-06	[Gpd2 - glycerol phosphate dehydrogenase 2, mitochondrial, Cyp39a1 - cytochrome p450, family 39, subfamily a, polypeptide 1, Ctps - cytidine 5'-triphosphate synthase, Gpi1 - glucose phosphate isomerase 1, Crtc2 - creb regulated transcription coactivator 2, Agxt - alanine-glyoxylate aminotransferase, Entpd2 - ectonucleoside triphosphate diphosphohydrolase 2, Pgp - phosphoglycolate phosphatase, Bcat2 - branched chain aminotransferase 2, mitochondrial, Ip6k2 - inositol hexaphosphate kinase 2, Aass - aminoadipate-semialdehyde synthase, Ptafr - platelet-activating factor receptor, Got1 - glutamate oxaloacetate transaminase 1, soluble, Pde5a - phosphodiesterase 5a, cgmp-specific, Acly - atp citrate lyase, Gch1 - gtp cyclohydrolase 1, Acacb - acetyl-coenzyme a carboxylase beta, Ptgds - prostaglandin d2 synthase (brain), Cyp7a1 - cytochrome p450, family 7, subfamily a, polypeptide 1, Elovl5 - elovl family member 5, elongation of long chain fatty acids (yeast), Elovl6 - elovl family member 6, elongation of long chain fatty acids (yeast), Itpkc - inositol 1,4,5-trisphosphate 3-kinase c, Acot11 - acyl-coa thioesterase 11, Dnph1 - 2'-deoxynucleoside 5'-phosphate n-hydrolase 1, Ass1 - argininosuccinate synthetase 1, Kdm3a - lysine (k)-specific demethylase 3a, Fads6 - fatty acid desaturase domain family, member 6, Plcd3 - phospholipase c, delta 3, Cth - cystathionase (cystathionine gamma-lyase), Ptges - prostaglandin e synthase, Gm5424 - argininosuccinate synthase pseudogene, Dhtkd1 - dehydrogenase e1 and transketolase domain containing 1, Asns - asparagine synthetase, Pck1 - phosphoenolpyruvate carboxykinase 1, cytosolic, Glul - glutamate-ammonia ligase (glutamine synthetase), Brca1 - breast cancer 1, Upp2 - uridine phosphorylase 2, Khk - ketohexokinase, Sds - serine dehydratase, Cbs - cystathionine beta-synthase, Cps1 - carbamoyl-phosphate synthetase 1, Hyal1 - hyaluronoglucosaminidase 1, Abat - 4-aminobutyrate aminotransferase, Atf3 - activating transcription factor 3, Gck - glucokinase, Acss2 - acyl-coa synthetase short-chain family member 2, Gm3839 - glyceraldehyde-3-phosphate dehydrogenase pseudogene, Gale - galactose-4-epimerase, udp, Insig2 - insulin induced gene 2, Nme1 - nme/nm23 nucleoside diphosphate kinase 1, Gls2 - glutaminase 2 (liver, mitochondrial), Ppip5k1 - diphosphoinositol pentakisphosphate kinase 1, G6pdx - glucose-6-phosphate dehydrogenase x-linked, Pgd - phosphogluconate dehydrogenase, Pklr - pyruvate kinase liver and red blood cell, Cyp1a1 - cytochrome p450, family 1, subfamily a, polypeptide 1, Pycr1 - pyrroline-5-carboxylate reductase 1, Fdps - farnesyl diphosphate synthetase, Srd5a2 - steroid 5 alpha-reductase 2, Apoa4 - apolipoprotein a-iv, Acaca - acetyl-coenzyme a carboxylase alpha, Csad - cysteine sulfinic acid decarboxylase]
GO: 0044281	small molecule metabolic process	5.47E-07	[Fasn - fatty acid synthase, Ptgis - prostaglandin i2 (prostacyclin) synthase, Cyp46a1 - cytochrome p450, family 46, subfamily a, polypeptide 1, Cyp2j6 - cytochrome p450, family 2, subfamily j, polypeptide 6, Cyp3a13 - cytochrome p450, family 3, subfamily a, polypeptide 13, Cbr3 - carbonyl reductase 3, Hmgcs1 - 3-hydroxy-3-methylglutaryl-coenzyme a synthase 1, Cyp2g1 - cytochrome p450, family 2, subfamily g, polypeptide 1, Ahcy - s-adenosylhomocysteine hydrolase, Ptafr - platelet-activating factor receptor, Nudt4 - nudix (nucleoside diphosphate linked moiety x)-type motif 4, Pde5a - phosphodiesterase 5a, cgmp-specific, Pcsk9 - proprotein convertase subtilisin/kexin type 9, Urad - ureidoimidazoline (2-oxo-4-hydroxy-4-carboxy-5) decarboxylase, Ptgds - prostaglandin d2 synthase (brain), Cyp7a1 - cytochrome p450, family 7, subfamily a, polypeptide 1, Abhd2 - abhydrolase domain containing 2, Dck - deoxycytidine kinase, Dcn - decorin, Asl - argininosuccinate lyase, Kdm3a - lysine (k)-specific demethylase 3a, Rpia - ribose 5-phosphate isomerase a, Psat1 - phosphoserine aminotransferase 1, Plcd3 - phospholipase c, delta 3, Fabp2 - fatty acid binding protein 2, intestinal, Serp1 - stress-associated endoplasmic reticulum protein 1, Gm5424 - argininosuccinate synthase pseudogene, Stat5a - signal transducer and activator of transcription 5a, Hal - histidine ammonia lyase, Dct - dopachrome tautomerase, Sdsl - serine dehydratase-like, Pdhb - pyruvate dehydrogenase (lipoamide) beta, Cbs - cystathionine beta-synthase, Cps1 - carbamoyl-phosphate synthetase 1, Rrm2 - ribonucleotide reductase m2, Nr4a3 - nuclear receptor subfamily 4, group a, member 3, Cyp2c55 - cytochrome p450, family 2, subfamily c, polypeptide 55, Tbxas1 - thromboxane a synthase 1, platelet, Ltc4s - leukotriene c4 synthase, Dpyd - dihydropyrimidine dehydrogenase, Sptlc2 - serine palmitoyltransferase, long chain base subunit 2, Scarb1 - scavenger receptor class b, member 1, Acnat2 -

			acyl-coenzyme a amino acid n-acyltransferase 2, Nme1 - nme/nm23 nucleoside diphosphate kinase 1, Cyp4a31 - cytochrome p450, family 4, subfamily a, polypeptide 31, Cyp4a32 - cytochrome p450, family 4, subfamily a, polypeptide 32, Cyp1a1 - cytochrome p450, family 1, subfamily a, polypeptide 1, Pycr1 - pyrroline-5-carboxylate reductase 1, Uap1l1 - udp-n-acteylglucosamine pyrophosphorylase 1-like 1, Cyp2c37 - cytochrome p450, family 2. subfamily c, polypeptide 37, Cyp2c29 - cytochrome p450, family 2, subfamily c, polypeptide 29, Cyp2s1 - cytochrome p450, family 2, subfamily s, polypeptide 1, Afp - alpha fetoprotein, Cyp2b9 - cytochrome p450, family 2, subfamily b, polypeptide 9, Acaca - acetyl-coenzyme a carboxylase alpha, Lpin3 - lipin 3, Csad - cysteine sulfinic acid decarboxylase, Cyp2b13 - cytochrome p450, family 2, subfamily b, polypeptide 13, Angpt1 - angiopoietin 1, Cyp2b10 - cytochrome p450, family 2, subfamily b, polypeptide 10, Tkt - transketolase, Scd1 - stearoyl-coenzyme a desaturase 1, Tk1 - thymidine kinase 1, Qrsl1 - glutaminyl-trna synthase (glutamine-hydrolyzing)-like 1, Cyp39a1 - cytochrome p450, family 39, subfamily a, polypeptide 1, Acot1 - acyl-coa thioesterase 1, Pgm2 - phosphoglucomutase 2, Acat2 - acetyl-coenzyme a acetyltransferase 2, Ctps - cytidine 5'-triphosphate synthase, Entpd2 - ectonucleoside triphosphate diphosphohydrolase 2, Agxt - alanine-glyoxylate aminotransferase, Mogat1 - monoacylglycerol o-acyltransferase 1, Cln6 - ceroid-lipofuscinosis, neuronal 6, Pdss1 - prenyl (solanesyl) diphosphate synthase, subunit 1, Ip6k2 - inositol hexaphosphate kinase 2, Mthfd1l - methylenetetrahydrofolate dehydrogenase (nadp+ dependent) 1-like, Saa1 - serum amyloid a 1, Acot11 - acyl-coa thioesterase 11, Vnn1 - vanin 1, Tnfrsf1a - tumor necrosis factor receptor superfamily, member 1a, Vldlr - very low density lipoprotein receptor, Acsm5 - acyl-coa synthetase medium-chain family member 5, Akr1c20 - aldo-keto reductase family 1, member c20, Cyb5r3 - cytochrome b5 reductase 3, Lrp1 - low density lipoprotein receptor-related protein 1, Wars - tryptophanyl-trna synthetase, Cd36 - cd36 antigen, Dlat - dihydrolipoamide s-acetyltransferase (e2 component of pyruvate dehydrogenase complex), Ampd3 - adenosine monophosphate deaminase 3, Pla2g4f - phospholipase a2, group ivf, Enpp3 - ectonucleotide pyrophosphatase/phosphodiesterase 3, G6pc3 - glucose 6 phosphatase, catalytic, 3, Dhtkd1 - dehydrogenase e1 and transketolase domain containing 1, Asns - asparagine synthetase, Acadsb - acyl-coenzyme a dehydrogenase, short/branched chain, Gne - glucosamine (udp-n-acetyl)-2-epimerase/n-acetylmannosamine kinase, Akr1c18 - aldo-keto reductase family 1, member c18, Upp2 - uridine phosphorylase 2, Sds - serine dehydratase, Gucy2c - guanylate cyclase 2c, Fabp4 - fatty acid binding protein 4, adipocyte, Gm3839 - glyceraldehyde-3-phosphate dehydrogenase pseudogene, Aox1 - aldehyde oxidase 1, Fuca2 - fucosidase, alpha-l- 2, plasma, Synj2 - synaptojanin 2, Agpat6 - 1-acylglycerol-3-phosphate o-acyltransferase 6 (lysophosphatidic acid acyltransferase, zeta), Pdk4 - pyruvate dehydrogenase kinase, isoenzyme 4, Atp1a2 - atpase, na+/k+ transporting, alpha 2 polypeptide, Ppat - phosphoribosyl pyrophosphate amidotransferase, Nags - n-acetylglutamate synthase, Acpp - acid phosphatase, prostate, Cyp2c68 - cytochrome p450, family 2, subfamily c, polypeptide 68, Slc35d1 - solute carrier family 35 (udp-glucuronic acid/udp-n-acetylgalactosamine dual transporter), member d1, Apoa4 - apolipoprotein a-iv, Lcat - lecithin cholesterol acyltransferase, Mrps36 - mitochondrial ribosomal protein s36, Acsl5 - acyl-coa synthetase long-chain family member 5, Nudt11 - nudix (nucleoside diphosphate linked moiety x)-type motif 11, Slc5a3 - solute carrier family 5 (inositol transporters), member 3, Cyp2j9 - cytochrome p450, family 2, subfamily j, polypeptide 9, Gpd2 - glycerol phosphate dehydrogenase 2, mitochondrial, Pgp - phosphoglycolate phosphatase, Aass - aminoadipate-semialdehyde synthase, Ucp3 - uncoupling protein 3 (mitochondrial, proton carrier), Apip - apaf1 interacting protein, Gba2 - glucosidase beta 2, Dpm1 - dolichol-phosphate (beta-d) mannosyltransferase 1, Afmid - arylformamidase, Macrod2 - macro domain containing 2, Ivd - isovaleryl coenzyme a dehydrogenase, Gch1 - gtp cyclohydrolase 1, Sardh - sarcosine dehydrogenase, Acot9 - acyl-coa thioesterase 9, Dnph1 - 2'-deoxynucleoside 5'-phosphate n-hydrolase 1, Fabp5 - fatty acid binding protein 5, epidermal, Nudt1 - nudix (nucleoside diphosphate linked moiety x)-type motif 1, Mthfd2 - methylenetetrahydrofolate dehydrogenase (nad+ dependent), methenyltetrahydrofolate cyclohydrolase, Ass1 - argininosuccinate synthetase 1, Gpd1l - glycerol-3-phosphate dehydrogenase 1-like, Pla2g16 - phospholipase a2, group xvi, Cyp2c67 - cytochrome p450, family 2, subfamily c, polypeptide 67, Cth - cystathionase (cystathionine gamma-lyase), Adrbk2 - adrenergic receptor kinase, beta 2, Gapdhs - glyceraldehyde-3-phosphate dehydrogenase, spermatogenic, Dhrs7b - dehydrogenase/reductase (sdr family) member 7b, Gapdh - glyceraldehyde-3-phosphate dehydrogenase, Atp2b2 - atpase, ca++ transporting, plasma membrane 2, Pck1 - phosphoenolpyruvate carboxykinase 1, cytosolic, Haghl - hydroxyacylglutathione hydrolase-like, Hyal1 - hyaluronoglucosaminidase 1, Atf3 - activating transcription factor 3, Acss2 - acyl-coa

			synthetase short-chain family member 2, Gck - glucokinase, Cnp - 2',3'-cyclic nucleotide 3' phosphodiesterase, Ugp2 - udp-glucose pyrophosphorylase 2, Pltp - phospholipid transfer protein, Insig2 - insulin induced gene 2, Gls2 - glutaminase 2 (liver, mitochondrial), Pmp22 - peripheral myelin protein 22, Pah - phenylalanine hydroxylase, G6pdx - glucose-6-phosphate dehydrogenase x-linked, Aldob - aldolase b, fructose-bisphosphate, Tat - tyrosine aminotransferase, Pgd - phosphogluconate dehydrogenase, Abcc2 - atp-binding cassette, sub-family c (cftr/mrp), member 2, Pklr - pyruvate kinase liver and red blood cell, Amdhd1 - amidohydrolase domain containing 1, Abcd2 - atp-binding cassette, sub-family d (ald), member 2, Cyp2c54 - cytochrome p450, family 2, subfamily c, polypeptide 54, Fdps - farnesyl diphosphate synthetase, Srd5a2 - steroid 5 alpha-reductase 2, Pnpla3 - patatin-like phospholipase domain containing 3, Aox3 - aldehyde oxidase 3, Atp5k - atp synthase, h+ transporting, mitochondrial f1f0 complex, subunit e, Acmsd - amino carboxymuconate semialdehyde decarboxylase, Plcd1 - phospholipase c, delta 1, Gpam - glycerol-3-phosphate acyltransferase, mitochondrial, Cyp4f14 - cytochrome p450, family 4, subfamily f, polypeptide 14, Gpi1 - glucose phosphate isomerase 1, Crtc2 - creb regulated transcription coactivator 2, Bcat2 - branched chain aminotransferase 2, mitochondrial, Gnmt - glycine n-methyltransferase, P4ha2 - procollagen-proline, 2-oxoglutarate 4-dioxygenase (proline 4-hydroxylase), alpha ii polypeptide, Dpys - dihydropyrimidinase, Mtmr7 - myotubularin related protein 7, Got1 - glutamate oxaloacetate transaminase 1, soluble, Entpd7 - ectonucleoside triphosphate diphosphohydrolase 7, Adcy3 - adenylate cyclase 3, Acly - atp citrate lyase, Lrat - lecithin-retinol acyltransferase (phosphatidylcholine-retinol-o-acyltransferase), Acacb - acetyl-coenzyme a carboxylase beta, Elovl5 - elovl family member 5, elongation of long chain fatty acids (yeast), Elovl6 - elovl family member 6, elongation of long chain fatty acids (yeast), Itpkc - inositol 1,4,5-trisphosphate 3-kinase c, Me1 - malic enzyme 1, nadp(+)-dependent, cytosolic, Impa2 - inositol (myo)-1(or 4)-monophosphatase 2, Nme6 - nme/nm23 nucleoside diphosphate kinase 6, Sorl1 - sortilin-related receptor, ldlr class a repeats-containing, Por - p450 (cytochrome) oxidoreductase, Taldo1 - transaldolase 1, Fads6 - fatty acid desaturase domain family, member 6, Oasl2 - 2'-5' oligoadenylate synthetase-like 2, Itih2 - inter-alpha trypsin inhibitor, heavy chain 2, Ptges - prostaglandin e synthase, Papss2 - 3'-phosphoadenosine 5'-phosphosulfate synthase 2, Rhoq - ras homolog gene family, member q, Glul - glutamate-ammonia ligase (glutamine synthetase), Brca1 - breast cancer 1, Cyp2d12 - cytochrome p450, family 2, subfamily d, polypeptide 12, Khk - ketohexokinase, Mthfd1 - methylenetetrahydrofolate dehydrogenase (nadp+ dependent), methenyltetrahydrofolate cyclohydrolase, formyltetrahydrofolate synthase, Abat - 4-aminobutyrate aminotransferase, Rdh11 - retinol dehydrogenase 11, Mecp2 - methyl cpg binding protein 2, Prkab2 - protein kinase, amp-activated, beta 2 non-catalytic subunit, Gale - galactose-4-epimerase, udp, Ttr - transthyretin, Ppip5k1 - diphosphoinositol pentakisphosphate kinase 1, Cs - citrate synthase, C3 - complement component 3, Cryl1 - crystallin, lambda 1, Slc2a9 - solute carrier family 2 (facilitated glucose transporter), member 9, Hacl1 - 2-hydroxyacyl-coa lyase 1, Hpd - 4-hydroxyphenylpyruvic acid dioxygenase, Abcg2 - atp-binding cassette, sub-family g (white), member 2, Gusb - glucuronidase, beta, Tlr2 - toll-like receptor 2, Tet2 - tet methylcytosine dioxygenase 2]
GO: 0006629	lipid metabolic process	4.17E-05	[Plscr1 - phospholipid scramblase 1, Cyp3a25 - cytochrome p450, family 3, subfamily a, polypeptide 25, Cyp46a1 - cytochrome p450, family 46, subfamily a, polypeptide 1, Acot1 - acyl-coa thioesterase 1, Plcxd1 - phosphatidylinositol-specific phospholipase c, x domain containing 1, Cyp3a11 - cytochrome p450, family 3, subfamily a, polypeptide 11, Pgp - phosphoglycolate phosphatase, Slc16a11 - solute carrier family 16 (monocarboxylic acid transporters), member 11, Mogat1 - monoacylglycerol o-acyltransferase 1, Cln6 - ceroid-lipofuscinosis, neuronal 6, Cyp2g1 - cytochrome p450, family 2, subfamily g, polypeptide 1, Hsd17b6 - hydroxysteroid (17-beta) dehydrogenase 6, Ttc39b - tetratricopeptide repeat domain 39b, Ivd - isovaleryl coenzyme a dehydrogenase, Acly - atp citrate lyase, Ptgds - prostaglandin d2 synthase (brain), Saa1 - serum amyloid a 1, Cyp7a1 - cytochrome p450, family 7, subfamily a, polypeptide 1, Elovl5 - elovl family member 5, elongation of long chain fatty acids (yeast), Acot11 - acyl-coa thioesterase 11, Vldlr - very low density lipoprotein receptor, Fabp5 - fatty acid binding protein 5, epidermal, Smpd3 - sphingomyelin phosphodiesterase 3, neutral, Cidea - cell death-inducing dna fragmentation factor, alpha subunit-like effector a, Pik3c2g - phosphatidylinositol 3-kinase, c2 domain containing, gamma polypeptide, Por - p450 (cytochrome) oxidoreductase, Cd36 - cd36 antigen, Pik3r3 - phosphatidylinositol 3 kinase, regulatory subunit, polypeptide 3 (p55), Plcd3 - phospholipase c, delta 3, Ang - angiogenin, ribonuclease, rnase a family, 5, Ptges - prostaglandin e

			synthase, Cyp3a59 - cytochrome p450, family 3, subfamily a, polypeptide 59, Serinc2 - serine incorporator 2, Ces1b - carboxylesterase 1b, Brca1 - breast cancer 1, Cps1 - carbamoyl-phosphate synthetase 1, 9130409I23Rik - riken cdna 9130409i23 gene, Sccpdh - saccharopine dehydrogenase (putative), Cyp2c55 - cytochrome p450, family 2, subfamily c, polypeptide 55, Ces4a - carboxylesterase 4a, Insig2 - insulin induced gene 2, Acnat2 - acyl-coenzyme a amino acid n-acyltransferase 2, C3 - complement component 3, Cryl1 - crystallin, lambda 1, G6pdx - glucose-6-phosphate dehydrogenase x-linked, Synj2 - synaptojanin 2, Cyp1a1 - cytochrome p450, family 1, subfamily a, polypeptide 1, Cyp2c54 - cytochrome p450, family 2, subfamily c, polypeptide 54, Hexa - hexosaminidase a, Vac14 - vac14 homolog (s. cerevisiae), Srd5a2 - steroid 5 alpha-reductase 2, Cyp2c29 - cytochrome p450, family 2, subfamily c, polypeptide 29, Pnpla3 - patatin-like phospholipase domain containing 3, Apoa4 - apolipoprotein a-iv, Ces1e - carboxylesterase 1e, Akr1c19 - aldo-keto reductase family 1, member c19, Cyp2b13 - cytochrome p450, family 2, subfamily b, polypeptide 13, Cyp2b10 - cytochrome p450, family 2, subfamily b, polypeptide 10, Plekha1 - pleckstrin homology domain containing, family a (phosphoinositide binding specific) member 1]
GO: 0010033	response to organic substance	1.45E-03	[Fasn - fatty acid synthase, Dll4 - delta-like 4 (drosophila), Ntrk1 - neurotrophic tyrosine kinase, receptor, type 1, Gsn - gelsolin, Prdm2 - pr domain containing 2, with znf domain, Capg - capping protein (actin filament), gelsolin-like, H2-Q7 - histocompatibility 2, q region locus 7, Ntrk2 - neurotrophic tyrosine kinase, receptor, type 2, Abl2 - v-abl abelson murine leukemia viral oncogene 2 (arg, abelson-related gene), Nfat5 - nuclear factor of activated t cells 5, Tmem38a - transmembrane protein 38a, Hmgcs1 - 3-hydroxy-3-methylglutaryl-coenzyme a synthase 1, Pde3a - phosphodiesterase 3a, cgmp inhibited, Ptafr - platelet-activating factor receptor, Adam23 - a disintegrin and metallopeptidase domain 23, Stx4a - syntaxin 4a (placental), Ptch1 - patched homolog 1, Ace - angiotensin i converting enzyme (peptidyl-dipeptidase a) 1, Pcsk9 - proprotein convertase subtilisin/kexin type 9, Snx10 - sorting nexin 10, Ankzf1 - ankyrin repeat and zinc finger domain containing 1, Derl3 - der1-like domain family, member 3, Lamtor4 - late endosomal/lysosomal adaptor, mapk and mtor activator 4, Cyp7a1 - cytochrome p450, family 7, subfamily a, polypeptide 1, Abhd2 - abhydrolase domain containing 2, F2r - coagulation factor ii (thrombin) receptor, P2rx3 - purinergic receptor p2x, ligand-gated ion channel, 3, Zfp36 - zinc finger protein 36, Hba-a1 - hemoglobin alpha, adult chain 1, Pdgfc - platelet-derived growth factor, c polypeptide, Actb - actin, beta, Cacnb4 - calcium channel, voltage-dependent, beta 4 subunit, Dntt - deoxynucleotidyltransferase, terminal, Kdm3a - lysine (k)-specific demethylase 3a, Ada - adenosine deaminase, Stat5a - signal transducer and activator of transcription 5a, Npc1 - niemann pick type c1, Acvr2b - activin receptor iib, Tiparp - tcdd-inducible poly(adp-ribose) polymerase, Runx3 - runt related transcription factor 3, Ly6d - lymphocyte antigen 6 complex, locus d, Cbs - cystathionine beta-synthase, Cps1 - carbamoyl-phosphate synthetase 1, Nr4a3 - nuclear receptor subfamily 4, group a, member 3, Dpyd - dihydropyrimidine dehydrogenase, Crebrf - creb3 regulatory factor, Scarb1 - scavenger receptor class b, member 1, Src - rous sarcoma oncogene, Rufy4 - run and fyve domain containing 4, Casp4 - caspase 4, apoptosis-related cysteine peptidase, Cyp1a1 - cytochrome p450, family 1, subfamily a, polypeptide 1, Smad3 - smad family member 3, Smad9 - smad family member 9, Insr - insulin receptor, Manf - mesencephalic astrocyte-derived neurotrophic factor, Smad7 - smad family member 7, Ly96 - lymphocyte antigen 96, Cyp2b9 - cytochrome p450, family 2, subfamily b, polypeptide 9, Esr1 - estrogen receptor 1 (alpha), Rragd - ras-related gtp binding d, Ptp4a3 - protein tyrosine phosphatase 4a3, Acaca - acetyl-coenzyme a carboxylase alpha, Lpin3 - lipin 3, P2ry4 - pyrimidinergic receptor p2y, g-protein coupled, 4, Cyp2a5 - cytochrome p450, family 2, subfamily a, polypeptide 5, Scd1 - stearoyl-coenzyme a desaturase 1, Agtr1a - angiotensin ii receptor, type 1a, Derl2 - der1-like domain family, member 2, Entpd2 - ectonucleoside triphosphate diphosphohydrolase 2, Agxt - alanine-glyoxylate aminotransferase, Ramp1 - receptor (calcitonin) activity modifying protein 1, Cd9 - cd9 antigen, Ip6k2 - inositol hexaphosphate kinase 2, Lifr - leukemia inhibitory factor receptor, Rftn2 - raftlin family member 2, Cd83 - cd83 antigen, Timp1 - tissue inhibitor of metalloproteinase 1, Wnt5a - wingless-related mmtv integration site 5a, Smo - smoothened homolog (drosophila), Rangap1 - ran gtpase activating protein 1, Timp2 - tissue inhibitor of metalloproteinase 2, Cd81 - cd81 antigen, Ddit4 - dna-damage-inducible transcript 4, Saa2 - serum amyloid a 2, Saa3 - serum amyloid a 3, Wt1 - wilms tumor 1 homolog, Fbxo2 - f-box protein 2, Rab34 - rab34, member of ras oncogene family, Fbxo44 - f-box protein 44, Ccl2 - chemokine (c-c motif) ligand 2, Hspa5 - heat shock protein 5, Vim - vimentin, Pcolce2 - procollagen c-endopeptidase enhancer 2, Kdm5b - lysine (k)-specific demethylase 5b, Lsp1 - lymphocyte specific 1, Mapkapk3 - mitogen-activated

			protein kinase-activated protein kinase 3, Lrp6 - low density lipoprotein receptor-related protein 6, Gsta2 - glutathione s-transferase, alpha 2 (yc2), Lrp1 - low density lipoprotein receptor-related protein 1, Ifi205 - interferon activated gene 205, Anxa1 - annexin a1, Cd36 - cd36 antigen, Ang - angiogenin, ribonuclease, rnase a family, 5, Pla2g4f - phospholipase a2, group ivf, Slc30a10 - solute carrier family 30, member 10, Cpeb4 - cytoplasmic polyadenylation element binding protein 4, Steap2 - six transmembrane epithelial antigen of prostate 2, Rif1 - rap1 interacting factor 1 homolog (yeast), Vmn1r53 - vomeronasal 1 receptor 53, Sec61b - sec61 beta subunit, Stk39 - serine/threonine kinase 39, Akr1c18 - aldo-keto reductase family 1, member c18, Anxa5 - annexin a5, Slc25a5 - solute carrier family 25 (mitochondrial carrier, adenine nucleotide translocator), member 5, Cxcl2 - chemokine (c-x-c motif) ligand 2, Cd300lb - cd300 antigen like family member b, Ccl9 - chemokine (c-c motif) ligand 9, Fabp4 - fatty acid binding protein 4, adipocyte, Plscr4 - phospholipid scramblase 4, Dnajc10 - dnaj (hsp40) homolog, subfamily c, member 10, Cxcl12 - chemokine (c-x-c motif) ligand 12, Gstt2 - glutathione s-transferase, theta 2, Ccl6 - chemokine (c-c motif) ligand 6, Zfp259 - zinc finger protein 259, Il6ra - interleukin 6 receptor, alpha, Cdh1 - cadherin 1, Ocstamp - osteoclast stimulatory transmembrane protein, Pdk4 - pyruvate dehydrogenase kinase, isoenzyme 4, Atp1a2 - atpase, na+/k+ transporting, alpha 2 polypeptide, Epb4.1l5 - erythrocyte protein band 4.1-like 5, Slc26a6 - solute carrier family 26, member 6, Apoa4 - apolipoprotein a-iv, Mup3 - major urinary protein 3, Lcat - lecithin cholesterol acyltransferase, Thbs1 - thrombospondin 1, Uchl3 - ubiquitin carboxyl-terminal esterase l3 (ubiquitin thiolesterase), Ar - androgen receptor, Adamts12 - a disintegrin-like and metallopeptidase (reprolysin type) with thrombospondin type 1 motif, 12, Hsd3b2 - hydroxy-delta-5-steroid dehydrogenase, 3 beta- and steroid delta-isomerase 2, Unc13b - unc-13 homolog b (c. elegans), Hspb1 - heat shock protein 1, Prkca - protein kinase c, alpha, Prkce - protein kinase c, epsilon, Sh3bp4 - sh3-domain binding protein 4, Aqp4 - aquaporin 4, Hlcs - holocarboxylase synthetase (biotin- [propriony-coenzyme a-carboxylase (atp-hydrolysing)] ligase), Gch1 - gtp cyclohydrolase 1, Prpf8 - pre-mrna processing factor 8, Pde3b - phosphodiesterase 3b, cgmp-inhibited, Tbc1d7 - tbc1 domain family, member 7, Trim2 - tripartite motif-containing 2, Gata6 - gata binding protein 6, Gata5 - gata binding protein 5, Kit - kit oncogene, Mapt - microtubule-associated protein tau, Smpd3 - sphingomyelin phosphodiesterase 3, neutral, Cidea - cell death-inducing dna fragmentation factor, alpha subunit-like effector a, Ass1 - argininosuccinate synthetase 1, Pdgfrb - platelet derived growth factor receptor, beta polypeptide, Hspa13 - heat shock protein 70 family, member 13, Ppp2r5b - protein phosphatase 2, regulatory subunit b (b56), beta isoform, Gas6 - growth arrest specific 6, Mapk15 - mitogen-activated protein kinase 15, Amigo1 - adhesion molecule with ig like domain 1, Irs2 - insulin receptor substrate 2, Cth - cystathionase (cystathionine gamma-lyase), Akap8 - a kinase (prka) anchor protein 8, Fbxo6 - f-box protein 6, Slc6a4 - solute carrier family 6 (neurotransmitter transporter, serotonin), member 4, Gapdh - glyceraldehyde-3-phosphate dehydrogenase, Mras - muscle and microspikes ras, Ikbke - inhibitor of kappab kinase epsilon, Pck1 - phosphoenolpyruvate carboxykinase 1, cytosolic, Icam1 - intercellular adhesion molecule 1, Col3a1 - collagen, type iii, alpha 1, Plscr2 - phospholipid scramblase 2, Vegfa - vascular endothelial growth factor a, Col4a2 - collagen, type iv, alpha 2, Trem2 - triggering receptor expressed on myeloid cells 2, Slc27a1 - solute carrier family 27 (fatty acid transporter), member 1, Pcolce - procollagen c-endopeptidase enhancer protein, Hyal1 - hyaluronoglucosaminidase 1, Gabrb3 - gamma-aminobutyric acid (gaba) a receptor, subunit beta 3, Gck - glucokinase, Cxcl10 - chemokine (c-x-c motif) ligand 10, Gab1 - growth factor receptor bound protein 2-associated protein 1, Pmp22 - peripheral myelin protein 22, Ccr7 - chemokine (c-c motif) receptor 7, Ccr5 - chemokine (c-c motif) receptor 5, G6pdx - glucose-6-phosphate dehydrogenase x-linked, Aldob - aldolase b, fructose-bisphosphate, Tat - tyrosine aminotransferase, Pklr - pyruvate kinase liver and red blood cell, Fzd5 - frizzled homolog 5 (drosophila), Ifit2 - interferon-induced protein with tetratricopeptide repeats 2, Srd5a2 - steroid 5 alpha-reductase 2, Dtx1 - deltex 1 homolog (drosophila), Serpine1 - serine (or cysteine) peptidase inhibitor, clade e, member 1, Socs2 - suppressor of cytokine signaling 2, Cib2 - calcium and integrin binding family member 2, Plcd1 - phospholipase c, delta 1, Plscr1 - phospholipid scramblase 1, Med1 - mediator complex subunit 1, Gpam - glycerol-3-phosphate acyltransferase, mitochondrial, Sparc - secreted acidic cysteine rich glycoprotein, Asxl1 - additional sex combs like 1, Sp5 - trans-acting transcription factor 5, Eno1 - enolase 1, alpha non-neuron, Irgm2 - immunity-related gtpase family m member 2, Gpi1 - glucose phosphate isomerase 1, Bcat2 - branched chain aminotransferase 2, mitochondrial, Serpina1e - serine (or cysteine) peptidase inhibitor, clade a, member 1e, Mmp2 - matrix metallopeptidase 2, Jup - junction plakoglobin, Jun - jun oncogene, Mmp13 - matrix metallopeptidase 13, Ppp1r1b - protein

			phosphatase 1, regulatory (inhibitor) subunit 1b, Bax - bcl2-associated x protein, Adcy3 - adenylate cyclase 3, Trim13 - tripartite motif-containing 13, Adnp2 - adnp homeobox 2, Lrat - lecithin-retinol acyltransferase (phosphatidylcholine-retinol-o-acyltransferase), Acacb - acetyl-coenzyme a carboxylase beta, Glrx - glutaredoxin, F830016B08Rik - riken cdna f830016b08 gene, Me1 - malic enzyme 1, nadp(+)-dependent, cytosolic, Gm4951 - predicted gene 4951, Herpud1 - homocysteine-inducible, endoplasmic reticulum stress-inducible, ubiquitin-like domain member 1, Twf2 - twinfilin, actin-binding protein, homolog 2 (drosophila), Itgb1bp1 - integrin beta 1 binding protein 1, Sox6 - sry-box containing gene 6, Por - p450 (cytochrome) oxidoreductase, Tank - traf family member-associated nf-kappa b activator, Peli1 - pellino 1, Col1a2 - collagen, type i, alpha 2, Relb - avian reticuloendotheliosis viral (v-rel) oncogene related b, Col1a1 - collagen, type i, alpha 1, Arid5b - at rich interactive domain 5b (mrf1-like), Itpr2 - inositol 1,4,5-triphosphate receptor 2, Epha4 - eph receptor a4, Epha3 - eph receptor a3, A230050P20Rik - riken cdna a230050p20 gene, Col6a1 - collagen, type vi, alpha 1, Upf2 - upf2 regulator of nonsense transcripts homolog (yeast), Rhoq - ras homolog gene family, member q, Rpl36al - ribosomal protein l36a-like, Rfx2 - regulatory factor x, 2 (influences hla class ii expression), Ednrb - endothelin receptor type b, Brca1 - breast cancer 1, Glul - glutamate-ammonia ligase (glutamine synthetase), Tuba1b - tubulin, alpha 1b, Khk - ketohexokinase, Spry2 - sprouty homolog 2 (drosophila), Bst2 - bone marrow stromal cell antigen 2, Spry4 - sprouty homolog 4 (drosophila), Ogt - o-linked n-acetylglucosamine (glcnac) transferase (udp-n-acetylglucosamine:polypeptide-n-acetylglucosaminyl transferase), Sirt1 - sirtuin 1, Adamts7 - a disintegrin-like and metallopeptidase (reprolysin type) with thrombospondin type 1 motif, 7, Trpv4 - transient receptor potential cation channel, subfamily v, member 4, Dusp10 - dual specificity phosphatase 10, Abat - 4-aminobutyrate aminotransferase, Trpm4 - transient receptor potential cation channel, subfamily m, member 4, Bmp7 - bone morphogenetic protein 7, Mecp2 - methyl cpg binding protein 2, Bmp6 - bone morphogenetic protein 6, Bmp4 - bone morphogenetic protein 4, Sdf2l1 - stromal cell-derived factor 2-like 1, Slc22a7 - solute carrier family 22 (organic anion transporter), member 7, Ocln - occludin, Ehd1 - eh-domain containing 1, Slc10a3 - solute carrier family 10 (sodium/bile acid cotransporter family), member 3, Hp - haptoglobin, Mga - max gene associated, Stap1 - signal transducing adaptor family member 1, Nr4a2 - nuclear receptor subfamily 4, group a, member 2, Btk - bruton agammaglobulinemia tyrosine kinase, Hprt - hypoxanthine guanine phosphoribosyl transferase, Tlr2 - toll-like receptor 2, Egr1 - early growth response 1, Ubd - ubiquitin d, Rapgef2 - rap guanine nucleotide exchange factor (gef) 2, Egr2 - early growth response 2]
GO: 0072330	monocarboxylic acid biosynthetic process	2.96E-03	[Acot11 - acyl-coa thioesterase 11, Pklr - pyruvate kinase liver and red blood cell, Brca1 - breast cancer 1, Sds - serine dehydratase, Khk - ketohexokinase, Cyp1a1 - cytochrome p450, family 1, subfamily a, polypeptide 1, Gpi1 - glucose phosphate isomerase 1, Agxt - alanine-glyoxylate aminotransferase, Gck - glucokinase, Gm3839 - glyceraldehyde-3-phosphate dehydrogenase pseudogene, Gale - galactose-4-epimerase, udp, Acly - atp citrate lyase, Ptges - prostaglandin e synthase, Ptgds - prostaglandin d2 synthase (brain), Cyp7a1 - cytochrome p450, family 7, subfamily a, polypeptide 1, Dhtkd1 - dehydrogenase e1 and transketolase domain containing 1, Elovl5 - elovl family member 5, elongation of long chain fatty acids (yeast), Asns - asparagine synthetase]
GO: 0035634	response to stilbenoid	4.15E-03	[Cyp2b9 - cytochrome p450, family 2, subfamily b, polypeptide 9, Cd36 - cd36 antigen, Apoa4 - apolipoprotein a-iv, Mup3 - major urinary protein 3, Ly6d - lymphocyte antigen 6 complex, locus d, Saa2 - serum amyloid a 2, Saa3 - serum amyloid a 3, Hba-a1 - hemoglobin alpha, adult chain 1, Gsta2 - glutathione s-transferase, alpha 2 (yc2), Cidea - cell death-inducing dna fragmentation factor, alpha subunit-like effector a, Cyp2a5 - cytochrome p450, family 2, subfamily a, polypeptide 5, Slc22a7 - solute carrier family 22 (organic anion transporter), member 7]
GO: 0005975	carbohydrate metabolic process	4.18E-03	[Gpd2 - glycerol phosphate dehydrogenase 2, mitochondrial, Khk - ketohexokinase, Sds - serine dehydratase, Crtc2 - creb regulated transcription coactivator 2, Gpi1 - glucose phosphate isomerase 1, Pgp - phosphoglycolate phosphatase, Mogat1 - monoacylglycerol o-acyltransferase 1, Gnmt - glycine n-methyltransferase, Gck - glucokinase, Atf3 - activating transcription factor 3, Mtmr7 - myotubularin related protein 7, Got1 - glutamate oxaloacetate transaminase 1, soluble, Gm3839 - glyceraldehyde-3-phosphate dehydrogenase pseudogene, Gale - galactose-4-epimerase, udp, Ugp2 - udp-glucose pyrophosphorylase 2, Fuca2 - fucosidase, alpha-l-2, plasma, G6pdx - glucose-6-phosphate dehydrogenase x-linked, Synj2 - synaptojanin 2, Nans - n-acetylneuraminic acid synthase (sialic acid synthase), Pklr - pyruvate kinase liver and red blood cell, Pgd - phosphogluconate dehydrogenase, Nhlrc1 - nhl repeat containing 1, Fabp5 - fatty acid binding protein 5, epidermal, Pdk4 - pyruvate dehydrogenase kinase, isoenzyme 4, Hexa - hexosaminidase a, Gyg -

			glycogenin, Dhtkd1 - dehydrogenase e1 and transketolase domain containing 1, Treh - trehalase (brush-border membrane glycoprotein), Extl1 - exostoses (multiple)-like 1]
GO: 0015849	organic acid transport	4.06E-03	[Slc38a2 - solute carrier family 38, member 2, Slco2a1 - solute carrier organic anion transporter family, member 2a1, Slc23a1 - solute carrier family 23 (nucleobase transporters), member 1, Agxt - alanine-glyoxylate aminotransferase, Slc16a11 - solute carrier family 16 (monocarboxylic acid transporters), member 11, Slc6a8 - solute carrier family 6 (neurotransmitter transporter, creatine), member 8, Slc16a13 - solute carrier family 16 (monocarboxylic acid transporters), member 13, Fabp4 - fatty acid binding protein 4, adipocyte, Ace - angiotensin i converting enzyme (peptidyl-dipeptidase a) 1, Plin2 - perilipin 2, Slc7a4 - solute carrier family 7 (cationic amino acid transporter, y+ system), member 4, Slco1a4 - solute carrier organic anion transporter family, member 1a4, Slc16a6 - solute carrier family 16 (monocarboxylic acid transporters), member 6, Aqp8 - aquaporin 8, Slc13a4 - solute carrier family 13 (sodium/sulfate symporters), member 4, Slc17a4 - solute carrier family 17 (sodium phosphate), member 4, Slc13a5 - solute carrier family 13 (sodium-dependent citrate transporter), member 5, Abcb11 - atp-binding cassette, sub-family b (mdr/tap), member 11, Slc7a2 - solute carrier family 7 (cationic amino acid transporter, y+ system), member 2, Slc13a3 - solute carrier family 13 (sodium-dependent dicarboxylate transporter), member 3, Cd36 - cd36 antigen, Anxa1 - annexin a1, Fabp2 - fatty acid binding protein 2, intestinal, Slc38a4 - solute carrier family 38, member 4, Slc6a6 - solute carrier family 6 (neurotransmitter transporter, taurine), member 6, Slc38a3 - solute carrier family 38, member 3, Slc10a2 - solute carrier family 10, member 2]
GO: 0062012	regulation of small molecule metabolic process	6.51E-03	[Brca1 - breast cancer 1, Khk - ketohexokinase, Mas1 - mas1 oncogene, Pgp - phosphoglycolate phosphatase, Gnmt - glycine n-methyltransferase, Gck - glucokinase, Wnt4 - wingless-related mmtv integration site 4, Ptafr - platelet-activating factor receptor, Ttc39b - tetratricopeptide repeat domain 39b, Serpina12 - serine (or cysteine) peptidase inhibitor, clade a (alpha-1 antiproteinase, antitrypsin), member 12, Insig2 - insulin induced gene 2, Src - rous sarcoma oncogene, Cyp7a1 - cytochrome p450, family 7, subfamily a, polypeptide 1, Elovl5 - elovl family member 5, elongation of long chain fatty acids (yeast), Me1 - malic enzyme 1, nadp(+)-dependent, cytosolic, Nupr1 - nuclear protein transcription regulator 1, Cox7a1 - cytochrome c oxidase subunit viia 1, Fabp5 - fatty acid binding protein 5, epidermal, Pdk4 - pyruvate dehydrogenase kinase, isoenzyme 4, Smpd3 - sphingomyelin phosphodiesterase 3, neutral, Por - p450 (cytochrome) oxidoreductase, Anxa1 - annexin a1, Apoa4 - apolipoprotein a-iv, Irs2 - insulin receptor substrate 2, Acmsd - amino carboxymuconate semialdehyde decarboxylase]
GO: 0033559	unsaturated fatty acid metabolic process	7.27E-03	[Akr1c18 - aldo-keto reductase family 1, member c18, Scd1 - stearoyl-coenzyme a desaturase 1, Ptgis - prostaglandin i2 (prostacyclin) synthase, Cyp2d12 - cytochrome p450, family 2, subfamily d, polypeptide 12, Cyp4f14 - cytochrome p450, family 4, subfamily f, polypeptide 14, Cyp2j6 - cytochrome p450, family 2, subfamily j, polypeptide 6, Cyp2d9 - cytochrome p450, family 2, subfamily d, polypeptide 9, Cyp2g1 - cytochrome p450, family 2, subfamily g, polypeptide 1, Cyp2c55 - cytochrome p450, family 2, subfamily c, polypeptide 55, Tbxas1 - thromboxane a synthase 1, platelet, Ptgds - prostaglandin d2 synthase (brain), Cyp4a31 - cytochrome p450, family 4, subfamily a, polypeptide 31, Tnfrsf1a - tumor necrosis factor receptor superfamily, member 1a, Cyp4a32 - cytochrome p450, family 4, subfamily a, polypeptide 32, Akr1c20 - aldo-keto reductase family 1, member c20, Cyp2c54 - cytochrome p450, family 2, subfamily c, polypeptide 54, Cyp2c37 - cytochrome p450, family 2. subfamily c, polypeptide 37, Cyp2c29 - cytochrome p450, family 2, subfamily c, polypeptide 29, Cyp2c67 - cytochrome p450, family 2, subfamily c, polypeptide 67, Cyp2b9 - cytochrome p450, family 2, subfamily b, polypeptide 9, Cyp2c68 - cytochrome p450, family 2, subfamily c, polypeptide 68, Cyp2s1 - cytochrome p450, family 2, subfamily s, polypeptide 1, Pla2g4f - phospholipase a2, group ivf, Ptges - prostaglandin e synthase, Cyp2b13 - cytochrome p450, family 2, subfamily b, polypeptide 13, Cyp2b10 - cytochrome p450, family 2, subfamily b, polypeptide 10, Cyp2a5 - cytochrome p450, family 2, subfamily a, polypeptide 5]
GO: 0042221	response to chemical	7.62E-03	[Fasn - fatty acid synthase, Dll4 - delta-like 4 (drosophila), Ntrk1 - neurotrophic tyrosine kinase, receptor, type 1, Ptgis - prostaglandin i2 (prostacyclin) synthase, Gsn - gelsolin, Prdm2 - pr domain containing 2, with znf domain, H2-Q7 - histocompatibility 2, q region locus 7, Capg - capping protein (actin filament), gelsolin-like, Ntrk2 - neurotrophic tyrosine kinase, receptor, type 2, Abl2 - v-abl abelson murine leukemia viral oncogene 2 (arg, abelson-related gene), Nfat5 - nuclear factor of activated t cells 5, Tmem38a - transmembrane protein 38a, Hmgcs1 - 3-hydroxy-3-methylglutaryl-coenzyme a synthase 1, Ahcy - s-adenosylhomocysteine hydrolase, Pde3a -

			phosphodiesterase 3a, cgmp inhibited, Adam23 - a disintegrin and metallopeptidase domain 23, Ptafr - platelet-activating factor receptor, Stx4a - syntaxin 4a (placental), Ace - angiotensin i converting enzyme (peptidyl-dipeptidase a) 1, Pcsk9 - proprotein convertase subtilisin/kexin type 9, Ptch1 - patched homolog 1, Snx10 - sorting nexin 10, Ankzf1 - ankyrin repeat and zinc finger domain containing 1, Derl3 - der1-like domain family, member 3, Lamtor4 - late endosomal/lysosomal adaptor, mapk and mtor activator 4, Cyp7a1 - cytochrome p450, family 7, subfamily a, polypeptide 1, Abhd2 - abhydrolase domain containing 2, Hyou1 - hypoxia up-regulated 1, Chrna4 - cholinergic receptor, nicotinic, alpha polypeptide 4, Nptx1 - neuronal pentraxin 1, Abcb11 - atp-binding cassette, sub-family b (mdr/tap), member 11, F2r - coagulation factor ii (thrombin) receptor, Tert - telomerase reverse transcriptase, Zfp36 - zinc finger protein 36, P2rx3 - purinergic receptor p2x, ligand-gated ion channel, 3, Hba-a1 - hemoglobin alpha, adult chain 1, Pdgfc - platelet-derived growth factor, c polypeptide, Actb - actin, beta, Cacnb4 - calcium channel, voltage-dependent, beta 4 subunit, Dntt - deoxynucleotidyltransferase, terminal, Kdm3a - lysine (k)-specific demethylase 3a, Fabp1 - fatty acid binding protein 1, liver, Ada - adenosine deaminase, Stat5a - signal transducer and activator of transcription 5a, Npc1 - niemann pick type c1, Acvr2b - activin receptor iib, Anxa2 - annexin a2, Runx3 - runt related transcription factor 3, Ly6d - lymphocyte antigen 6 complex, locus d, Cbs - cystathionine beta-synthase, Cps1 - carbamoyl-phosphate synthetase 1, Adam8 - a disintegrin and metallopeptidase domain 8, Nr4a3 - nuclear receptor subfamily 4, group a, member 3, Lrp11 - low density lipoprotein receptor-related protein 11, Crebrf - creb3 regulatory factor, Dpyd - dihydropyrimidine dehydrogenase, Scarb1 - scavenger receptor class b, member 1, Src - rous sarcoma oncogene, Rufy4 - run and fyve domain containing 4, S100a10 - s100 calcium binding protein a10 (calpactin), Casp4 - caspase 4, apoptosis-related cysteine peptidase, Cyp1a1 - cytochrome p450, family 1, subfamily a, polypeptide 1, Pycr1 - pyrroline-5-carboxylate reductase 1, Smad3 - smad family member 3, Smad9 - smad family member 9, Insr - insulin receptor, Manf - mesencephalic astrocyte-derived neurotrophic factor, Smad7 - smad family member 7, Ly96 - lymphocyte antigen 96, Cyp2b9 - cytochrome p450, family 2, subfamily b, polypeptide 9, Esr1 - estrogen receptor 1 (alpha), Rragd - ras-related gtp binding d, Ptp4a3 - protein tyrosine phosphatase 4a3, Acaca - acetyl-coenzyme a carboxylase alpha, Lpin3 - lipin 3, P2ry4 - pyrimidinergic receptor p2y, g-protein coupled, 4, Cyp2a5 - cytochrome p450, family 2, subfamily a, polypeptide 5, Lmnb1 - lamin b1, Scd1 - stearoyl-coenzyme a desaturase 1, Loxl2 - lysyl oxidase-like 2, Agtr1a - angiotensin ii receptor, type 1a, Derl2 - der1-like domain family, member 2, Entpd2 - ectonucleoside triphosphate diphosphohydrolase 2, Agxt - alanine-glyoxylate aminotransferase, Ramp1 - receptor (calcitonin) activity modifying protein 1, Ip6k2 - inositol hexaphosphate kinase 2, Lifr - leukemia inhibitory factor receptor, Rftn2 - raftlin family member 2, Wnt5a - wingless-related mmtv integration site 5a, Cd83 - cd83 antigen, Timp1 - tissue inhibitor of metalloproteinase 1, Smo - smoothened homolog (drosophila), Rangap1 - ran gtpase activating protein 1, Timp2 - tissue inhibitor of metalloproteinase 2, Cd81 - cd81 antigen, Rasgrp2 - ras, guanyl releasing protein 2, Ddit4 - dna-damage-inducible transcript 4, Saa2 - serum amyloid a 2, Saa3 - serum amyloid a 3, Sucnr1 - succinate receptor 1, Rab34 - rab34, member of ras oncogene family, Fbxo44 - f-box protein 44, Ccl2 - chemokine (c-c motif) ligand 2, Hspa5 - heat shock protein 5, Vim - vimentin, Pcolce2 - procollagen c-endopeptidase enhancer 2, Lsp1 - lymphocyte specific 1, Kdm5b - lysine (k)-specific demethylase 5b, Vldlr - very low density lipoprotein receptor, Mapkapk3 - mitogen-activated protein kinase-activated protein kinase 3, Lrp6 - low density lipoprotein receptor-related protein 6, Gsta2 - glutathione s-transferase, alpha 2 (yc2), Lrp1 - low density lipoprotein receptor-related protein 1, Ifi205 - interferon activated gene 205, Anxa1 - annexin a1, Cd36 - cd36 antigen, Ang - angiogenin, ribonuclease, rnase a family, 5, Pla2g4f - phospholipase a2, group ivf, Slc30a10 - solute carrier family 30, member 10, Cpeb4 - cytoplasmic polyadenylation element binding protein 4, Steap2 - six transmembrane epithelial antigen of prostate 2, Rif1 - rap1 interacting factor 1 homolog (yeast), Vmn1r53 - vomeronasal 1 receptor 53, Sec61b - sec61 beta subunit, Stk39 - serine/threonine kinase 39, Akr1c18 - aldo-keto reductase family 1, member c18, Anxa5 - annexin a5, Slc25a5 - solute carrier family 25 (mitochondrial carrier, adenine nucleotide translocator), member 5, Srxn1 - sulfiredoxin 1 homolog (s. cerevisiae), Gucy2c - guanylate cyclase 2c, Cpne8 - copine viii, Cxcl2 - chemokine (c-x-c motif) ligand 2, Cd300lb - cd300 antigen like family member b, Ccl9 - chemokine (c-c motif) ligand

			9, Fabp4 - fatty acid binding protein 4, adipocyte, Pcgf2 - polycomb group ring finger 2, Plscr4 - phospholipid scramblase 4, Dnajc10 - dnaj (hsp40) homolog, subfamily c, member 10, Cxcl12 - chemokine (c-x-c motif) ligand 12, Higd1a - hig1 domain family, member 1a, Gstt2 - glutathione s-transferase, theta 2, Zfp259 - zinc finger protein 259, Ccl6 - chemokine (c-c motif) ligand 6, Syt1 - synaptotagmin i, Il6ra - interleukin 6 receptor, alpha, Cdh1 - cadherin 1, Nupr1 - nuclear protein transcription regulator 1, Ocstamp - osteoclast stimulatory transmembrane protein, Pdk4 - pyruvate dehydrogenase kinase, isoenzyme 4, Atp1a2 - atpase, na+/k+ transporting, alpha 2 polypeptide, Epb4.1l5 - erythrocyte protein band 4.1-like 5, Apoa4 - apolipoprotein a-iv, Mup3 - major urinary protein 3, Lcat - lecithin cholesterol acyltransferase, Thbs1 - thrombospondin 1, Lcn2 - lipocalin 2, Uchl3 - ubiquitin carboxyl-terminal esterase l3 (ubiquitin thiolesterase), Srp19 - signal recognition particle 19, Ar - androgen receptor, Adamts12 - a disintegrin-like and metallopeptidase (reprolysin type) with thrombospondin type 1 motif, 12, Slc23a1 - solute carrier family 23 (nucleobase transporters), member 1, Hsd3b2 - hydroxy-delta-5-steroid dehydrogenase, 3 beta- and steroid delta-isomerase 2, Unc13b - unc-13 homolog b (c. elegans), Krt10 - keratin 10, Hspb1 - heat shock protein 1, Ucp3 - uncoupling protein 3 (mitochondrial, proton carrier), Prkca - protein kinase c, alpha, Sh3bp4 - sh3-domain binding protein 4, Prkce - protein kinase c, epsilon, Mt1 - metallothionein 1, Aqp4 - aquaporin 4, Hlcs - holocarboxylase synthetase (biotin- [propriony-coenzyme a-carboxylase (atp-hydrolysing)] ligase), Gch1 - gtp cyclohydrolase 1, Prpf8 - pre-mrna processing factor 8, Ucp2 - uncoupling protein 2 (mitochondrial, proton carrier), Pde3b - phosphodiesterase 3b, cgmp-inhibited, Tfr2 - transferrin receptor 2, Fancd2 - fanconi anemia, complementation group d2, Mt2 - metallothionein 2, Tbc1d7 - tbc1 domain family, member 7, Trim2 - tripartite motif-containing 2, Gata6 - gata binding protein 6, Gata5 - gata binding protein 5, Kit - kit oncogene, Mapt - microtubule-associated protein tau, Smpd3 - sphingomyelin phosphodiesterase 3, neutral, Cidea - cell death-inducing dna fragmentation factor, alpha subunit-like effector a, Ass1 - argininosuccinate synthetase 1, Pdgfrb - platelet derived growth factor receptor, beta polypeptide, Ppp2r5b - protein phosphatase 2, regulatory subunit b (b56), beta isoform, Hspa13 - heat shock protein 70 family, member 13, Gas6 - growth arrest specific 6, Mapk15 - mitogen-activated protein kinase 15, Map3k5 - mitogen-activated protein kinase kinase kinase 5, Amigo1 - adhesion molecule with ig like domain 1, Irs2 - insulin receptor substrate 2, Cth - cystathionase (cystathionine gamma-lyase), Akap8 - a kinase (prka) anchor protein 8, Fbxo6 - f-box protein 6, Slc6a4 - solute carrier family 6 (neurotransmitter transporter, serotonin), member 4, Nmral1 - nmra-like family domain containing 1, Gapdh - glyceraldehyde-3-phosphate dehydrogenase, Mras - muscle and microspikes ras, Ikbke - inhibitor of kappab kinase epsilon, Pck1 - phosphoenolpyruvate carboxykinase 1, cytosolic, Icam1 - intercellular adhesion molecule 1, Foxo3 - forkhead box o3, Col3a1 - collagen, type iii, alpha 1, Plscr2 - phospholipid scramblase 2, Vegfa - vascular endothelial growth factor a, Col4a2 - collagen, type iv, alpha 2, Trem2 - triggering receptor expressed on myeloid cells 2, Slc27a1 - solute carrier family 27 (fatty acid transporter), member 1, Pcolce - procollagen c-endopeptidase enhancer protein, Hyal1 - hyaluronoglucosaminidase 1, Atp4a - atpase, h+/k+ exchanging, gastric, alpha polypeptide, Gabrb3 - gamma-aminobutyric acid (gaba) a receptor, subunit beta 3, Gck - glucokinase, Cnp - 2',3'-cyclic nucleotide 3' phosphodiesterase, Cxcl10 - chemokine (c-x-c motif) ligand 10, Cpne2 - copine ii, Ces2c - carboxylesterase 2c, Gab1 - growth factor receptor bound protein 2-associated protein 1, Pmp22 - peripheral myelin protein 22, Ccr7 - chemokine (c-c motif) receptor 7, G6pdx - glucose-6-phosphate dehydrogenase x-linked, Aldob - aldolase b, fructose-bisphosphate, Tat - tyrosine aminotransferase, Pklr - pyruvate kinase liver and red blood cell, Abcc2 - atp-binding cassette, sub-family c (cftr/mrp), member 2, Fzd5 - frizzled homolog 5 (drosophila), Ifit2 - interferon-induced protein with tetratricopeptide repeats 2, Srd5a2 - steroid 5 alpha-reductase 2, Dtx1 - deltex 1 homolog (drosophila), Serpine1 - serine (or cysteine) peptidase inhibitor, clade e, member 1, Socs2 - suppressor of cytokine signaling 2, Cib2 - calcium and integrin binding family member 2, Plcd1 - phospholipase c, delta 1, Plekha1 - pleckstrin homology domain containing, family a (phosphoinositide binding specific) member 1, Plscr1 - phospholipid scramblase 1, Med1 - mediator complex subunit 1, Crip1 - cysteine-rich protein 1 (intestinal), Gpam - glycerol-3-phosphate acyltransferase, mitochondrial, Asxl1 - additional sex combs like 1, Sp5 - trans-acting transcription factor 5, Eno1 - enolase 1, alpha non-neuron, Irgm2 - immunity-related gtpase family m

			member 2, Gpi1 - glucose phosphate isomerase 1, Bcat2 - branched chain aminotransferase 2, mitochondrial, Serpina1e - serine (or cysteine) peptidase inhibitor, clade a, member 1e, Mmp2 - matrix metallopeptidase 2, Jup - junction plakoglobin, Jun - jun oncogene, Got1 - glutamate oxaloacetate transaminase 1, soluble, Mmp13 - matrix metallopeptidase 13, Ppp1r1b - protein phosphatase 1, regulatory (inhibitor) subunit 1b, Bax - bcl2-associated x protein, Adcy3 - adenylate cyclase 3, Trim13 - tripartite motif-containing 13, Adnp2 - adnp homeobox 2, Lrat - lecithin-retinol acyltransferase (phosphatidylcholine-retinol-o-acyltransferase), Acacb - acetyl-coenzyme a carboxylase beta, Glrx - glutaredoxin, F830016B08Rik - riken cdna f830016b08 gene, Me1 - malic enzyme 1, nadp(+)-dependent, cytosolic, Gm4951 - predicted gene 4951, Gngt1 - guanine nucleotide binding protein (g protein), gamma transducing activity polypeptide 1, Herpud1 - homocysteine-inducible, endoplasmic reticulum stress-inducible, ubiquitin-like domain member 1, Twf2 - twinfilin, actin-binding protein, homolog 2 (drosophila), Itgb1bp1 - integrin beta 1 binding protein 1, Sox6 - sry-box containing gene 6, Por - p450 (cytochrome) oxidoreductase, Tank - traf family member-associated nf-kappa b activator, Peli1 - pellino 1, Relb - avian reticuloendotheliosis viral (v-rel) oncogene related b, Col1a2 - collagen, type i, alpha 2, Col1a1 - collagen, type i, alpha 1, Arid5b - at rich interactive domain 5b (mrf1-like), Itpr2 - inositol 1,4,5-triphosphate receptor 2, Epha4 - eph receptor a4, Epha3 - eph receptor a3, A230050P20Rik - riken cdna a230050p20 gene, Col6a1 - collagen, type vi, alpha 1, Upf2 - upf2 regulator of nonsense transcripts homolog (yeast), Rhoq - ras homolog gene family, member q, Rpl36al - ribosomal protein l36a-like, Rfx2 - regulatory factor x, 2 (influences hla class ii expression), Ednrb - endothelin receptor type b, Glul - glutamate-ammonia ligase (glutamine synthetase), Brca1 - breast cancer 1, Tuba1b - tubulin, alpha 1b, Spry2 - sprouty homolog 2 (drosophila), Khk - ketohexokinase, Bst2 - bone marrow stromal cell antigen 2, Spry4 - sprouty homolog 4 (drosophila), Ogt - o-linked n-acetylglucosamine (glcnac) transferase (udp-n-acetylglucosamine:polypeptide-n-acetylglucosaminyl transferase), Sirt1 - sirtuin 1, Adamts7 - a disintegrin-like and metallopeptidase (reprolysin type) with thrombospondin type 1 motif, 7, Trpv4 - transient receptor potential cation channel, subfamily v, member 4, Dusp10 - dual specificity phosphatase 10, Abat - 4-aminobutyrate aminotransferase, Bmp7 - bone morphogenetic protein 7, Mecp2 - methyl cpg binding protein 2, Bmp6 - bone morphogenetic protein 6, Bmp4 - bone morphogenetic protein 4, Sdf2l1 - stromal cell-derived factor 2-like 1, Slc22a7 - solute carrier family 22 (organic anion transporter), member 7, Ocln - occludin, Ehd1 - eh-domain containing 1, Slc10a3 - solute carrier family 10 (sodium/bile acid cotransporter family), member 3, Hp - haptoglobin, Mga - max gene associated, Stap1 - signal transducing adaptor family member 1, Nr4a2 - nuclear receptor subfamily 4, group a, member 2, Btk - bruton agammaglobulinemia tyrosine kinase, Hprt - hypoxanthine guanine phosphoribosyl transferase, Nol3 - nucleolar protein 3 (apoptosis repressor with card domain), Tlr2 - toll-like receptor 2, Egr1 - early growth response 1, Ubd - ubiquitin d, Rapgef2 - rap guanine nucleotide exchange factor (gef) 2, Egr2 - early growth response 2]
GO: 0017144	drug metabolic process	8.63E-03	[Cyp46a1 - cytochrome p450, family 46, subfamily a, polypeptide 1, Cyp2d12 - cytochrome p450, family 2, subfamily d, polypeptide 12, Khk - ketohexokinase, Cbs - cystathionine beta-synthase, Agtr1a - angiotensin ii receptor, type 1a, Cps1 - carbamoyl-phosphate synthetase 1, Gpi1 - glucose phosphate isomerase 1, Agxt - alanine-glyoxylate aminotransferase, Cyp2g1 - cytochrome p450, family 2, subfamily g, polypeptide 1, Gnmt - glycine n-methyltransferase, Ahcy - s-adenosylhomocysteine hydrolase, Cyp2c55 - cytochrome p450, family 2, subfamily c, polypeptide 55, Gck - glucokinase, Aass - aminoadipate-semialdehyde synthase, Ace - angiotensin i converting enzyme (peptidyl-dipeptidase a) 1, Gm3839 - glyceraldehyde-3-phosphate dehydrogenase pseudogene, Urad - ureidoimidazoline (2-oxo-4-hydroxy-4-carboxy-5) decarboxylase, Gale - galactose-4-epimerase, udp, Acly - atp citrate lyase, Gch1 - gtp cyclohydrolase 1, Gpx3 - glutathione peroxidase 3, Cs - citrate synthase, Tat - tyrosine aminotransferase, Pklr - pyruvate kinase liver and red blood cell, Slc7a2 - solute carrier family 7 (cationic amino acid transporter, y+ system), member 2, Cyp1a1 - cytochrome p450, family 1, subfamily a, polypeptide 1, Hpd - 4-hydroxyphenylpyruvic acid dioxygenase, Pycr1 - pyrroline-5-carboxylate reductase 1, Cyp2c54 - cytochrome p450, family 2, subfamily c, polypeptide 54, Nr4a2 - nuclear receptor subfamily 4, group a, member 2, Acpp - acid phosphatase, prostate, Cycs - cytochrome c, somatic, Por - p450 (cytochrome) oxidoreductase, Cyp2c29 - cytochrome p450, family

			2, subfamily c, polypeptide 29, Kdm3a - lysine (k)-specific demethylase 3a, Apoa4 - apolipoprotein a-iv, Cth - cystathionase (cystathionine gamma-lyase), Acmsd - amino carboxymuconate semialdehyde decarboxylase, Tlr2 - toll-like receptor 2, Cyp2b13 - cytochrome p450, family 2, subfamily b, polypeptide 13, Cyp2b10 - cytochrome p450, family 2, subfamily b, polypeptide 10, Dhtkd1 - dehydrogenase e1 and transketolase domain containing 1]
GO: 0050896	response to stimulus	8.51E-03	[H2-T24 - histocompatibility 2, t region locus 24, Mr1 - major histocompatibility complex, class i-related, Ptgis - prostaglandin i2 (prostacyclin) synthase, Gsn - gelsolin, Prdm2 - pr domain containing 2, with znf domain, Cblb - casitas b-lineage lymphoma b, H2-Q7 - histocompatibility 2, q region locus 7, Abl2 - v-abl abelson murine leukemia viral oncogene 2 (arg, abelson-related gene), Cyp3a11 - cytochrome p450, family 3, subfamily a, polypeptide 11, Tmem38a - transmembrane protein 38a, Hmgcs1 - 3-hydroxy-3-methylglutaryl-coenzyme a synthase 1, Ahcy - s-adenosylhomocysteine hydrolase, Adam23 - a disintegrin and metallopeptidase domain 23, Ptafr - platelet-activating factor receptor, Stx4a - syntaxin 4a (placental), H2-Q1 - histocompatibility 2, q region locus 1, Ace - angiotensin i converting enzyme (peptidyl-dipeptidase a) 1, Ptch1 - patched homolog 1, Pcsk9 - proprotein convertase subtilisin/kexin type 9, Snx10 - sorting nexin 10, Derl3 - der1-like domain family, member 3, Cxcl14 - chemokine (c-x-c motif) ligand 14, Esco2 - establishment of cohesion 1 homolog 2 (s. cerevisiae), Cyp7a1 - cytochrome p450, family 7, subfamily a, polypeptide 1, Chrna4 - cholinergic receptor, nicotinic, alpha polypeptide 4, Rnf186 - ring finger protein 186, Tnks1bp1 - tankyrase 1 binding protein 1, Zfp36 - zinc finger protein 36, P2rx3 - purinergic receptor p2x, ligand-gated ion channel, 3, Mcm3 - minichromosome maintenance deficient 3 (s. cerevisiae), Hba-a1 - hemoglobin alpha, adult chain 1, Scamp5 - secretory carrier membrane protein 5, Stk10 - serine/threonine kinase 10, Actb - actin, beta, Matn2 - matrilin 2, Kdm3a - lysine (k)-specific demethylase 3a, Serp1 - stress-associated endoplasmic reticulum protein 1, Ada - adenosine deaminase, Stat5a - signal transducer and activator of transcription 5a, Acvr2b - activin receptor iib, Sulf2 - sulfatase 2, Cotl1 - coactosin-like 1 (dictyostelium), Ly6d - lymphocyte antigen 6 complex, locus d, Cps1 - carbamoyl-phosphate synthetase 1, Adam8 - a disintegrin and metallopeptidase domain 8, Lrp11 - low density lipoprotein receptor-related protein 11, Uba5 - ubiquitin-like modifier activating enzyme 5, Crebrf - creb3 regulatory factor, Ltbr - lymphotoxin b receptor, Dpyd - dihydropyrimidine dehydrogenase, Pvt1 - plasmacytoma variant translocation 1, Scarb1 - scavenger receptor class b, member 1, Isg20 - interferon-stimulated protein, Src - rous sarcoma oncogene, Cfd - complement factor d (adipsin), Zfp651 - zinc finger protein 651, Gpx6 - glutathione peroxidase 6, Cyp1a1 - cytochrome p450, family 1, subfamily a, polypeptide 1, Pycr1 - pyrroline-5-carboxylate reductase 1, Adrb3 - adrenergic receptor, beta 3, Smad3 - smad family member 3, Smad7 - smad family member 7, Dclre1c - dna cross-link repair 1c, pso2 homolog (s. cerevisiae), Ly96 - lymphocyte antigen 96, Cyp2b9 - cytochrome p450, family 2, subfamily b, polypeptide 9, Ptp4a3 - protein tyrosine phosphatase 4a3, Cd52 - cd52 antigen, P2ry4 - pyrimidinergic receptor p2y, g-protein coupled, 4, Ing4 - inhibitor of growth family, member 4, Cyp2b10 - cytochrome p450, family 2, subfamily b, polypeptide 10, F13a1 - coagulation factor xiii, a1 subunit, Cyp2a5 - cytochrome p450, family 2, subfamily a, polypeptide 5, Lmnb1 - lamin b1, Zmat3 - zinc finger matrin type 3, Hamp - hepcidin antimicrobial peptide, Agtr1a - angiotensin ii receptor, type 1a, Derl2 - der1-like domain family, member 2, Swsap1 - swim type zinc finger 7 associated protein 1, Agxt - alanine-glyoxylate aminotransferase, Slc25a27 - solute carrier family 25, member 27, Lifr - leukemia inhibitory factor receptor, Wnt4 - wingless-related mmtv integration site 4, Rftn2 - raftlin family member 2, Wnt5a - wingless-related mmtv integration site 5a, Rangap1 - ran gtpase activating protein 1, Rasgrp2 - ras, guanyl releasing protein 2, D130043K22Rik - riken cdna d130043k22 gene, Als2 - amyotrophic lateral sclerosis 2 (juvenile), Neat1 - nuclear paraspeckle assembly transcript 1 (non-protein coding), Sucnr1 - succinate receptor 1, Mms19 - mms19 (met18 s. cerevisiae), Gpx3 - glutathione peroxidase 3, Rab34 - rab34, member of ras oncogene family, Trim59 - tripartite motif-containing 59, Hspa5 - heat shock protein 5, Vim - vimentin, Vnn1 - vanin 1, Kdm5b - lysine (k)-specific demethylase 5b, Lsp1 - lymphocyte specific 1, Vldlr - very low density lipoprotein receptor, Tas1r3 - taste receptor, type 1, member 3, Rad51b - rad51 homolog b, Tnip1 - tnfaip3 interacting protein 1, Rd3 - retinal degeneration 3, Lrp6 - low density lipoprotein receptor-related protein 6, Gsta2 - glutathione s-transferase, alpha 2 (yc2), Sbspon - somatomedin b and thrombospondin, type 1 domain

			containing, Lrp1 - low density lipoprotein receptor-related protein 1, Ifi205 - interferon activated gene 205, Anxa1 - annexin a1, Polr3k - polymerase (rna) iii (dna directed) polypeptide k, Ang - angiogenin, ribonuclease, rnase a family, 5, Pla2g4f - phospholipase a2, group ivf, Enpp3 - ectonucleotide pyrophosphatase/phosphodiesterase 3, Usp28 - ubiquitin specific peptidase 28, Slc30a10 - solute carrier family 30, member 10, Grm8 - glutamate receptor, metabotropic 8, Steap2 - six transmembrane epithelial antigen of prostate 2, Psmb9 - proteasome (prosome, macropain) subunit, beta type 9 (large multifunctional peptidase 2), Stk39 - serine/threonine kinase 39, Akr1c18 - aldo-keto reductase family 1, member c18, Defb1 - defensin beta 1, Anxa5 - annexin a5, Slc25a5 - solute carrier family 25 (mitochondrial carrier, adenine nucleotide translocator), member 5, Smc5 - structural maintenance of chromosomes 5, Tril - tlr4 interactor with leucine-rich repeats, Gucy2c - guanylate cyclase 2c, Guca1a - guanylate cyclase activator 1a (retina), Parp3 - poly (adp-ribose) polymerase family, member 3, Cpne8 - copine viii, Fabp4 - fatty acid binding protein 4, adipocyte, Pcgf2 - polycomb group ring finger 2, Plscr4 - phospholipid scramblase 4, Pmaip1 - phorbol-12-myristate-13-acetate-induced protein 1, Dnajc10 - dnaj (hsp40) homolog, subfamily c, member 10, Rnase4 - ribonuclease, rnase a family 4, C8b - complement component 8, beta polypeptide, Gstt2 - glutathione s-transferase, theta 2, Fuca2 - fucosidase, alpha-l- 2, plasma, Mad2l2 - mad2 mitotic arrest deficient-like 2, Zfp259 - zinc finger protein 259, Mcm10 - minichromosome maintenance deficient 10 (s. cerevisiae), Syt1 - synaptotagmin i, H2-D1 - histocompatibility 2, d region locus 1, Lgals3 - lectin, galactose binding, soluble 3, Apbb1 - amyloid beta (a4) precursor protein-binding, family b, member 1, Mir22hg - mir22 host gene (non-protein coding), Nhlrc1 - nhl repeat containing 1, Atp1a2 - atpase, na+/k+ transporting, alpha 2 polypeptide, Epb4.1l5 - erythrocyte protein band 4.1-like 5, Gzma - granzyme a, Apoa4 - apolipoprotein a-iv, Dnaja4 - dnaj (hsp40) homolog, subfamily a, member 4, Lcat - lecithin cholesterol acyltransferase, Sdc1 - syndecan 1, Themis - thymocyte selection associated, Baiap2l1 - bai1-associated protein 2-like 1, Lcn2 - lipocalin 2, Slc2a1 - solute carrier family 2 (facilitated glucose transporter), member 1, Srp19 - signal recognition particle 19, Ar - androgen receptor, Slc23a1 - solute carrier family 23 (nucleobase transporters), member 1, Hsd3b2 - hydroxy-delta-5-steroid dehydrogenase, 3 beta- and steroid delta-isomerase 2, Tnrc6a - trinucleotide repeat containing 6a, Unc13b - unc-13 homolog b (c. elegans), Unc119 - unc-119 homolog (c. elegans), Ucp3 - uncoupling protein 3 (mitochondrial, proton carrier), Hspb1 - heat shock protein 1, Prkca - protein kinase c, alpha, Macrod2 - macro domain containing 2, Prkce - protein kinase c, epsilon, Sh3bp4 - sh3-domain binding protein 4, Aqp4 - aquaporin 4, Mt1 - metallothionein 1, Phlpp1 - ph domain and leucine rich repeat protein phosphatase 1, Hlcs - holocarboxylase synthetase (biotin- [propriony-coenzyme a-carboxylase (atp-hydrolysing)] ligase), Ucp2 - uncoupling protein 2 (mitochondrial, proton carrier), Fancd2 - fanconi anemia, complementation group d2, Mt2 - metallothionein 2, Sprtn - sprt-like n-terminal domain, Trim2 - tripartite motif-containing 2, Nudt1 - nudix (nucleoside diphosphate linked moiety x)-type motif 1, Hamp2 - hepcidin antimicrobial peptide 2, Smpd3 - sphingomyelin phosphodiesterase 3, neutral, Tifa - traf-interacting protein with forkhead-associated domain, Mapt - microtubule-associated protein tau, Pigr - polymeric immunoglobulin receptor, Ass1 - argininosuccinate synthetase 1, Ppp2r5b - protein phosphatase 2, regulatory subunit b (b56), beta isoform, Hspa13 - heat shock protein 70 family, member 13, Pla2g16 - phospholipase a2, group xvi, Mapk15 - mitogen-activated protein kinase 15, Irs2 - insulin receptor substrate 2, Cth - cystathionase (cystathionine gamma-lyase), Cds2 - cdp-diacylglycerol synthase (phosphatidate cytidylyltransferase) 2, Ivns1abp - influenza virus ns1a binding protein, Slc10a2 - solute carrier family 10, member 2, Cxcr6 - chemokine (c-x-c motif) receptor 6, Slc6a4 - solute carrier family 6 (neurotransmitter transporter, serotonin), member 4, Nmral1 - nmra-like family domain containing 1, Gtf2h5 - general transcription factor iih, polypeptide 5, Atp2b2 - atpase, ca++ transporting, plasma membrane 2, Serpinf2 - serine (or cysteine) peptidase inhibitor, clade f, member 2, Synpo - synaptopodin, Mras - muscle and microspikes ras, Icam1 - intercellular adhesion molecule 1, Vegfb - vascular endothelial growth factor b, Plscr2 - phospholipid scramblase 2, Vegfa - vascular endothelial growth factor a, Mus81 - mus81 endonuclease homolog (yeast), Trem2 - triggering receptor expressed on myeloid cells 2, Atp4a - atpase, h+/k+ exchanging, gastric, alpha polypeptide, Hyal1 - hyaluronoglucosaminidase 1, Gck - glucokinase, Atf3 - activating transcription factor 3, Zswim7 - zinc finger swim-type containing 7,

			Clic5 - chloride intracellular channel 5, Cxcl10 - chemokine (c-x-c motif) ligand 10, Cpne2 - copine ii, Fndc5 - fibronectin type iii domain containing 5, Atox1 - atx1 (antioxidant protein 1) homolog 1 (yeast), Insig2 - insulin induced gene 2, Ces2c - carboxylesterase 2c, Pmp22 - peripheral myelin protein 22, Tat - tyrosine aminotransferase, Pklr - pyruvate kinase liver and red blood cell, Ccdc80 - coiled-coil domain containing 80, Ifit2 - interferon-induced protein with tetratricopeptide repeats 2, Msh5 - muts homolog 5 (e. coli), Vac14 - vac14 homolog (s. cerevisiae), Pnpla3 - patatin-like phospholipase domain containing 3, Serpine1 - serine (or cysteine) peptidase inhibitor, clade e, member 1, Atmin - atm interactor, Nek6 - nima (never in mitosis gene a)-related expressed kinase 6, Plat - plasminogen activator, tissue, Atpif1 - atpase inhibitory factor 1, Ifngr2 - interferon gamma receptor 2, Plcd1 - phospholipase c, delta 1, Siglecg - sialic acid binding ig-like lectin g, Plscr1 - phospholipid scramblase 1, Med1 - mediator complex subunit 1, Cebpe - ccaat/enhancer binding protein (c/ebp), epsilon, Emp2 - epithelial membrane protein 2, Bhlha15 - basic helix-loop-helix family, member a15, Fam19a2 - family with sequence similarity 19, member a2, Eno1 - enolase 1, alpha non-neuron, Irgm2 - immunity-related gtpase family m member 2, Marf1 - meiosis arrest female 1, Bcat2 - branched chain aminotransferase 2, mitochondrial, Serpina1e - serine (or cysteine) peptidase inhibitor, clade a, member 1e, Mmp2 - matrix metallopeptidase 2, Arhgef19 - rho guanine nucleotide exchange factor (gef) 19, Mmp13 - matrix metallopeptidase 13, Ppp1r1b - protein phosphatase 1, regulatory (inhibitor) subunit 1b, Eme1 - essential meiotic endonuclease 1 homolog 1 (s. pombe), Bax - bcl2-associated x protein, Adcy3 - adenylate cyclase 3, Mmp12 - matrix metallopeptidase 12, Trim13 - tripartite motif-containing 13, Bach1 - btb and cnc homology 1, Lrat - lecithin-retinol acyltransferase (phosphatidylcholine-retinol-o-acyltransferase), Ppl - periplakin, Gdap10 - ganglioside-induced differentiation-associated-protein 10, Taok3 - tao kinase 3, Me1 - malic enzyme 1, nadp(+)-dependent, cytosolic, Hrk - harakiri, bcl2 interacting protein (contains only bh3 domain), Bcl2l11 - bcl2-like 11 (apoptosis facilitator), Pola1 - polymerase (dna directed), alpha 1, Fabp7 - fatty acid binding protein 7, brain, Polb - polymerase (dna directed), beta, Wrnip1 - werner helicase interacting protein 1, Chd6 - chromodomain helicase dna binding protein 6, Tpm1 - tropomyosin 1, alpha, Sox6 - sry-box containing gene 6, Por - p450 (cytochrome) oxidoreductase, Arid5b - at rich interactive domain 5b (mrf1-like), Pole - polymerase (dna directed), epsilon, Foxa3 - forkhead box a3, Epha4 - eph receptor a4, Epha3 - eph receptor a3, Pspc1 - paraspeckle protein 1, Rhoq - ras homolog gene family, member q, Ednrb - endothelin receptor type b, Rpl36al - ribosomal protein l36a-like, Prlr - prolactin receptor, Brca1 - breast cancer 1, Tuba1b - tubulin, alpha 1b, Bst2 - bone marrow stromal cell antigen 2, Ogt - o-linked n-acetylglucosamine (glcnac) transferase (udp-n-acetylglucosamine:polypeptide-n-acetylglucosaminyl transferase), Adamts7 - a disintegrin-like and metallopeptidase (reprolysin type) with thrombospondin type 1 motif, 7, Cyld - cylindromatosis (turban tumor syndrome), Abat - 4-aminobutyrate aminotransferase, N4bp1 - nedd4 binding protein 1, Bmp7 - bone morphogenetic protein 7, Bmp6 - bone morphogenetic protein 6, Mecp2 - methyl cpg binding protein 2, Aen - apoptosis enhancing nuclease, Tonsl - tonsoku-like, dna repair protein, Bmp4 - bone morphogenetic protein 4, S1pr3 - sphingosine-1-phosphate receptor 3, Slc22a7 - solute carrier family 22 (organic anion transporter), member 7, Eda - ectodysplasin-a, C3 - complement component 3, Ehd1 - eh-domain containing 1, Hp - haptoglobin, Crebzf - creb/atf bzip transcription factor, Prkg2 - protein kinase, cgmp-dependent, type ii, Btk - bruton agammaglobulinemia tyrosine kinase, Smpd2 - sphingomyelin phosphodiesterase 2, neutral, Hprt - hypoxanthine guanine phosphoribosyl transferase, Kmt2a - lysine (k)-specific methyltransferase 2a, Egr1 - early growth response 1, Ago3 - argonaute risc catalytic subunit 3, Rapgef2 - rap guanine nucleotide exchange factor (gef) 2, Egr2 - early growth response 2, Fasn - fatty acid synthase, Lig4 - ligase iv, dna, atp-dependent, Dll4 - delta-like 4 (drosophila), Ntrk1 - neurotrophic tyrosine kinase, receptor, type 1, Capg - capping protein (actin filament), gelsolin-like, Ntrk2 - neurotrophic tyrosine kinase, receptor, type 2, Nfat5 - nuclear factor of activated t cells 5, Pde3a - phosphodiesterase 3a, cgmp inhibited, Vtcn1 - v-set domain containing t cell activation inhibitor 1, Ankzf1 - ankyrin repeat and zinc finger domain containing 1, Gpx4 - glutathione peroxidase 4, Car3 - carbonic anhydrase 3, Bbs1 - bardet-biedl syndrome 1 (human), Chchd6 - coiled-coil-helix-coiled-coil-helix domain containing 6, Abhd2 - abhydrolase domain containing 2, Lamtor4 - late endosomal/lysosomal adaptor, mapk and mtor activator 4, Hyou1 - hypoxia up-regulated 1, Abcb11 - atp-

			binding cassette, sub-family b (mdr/tap), member 11, Nptx1 - neuronal pentraxin 1, Tert - telomerase reverse transcriptase, F2r - coagulation factor ii (thrombin) receptor, Scara5 - scavenger receptor class a, member 5 (putative), Pdgfc - platelet-derived growth factor, c polypeptide, Zfp516 - zinc finger protein 516, Dntt - deoxynucleotidyltransferase, terminal, Cacnb4 - calcium channel, voltage-dependent, beta 4 subunit, Notch2 - notch 2, Rpgr - retinitis pigmentosa gtpase regulator, Rcan1 - regulator of calcineurin 1, Fabp1 - fatty acid binding protein 1, liver, Tdg - thymine dna glycosylase, Cacna1f - calcium channel, voltage-dependent, alpha 1f subunit, Npc1 - niemann pick type c1, Ube2e2 - ubiquitin-conjugating enzyme e2e 2, Anxa2 - annexin a2, Runx3 - runt related transcription factor 3, Cbs - cystathionine beta-synthase, Nr4a3 - nuclear receptor subfamily 4, group a, member 3, Evpl - envoplakin, Rufy4 - run and fyve domain containing 4, Ercc3 - excision repair cross-complementing rodent repair deficiency, complementation group 3, Mfap4 - microfibrillar-associated protein 4, Casp4 - caspase 4, apoptosis-related cysteine peptidase, S100a10 - s100 calcium binding protein a10 (calpactin), Casp12 - caspase 12, Smad9 - smad family member 9, Manf - mesencephalic astrocyte-derived neurotrophic factor, Insr - insulin receptor, S100a8 - s100 calcium binding protein a8 (calgranulin a), Esr1 - estrogen receptor 1 (alpha), Nfkb2 - nuclear factor of kappa light polypeptide gene enhancer in b cells 2, p49/p100, Rragd - ras-related gtp binding d, Acaca - acetyl-coenzyme a carboxylase alpha, Lpin3 - lipin 3, Eepd1 - endonuclease/exonuclease/phosphatase family domain containing 1, Slc38a3 - solute carrier family 38, member 3, 2310040G24Rik - riken cdna 2310040g24 gene, Scd1 - stearoyl-coenzyme a desaturase 1, Loxl2 - lysyl oxidase-like 2, Agbl5 - atp/gtp binding protein-like 5, Ifi27l2b - interferon, alpha-inducible protein 27 like 2b, Ighg2c - immunoglobulin heavy constant gamma 2c, Entpd2 - ectonucleoside triphosphate diphosphohydrolase 2, Ramp1 - receptor (calcitonin) activity modifying protein 1, Rnase6 - ribonuclease, rnase a family, 6, Bok - bcl2-related ovarian killer protein, Ip6k2 - inositol hexaphosphate kinase 2, Timp1 - tissue inhibitor of metalloproteinase 1, Cd83 - cd83 antigen, Tnfrsf14 - tumor necrosis factor receptor superfamily, member 14 (herpesvirus entry mediator), Timp2 - tissue inhibitor of metalloproteinase 2, Smo - smoothened homolog (drosophila), Apcs - serum amyloid p-component, Cd81 - cd81 antigen, Fstl1 - follistatin-like 1, Ddit4 - dna-damage-inducible transcript 4, Saa2 - serum amyloid a 2, Saa3 - serum amyloid a 3, Saa1 - serum amyloid a 1, Cd63 - cd63 antigen, Fbxo44 - f-box protein 44, Ccl2 - chemokine (c-c motif) ligand 2, Acot11 - acyl-coa thioesterase 11, Hsbp1l1 - heat shock factor binding protein 1-like 1, Pcolce2 - procollagen c-endopeptidase enhancer 2, Tnfrsf1a - tumor necrosis factor receptor superfamily, member 1a, Mapkapk3 - mitogen-activated protein kinase-activated protein kinase 3, Tnfsf12 - tumor necrosis factor (ligand) superfamily, member 12, Ece1 - endothelin converting enzyme 1, Scnn1a - sodium channel, nonvoltage-gated 1 alpha, Cd36 - cd36 antigen, Serpinb1a - serine (or cysteine) peptidase inhibitor, clade b, member 1a, Ighg2b - immunoglobulin heavy constant gamma 2b, Ighm - immunoglobulin heavy constant mu, Tnc - tenascin c, Cpeb4 - cytoplasmic polyadenylation element binding protein 4, Cd1d2 - cd1d2 antigen, Rif1 - rap1 interacting factor 1 homolog (yeast), Asns - asparagine synthetase, Sec61b - sec61 beta subunit, Cd19 - cd19 antigen, Vmn1r53 - vomeronasal 1 receptor 53, Oasl1 - 2'-5' oligoadenylate synthetase-like 1, C4a - complement component 4a (rodgers blood group), Srxn1 - sulfiredoxin 1 homolog (s. cerevisiae), Cxcl2 - chemokine (c-x-c motif) ligand 2, Figf - c-fos induced growth factor, Cd300lb - cd300 antigen like family member b, Ccl9 - chemokine (c-c motif) ligand 9, Dsp - desmoplakin, Chek2 - checkpoint kinase 2, Cxcl12 - chemokine (c-x-c motif) ligand 12, Mustn1 - musculoskeletal, embryonic nuclear protein 1, Higd1a - hig1 domain family, member 1a, Chaf1a - chromatin assembly factor 1, subunit a (p150), Plac8 - placenta-specific 8, Ccl6 - chemokine (c-c motif) ligand 6, Il6ra - interleukin 6 receptor, alpha, Thrsp - thyroid hormone responsive, Nupr1 - nuclear protein transcription regulator 1, Cdh1 - cadherin 1, Taok2 - tao kinase 2, Bmf - bcl2 modifying factor, Ocstamp - osteoclast stimulatory transmembrane protein, Pdk4 - pyruvate dehydrogenase kinase, isoenzyme 4, Phlda3 - pleckstrin homology-like domain, family a, member 3, Cdkn1a - cyclin-dependent kinase inhibitor 1a (p21), Sema5b - sema domain, seven thrombospondin repeats (type 1 and type 1-like), transmembrane domain (tm) and short cytoplasmic domain, (semaphorin) 5b, Thbs1 - thrombospondin 1, Mup3 - major urinary protein 3, Tipin - timeless interacting protein, Uchl3 - ubiquitin carboxyl-terminal esterase l3 (ubiquitin thiolesterase), Fcor - foxo1 corepressor, Cyp3a25 - cytochrome p450, family 3, subfamily a, polypeptide 25, Adamts12 - a

			disintegrin-like and metallopeptidase (reprolysin type) with thrombospondin type 1 motif, 12, Nlrp6 - nlr family, pyrin domain containing 6, Krt10 - keratin 10, Mapk8ip3 - mitogen-activated protein kinase 8 interacting protein 3, Gch1 - gtp cyclohydrolase 1, Prpf8 - pre-mrna processing factor 8, Tfr2 - transferrin receptor 2, Pde3b - phosphodiesterase 3b, cgmp-inhibited, Rspo1 - r-spondin homolog (xenopus laevis), Tbc1d7 - tbc1 domain family, member 7, Gata6 - gata binding protein 6, Gata5 - gata binding protein 5, Kit - kit oncogene, Pdgfa - platelet derived growth factor, alpha, Ddx17 - dead (asp-glu-ala-asp) box polypeptide 17, Cidea - cell death-inducing dna fragmentation factor, alpha subunit-like effector a, Pdgfrb - platelet derived growth factor receptor, beta polypeptide, Gas6 - growth arrest specific 6, Map3k8 - mitogen-activated protein kinase kinase kinase 8, Map3k5 - mitogen-activated protein kinase kinase kinase 5, 5330417C22Rik - riken cdna 5330417c22 gene, Amigo1 - adhesion molecule with ig like domain 1, Enpp2 - ectonucleotide pyrophosphatase/phosphodiesterase 2, Hba-a2 - hemoglobin alpha, adult chain 2, Akap8 - a kinase (prka) anchor protein 8, Fbxo6 - f-box protein 6, Gapdh - glyceraldehyde-3-phosphate dehydrogenase, Igha - immunoglobulin heavy constant alpha, Ikbke - inhibitor of kappab kinase epsilon, Pck1 - phosphoenolpyruvate carboxykinase 1, cytosolic, Foxo3 - forkhead box o3, Col3a1 - collagen, type iii, alpha 1, Poli - polymerase (dna directed), iota, Col4a2 - collagen, type iv, alpha 2, Arhgdia - rho gdp dissociation inhibitor (gdi) alpha, Slc27a1 - solute carrier family 27 (fatty acid transporter), member 1, Pcolce - procollagen c-endopeptidase enhancer protein, Szt2 - seizure threshold 2, Gabrb3 - gamma-aminobutyric acid (gaba) a receptor, subunit beta 3, Cnp - 2',3'-cyclic nucleotide 3' phosphodiesterase, Clec2h - c-type lectin domain family 2, member h, Foxp2 - forkhead box p2, Gab1 - growth factor receptor bound protein 2-associated protein 1, Ccr7 - chemokine (c-c motif) receptor 7, G6pdx - glucose-6-phosphate dehydrogenase x-linked, Clec4a2 - c-type lectin domain family 4, member a2, Aldob - aldolase b, fructose-bisphosphate, Ccr2 - chemokine (c-c motif) receptor 2, Abcc2 - atp-binding cassette, sub-family c (cftr/mrp), member 2, Abcd2 - atp-binding cassette, sub-family d (ald), member 2, Fzd5 - frizzled homolog 5 (drosophila), Srd5a2 - steroid 5 alpha-reductase 2, Dtx1 - deltex 1 homolog (drosophila), Socs2 - suppressor of cytokine signaling 2, Brip1 - brca1 interacting protein c-terminal helicase 1, Cnnm4 - cyclin m4, Cib2 - calcium and integrin binding family member 2, Wipi1 - wd repeat domain, phosphoinositide interacting 1, Zbtb18 - zinc finger and btb domain containing 18, Plekha1 - pleckstrin homology domain containing, family a (phosphoinositide binding specific) member 1, Nabp1 - nucleic acid binding protein 1, Gpam - glycerol-3-phosphate acyltransferase, mitochondrial, Crip1 - cysteine-rich protein 1 (intestinal), Asxl1 - additional sex combs like 1, Orm2 - orosomucoid 2, Sp5 - trans-acting transcription factor 5, Ccm2l - cerebral cavernous malformation 2-like, Gpi1 - glucose phosphate isomerase 1, Jup - junction plakoglobin, Got1 - glutamate oxaloacetate transaminase 1, soluble, Jun - jun oncogene, Raet1e - retinoic acid early transcript 1e, Adnp2 - adnp homeobox 2, Glrx - glutaredoxin, Acacb - acetyl-coenzyme a carboxylase beta, Dcdc2a - doublecortin domain containing 2a, Peli3 - pellino 3, F830016B08Rik - riken cdna f830016b08 gene, Gm4951 - predicted gene 4951, Clec4n - c-type lectin domain family 4, member n, Gngt1 - guanine nucleotide binding protein (g protein), gamma transducing activity polypeptide 1, Herpud1 - homocysteine-inducible, endoplasmic reticulum stress-inducible, ubiquitin-like domain member 1, Twf2 - twinfilin, actin-binding protein, homolog 2 (drosophila), Gng7 - guanine nucleotide binding protein (g protein), gamma 7, Itgb1bp1 - integrin beta 1 binding protein 1, Tank - traf family member-associated nf-kappa b activator, Peli1 - pellino 1, Orm1 - orosomucoid 1, Oasl2 - 2'-5' oligoadenylate synthetase-like 2, Relb - avian reticuloendotheliosis viral (v-rel) oncogene related b, Pygl - liver glycogen phosphorylase, Col1a2 - collagen, type i, alpha 2, Col1a1 - collagen, type i, alpha 1, Gnat1 - guanine nucleotide binding protein, alpha transducing 1, Cabp2 - calcium binding protein 2, Itpr2 - inositol 1,4,5-triphosphate receptor 2, Ifi27l2a - interferon, alpha-inducible protein 27 like 2a, Col6a1 - collagen, type vi, alpha 1, A230050P20Rik - riken cdna a230050p20 gene, Upf2 - upf2 regulator of nonsense transcripts homolog (yeast), Rfx2 - regulatory factor x, 2 (influences hla class ii expression), Kmt2d - lysine (k)-specific methyltransferase 2d, Glul - glutamate-ammonia ligase (glutamine synthetase), Spry2 - sprouty homolog 2 (drosophila), Khk - ketohexokinase, Spry4 - sprouty homolog 4 (drosophila), Immp2l - imp2 inner mitochondrial membrane peptidase-like (s. cerevisiae), Sirt1 - sirtuin 1, Trpv4 - transient receptor potential cation channel, subfamily v, member 4, Dusp10 - dual specificity phosphatase 10, Sdf2l1 - stromal cell-

			derived factor 2-like 1, Kif22 - kinesin family member 22, Ocln - occludin, Zfp385a - zinc finger protein 385a, Slc10a3 - solute carrier family 10 (sodium/bile acid cotransporter family), member 3, Tsc22d3 - tsc22 domain family, member 3, Mga - max gene associated, Stap1 - signal transducing adaptor family member 1, Ticrr - topbp1-interacting checkpoint and replication regulator, Gja10 - gap junction protein, alpha 10, Nr4a2 - nuclear receptor subfamily 4, group a, member 2, Rec8 - rec8 homolog (yeast), Nol3 - nucleolar protein 3 (apoptosis repressor with card domain), Tlr2 - toll-like receptor 2, Ubd - ubiquitin d]
GO: 0042866	pyruvate biosynthetic process	1.28E-02	[Gck - glucokinase, Pklr - pyruvate kinase liver and red blood cell, Gm3839 - glyceraldehyde-3-phosphate dehydrogenase pseudogene, Gale - galactose-4-epimerase, udp, Khk - ketohexokinase, Sds - serine dehydratase, Gpi1 - glucose phosphate isomerase 1, Agxt - alanine-glyoxylate aminotransferase, Dhtkd1 - dehydrogenase e1 and transketolase domain containing 1]
GO: 0009605	response to external stimulus	1.25E-02	[Slc2a1 - solute carrier family 2 (facilitated glucose transporter), member 1, Fcor - foxo1 corepressor, Cyp3a25 - cytochrome p450, family 3, subfamily a, polypeptide 25, Mr1 - major histocompatibility complex, class i-related, Tnrc6a - trinucleotide repeat containing 6a, Cyp3a11 - cytochrome p450, family 3, subfamily a, polypeptide 11, Ahcy - s-adenosylhomocysteine hydrolase, Nlrp6 - nlr family, pyrin domain containing 6, Vtcn1 - v-set domain containing t cell activation inhibitor 1, Ptafr - platelet-activating factor receptor, Ace - angiotensin i converting enzyme (peptidyl-dipeptidase a) 1, Pcsk9 - proprotein convertase subtilisin/kexin type 9, Macrod2 - macro domain containing 2, Phlpp1 - ph domain and leucine rich repeat protein phosphatase 1, Gch1 - gtp cyclohydrolase 1, Hlcs - holocarboxylase synthetase (biotin- [propriony-coenzyme a-carboxylase (atp-hydrolysing)] ligase), Prpf8 - pre-mrna processing factor 8, Mt2 - metallothionein 2, F2r - coagulation factor ii (thrombin) receptor, Nudt1 - nudix (nucleoside diphosphate linked moiety x)-type motif 1, Zfp36 - zinc finger protein 36, P2rx3 - purinergic receptor p2x, ligand-gated ion channel, 3, Hamp2 - hepcidin antimicrobial peptide 2, Hba-a1 - hemoglobin alpha, adult chain 1, Ddx17 - dead (asp-glu-ala-asp) box polypeptide 17, Mapt - microtubule-associated protein tau, Gas6 - growth arrest specific 6, Notch2 - notch 2, Dntt - deoxynucleotidyltransferase, terminal, Pla2g16 - phospholipase a2, group xvi, 5330417C22Rik - riken cdna 5330417c22 gene, Map3k5 - mitogen-activated protein kinase kinase kinase 5, Amigo1 - adhesion molecule with ig like domain 1, Hba-a2 - hemoglobin alpha, adult chain 2, Cds2 - cdp-diacylglycerol synthase (phosphatidate cytidylyltransferase) 2, Akap8 - a kinase (prka) anchor protein 8, Cacna1f - calcium channel, voltage-dependent, alpha 1f subunit, Slc10a2 - solute carrier family 10, member 2, Cotl1 - coactosin-like 1 (dictyostelium), Gapdh - glyceraldehyde-3-phosphate dehydrogenase, Nmral1 - nmra-like family domain containing 1, Atp2b2 - atpase, ca++ transporting, plasma membrane 2, Igha - immunoglobulin heavy constant alpha, Ikbke - inhibitor of kappab kinase epsilon, Pck1 - phosphoenolpyruvate carboxykinase 1, cytosolic, Foxo3 - forkhead box o3, Cbs - cystathionine beta-synthase, Trem2 - triggering receptor expressed on myeloid cells 2, Arhgdia - rho gdp dissociation inhibitor (gdi) alpha, Cps1 - carbamoyl-phosphate synthetase 1, Hyal1 - hyaluronoglucosaminidase 1, Szt2 - seizure threshold 2, Lrp11 - low density lipoprotein receptor-related protein 11, Gck - glucokinase, Atf3 - activating transcription factor 3, Clic5 - chloride intracellular channel 5, Cxcl10 - chemokine (c-x-c motif) ligand 10, Dpyd - dihydropyrimidine dehydrogenase, Foxp2 - forkhead box p2, Scarb1 - scavenger receptor class b, member 1, Isg20 - interferon-stimulated protein, Cfd - complement factor d (adipsin), Pmp22 - peripheral myelin protein 22, Ccr7 - chemokine (c-c motif) receptor 7, Pklr - pyruvate kinase liver and red blood cell, Ccdc80 - coiled-coil domain containing 80, Abcd2 - atp-binding cassette, sub-family d (ald), member 2, Smad3 - smad family member 3, Fzd5 - frizzled homolog 5 (drosophila), Ifit2 - interferon-induced protein with tetratricopeptide repeats 2, Ly96 - lymphocyte antigen 96, Pnpla3 - patatin-like phospholipase domain containing 3, Serpine1 - serine (or cysteine) peptidase inhibitor, clade e, member 1, Rragd - ras-related gtp binding d, Cib2 - calcium and integrin binding family member 2, Slc38a3 - solute carrier family 38, member 3, Ifngr2 - interferon gamma receptor 2, Cyp2b10 - cytochrome p450, family 2, subfamily b, polypeptide 10, Plscr1 - phospholipid scramblase 1, Gpam - glycerol-3-phosphate acyltransferase, mitochondrial, Scd1 - stearoyl-coenzyme a desaturase 1, Agbl5 - atp/gtp binding protein-like 5, Hamp - hepcidin antimicrobial peptide, Ifi27l2b - interferon, alpha-inducible protein 27 like 2b, Cebpe - ccaat/enhancer binding protein (c/ebp), epsilon, Bhlha15 - basic helix-loop-helix family, member a15, Ighg2c - immunoglobulin heavy constant gamma 2c, Irgm2 - immunity-related gtpase family m member 2, Rnase6 - ribonuclease, rnase a family, 6, Jup - junction plakoglobin, Wnt4 - wingless-related mmtv integration site 4, Got1 - glutamate oxaloacetate transaminase 1, soluble, Wnt5a -

			wingless-related mmtv integration site 5a, Tnfrsf14 - tumor necrosis factor receptor superfamily, member 14 (herpesvirus entry mediator), D130043K22Rik - riken cdna d130043k22 gene, Mmp12 - matrix metallopeptidase 12, Fstl1 - follistatin-like 1, Ddit4 - dna-damage-inducible transcript 4, Lrat - lecithin-retinol acyltransferase (phosphatidylcholine-retinol-o-acyltransferase), Saa3 - serum amyloid a 3, Acacb - acetyl-coenzyme a carboxylase beta, Saa1 - serum amyloid a 1, Gdap10 - ganglioside-induced differentiation-associated-protein 10, Peli3 - pellino 3, Cd63 - cd63 antigen, Ccl2 - chemokine (c-c motif) ligand 2, Hspa5 - heat shock protein 5, Hrk - harakiri, bcl2 interacting protein (contains only bh3 domain), Vim - vimentin, Bcl2l11 - bcl2-like 11 (apoptosis facilitator), Clec4n - c-type lectin domain family 4, member n, Tnfrsf1a - tumor necrosis factor receptor superfamily, member 1a, Fabp7 - fatty acid binding protein 7, brain, Gngt1 - guanine nucleotide binding protein (g protein), gamma transducing activity polypeptide 1, Mapkapk3 - mitogen-activated protein kinase-activated protein kinase 3, Lrp6 - low density lipoprotein receptor-related protein 6, Gsta2 - glutathione s-transferase, alpha 2 (yc2), Ifi205 - interferon activated gene 205, Cd36 - cd36 antigen, Peli1 - pellino 1, Polr3k - polymerase (rna) iii (dna directed) polypeptide k, Ighg2b - immunoglobulin heavy constant gamma 2b, Pygl - liver glycogen phosphorylase, Ighm - immunoglobulin heavy constant mu, Col1a1 - collagen, type i, alpha 1, Gnat1 - guanine nucleotide binding protein, alpha transducing 1, Foxa3 - forkhead box a3, Grm8 - glutamate receptor, metabotropic 8, Ifi27l2a - interferon, alpha-inducible protein 27 like 2a, Psmb9 - proteasome (prosome, macropain) subunit, beta type 9 (large multifunctional peptidase 2), A230050P20Rik - riken cdna a230050p20 gene, Asns - asparagine synthetase, Defb1 - defensin beta 1, Oasl1 - 2'-5' oligoadenylate synthetase-like 1, Ednrb - endothelin receptor type b, Prlr - prolactin receptor, Glul - glutamate-ammonia ligase (glutamine synthetase), Bst2 - bone marrow stromal cell antigen 2, Ogt - o-linked n-acetylglucosamine (glcnac) transferase (udp-n-acetylglucosamine:polypeptide-n-acetylglucosaminyl transferase), Sirt1 - sirtuin 1, Guca1a - guanylate cyclase activator 1a (retina), Figf - c-fos induced growth factor, Cd300lb - cd300 antigen like family member b, Fabp4 - fatty acid binding protein 4, adipocyte, Plscr4 - phospholipid scramblase 4, Mecp2 - methyl cpg binding protein 2, Bmp6 - bone morphogenetic protein 6, Cxcl12 - chemokine (c-x-c motif) ligand 12, Rnase4 - ribonuclease, rnase a family 4, Higd1a - hig1 domain family, member 1a, Fuca2 - fucosidase, alpha-l- 2, plasma, Plac8 - placenta-specific 8, C3 - complement component 3, Thrsp - thyroid hormone responsive, Hp - haptoglobin, Bmf - bcl2 modifying factor, Pdk4 - pyruvate dehydrogenase kinase, isoenzyme 4, Atp1a2 - atpase, na+/k+ transporting, alpha 2 polypeptide, Stap1 - signal transducing adaptor family member 1, Gja10 - gap junction protein, alpha 10, Nr4a2 - nuclear receptor subfamily 4, group a, member 2, Crebzf - creb/atf bzip transcription factor, Gzma - granzyme a, Btk - bruton agammaglobulinemia tyrosine kinase, Cdkn1a - cyclin-dependent kinase inhibitor 1a (p21), Smpd2 - sphingomyelin phosphodiesterase 2, neutral, Sema5b - sema domain, seven thrombospondin repeats (type 1 and type 1-like), transmembrane domain (tm) and short cytoplasmic domain, (semaphorin) 5b, Thbs1 - thrombospondin 1, Tlr2 - toll-like receptor 2, Baiap2l1 - bai1-associated protein 2-like 1, Lcn2 - lipocalin 2, Ago3 - argonaute risc catalytic subunit 3]
GO: 0006811	ion transport	1.25E-02	[Chrna4 - cholinergic receptor, nicotinic, alpha polypeptide 4, Slco2a1 - solute carrier organic anion transporter family, member 2a1, Slc7a2 - solute carrier family 7 (cationic amino acid transporter, y+ system), member 2, Scara5 - scavenger receptor class a, member 5 (putative), Smpd3 - sphingomyelin phosphodiesterase 3, neutral, Agxt - alanine-glyoxylate aminotransferase, Slc22a26 - solute carrier family 22 (organic cation transporter), member 26, Atp6v0d2 - atpase, h+ transporting, lysosomal v0 subunit d2, Tmc4 - transmembrane channel-like gene family 4, Slc16a11 - solute carrier family 16 (monocarboxylic acid transporters), member 11, Slc6a8 - solute carrier family 6 (neurotransmitter transporter, creatine), member 8, Tmem28 - transmembrane protein 28, Slc16a13 - solute carrier family 16 (monocarboxylic acid transporters), member 13, Scnn1a - sodium channel, nonvoltage-gated 1 alpha, Grid1 - glutamate receptor, ionotropic, delta 1, Slc4a9 - solute carrier family 4, sodium bicarbonate cotransporter, member 9, Apoa4 - apolipoprotein a-iv, Grin3b - glutamate receptor, ionotropic, nmda3b, Slco1a4 - solute carrier organic anion transporter family, member 1a4, Slc16a6 - solute carrier family 16 (monocarboxylic acid transporters), member 6, Aqp8 - aquaporin 8, Lcn2 - lipocalin 2]
GO: 0055114	oxidation-reduction process	1.57E-02	[Fasn - fatty acid synthase, Sdr39u1 - short chain dehydrogenase/reductase family 39u, member 1, Cyp4a14 - cytochrome p450, family 4, subfamily a, polypeptide 14, Cyp2j9 - cytochrome p450, family 2, subfamily j, polypeptide 9, Cyp3a25 - cytochrome p450, family 3, subfamily a, polypeptide 25, Gpd2 - glycerol phosphate dehydrogenase 2, mitochondrial, Ptgis - prostaglandin i2 (prostacyclin) synthase, Cyp46a1 - cytochrome p450, family 46, subfamily a, polypeptide 1, Cyp2j6 - cytochrome p450, family 2, subfamily j, polypeptide 6,

			Cyp3a11 - cytochrome p450, family 3, subfamily a, polypeptide 11, Cyp3a13 - cytochrome p450, family 3, subfamily a, polypeptide 13, Steap1 - six transmembrane epithelial antigen of the prostate 1, Cyp2g1 - cytochrome p450, family 2, subfamily g, polypeptide 1, Hsd17b6 - hydroxysteroid (17-beta) dehydrogenase 6, Aass - aminoadipate-semialdehyde synthase, Ivd - isovaleryl coenzyme a dehydrogenase, Sardh - sarcosine dehydrogenase, Gbe1 - glucan (1,4-alpha-), branching enzyme 1, Rsbn1 - rosbin, round spermatid basic protein 1, Mthfd2 - methylenetetrahydrofolate dehydrogenase (nad+ dependent), methenyltetrahydrofolate cyclohydrolase, Pyroxd2 - pyridine nucleotide-disulphide oxidoreductase domain 2, Gpd1l - glycerol-3-phosphate dehydrogenase 1-like, Chdh - choline dehydrogenase, Cyp2c67 - cytochrome p450, family 2, subfamily c, polypeptide 67, Kdm3a - lysine (k)-specific demethylase 3a, Cyp3a59 - cytochrome p450, family 3, subfamily a, polypeptide 59, Rtn4ip1 - reticulon 4 interacting protein 1, Dhrs7b - dehydrogenase/reductase (sdr family) member 7b, Hsd17b10 - hydroxysteroid (17-beta) dehydrogenase 10, Pdhb - pyruvate dehydrogenase (lipoamide) beta, Rrm2 - ribonucleotide reductase m2, Nr4a3 - nuclear receptor subfamily 4, group a, member 3, Sccpdh - saccharopine dehydrogenase (putative), Cyp2c55 - cytochrome p450, family 2, subfamily c, polypeptide 55, Gck - glucokinase, Tbxas1 - thromboxane a synthase 1, platelet, Dpyd - dihydropyrimidine dehydrogenase, Ugp2 - udp-glucose pyrophosphorylase 2, Dio1 - deiodinase, iodothyronine, type i, G6pdx - glucose-6-phosphate dehydrogenase x-linked, Aldob - aldolase b, fructose-bisphosphate, Cyp4a31 - cytochrome p450, family 4, subfamily a, polypeptide 31, Pgd - phosphogluconate dehydrogenase, Cyp4a32 - cytochrome p450, family 4, subfamily a, polypeptide 32, Cyp1a1 - cytochrome p450, family 1, subfamily a, polypeptide 1, Cyb561 - cytochrome b-561, Abcd2 - atp-binding cassette, sub-family d (ald), member 2, Cyp2c54 - cytochrome p450, family 2, subfamily c, polypeptide 54, Cycs - cytochrome c, somatic, Kdm6a - lysine (k)-specific demethylase 6a, Cyp2c37 - cytochrome p450, family 2. subfamily c, polypeptide 37, Tet3 - tet methylcytosine dioxygenase 3, Cyp2c29 - cytochrome p450, family 2, subfamily c, polypeptide 29, Cyp2b9 - cytochrome p450, family 2, subfamily b, polypeptide 9, Cyp2s1 - cytochrome p450, family 2, subfamily s, polypeptide 1, Ndufs5 - nadh dehydrogenase (ubiquinone) fe-s protein 5, Aox3 - aldehyde oxidase 3, Jhdm1d - jumonji c domain-containing histone demethylase 1 homolog d (s. cerevisiae), Cyp2b13 - cytochrome p450, family 2, subfamily b, polypeptide 13, Cyp2b10 - cytochrome p450, family 2, subfamily b, polypeptide 10, Ndufa8 - nadh dehydrogenase (ubiquinone) 1 alpha subcomplex, 8, Pir - pirin, Scd1 - stearoyl-coenzyme a desaturase 1, Loxl2 - lysyl oxidase-like 2, Cyp39a1 - cytochrome p450, family 39, subfamily a, polypeptide 1, Leprel2 - leprecan-like 2, Pgm2 - phosphoglucomutase 2, Cyp4f14 - cytochrome p450, family 4, subfamily f, polypeptide 14, Acat2 - acetyl-coenzyme a acetyltransferase 2, Gpi1 - glucose phosphate isomerase 1, Aifm3 - apoptosis-inducing factor, mitochondrion-associated 3, Gnmt - glycine n-methyltransferase, P4ha2 - procollagen-proline, 2-oxoglutarate 4-dioxygenase (proline 4-hydroxylase), alpha ii polypeptide, Mthfd1l - methylenetetrahydrofolate dehydrogenase (nadp+ dependent) 1-like, Bax - bcl2-associated x protein, Gfod1 - glucose-fructose oxidoreductase domain containing 1, Glrx - glutaredoxin, Kdm5b - lysine (k)-specific demethylase 5b, Cox6a1 - cytochrome c oxidase subunit via polypeptide 1, Cyb5r3 - cytochrome b5 reductase 3, Por - p450 (cytochrome) oxidoreductase, Cd36 - cd36 antigen, Fads6 - fatty acid desaturase domain family, member 6, Loxl1 - lysyl oxidase-like 1, Pygl - liver glycogen phosphorylase, Ptges - prostaglandin e synthase, Dhtkd1 - dehydrogenase e1 and transketolase domain containing 1, Steap2 - six transmembrane epithelial antigen of prostate 2, Acadsb - acyl-coenzyme a dehydrogenase, short/branched chain, Akr1c18 - aldo-keto reductase family 1, member c18, Cyp2d12 - cytochrome p450, family 2, subfamily d, polypeptide 12, Immp2l - imp2 inner mitochondrial membrane peptidase-like (s. cerevisiae), Mthfd1 - methylenetetrahydrofolate dehydrogenase (nadp+ dependent), methenyltetrahydrofolate cyclohydrolase, formyltetrahydrofolate synthase, Srxn1 - sulfiredoxin 1 homolog (s. cerevisiae), Rdh11 - retinol dehydrogenase 11, Mecp2 - methyl cpg binding protein 2, Dnajc10 - dnaj (hsp40) homolog, subfamily c, member 10, Aox1 - aldehyde oxidase 1, Ndufb3 - nadh dehydrogenase (ubiquinone) 1 beta subcomplex 3, Higd1a - hig1 domain family, member 1a, Cryl1 - crystallin, lambda 1, Cyp4a12a - cytochrome p450, family 4, subfamily a, polypeptide 12a, Nhlrc1 - nhl repeat containing 1, Hacl1 - 2-hydroxyacyl-coa lyase 1, Hpd - 4-hydroxyphenylpyruvic acid dioxygenase, Tmx4 - thioredoxin-related transmembrane protein 4, Cyp2c68 - cytochrome p450, family 2, subfamily c, polypeptide 68, Gys1 - glycogen synthase 1, muscle, Hr - hairless, Gyg - glycogenin, Tet2 - tet methylcytosine dioxygenase 2, Kdm5a - lysine (k)-specific demethylase 5a, Hsd17b12 - hydroxysteroid (17-beta) dehydrogenase 12, Sdr9c7 - 4short chain dehydrogenase/reductase family 9c, member 7]

GO: 0006690	eicosanoid metabolic process	1.68E-02	[Akr1c18 - aldo-keto reductase family 1, member c18, Ptgis - prostaglandin i2 (prostacyclin) synthase, Cyp2d12 - cytochrome p450, family 2, subfamily d, polypeptide 12, Cyp2j6 - cytochrome p450, family 2, subfamily j, polypeptide 6, Cyp4f14 - cytochrome p450, family 4, subfamily f, polypeptide 14, Cyp2d9 - cytochrome p450, family 2, subfamily d, polypeptide 9, Cyp2g1 - cytochrome p450, family 2, subfamily g, polypeptide 1, Cyp2c55 - cytochrome p450, family 2, subfamily c, polypeptide 55, Tbxas1 - thromboxane a synthase 1, platelet, Ltc4s - leukotriene c4 synthase, Ptgds - prostaglandin d2 synthase (brain), Cyp4a31 - cytochrome p450, family 4, subfamily a, polypeptide 31, Tnfrsf1a - tumor necrosis factor receptor superfamily, member 1a, Cyp4a32 - cytochrome p450, family 4, subfamily a, polypeptide 32, Akr1c20 - aldo-keto reductase family 1, member c20, Cyp2c54 - cytochrome p450, family 2, subfamily c, polypeptide 54, Cyp2c37 - cytochrome p450, family 2. subfamily c, polypeptide 37, Cyp2c29 - cytochrome p450, family 2, subfamily c, polypeptide 29, Cyp2c67 - cytochrome p450, family 2, subfamily c, polypeptide 67, Cyp2b9 - cytochrome p450, family 2, subfamily b, polypeptide 9, Cyp2c68 - cytochrome p450, family 2, subfamily c, polypeptide 68, Cyp2s1 - cytochrome p450, family 2, subfamily s, polypeptide 1, Pla2g4f - phospholipase a2, group ivf, Ptges - prostaglandin e synthase, Tlr2 - toll-like receptor 2, Cyp2b13 - cytochrome p450, family 2, subfamily b, polypeptide 13, Cyp2b10 - cytochrome p450, family 2, subfamily b, polypeptide 10, Cyp2a5 - cytochrome p450, family 2, subfamily a, polypeptide 5]
GO: 0046890	regulation of lipid biosynthetic process	1.90E-02	[C3 - complement component 3, Brca1 - breast cancer 1, Fabp5 - fatty acid binding protein 5, epidermal, Pdk4 - pyruvate dehydrogenase kinase, isoenzyme 4, Smpd3 - sphingomyelin phosphodiesterase 3, neutral, Por - p450 (cytochrome) oxidoreductase, Anxa1 - annexin a1, Wnt4 - wingless-related mmtv integration site 4, Apoa4 - apolipoprotein a-iv, Serpina12 - serine (or cysteine) peptidase inhibitor, clade a (alpha-1 antiproteinase, antitrypsin), member 12, Insig2 - insulin induced gene 2, Enho - energy homeostasis associated, Ccdc3 - coiled-coil domain containing 3, Cyp7a1 - cytochrome p450, family 7, subfamily a, polypeptide 1, Elovl5 - elovl family member 5, elongation of long chain fatty acids (yeast)]
GO: 0055086	nucleobase-containing small molecule metabolic process	1.92E-02	[Fasn - fatty acid synthase, Gpam - glycerol-3-phosphate acyltransferase, mitochondrial, Tkt - transketolase, Tk1 - thymidine kinase 1, Gpd2 - glycerol phosphate dehydrogenase 2, mitochondrial, Acot1 - acyl-coa thioesterase 1, Ctps - cytidine 5'-triphosphate synthase, Gpi1 - glucose phosphate isomerase 1, Entpd2 - ectonucleoside triphosphate diphosphohydrolase 2, Hmgcs1 - 3-hydroxy-3-methylglutaryl-coenzyme a synthase 1, Gnmt - glycine n-methyltransferase, Ahcy - s-adenosylhomocysteine hydrolase, Dpys - dihydropyrimidinase, Dpm1 - dolichol-phosphate (beta-d) mannosyltransferase 1, Pde5a - phosphodiesterase 5a, cgmp-specific, Nudt4 - nudix (nucleoside diphosphate linked moiety x)-type motif 4, Entpd7 - ectonucleoside triphosphate diphosphohydrolase 7, Pgam1 - phosphoglycerate mutase 1, Macrod2 - macro domain containing 2, Mthfd1l - methylenetetrahydrofolate dehydrogenase (nadp+ dependent) 1-like, Urad - ureidoimidazoline (2-oxo-4-hydroxy-4-carboxy-5) decarboxylase, Adcy3 - adenylate cyclase 3, Acly - atp citrate lyase, Acot9 - acyl-coa thioesterase 9, Acacb - acetyl-coenzyme a carboxylase beta, Acot11 - acyl-coa thioesterase 11, Dck - deoxycytidine kinase, Dnph1 - 2'-deoxynucleoside 5'-phosphate n-hydrolase 1, Nme6 - nme/nm23 nucleoside diphosphate kinase 6, Nudt1 - nudix (nucleoside diphosphate linked moiety x)-type motif 1, Acsm5 - acyl-coa synthetase medium-chain family member 5, Acot6 - acyl-coa thioesterase 6, Gpd1l - glycerol-3-phosphate dehydrogenase 1-like, Cacnb4 - calcium channel, voltage-dependent, beta 4 subunit, Taldo1 - transaldolase 1, Rpia - ribose 5-phosphate isomerase a, Dlat - dihydrolipoamide s-acetyltransferase (e2 component of pyruvate dehydrogenase complex), Oasl2 - 2'-5' oligoadenylate synthetase-like 2, Far2 - fatty acyl coa reductase 2, Ampd3 - adenosine monophosphate deaminase 3, Enpp3 - ectonucleotide pyrophosphatase/phosphodiesterase 3, Ada - adenosine deaminase, Papss2 - 3'-phosphoadenosine 5'-phosphosulfate synthase 2, Gapdhs - glyceraldehyde-3-phosphate dehydrogenase, spermatogenic, Acss1 - acyl-coa synthetase short-chain family member 1, Dhtkd1 - dehydrogenase e1 and transketolase domain containing 1, Gapdh - glyceraldehyde-3-phosphate dehydrogenase, Gne - glucosamine (udp-n-acetyl)-2-epimerase/n-acetylmannosamine kinase, Acadsb - acyl-coenzyme a dehydrogenase, short/branched chain, Atp2b2 - atpase, ca++ transporting, plasma membrane 2, Rhoq - ras homolog gene family, member q, Pdhb - pyruvate dehydrogenase (lipoamide) beta, Upp2 - uridine phosphorylase 2, Khk - ketohexokinase, Mthfd1 - methylenetetrahydrofolate dehydrogenase (nadp+ dependent),

			methenyltetrahydrofolate cyclohydrolase, formyltetrahydrofolate synthase, Cps1 - carbamoyl-phosphate synthetase 1, Gucy2c - guanylate cyclase 2c, Rrm2 - ribonucleotide reductase m2, Tdo2 - tryptophan 2,3-dioxygenase, Acss2 - acyl-coa synthetase short-chain family member 2, Gck - glucokinase, Cnp - 2',3'-cyclic nucleotide 3' phosphodiesterase, Gm3839 - glyceraldehyde-3-phosphate dehydrogenase pseudogene, Ugp2 - udp-glucose pyrophosphorylase 2, Dpyd - dihydropyrimidine dehydrogenase, Gale - galactose-4-epimerase, udp, Aox1 - aldehyde oxidase 1, Ttr - transthyretin, Nme1 - nme/nm23 nucleoside diphosphate kinase 1, Acnat2 - acyl-coenzyme a amino acid n-acyltransferase 2, Cs - citrate synthase, G6pdx - glucose-6-phosphate dehydrogenase x-linked, Aldob - aldolase b, fructose-bisphosphate, Pgd - phosphogluconate dehydrogenase, Agpat6 - 1-acylglycerol-3-phosphate o-acyltransferase 6 (lysophosphatidic acid acyltransferase, zeta), Pklr - pyruvate kinase liver and red blood cell, Atp1a2 - atpase, na+/k+ transporting, alpha 2 polypeptide, Ppat - phosphoribosyl pyrophosphate amidotransferase, Uap1l1 - udp-n-acteylglucosamine pyrophosphorylase 1-like 1, Acpp - acid phosphatase, prostate, Aox3 - aldehyde oxidase 3, Atp5k - atp synthase, h+ transporting, mitochondrial f1f0 complex, subunit e, Acaca - acetyl-coenzyme a carboxylase alpha, Hprt - hypoxanthine guanine phosphoribosyl transferase, Ehhadh - enoyl-coenzyme a, hydratase/3-hydroxyacyl coenzyme a dehydrogenase, Acsl5 - acyl-coa synthetase long-chain family member 5, Tet2 - tet methylcytosine dioxygenase 2, Nudt11 - nudix (nucleoside diphosphate linked moiety x)-type motif 11]
GO: 0006790	sulfur compound metabolic process	1.93E-02	[Fasn - fatty acid synthase, Acadsb - acyl-coenzyme a dehydrogenase, short/branched chain, Gpam - glycerol-3-phosphate acyltransferase, mitochondrial, Pdhb - pyruvate dehydrogenase (lipoamide) beta, Sult1c2 - sulfotransferase family, cytosolic, 1c, member 2, Acot1 - acyl-coa thioesterase 1, Cbs - cystathionine beta-synthase, Cps1 - carbamoyl-phosphate synthetase 1, Agxt - alanine-glyoxylate aminotransferase, Hmgcs1 - 3-hydroxy-3-methylglutaryl-coenzyme a synthase 1, Gnmt - glycine n-methyltransferase, Ahcy - s-adenosylhomocysteine hydrolase, Acss2 - acyl-coa synthetase short-chain family member 2, Gstm5 - glutathione s-transferase, mu 5, Gsta4 - glutathione s-transferase, alpha 4, Acly - atp citrate lyase, Acacb - acetyl-coenzyme a carboxylase beta, Acnat2 - acyl-coenzyme a amino acid n-acyltransferase 2, Mms19 - mms19 (met18 s. cerevisiae), Gpx3 - glutathione peroxidase 3, Gstt2 - glutathione s-transferase, theta 2, Cs - citrate synthase, G6pdx - glucose-6-phosphate dehydrogenase x-linked, Acot11 - acyl-coa thioesterase 11, Dcn - decorin, Hscb - hscb iron-sulfur cluster co-chaperone homolog (e. coli), Agpat6 - 1-acylglycerol-3-phosphate o-acyltransferase 6 (lysophosphatidic acid acyltransferase, zeta), Ggt6 - gamma-glutamyltransferase 6, Gsta2 - glutathione s-transferase, alpha 2 (yc2), Acpp - acid phosphatase, prostate, Slc35d1 - solute carrier family 35 (udp-glucuronic acid/udp-n-acetylgalactosamine dual transporter), member d1, Acaca - acetyl-coenzyme a carboxylase alpha, Cth - cystathionase (cystathionine gamma-lyase), Csad - cysteine sulfinic acid decarboxylase, Ptges - prostaglandin e synthase, Stat5a - signal transducer and activator of transcription 5a, Papss2 - 3'-phosphoadenosine 5'-phosphosulfate synthase 2, Angpt1 - angiopoietin 1, Sulf2 - sulfatase 2]
GO: 0044262	cellular carbohydrate metabolic process	2.02E-02	[Slc5a3 - solute carrier family 5 (inositol transporters), member 3, Synj2 - synaptojanin 2, Impa2 - inositol (myo)-1(or 4)-monophosphatase 2, Pck1 - phosphoenolpyruvate carboxykinase 1, cytosolic, Pgd - phosphogluconate dehydrogenase, Nhlrc1 - nhl repeat containing 1, Pgm2 - phosphoglucomutase 2, Abcg2 - atp-binding cassette, sub-family g (white), member 2, Pgp - phosphoglycolate phosphatase, Mogat1 - monoacylglycerol o-acyltransferase 1, Gnmt - glycine n-methyltransferase, Gck - glucokinase, Mtmr7 - myotubularin related protein 7, B4galt5 - udp-gal:betaglcnac beta 1,4-galactosyltransferase, polypeptide 5, Gys1 - glycogen synthase 1, muscle, Gusb - glucuronidase, beta, Got1 - glutamate oxaloacetate transaminase 1, soluble, Gyg - glycogenin, Pygl - liver glycogen phosphorylase, Ugp2 - udp-glucose pyrophosphorylase 2, Gbe1 - glucan (1,4-alpha-), branching enzyme 1, Treh - trehalase (brush-border membrane glycoprotein), Ppip5k1 - diphosphoinositol pentakisphosphate kinase 1, Extl1 - exostoses (multiple)-like 1]
GO: 1901615	organic hydroxy compound metabolic process	2.27E-02	[Med1 - mediator complex subunit 1, Atp2b2 - atpase, ca++ transporting, plasma membrane 2, Slc5a3 - solute carrier family 5 (inositol transporters), member 3, Ebpl - emopamil binding protein-like, Pck1 - phosphoenolpyruvate carboxykinase 1, cytosolic, Cyp39a1 - cytochrome p450, family 39, subfamily a, polypeptide 1, Cyp46a1 - cytochrome p450, family 46, subfamily a, polypeptide 1, Agtr1a - angiotensin ii receptor, type 1a, Pgp - phosphoglycolate phosphatase, Mogat1 - monoacylglycerol o-acyltransferase 1, Cln6 - ceroid-lipofuscinosis, neuronal 6, Rdh11 - retinol dehydrogenase 11, Mtmr7 - myotubularin related protein 7, Ip6k2 - inositol hexaphosphate

			kinase 2, Gcnt4 - glucosaminyl (n-acetyl) transferase 4, core 2 (beta-1,6-n-acetylglucosaminyltransferase), Ptafr - platelet-activating factor receptor, Gba2 - glucosidase beta 2, Got1 - glutamate oxaloacetate transaminase 1, soluble, Pcsk9 - proprotein convertase subtilisin/kexin type 9, Dio1 - deiodinase, iodothyronine, type i, Sptlc2 - serine palmitoyltransferase, long chain base subunit 2, Gch1 - gtp cyclohydrolase 1, Ttr - transthyretin, Insig2 - insulin induced gene 2, Cyp7a1 - cytochrome p450, family 7, subfamily a, polypeptide 1, Saa1 - serum amyloid a 1, Ppip5k1 - diphosphoinositol pentakisphosphate kinase 1, Pah - phenylalanine hydroxylase, Itpkc - inositol 1,4,5-trisphosphate 3-kinase c, G6pdx - glucose-6-phosphate dehydrogenase x-linked, Synj2 - synaptojanin 2, Impa2 - inositol (myo)-1(or 4)-monophosphatase 2, Cyp4a32 - cytochrome p450, family 4, subfamily a, polypeptide 32, Vldlr - very low density lipoprotein receptor, Sorl1 - sortilin-related receptor, ldlr class a repeats-containing, Cyp1a1 - cytochrome p450, family 1, subfamily a, polypeptide 1, Nr4a2 - nuclear receptor subfamily 4, group a, member 2, Lrp1 - low density lipoprotein receptor-related protein 1, Fdps - farnesyl diphosphate synthetase, Srd5a2 - steroid 5 alpha-reductase 2, Plcd3 - phospholipase c, delta 3, Slc16a10 - solute carrier family 16 (monocarboxylic acid transporters), member 10, Apoa4 - apolipoprotein a-iv, Adrbk2 - adrenergic receptor kinase, beta 2, Dct - dopachrome tautomerase]
GO: 0051156	glucose 6-phosphate metabolic process	2.41E-02	[Gck - glucokinase, G6pdx - glucose-6-phosphate dehydrogenase x-linked, Tkt - transketolase, Rpia - ribose 5-phosphate isomerase a, Taldo1 - transaldolase 1, Pgd - phosphogluconate dehydrogenase, G6pc3 - glucose 6 phosphatase, catalytic, 3, Gpi1 - glucose phosphate isomerase 1]
GO: 0032429	regulation of phospholipase A2 activity	2.55E-02	[Anxa1 - annexin a1, Ang - angiogenin, ribonuclease, rnase a family, 5, Agtr1a - angiotensin ii receptor, type 1a, Anxa8 - annexin a8, Lrp1 - low density lipoprotein receptor-related protein 1]
GO: 0072521	purine-containing compound metabolic process	2.50E-02	[Fasn - fatty acid synthase, Gpam - glycerol-3-phosphate acyltransferase, mitochondrial, Acot1 - acyl-coa thioesterase 1, Gpi1 - glucose phosphate isomerase 1, Hmgcs1 - 3-hydroxy-3-methylglutaryl-coenzyme a synthase 1, Ahcy - s-adenosylhomocysteine hydrolase, Gnmt - glycine n-methyltransferase, Pde5a - phosphodiesterase 5a, cgmp-specific, Nudt4 - nudix (nucleoside diphosphate linked moiety x)-type motif 4, Pgam1 - phosphoglycerate mutase 1, Mthfd1l - methylenetetrahydrofolate dehydrogenase (nadp+ dependent) 1-like, Macrod2 - macro domain containing 2, Urad - ureidoimidazoline (2-oxo-4-hydroxy-4-carboxy-5) decarboxylase, Adcy3 - adenylate cyclase 3, Acly - atp citrate lyase, Acot9 - acyl-coa thioesterase 9, Acacb - acetyl-coenzyme a carboxylase beta, Acot11 - acyl-coa thioesterase 11, Nme6 - nme/nm23 nucleoside diphosphate kinase 6, Nudt1 - nudix (nucleoside diphosphate linked moiety x)-type motif 1, Acsm5 - acyl-coa synthetase medium-chain family member 5, Acot6 - acyl-coa thioesterase 6, Cacnb4 - calcium channel, voltage-dependent, beta 4 subunit, Dlat - dihydrolipoamide s-acetyltransferase (e2 component of pyruvate dehydrogenase complex), Oasl2 - 2'-5' oligoadenylate synthetase-like 2, Far2 - fatty acyl coa reductase 2, Ampd3 - adenosine monophosphate deaminase 3, Enpp3 - ectonucleotide pyrophosphatase/phosphodiesterase 3, Ada - adenosine deaminase, Papss2 - 3'-phosphoadenosine 5'-phosphosulfate synthase 2, Gapdhs - glyceraldehyde-3-phosphate dehydrogenase, spermatogenic, Acss1 - acyl-coa synthetase short-chain family member 1, Dhtkd1 - dehydrogenase e1 and transketolase domain containing 1, Gapdh - glyceraldehyde-3-phosphate dehydrogenase, Rhoq - ras homolog gene family, member q, Acadsb - acyl-coenzyme a dehydrogenase, short/branched chain, Atp2b2 - atpase, ca++ transporting, plasma membrane 2, Pdhb - pyruvate dehydrogenase (lipoamide) beta, Khk - ketohexokinase, Mthfd1 - methylenetetrahydrofolate dehydrogenase (nadp+ dependent), methenyltetrahydrofolate cyclohydrolase, formyltetrahydrofolate synthase, Gucy2c - guanylate cyclase 2c, Tdo2 - tryptophan 2,3-dioxygenase, Gck - glucokinase, Acss2 - acyl-coa synthetase short-chain family member 2, Gm3839 - glyceraldehyde-3-phosphate dehydrogenase pseudogene, Gale - galactose-4-epimerase, udp, Dpyd - dihydropyrimidine dehydrogenase, Aox1 - aldehyde oxidase 1, Ttr - transthyretin, Nme1 - nme/nm23 nucleoside diphosphate kinase 1, Acnat2 - acyl-coenzyme a amino acid n-acyltransferase 2, Cs - citrate synthase, Aldob - aldolase b, fructose-bisphosphate, Agpat6 - 1-acylglycerol-3-phosphate o-acyltransferase 6 (lysophosphatidic acid acyltransferase, zeta), Pklr - pyruvate kinase liver and red blood cell, Slc2a9 - solute carrier family 2 (facilitated glucose transporter), member 9, Atp1a2 - atpase, na+/k+ transporting, alpha 2 polypeptide, Abcg2 - atp-binding cassette, sub-family g (white), member 2, Acpp - acid phosphatase, prostate, Aox3 - aldehyde oxidase 3, Atp5k -

			atp synthase, h+ transporting, mitochondrial f1f0 complex, subunit e, Acaca - acetyl-coenzyme a carboxylase alpha, Hprt - hypoxanthine guanine phosphoribosyl transferase, Ehhadh - enoyl-coenzyme a, hydratase/3-hydroxyacyl coenzyme a dehydrogenase, Acsl5 - acyl-coa synthetase long-chain family member 5, Nudt11 - nudix (nucleoside diphosphate linked moiety x)-type motif 11]
GO: 0009719	response to endogenous stimulus	2.48E-02	[Ar - androgen receptor, Dll4 - delta-like 4 (drosophila), Adamts12 - a disintegrin-like and metallopeptidase (reprolysin type) with thrombospondin type 1 motif, 12, Ntrk1 - neurotrophic tyrosine kinase, receptor, type 1, Hsd3b2 - hydroxy-delta-5-steroid dehydrogenase, 3 beta- and steroid delta-isomerase 2, Prdm2 - pr domain containing 2, with znf domain, Ntrk2 - neurotrophic tyrosine kinase, receptor, type 2, Tmem38a - transmembrane protein 38a, Hmgcs1 - 3-hydroxy-3-methylglutaryl-coenzyme a synthase 1, Pde3a - phosphodiesterase 3a, cgmp inhibited, Prkca - protein kinase c, alpha, Pcsk9 - proprotein convertase subtilisin/kexin type 9, Prkce - protein kinase c, epsilon, Sh3bp4 - sh3-domain binding protein 4, Lamtor4 - late endosomal/lysosomal adaptor, mapk and mtor activator 4, Abhd2 - abhydrolase domain containing 2, Pde3b - phosphodiesterase 3b, cgmp-inhibited, Gata6 - gata binding protein 6, Zfp36 - zinc finger protein 36, P2rx3 - purinergic receptor p2x, ligand-gated ion channel, 3, Gata5 - gata binding protein 5, Kit - kit oncogene, Smpd3 - sphingomyelin phosphodiesterase 3, neutral, Pdgfc - platelet-derived growth factor, c polypeptide, Ass1 - argininosuccinate synthetase 1, Actb - actin, beta, Mapk15 - mitogen-activated protein kinase 15, Amigo1 - adhesion molecule with ig like domain 1, Irs2 - insulin receptor substrate 2, Stat5a - signal transducer and activator of transcription 5a, Akap8 - a kinase (prka) anchor protein 8, Npc1 - niemann pick type c1, Slc6a4 - solute carrier family 6 (neurotransmitter transporter, serotonin), member 4, Runx3 - runt related transcription factor 3, Pck1 - phosphoenolpyruvate carboxykinase 1, cytosolic, Icam1 - intercellular adhesion molecule 1, Col3a1 - collagen, type iii, alpha 1, Col4a2 - collagen, type iv, alpha 2, Mus81 - mus81 endonuclease homolog (yeast), Trem2 - triggering receptor expressed on myeloid cells 2, Slc27a1 - solute carrier family 27 (fatty acid transporter), member 1, Nr4a3 - nuclear receptor subfamily 4, group a, member 3, Hyal1 - hyaluronoglucosaminidase 1, Gabrb3 - gamma-aminobutyric acid (gaba) a receptor, subunit beta 3, Gck - glucokinase, Src - rous sarcoma oncogene, Ccr7 - chemokine (c-c motif) receptor 7, Aldob - aldolase b, fructose-bisphosphate, Tat - tyrosine aminotransferase, Pklr - pyruvate kinase liver and red blood cell, Casp4 - caspase 4, apoptosis-related cysteine peptidase, Smad3 - smad family member 3, Insr - insulin receptor, Smad7 - smad family member 7, Srd5a2 - steroid 5 alpha-reductase 2, Esr1 - estrogen receptor 1 (alpha), Socs2 - suppressor of cytokine signaling 2, Serpine1 - serine (or cysteine) peptidase inhibitor, clade e, member 1, Rragd - ras-related gtp binding d, Acaca - acetyl-coenzyme a carboxylase alpha, P2ry4 - pyrimidinergic receptor p2y, g-protein coupled, 4, Lpin3 - lipin 3, Cib2 - calcium and integrin binding family member 2, Plcd1 - phospholipase c, delta 1, Med1 - mediator complex subunit 1, Gpam - glycerol-3-phosphate acyltransferase, mitochondrial, Agtr1a - angiotensin ii receptor, type 1a, Agxt - alanine-glyoxylate aminotransferase, Ramp1 - receptor (calcitonin) activity modifying protein 1, Serpina1e - serine (or cysteine) peptidase inhibitor, clade a, member 1e, Jup - junction plakoglobin, Mmp2 - matrix metallopeptidase 2, Cd9 - cd9 antigen, Jun - jun oncogene, Timp1 - tissue inhibitor of metalloproteinase 1, Rangap1 - ran gtpase activating protein 1, Mmp13 - matrix metallopeptidase 13, Timp2 - tissue inhibitor of metalloproteinase 2, Eme1 - essential meiotic endonuclease 1 homolog 1 (s. pombe), Cd81 - cd81 antigen, Ddit4 - dna-damage-inducible transcript 4, Glrx - glutaredoxin, Wt1 - wilms tumor 1 homolog, Me1 - malic enzyme 1, nadp(+)-dependent, cytosolic, Vim - vimentin, Itgb1bp1 - integrin beta 1 binding protein 1, Lrp1 - low density lipoprotein receptor-related protein 1, Sox6 - sry-box containing gene 6, Por - p450 (cytochrome) oxidoreductase, Anxa1 - annexin a1, Cd36 - cd36 antigen, Col1a2 - collagen, type i, alpha 2, Ang - angiogenin, ribonuclease, rnase a family, 5, Col1a1 - collagen, type i, alpha 1, Slc30a10 - solute carrier family 30, member 10, Cpeb4 - cytoplasmic polyadenylation element binding protein 4, Itpr2 - inositol 1,4,5-triphosphate receptor 2, Epha4 - eph receptor a4, Epha3 - eph receptor a3, Col6a1 - collagen, type vi, alpha 1, Steap2 - six transmembrane epithelial antigen of prostate 2, Rhoq - ras homolog gene family, member q, Akr1c18 - aldo-keto reductase family 1, member c18, Anxa5 - annexin a5, Ednrb - endothelin receptor type b, Brca1 - breast cancer 1, Khk - ketohexokinase, Ogt - o-linked n-acetylglucosamine (glcnac) transferase (udp-n-acetylglucosamine:polypeptide-n-acetylglucosaminyl transferase), Sirt1 - sirtuin 1,

			Adamts7 - a disintegrin-like and metallopeptidase (reprolysin type) with thrombospondin type 1 motif, 7, Trpv4 - transient receptor potential cation channel, subfamily v, member 4, Trpm4 - transient receptor potential cation channel, subfamily m, member 4, Bmp7 - bone morphogenetic protein 7, Mecp2 - methyl cpg binding protein 2, Bmp6 - bone morphogenetic protein 6, Bmp4 - bone morphogenetic protein 4, Zfp259 - zinc finger protein 259, Ehd1 - eh-domain containing 1, Cdh1 - cadherin 1, Atp1a2 - atpase, na+/k+ transporting, alpha 2 polypeptide, Nr4a2 - nuclear receptor subfamily 4, group a, member 2, Epb4.1l5 - erythrocyte protein band 4.1-like 5, Slc26a6 - solute carrier family 26, member 6, Lcat - lecithin cholesterol acyltransferase, Tlr2 - toll-like receptor 2, Egr1 - early growth response 1, Rapgef2 - rap guanine nucleotide exchange factor (gef) 2, Egr2 - early growth response 2, Uchl3 - ubiquitin carboxyl-terminal esterase l3 (ubiquitin thiolesterase)]
GO: 1901135	carbo-hydrate derivative metabolic process	2.46E-02	[Fasn - fatty acid synthase, Gpam - glycerol-3-phosphate acyltransferase, mitochondrial, Gpc1 - glypican 1, Tkt - transketolase, Tk1 - thymidine kinase 1, Adamts12 - a disintegrin-like and metallopeptidase (reprolysin type) with thrombospondin type 1 motif, 12, Gpd2 - glycerol phosphate dehydrogenase 2, mitochondrial, Acot1 - acyl-coa thioesterase 1, Ctps - cytidine 5'-triphosphate synthase, Gpi1 - glucose phosphate isomerase 1, Cln6 - ceroid-lipofuscinosis, neuronal 6, Hmgcs1 - 3-hydroxy-3-methylglutaryl-coenzyme a synthase 1, Gnmt - glycine n-methyltransferase, Ahcy - s-adenosylhomocysteine hydrolase, Gba2 - glucosidase beta 2, Ihh - indian hedgehog, Dpm1 - dolichol-phosphate (beta-d) mannosyltransferase 1, Pde5a - phosphodiesterase 5a, cgmp-specific, Macrod2 - macro domain containing 2, Bax - bcl2-associated x protein, Mmp12 - matrix metallopeptidase 12, Acly - atp citrate lyase, Acacb - acetyl-coenzyme a carboxylase beta, Fbxo44 - f-box protein 44, Acot11 - acyl-coa thioesterase 11, Pigp - phosphatidylinositol glycan anchor biosynthesis, class p, Dck - deoxycytidine kinase, Dcn - decorin, Nme6 - nme/nm23 nucleoside diphosphate kinase 6, Nudt1 - nudix (nucleoside diphosphate linked moiety x)-type motif 1, Tnip1 - tnfaip3 interacting protein 1, Akr1c20 - aldo-keto reductase family 1, member c20, A4gnt - alpha-1,4-n-acetylglucosaminyltransferase, Pdgfrb - platelet derived growth factor receptor, beta polypeptide, Taldo1 - transaldolase 1, Rpia - ribose 5-phosphate isomerase a, Pygl - liver glycogen phosphorylase, Itih2 - inter-alpha trypsin inhibitor, heavy chain 2, Enpp3 - ectonucleotide pyrophosphatase/phosphodiesterase 3, Papss2 - 3'-phosphoadenosine 5'-phosphosulfate synthase 2, Fbxo6 - f-box protein 6, Gapdhs - glyceraldehyde-3-phosphate dehydrogenase, spermatogenic, Dhtkd1 - dehydrogenase e1 and transketolase domain containing 1, Sulf2 - sulfatase 2, Gapdh - glyceraldehyde-3-phosphate dehydrogenase, Extl1 - exostoses (multiple)-like 1, Mppe1 - metallophosphoesterase 1, Acadsb - acyl-coenzyme a dehydrogenase, short/branched chain, Atp2b2 - atpase, ca++ transporting, plasma membrane 2, Rhoq - ras homolog gene family, member q, Akr1c18 - aldo-keto reductase family 1, member c18, Pdhb - pyruvate dehydrogenase (lipoamide) beta, Upp2 - uridine phosphorylase 2, Khk - ketohexokinase, Ogt - o-linked n-acetylglucosamine (glcnac) transferase (udp-n-acetylglucosamine:polypeptide-n-acetylglucosaminyl transferase), Gucy2c - guanylate cyclase 2c, Rrm2 - ribonucleotide reductase m2, Hyal1 - hyaluronoglucosaminidase 1, Sccpdh - saccharopine dehydrogenase (putative), Gck - glucokinase, Acss2 - acyl-coa synthetase short-chain family member 2, Gm3839 - glyceraldehyde-3-phosphate dehydrogenase pseudogene, Ugp2 - udp-glucose pyrophosphorylase 2, Dpyd - dihydropyrimidine dehydrogenase, Gale - galactose-4-epimerase, udp, Acnat2 - acyl-coenzyme a amino acid n-acyltransferase 2, Nme1 - nme/nm23 nucleoside diphosphate kinase 1, Fuca2 - fucosidase, alpha-l- 2, plasma, St8sia3 - st8 alpha-n-acetyl-neuraminide alpha-2,8-sialyltransferase 3, Cs - citrate synthase, G6pdx - glucose-6-phosphate dehydrogenase x-linked, Pgd - phosphogluconate dehydrogenase, Agpat6 - 1-acylglycerol-3-phosphate o-acyltransferase 6 (lysophosphatidic acid acyltransferase, zeta), Pklr - pyruvate kinase liver and red blood cell, Mdp1 - magnesium-dependent phosphatase 1, Atp1a2 - atpase, na+/k+ transporting, alpha 2 polypeptide, Ppat - phosphoribosyl pyrophosphate amidotransferase, Hexa - hexosaminidase a, Abcg2 - atp-binding cassette, sub-family g (white), member 2, Hexb - hexosaminidase b, Uap1l1 - udp-n-acteylglucosamine pyrophosphorylase 1-like 1, Acpp - acid phosphatase, prostate, B4galt5 - udp-gal:betaglcnac beta 1,4-galactosyltransferase, polypeptide 5, Slc35d1 - solute carrier family 35 (udp-glucuronic acid/udp-n-acetylgalactosamine dual transporter), member d1, Alg12 - asparagine-linked glycosylation 12 (alpha-1,6-mannosyltransferase), Gusb - glucuronidase, beta, Acaca - acetyl-coenzyme a carboxylase alpha, Pgap1 - post-gpi attachment to proteins 1, Angpt1 - angiopoietin 1, Nudt11 - nudix (nucleoside diphosphate linked moiety x)-type motif 11]

GO: 0006066	alcohol metabolic process	2.71E-02	[Slc5a3 - solute carrier family 5 (inositol transporters), member 3, Pck1 - phosphoenolpyruvate carboxykinase 1, cytosolic, Cyp39a1 - cytochrome p450, family 39, subfamily a, polypeptide 1, Cyp46a1 - cytochrome p450, family 46, subfamily a, polypeptide 1, Pgp - phosphoglycolate phosphatase, Mogat1 - monoacylglycerol o-acyltransferase 1, Cln6 - ceroid-lipofuscinosis, neuronal 6, Hmgcs1 - 3-hydroxy-3-methylglutaryl-coenzyme a synthase 1, Rdh11 - retinol dehydrogenase 11, Mtmr7 - myotubularin related protein 7, Ip6k2 - inositol hexaphosphate kinase 2, Ptafr - platelet-activating factor receptor, Gba2 - glucosidase beta 2, Got1 - glutamate oxaloacetate transaminase 1, soluble, Dpm1 - dolichol-phosphate (beta-d) mannosyltransferase 1, Pcsk9 - proprotein convertase subtilisin/kexin type 9, Sptlc2 - serine palmitoyltransferase, long chain base subunit 2, Scarb1 - scavenger receptor class b, member 1, Gch1 - gtp cyclohydrolase 1, Ttr - transthyretin, Lrat - lecithin-retinol acyltransferase (phosphatidylcholine-retinol-o-acyltransferase), Insig2 - insulin induced gene 2, Cyp7a1 - cytochrome p450, family 7, subfamily a, polypeptide 1, Saa1 - serum amyloid a 1, Ppip5k1 - diphosphoinositol pentakisphosphate kinase 1, Pmp22 - peripheral myelin protein 22, Pah - phenylalanine hydroxylase, Itpkc - inositol 1,4,5-trisphosphate 3-kinase c, G6pdx - glucose-6-phosphate dehydrogenase x-linked, Synj2 - synaptojanin 2, Impa2 - inositol (myo)-1(or 4)-monophosphatase 2, Vldlr - very low density lipoprotein receptor, Sorl1 - sortilin-related receptor, ldlr class a repeats-containing, Cyp1a1 - cytochrome p450, family 1, subfamily a, polypeptide 1, Akr1c20 - aldo-keto reductase family 1, member c20, Lrp1 - low density lipoprotein receptor-related protein 1, Fdps - farnesyl diphosphate synthetase, Plcd3 - phospholipase c, delta 3, Apoa4 - apolipoprotein a-iv, Adrbk2 - adrenergic receptor kinase, beta 2, Nudt11 - nudix (nucleoside diphosphate linked moiety x)-type motif 11]
GO: 0019637	organo-phosphate metabolic process	2.77E-02	[Fasn - fatty acid synthase, Gpd2 - glycerol phosphate dehydrogenase 2, mitochondrial, Pgp - phosphoglycolate phosphatase, Hmgcs1 - 3-hydroxy-3-methylglutaryl-coenzyme a synthase 1, Ptafr - platelet-activating factor receptor, Dpm1 - dolichol-phosphate (beta-d) mannosyltransferase 1, Nudt4 - nudix (nucleoside diphosphate linked moiety x)-type motif 4, Pde5a - phosphodiesterase 5a, cgmp-specific, Pcsk9 - proprotein convertase subtilisin/kexin type 9, Pgam1 - phosphoglycerate mutase 1, Acot9 - acyl-coa thioesterase 9, Dck - deoxycytidine kinase, Dnph1 - 2'-deoxynucleoside 5'-phosphate n-hydrolase 1, Nudt1 - nudix (nucleoside diphosphate linked moiety x)-type motif 1, Fabp5 - fatty acid binding protein 5, epidermal, Gata6 - gata binding protein 6, Smpd3 - sphingomyelin phosphodiesterase 3, neutral, Pik3c2g - phosphatidylinositol 3-kinase, c2 domain containing, gamma polypeptide, Gpd1l - glycerol-3-phosphate dehydrogenase 1-like, Pdgfrb - platelet derived growth factor receptor, beta polypeptide, Pla2g16 - phospholipase a2, group xvi, Cacnb4 - calcium channel, voltage-dependent, beta 4 subunit, Rpia - ribose 5-phosphate isomerase a, Pik3r3 - phosphatidylinositol 3 kinase, regulatory subunit, polypeptide 3 (p55), Plcd3 - phospholipase c, delta 3, Enpp2 - ectonucleotide pyrophosphatase/phosphodiesterase 2, Cds2 - cdp-diacylglycerol synthase (phosphatidate cytidylyltransferase) 2, Ada - adenosine deaminase, Adrbk2 - adrenergic receptor kinase, beta 2, Gapdhs - glyceraldehyde-3-phosphate dehydrogenase, spermatogenic, Mppe1 - metallophosphoesterase 1, Gapdh - glyceraldehyde-3-phosphate dehydrogenase, Atp2b2 - atpase, ca++ transporting, plasma membrane 2, Sgms2 - sphingomyelin synthase 2, Pdhb - pyruvate dehydrogenase (lipoamide) beta, Cps1 - carbamoyl-phosphate synthetase 1, Slc27a1 - solute carrier family 27 (fatty acid transporter), member 1, Rrm2 - ribonucleotide reductase m2, Acss2 - acyl-coa synthetase short-chain family member 2, Gck - glucokinase, Cnp - 2',3'-cyclic nucleotide 3' phosphodiesterase, Ugp2 - udp-glucose pyrophosphorylase 2, Sptlc2 - serine palmitoyltransferase, long chain base subunit 2, Acnat2 - acyl-coenzyme a amino acid n-acyltransferase 2, Nme1 - nme/nm23 nucleoside diphosphate kinase 1, G6pdx - glucose-6-phosphate dehydrogenase x-linked, Aldob - aldolase b, fructose-bisphosphate, Pgd - phosphogluconate dehydrogenase, Pklr - pyruvate kinase liver and red blood cell, Agpat2 - 1-acylglycerol-3-phosphate o-acyltransferase 2 (lysophosphatidic acid acyltransferase, beta), Hexb - hexosaminidase b, Vac14 - vac14 homolog (s. cerevisiae), Fdps - farnesyl diphosphate synthetase, Pnpla3 - patatin-like phospholipase domain containing 3, Socs2 - suppressor of cytokine signaling 2, Atp5k - atp synthase, h+ transporting, mitochondrial f1f0 complex, subunit e, Acaca - acetyl-coenzyme a carboxylase alpha, Pgap1 - post-gpi attachment to proteins 1, Plcd1 - phospholipase c, delta 1, Plscr1 - phospholipid scramblase 1, Gpam - glycerol-3-phosphate acyltransferase, mitochondrial, Fam126a - family with sequence similarity 126, member a, Tkt - transketolase, Acot1 - acyl-coa thioesterase 1, Ctps - cytidine 5'-triphosphate synthase, Gpi1 - glucose phosphate isomerase 1,

			Entpd2 - ectonucleoside triphosphate diphosphohydrolase 2, Mtmr7 - myotubularin related protein 7, Ip6k2 - inositol hexaphosphate kinase 2, Entpd7 - ectonucleoside triphosphate diphosphohydrolase 7, Adcy3 - adenylate cyclase 3, Acly - atp citrate lyase, Lrat - lecithin-retinol acyltransferase (phosphatidylcholine-retinol-o-acyltransferase), Samd8 - sterile alpha motif domain containing 8, Acacb - acetyl-coenzyme a carboxylase beta, Itpkc - inositol 1,4,5-trisphosphate 3-kinase c, Pigp - phosphatidylinositol glycan anchor biosynthesis, class p, Acot11 - acyl-coa thioesterase 11, Itpka - inositol 1,4,5-trisphosphate 3-kinase a, Impa2 - inositol (myo)-1(or 4)-monophosphatase 2, Nme6 - nme/nm23 nucleoside diphosphate kinase 6, Acsm5 - acyl-coa synthetase medium-chain family member 5, Taldo1 - transaldolase 1, Dlat - dihydrolipoamide s-acetyltransferase (e2 component of pyruvate dehydrogenase complex), Pygl - liver glycogen phosphorylase, Oasl2 - 2'-5' oligoadenylate synthetase-like 2, Pla2g4f - phospholipase a2, group ivf, Ampd3 - adenosine monophosphate deaminase 3, G6pc3 - glucose 6 phosphatase, catalytic, 3, Enpp3 - ectonucleotide pyrophosphatase/phosphodiesterase 3, Papss2 - 3'-phosphoadenosine 5'-phosphosulfate synthase 2, Serinc2 - serine incorporator 2, Acss1 - acyl-coa synthetase short-chain family member 1, Dhtkd1 - dehydrogenase e1 and transketolase domain containing 1, Acadsb - acyl-coenzyme a dehydrogenase, short/branched chain, Rhoq - ras homolog gene family, member q, Upp2 - uridine phosphorylase 2, Khk - ketohexokinase, Mthfd1 - methylenetetrahydrofolate dehydrogenase (nadp+ dependent), methenyltetrahydrofolate cyclohydrolase, formyltetrahydrofolate synthase, Gucy2c - guanylate cyclase 2c, Mecp2 - methyl cpg binding protein 2, Gm3839 - glyceraldehyde-3-phosphate dehydrogenase pseudogene, Gale - galactose-4-epimerase, udp, Fitm1 - fat storage-inducing transmembrane protein 1, Ppip5k1 - diphosphoinositol pentakisphosphate kinase 1, Cs - citrate synthase, Synj2 - synaptojanin 2, Agpat6 - 1-acylglycerol-3-phosphate o-acyltransferase 6 (lysophosphatidic acid acyltransferase, zeta), Atp1a2 - atpase, na+/k+ transporting, alpha 2 polypeptide, Ppat - phosphoribosyl pyrophosphate amidotransferase, Acpp - acid phosphatase, prostate, Apoa4 - apolipoprotein a-iv, Hprt - hypoxanthine guanine phosphoribosyl transferase, Smpd2 - sphingomyelin phosphodiesterase 2, neutral, Lcat - lecithin cholesterol acyltransferase, Acsl5 - acyl-coa synthetase long-chain family member 5, Nudt11 - nudix (nucleoside diphosphate linked moiety x)-type motif 11]
GO: 0008202	steroid metabolic process	2.88E-02	[Med1 - mediator complex subunit 1, Serpina6 - serine (or cysteine) peptidase inhibitor, clade a, member 6, Ebpl - emopamil binding protein-like, Akr1c18 - aldo-keto reductase family 1, member c18, Cyp3a25 - cytochrome p450, family 3, subfamily a, polypeptide 25, Scd1 - stearoyl-coenzyme a desaturase 1, Hsd3b2 - hydroxy-delta-5-steroid dehydrogenase, 3 beta- and steroid delta-isomerase 2, Cyp46a1 - cytochrome p450, family 46, subfamily a, polypeptide 1, Cyp39a1 - cytochrome p450, family 39, subfamily a, polypeptide 1, Cyp3a11 - cytochrome p450, family 3, subfamily a, polypeptide 11, Cyp3a13 - cytochrome p450, family 3, subfamily a, polypeptide 13, Cln6 - ceroid-lipofuscinosis, neuronal 6, Hmgcs1 - 3-hydroxy-3-methylglutaryl-coenzyme a synthase 1, Hsd17b6 - hydroxysteroid (17-beta) dehydrogenase 6, Gba2 - glucosidase beta 2, Mecp2 - methyl cpg binding protein 2, Pcsk9 - proprotein convertase subtilisin/kexin type 9, Scarb1 - scavenger receptor class b, member 1, Insig2 - insulin induced gene 2, Cyp7a1 - cytochrome p450, family 7, subfamily a, polypeptide 1, Saa1 - serum amyloid a 1, Pmp22 - peripheral myelin protein 22, G6pdx - glucose-6-phosphate dehydrogenase x-linked, Vldlr - very low density lipoprotein receptor, Sorl1 - sortilin-related receptor, ldlr class a repeats-containing, Cyp1a1 - cytochrome p450, family 1, subfamily a, polypeptide 1, Akr1c20 - aldo-keto reductase family 1, member c20, Cyb5r3 - cytochrome b5 reductase 3, Lrp1 - low density lipoprotein receptor-related protein 1, Fdps - farnesyl diphosphate synthetase, Srd5a2 - steroid 5 alpha-reductase 2, Afp - alpha fetoprotein, Esr1 - estrogen receptor 1 (alpha), Apoa4 - apolipoprotein a-iv, Lcat - lecithin cholesterol acyltransferase, Cyp3a59 - cytochrome p450, family 3, subfamily a, polypeptide 59, Akr1c19 - aldo-keto reductase family 1, member c19, Cyp2b10 - cytochrome p450, family 2, subfamily b, polypeptide 10, Plekha1 - pleckstrin homology domain containing, family a (phosphoinositide binding specific) member 1, Hsd17b12 - hydroxysteroid (17-beta) dehydrogenase 12]
GO: 0019216	regulation of lipid metabolic process	2.94E-02	[C3 - complement component 3, Brca1 - breast cancer 1, Fabp5 - fatty acid binding protein 5, epidermal, Pdk4 - pyruvate dehydrogenase kinase, isoenzyme 4, Smpd3 - sphingomyelin phosphodiesterase 3, neutral, Cidea - cell death-inducing dna fragmentation factor, alpha subunit-like effector a, Por - p450 (cytochrome) oxidoreductase, Cd36 - cd36 antigen, Anxa1 - annexin a1, Wnt4 - wingless-related mmtv integration site 4, Pik3r3 - phosphatidylinositol 3 kinase, regulatory subunit, polypeptide 3 (p55), Apoa4 -

			apolipoprotein a-iv, Ttc39b - tetratricopeptide repeat domain 39b, Irs2 - insulin receptor substrate 2, Serpina12 - serine (or cysteine) peptidase inhibitor, clade a (alpha-1 antiproteinase, antitrypsin), member 12, Stat5a - signal transducer and activator of transcription 5a, Enho - energy homeostasis associated, Insig2 - insulin induced gene 2, Cyp7a1 - cytochrome p450, family 7, subfamily a, polypeptide 1, Ccdc3 - coiled-coil domain containing 3, Src - rous sarcoma oncogene, Elovl5 - elovl family member 5, elongation of long chain fatty acids (yeast)]
GO: 0031668	cellular response to extracellular stimulus	3.43E-02	[Fcor - foxo1 corepressor, Pck1 - phosphoenolpyruvate carboxykinase 1, cytosolic, Glul - glutamate-ammonia ligase (glutamine synthetase), Hamp - hepcidin antimicrobial peptide, Bmf - bcl2 modifying factor, Bhlha15 - basic helix-loop-helix family, member a15, Tnrc6a - trinucleotide repeat containing 6a, P2rx3 - purinergic receptor p2x, ligand-gated ion channel, 3, Pdk4 - pyruvate dehydrogenase kinase, isoenzyme 4, Nr4a2 - nuclear receptor subfamily 4, group a, member 2, Atf3 - activating transcription factor 3, Gck - glucokinase, Gas6 - growth arrest specific 6, Wnt4 - wingless-related mmtv integration site 4, Rragd - ras-related gtp binding d, Bmp6 - bone morphogenetic protein 6, 5330417C22Rik - riken cdna 5330417c22 gene, Amigo1 - adhesion molecule with ig like domain 1, Cdkn1a - cyclin-dependent kinase inhibitor 1a (p21), Cib2 - calcium and integrin binding family member 2, Foxa3 - forkhead box a3, Rnase4 - ribonuclease, rnase a family 4, Slc38a3 - solute carrier family 38, member 3, Asns - asparagine synthetase]
GO: 0070988	demethylation	3.65E-02	[Cyp3a25 - cytochrome p450, family 3, subfamily a, polypeptide 25, Kdm5b - lysine (k)-specific demethylase 5b, Cyp3a11 - cytochrome p450, family 3, subfamily a, polypeptide 11, Cyp3a13 - cytochrome p450, family 3, subfamily a, polypeptide 13, Kdm6a - lysine (k)-specific demethylase 6a, Tet3 - tet methylcytosine dioxygenase 3, Por - p450 (cytochrome) oxidoreductase, Kdm3a - lysine (k)-specific demethylase 3a, Tdg - thymine dna glycosylase, Cyp3a59 - cytochrome p450, family 3, subfamily a, polypeptide 59, Jhdm1d - jumonji c domain-containing histone demethylase 1 homolog d (s. cerevisiae), Tet2 - tet methylcytosine dioxygenase 2, Kdm5a - lysine (k)-specific demethylase 5a]
GO: 0006637	acyl-CoA metabolic process	3.55E-02	[Acadsb - acyl-coenzyme a dehydrogenase, short/branched chain, Fasn - fatty acid synthase, Gpam - glycerol-3-phosphate acyltransferase, mitochondrial, Acot11 - acyl-coa thioesterase 11, Pdhb - pyruvate dehydrogenase (lipoamide) beta, Agpat6 - 1-acylglycerol-3-phosphate o-acyltransferase 6 (lysophosphatidic acid acyltransferase, zeta), Acot1 - acyl-coa thioesterase 1, Acsm5 - acyl-coa synthetase medium-chain family member 5, Tdo2 - tryptophan 2,3-dioxygenase, Hmgcs1 - 3-hydroxy-3-methylglutaryl-coenzyme a synthase 1, Acot6 - acyl-coa thioesterase 6, Acss2 - acyl-coa synthetase short-chain family member 2, Dlat - dihydrolipoamide s-acetyltransferase (e2 component of pyruvate dehydrogenase complex), Acaca - acetyl-coenzyme a carboxylase alpha, Far2 - fatty acyl coa reductase 2, Acly - atp citrate lyase, Ehhadh - enoyl-coenzyme a, hydratase/3-hydroxyacyl coenzyme a dehydrogenase, Acot9 - acyl-coa thioesterase 9, Acsl5 - acyl-coa synthetase long-chain family member 5, Acnat2 - acyl-coenzyme a amino acid n-acyltransferase 2, Acacb - acetyl-coenzyme a carboxylase beta, Acss1 - acyl-coa synthetase short-chain family member 1, Cs - citrate synthase]
GO: 0009112	nucleobase metabolic process	3.89E-02	[Mthfd1 - methylenetetrahydrofolate dehydrogenase (nadp+ dependent), methenyltetrahydrofolate cyclohydrolase, formyltetrahydrofolate synthase, Ctps - cytidine 5'-triphosphate synthase, Cps1 - carbamoyl-phosphate synthetase 1, Acpp - acid phosphatase, prostate, Dpys - dihydropyrimidinase, Aox3 - aldehyde oxidase 3, Mthfd1l - methylenetetrahydrofolate dehydrogenase (nadp+ dependent) 1-like, Urad - ureidoimidazoline (2-oxo-4-hydroxy-4-carboxy-5) decarboxylase, Dpyd - dihydropyrimidine dehydrogenase, Hprt - hypoxanthine guanine phosphoribosyl transferase, Ada - adenosine deaminase, Aox1 - aldehyde oxidase 1, Ttr - transthyretin, Tet2 - tet methylcytosine dioxygenase 2]
GO: 0032330	regulation of chondrocyte differentiation	4.06E-02	[Sox6 - sry-box containing gene 6, Por - p450 (cytochrome) oxidoreductase, Efemp1 - epidermal growth factor-containing fibulin-like extracellular matrix protein 1, Adamts12 - a disintegrin-like and metallopeptidase (reprolysin type) with thrombospondin type 1 motif, 12, Loxl2 - lysyl oxidase-like 2, Bmp6 - bone morphogenetic protein 6, Bmp4 - bone morphogenetic protein 4, Chadl - chondroadherin-like, Adamts7 - a disintegrin-like and metallopeptidase (reprolysin type) with thrombospondin type 1 motif, 7, Mustn1 - musculoskeletal, embryonic nuclear protein 1, Anxa2 - annexin a2]
GO: 0006820	anion transport	4.04E-02	[Slc16a13 - solute carrier family 16 (monocarboxylic acid transporters), member 13, Slc4a9 - solute carrier family 4, sodium bicarbonate cotransporter, member 9, Slc7a2 - solute carrier family 7 (cationic amino acid transporter, y+ system), member 2, Apoa4 - apolipoprotein a-iv, Slco1a4 - solute carrier organic anion transporter family, member 1a4, Aqp8 - aquaporin 8, Slc22a26 - solute

			carrier family 22 (organic cation transporter), member 26, Slc16a6 - solute carrier family 16 (monocarboxylic acid transporters), member 6, Slc16a11 - solute carrier family 16 (monocarboxylic acid transporters), member 11, Slc6a8 - solute carrier family 6 (neurotransmitter transporter, creatine), member 8]
GO: 0006953	acute-phase response	4.19E-02	[Orm2 - orosomucoid 2, Saa2 - serum amyloid a 2, Saa1 - serum amyloid a 1]
GO: 0014037	Schwann cell differentiation	4.73E-02	[Gpc1 - glypican 1, Erbb3 - v-erb-b2 erythroblastic leukemia viral oncogene homolog 3 (avian), Nab2 - ngfi-a binding protein 2, Egr2 - early growth response 2, Pmp22 - peripheral myelin protein 22]
GO: 0051186	cofactor metabolic process	4.97E-02	[Fasn - fatty acid synthase, Gpam - glycerol-3-phosphate acyltransferase, mitochondrial, Tkt - transketolase, Gpd2 - glycerol phosphate dehydrogenase 2, mitochondrial, Acot1 - acyl-coa thioesterase 1, Gpi1 - glucose phosphate isomerase 1, Cbr3 - carbonyl reductase 3, Pdss1 - prenyl (solanesyl) diphosphate synthase, subunit 1, Hmgcs1 - 3-hydroxy-3-methylglutaryl-coenzyme a synthase 1, Gnmt - glycine n-methyltransferase, Ahcy - s-adenosylhomocysteine hydrolase, Got1 - glutamate oxaloacetate transaminase 1, soluble, Pgam1 - phosphoglycerate mutase 1, Mthfd1l - methylenetetrahydrofolate dehydrogenase (nadp+ dependent) 1-like, Gpx4 - glutathione peroxidase 4, Acly - atp citrate lyase, Gch1 - gtp cyclohydrolase 1, Sardh - sarcosine dehydrogenase, Acot9 - acyl-coa thioesterase 9, Acacb - acetyl-coenzyme a carboxylase beta, Aadat - aminoadipate aminotransferase, Mms19 - mms19 (met18 s. cerevisiae), Gpx3 - glutathione peroxidase 3, Acot11 - acyl-coa thioesterase 11, Hscb - hscb iron-sulfur cluster co-chaperone homolog (e. coli), Vnn1 - vanin 1, Acsm5 - acyl-coa synthetase medium-chain family member 5, Slc23a2 - solute carrier family 23 (nucleobase transporters), member 2, Akr1c20 - aldo-keto reductase family 1, member c20, Hba-a1 - hemoglobin alpha, adult chain 1, Mthfd2 - methylenetetrahydrofolate dehydrogenase (nad+ dependent), methenyltetrahydrofolate cyclohydrolase, Gsta2 - glutathione s-transferase, alpha 2 (yc2), Acot6 - acyl-coa thioesterase 6, Gpd1l - glycerol-3-phosphate dehydrogenase 1-like, Taldo1 - transaldolase 1, Rpia - ribose 5-phosphate isomerase a, Dlat - dihydrolipoamide s-acetyltransferase (e2 component of pyruvate dehydrogenase complex), Far2 - fatty acyl coa reductase 2, Cth - cystathionase (cystathionine gamma-lyase), Ptges - prostaglandin e synthase, Gapdhs - glyceraldehyde-3-phosphate dehydrogenase, spermatogenic, Acss1 - acyl-coa synthetase short-chain family member 1, Dhtkd1 - dehydrogenase e1 and transketolase domain containing 1, Gapdh - glyceraldehyde-3-phosphate dehydrogenase, Acadsb - acyl-coenzyme a dehydrogenase, short/branched chain, Akr1c18 - aldo-keto reductase family 1, member c18, Pdhb - pyruvate dehydrogenase (lipoamide) beta, Khk - ketohexokinase, Mthfd1 - methylenetetrahydrofolate dehydrogenase (nadp+ dependent), methenyltetrahydrofolate cyclohydrolase, formyltetrahydrofolate synthase, Tdo2 - tryptophan 2,3-dioxygenase, Gstm5 - glutathione s-transferase, mu 5, Acss2 - acyl-coa synthetase short-chain family member 2, Gck - glucokinase, Gsta4 - glutathione s-transferase, alpha 4, Gm3839 - glyceraldehyde-3-phosphate dehydrogenase pseudogene, Gale - galactose-4-epimerase, udp, Abcb6 - atp-binding cassette, sub-family b (mdr/tap), member 6, Acnat2 - acyl-coenzyme a amino acid n-acyltransferase 2, Gstt2 - glutathione s-transferase, theta 2, Cs - citrate synthase, Pah - phenylalanine hydroxylase, G6pdx - glucose-6-phosphate dehydrogenase x-linked, Aldob - aldolase b, fructose-bisphosphate, Tat - tyrosine aminotransferase, Pgd - phosphogluconate dehydrogenase, Agpat6 - 1-acylglycerol-3-phosphate o-acyltransferase 6 (lysophosphatidic acid acyltransferase, zeta), Pklr - pyruvate kinase liver and red blood cell, Ppt1 - palmitoyl-protein thioesterase 1, Cyp1a1 - cytochrome p450, family 1, subfamily a, polypeptide 1, Ggt6 - gamma-glutamyltransferase 6, Cycs - cytochrome c, somatic, Apoa4 - apolipoprotein a-iv, Acaca - acetyl-coenzyme a carboxylase alpha, Atpif1 - atpase inhibitory factor 1, Ehhadh - enoyl-coenzyme a, hydratase/3-hydroxyacyl coenzyme a dehydrogenase, Aldh1l2 - aldehyde dehydrogenase 1 family, member l2, Mrps36 - mitochondrial ribosomal protein s36, Acsl5 - acyl-coa synthetase long-chain family member 5, Cyp2a5 - cytochrome p450, family 2, subfamily a, polypeptide 5]
